# Bifunctional Squaramide‐Catalyzed Oxidative Kinetic Resolution: Simultaneous Access to Axially Chiral Thioether and Sulfoxide

**DOI:** 10.1002/advs.202402429

**Published:** 2024-05-15

**Authors:** Wei Wen, Chang‐Lin Yang, Zhu‐Lian Wu, Dong‐Rong Xiao, Qi‐Xiang Guo

**Affiliations:** ^1^ Key Laboratory of Applied Chemistry of Chongqing Municipality Chongqing Key Laboratory of Soft‐Matter Material Manufacturing School of Chemistry and Chemical Engineering Southwest University Chongqing 400715 China; ^2^ School of Chemistry and Chemical Engineering Southwest University Chongqing 400715 China

**Keywords:** bifunctional squaramide, kinetic resolution, multiple chirality, organocatalysis, sulfoxidation

## Abstract

Axially chiral thioethers and sulfoxides emerge as two pivotal classes of ligands and organocatalysts, which have remarkable features in the stereoinduction of various asymmetric transformations. However, the lack of easy methods to access such molecules with diverse structures has hampered their broader utilization. Herein, an oxidative kinetic resolution for sulfides using a chiral bifunctional squaramide as the catalyst with cumene hydroperoxide as the terminal oxidant is established. This asymmetric approach provides a variety of axially chiral thioethers as well as sulfoxides bearing both axial and central chirality, with excellent diastereo‐ and enantioselectivities. This catalytic system also successfully extends to the kinetic resolution of benzothiophene‐based sulfides. Preliminary mechanism investigation indicates that the multiple hydrogen bonding interactions between the bifunctional squaramide catalyst and substrates play a crucial role in determining the enantioselectivity and reactivity.

## Graphical abstract

A bifunctional squaramide‐catalyzed enantioselective oxidation of sulfides is developed using cumene hydroperoxide as the terminal oxidant, which allows one to achieve a kinetic resolution of 2‐hydroxy‐2ʹ‐alkylthio‐1,1ʹ‐biaryls. This approach provides a variety of axially chiral thioethers and sulfoxides, which can be readily converted into valuable axially chiral compounds and sulfur‐containing ligands.

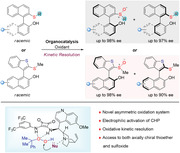

## 1 Introduction

**Figure 1:**
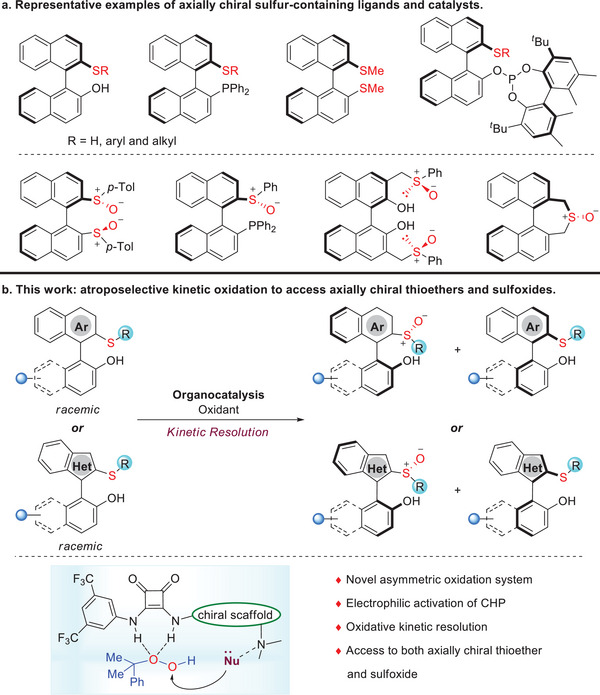
Examples and synthetic design of axially chiral sulfur‐containing compounds.

Axially chiral biaryls are prevalent structural motifs in natural products,^[^ ^1^ ^]^ bioactive molecules,^[^ ^2^ ^]^ and functional materials,^[^ ^3^ ^]^ as well as in privileged chiral catalysts or ligands in organic synthesis.^[^ ^4^ ^]^ Accordingly, the development of efficient enantio‐selective approaches for the synthesis of axially chiral biaryls has attracted extensive attention.^[^ ^5^, ^6^, ^7^ ^]^ In the last two decades, a large number of elegant synthetic methods have been established by transition‐metal catalysis^[^ ^6^ ^]^ or organocatalysis.^[^ ^7^ ^]^ In particular, axially chiral biaryl sulfur‐containing compounds, including thioethers and sulfoxides, represent an important class of versatile auxiliaries, ligands, and organocatalysts in asymmetric synthesis (**Figure** **1a**; for selected recent reviews and examples, see refs.[8, 9, 18]). However, they are typically prepared from optically active 1,1’‐bi‐2,2‘‐naphthol, resulting in very restricted structural diversity. In this context, the development of straightforward and efficient approaches would greatly widen the application scope of this particular class of chiral catalyst in asymmetric catalysis. Several elegant catalytic asymmetric methods for axially chiral thioethers and thiophenols have been reported.^[^ ^10^, ^11^, ^12^, ^13^, ^14^, ^15^ ^]^ For example, the organocatalytic asymmetric addition or substitution synthesis of axially chiral thioethers using thiophenol as a nucleophile developed by Smith^[^ ^11a^ ^]^ and Gustafson.^[^ ^11b‐d^ ^]^ The transitin‐metal‐catalyzed thioether‐directed atroposelective C─H functionalization strategy developed by Shi,^[^ ^12a,b^ ^]^ Ackermann,^[^ ^12c^ ^]^ Li,^[^ ^12d^ ^]^ and the Song groups.^[^ ^12e^ ^]^ The Hayashi^[^ ^13a^ ^]^ and Gu groups^[^ ^13b,c^ ^]^ reported the transition‐metal‐catalyzed ring‐opening/cross‐coupling of five‐membered distorted cyclic compounds for the asymmetric synthesis atropisomeric thioethers and thiophenols. Moreover, Zhao^[^ ^14a^ ^]^ and Chen^[^ ^14b‐c^ ^]^ developed the catalytic enantioselective electrophilic thiolation syntheses of axially chiral sulfur‐containing compounds. Recently, Hornillos and co‐workers developed an Rh‐catalyzed atroposelective reductive aldol reaction of sulfenylated biaryls to access axially chiral sulfur‐containing indole derivatives.^[^ ^15^ ^]^ On the other hand, the catalytic asymmetric synthesis of atropisomers bearing chiral sulfoxide is much less developed.^[^ ^16^ ^]^ Of course, the diastereoselective transformations of enantiopure sulfoxides offer an efficient route to the axially chiral sulfoxide congeners.^[^ ^17^ ^]^

Chiral 2‐hydroxy‐2ʹ‐thio‐1,1ʹ‐binaphthyl compounds are not only effective ligands for asymmetric catalysis, but also serve as important platform molecules for the stereoselective synthesis of other sulfur‐containing catalysts and ligands.^[^ ^9^, ^18^ ^]^ Yorimitsu and co‐workers successfully developed a strategy for metal‐free C─H/C─H cross‐coupling of aryl sulfoxides with phenols, which is used to construct these biaryls possess alkylthio and hydroxyl groups in close proximity, greatly simplifying access to this core framework and its derivatives.^[^ ^19^ ^]^ Unfortunately, the chiral synthesis of this type of atropisomeric biaryls has not been achieved. Considering that significant advancements have been made in the catalytic stereospecific oxidation of sulfides to access optically active sulfoxides.^[^ ^20^, ^21^ ^]^ We aim to develop a practical kinetic resolution strategy that relies on the enantioselective oxidation of sulfides to simultaneously access axially chiral thioethers and sulfoxides, as well as their structurally diverse derivatives, in high levels of enantioenrichment (Figure 1b).

Thus far, a number of methods for the enantioselective oxidation of sulfides catalyzed by chiral transition‐metal complexes and organocatalysts have been reported.^[^ ^20^, ^21^ ^]^ These great advances focused primarily on the construction of centrally chiral sulfoxides, while the application in the enantioselective construction of axially chiral molecules is still limited.^[^ ^16^ ^]^ Attempts were made to use these catalytic systems for the kinetic resolution of racemic 2ʹ‐(methylthio)−1,1ʹ‐naphthalene‐2‐ol (**1a**), but the results were not satisfactory. Clearly, a new catalytic oxidative system capable of promoting the kinetic resolution of 2‐hydroxy‐2ʹ‐alkylthio‐1,1ʹ‐binaphthyl compounds with a high level of enantioselectivity and a broad scope is urgently needed to streamline the synthesis of the privileged axially chiral biaryls.

The efficacy and synthetic versatility of asymmetric organocatalysis have contributed enormously to the field of organic synthesis.^[^ ^22^ ^]^ Bifunctional chiral tertiary amine‐squaramides have exhibited great versatility and remarkable stereoinduction capability in facilitating many transformations, attributed to their ability for synergistic dual acid and base activation.^[^ ^23^ ^]^ However, there is currently a lack of reports on the utilization of chiral tertiary amine‐squaramide catalysts for enantioselective oxidation of sulfur atoms.^[^ ^24^ ^]^ We envisioned that squaramide electrophilically activated the peralcohol by hydrogen‐bonding interactions and directed it toward the nucleophilic S atom in an enantioselective manner. In this study, we present the first application of chiral tertiary amine‐squaramides in enantioselective oxidation of a heteroatom, providing a metal‐free practical kinetic resolution strategy for the synthesis of highly enantioenriched 2‐hydroxyl‐2′‐thio axially chiral compounds and their structurally diverse derivatives (Figure 1b). It is worth noting that this reaction not only offers axially chiral thioethers, but also generates sulfoxide derivatives with both axial and S‐central chirality. In fact, catalytic asymmetric synthesis of atropisomers bearing multiple chiral elements has become an emerging area, as the combination of multiple chiral elements will endow compounds with new properties, which provides opportunities for their new applications.^[^ ^25^ ^]^ Moreover, this method is also suitable for the highly efficient acquisition of benzothiophene‐based atropsiomers, and currently there are still fewer methods available for the synthesis of such axially chiral heteroaryl molecules (for selected recent reviews and examples, see refs. [26, 27]).

## 2 Results and Discussion

### 2.1 Optimizing Reaction Conditions

We commenced our investigation by using racemic 2ʹ‐(methylthio)−1,1ʹ‐naphthalene‐2‐ol **1a** as a model substrate (**Table** **1**). At the outset, the known oxidation systems based on transition metals or chiral Brønsted acid catalysis^[^ ^21^ ^]^ were utilized to verify the kinetic separation reaction of (±)−**1a**. Indeed, the kinetic separation occurred smoothly and product **3a** was given; however, the enantioselectivity was only low to moderate, even after extensive conditions optimization (see the Supporting Information for details). Hence, we turned our attention to a newly designed oxidation system based on a chiral tertiary amine‐squaramide catalyst. The tertiary amine‐squaramide **2a** was used as a catalyst to promote the oxidative kinetic resolution of (±)−**1a** with *tert*‐butyl hydroperoxide (TBHP) as an oxidant, giving in 58% ee of sulfoxide **3a** as well as 23% ee of the remained **1a**, corresponding to the selectivity factor (*s*) of 4.7 (entry 1). The related urea **2b** and thiourea **2c** were also examined, and notable reductions in conversion and selectivity were observed (entries 2 and 3). Encouraged by the initial result, we extensively screened the tertiary amine‐squaramide catalysts (entries 4–9). The results indicate that the backbone of the catalyst was sensitive to the reaction. By contrast, cinchona alkaloid‐based catalysts **2g**‐**2i** exhibited a more satisfactory kinetic resolution performance (entries 7–9). Subsequently, **2g** was utilized as a catalyst to probe the oxidants (entries 10 and 11). To our delight, when the cumene hydroperoxide (CHP) was used, it significantly improved the outcome, furnishing **3a** in 45% yield with 84% ee and the recovered **1a** in 52% yield with 94% ee, and the *s*‐factor enhanced to 40 (entry 11). Initially, MgSO_4_ was added to remove water present in THBP solution or aqueous hydrogen peroxide. Further research found that the absence of MgSO_4_ had no marked effect on the reaction when using CHP as an oxidant (entry 12). The effect of solvents was also studied (entries 13–18), indicating that the 1,1,2,2‐tetrachloroethane (TTCE) is the optimal solvent (entry 15). Finally, the optimal reaction conditions were determined by subtly adjusting other parameters (see the Supporting Information for details). Under these conditions, the oxidized product **3a** and the remaining **1a** both exhibit high enantioselectivity (both 93% ee), with a selectivity factor of 94 (entry 20). Notably, in all cases, the sulfoxide **3a** was obtained as a single diastereoisomer.

**Table 1:** Evaluation of catalysts and optimization of reaction conditions.

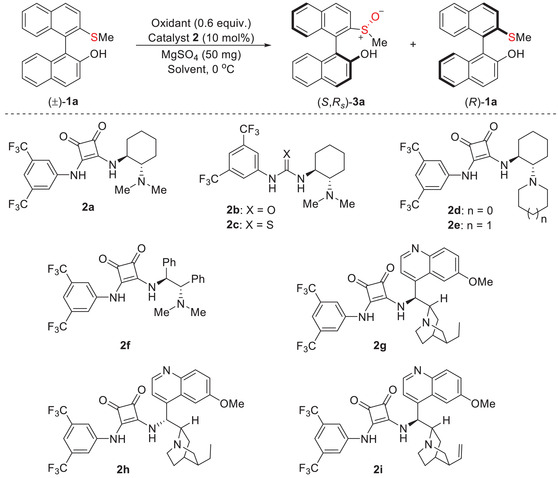									
entry^a)^	cat. 2	t [h]	oxidant	solvent	**3a**		**1a**		*s* ^d)^
					yield [%]^b)^	ee_p_ [%]^c)^	yield [%]^b)^	ee_s_ [%]^c)^
1	**2a**	20	TBHP	CH_2_Cl_2_	26	58	68	23	4.7
2	**2b**	27	TBHP	CH_2_Cl_2_	6	25	87	2	1.7
3	**2c**	58	TBHP	CH_2_Cl_2_	6	43	88	2	2.6
4	**2d**	18	TBHP	CH_2_Cl_2_	17	57	80	11	4.1
5	**2e**	27	TBHP	CH_2_Cl_2_	8	28	86	3	1.8
6	**2f**	28	TBHP	CH_2_Cl_2_	18	‐39	78	‐7	2.4
7	**2g**	27	TBHP	CH_2_Cl_2_	29	79	70	31	12
8	**2h**	23	TBHP	CH_2_Cl_2_	26	‐71	67	‐24	7.4
9	**2i**	12	TBHP	CH_2_Cl_2_	20	79	76	20	10
10	**2g**	17	30% aq. H_2_O_2_	CH_2_Cl_2_	44	62	56	50	6.9
11	**2g**	16	CHP	CH_2_Cl_2_	45	84	52	94	40
12^e)^	**2g**	16	CHP	CH_2_Cl_2_	43	86	56	94	47
13^e)^	**2g**	18	CHP	DCE	47	84	52	93	39
14^e)^	**2g**	12	CHP	CHCl_3_	48	82	52	98	46
15^e)^	**2g**	12	CHP	TTCE	50	86	46	96	52
16^e)^	**2g**	13	CHP	EtOAc	15	87	82	12	16
17^e)^	**2g**	22	CHP	THF	10	70	86	8	6
18^e)^	**2g**	17	CHP	PhCH_3_	54	58	43	88	10
19^e)^, ^f)^	**2g**	10	CHP	TTCE	41	94	55	84	86
20^e)^, ^f)^	**2i**	11	CHP	TTCE	46	93	48	93	94

^a)^ Reactions were performed with (±)−**1a** (0.1 mmol), oxidant (0.06 mmol), catalyst **2** (0.01 mmol), and MgSO_4_ (50 mg) in solvent (1 mL) at 0 °C for a specified time

^b)^ Isolated yield

^c)^ Determined by chiral‐phase HPLC analysis

^d)^ The selectivity factor was calculated as *s = *ln[(1‐*C*)(1‐ee_s_)]/ln[(1‐*C*)(1+ee_s_)], *C = *ee_s_/(ee_s_+ee_p_)

^e)^ Without MgSO_4_

^f)^ With 0.05 mmol CHP, in TTCE (0.5 mL) at ‐10 °C. TBHP = *tert*‐butyl hydroperoxide (70% solution in water); CHP = cumene hydroperoxide; TTCE = 1,1,2,2‐tetrachloroethane.

### 2.2 Exploring the Substrate Scope

With these satisfying results, we set out to explore the substrate generality of this procedure. As shown in **Table** **2**, studies on the effect of substituents on the skeleton of (±)−**1** revealed that the reaction was also efficient for substrates with electron‐donating (**1b**, **1d**) and withdrawing (**1c**, **1e**) substituents at the C8 and C7 site with good yields and good to excellent enantioselectivities. Meanwhile, different substituents at the C6 position were examined, including cyano, formyl, ester, phenyl and substituted phenyl groups, all of which were well tolerated under the optimal conditions, providing the corresponding (*S*,*R_s_*)−**3** and the recovered (*R*)−**1** both with high enantioselectivities (**1f**–**1q**). We also tested the C2‐bromo substituted **1r** and only observed 75% ee in the produced **3r** while 70% ee in the recovered **1r**. The absolute configuration of the oxidized product **3a** was determined to be (*S*,*R_s_*) by X‐ray diffraction analysis.^[^ ^28^ ^]^ The recovered **1a** was then deduced to have the *R‐*configuration.

**Table 2:** Substrate generality of kinetic resolution of biaryl thioethers **1**.

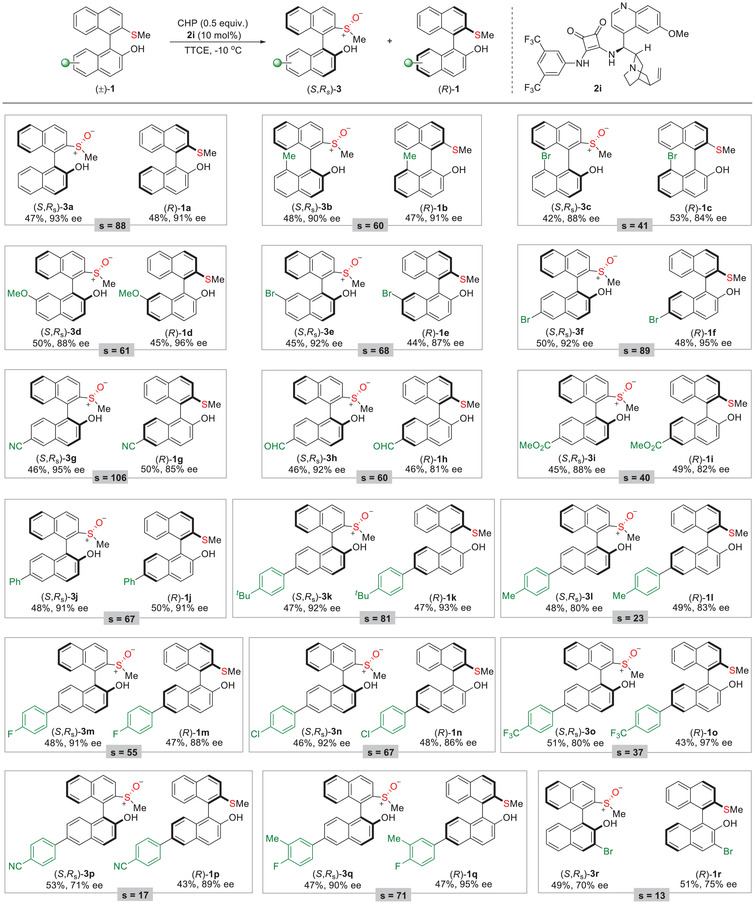 ^a)^, ^b)^

^a)^ Reactions were performed with (±)−**1** (0.20 mmol), CHP (0.10 mmol), and catalyst **2i** (0.02 mmol) in TTCE (1 mL) at ‐10 °C. Reported yields are for the isolated product, and ee values were determined by chiral‐phase HPLC analysis. The selectivity factor was calculated as *s* = ln[(1‐C)(1‐ee(**1**))]/ln[(1‐C)(1+ee(**1**))], C = ee(**1**)/(ee(**3**)+ee(**1**))

^b)^ For all the sulfoxide products **3**, dr was >20:1, as determined by ^1^HNMR analysis.

To further probe the diversity of this transformation, the current reaction system was extended to the biaryl derivatives of phenol (**4a** and **4b**) and 1‐naphthalenol (**4c**) with moderate results (**Table** **3**). Subsequently, a series of alkyl groups other than the methyl group in the thioether moiety were also examined. Surprisingly, the increased steric resistance did not affect the activity of the reaction, and these substrates **4d**–**4f** were feasible with this kinetic resolution, demonstrating a selectivity factor of up to 47.

**Table 3:** Substrate generality of kinetic resolution of biaryl thioethers **4**.

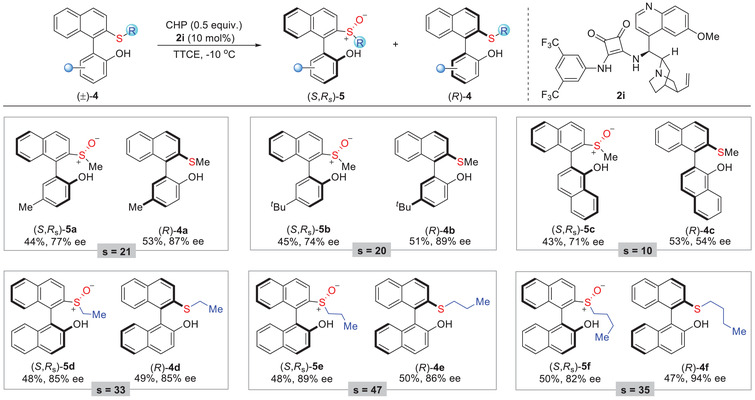 ^a)^, ^b)^

^a)^ Reactions were performed with (±)−**4** (0.20 mmol), CHP (0.10 mmol), and catalyst **2i** (0.02 mmol) in TTCE (1 mL) at ‐10 °C. Reported yields are for the isolated product, and ee values were determined by chiral‐phase HPLC analysis. The selectivity factor was calculated as *s* = ln[(1‐C)(1‐ee(**4**))]/ln[(1‐C)(1+ee(**4**))], C = ee(**4**)/(ee(**5**)+ee(**4**))

^b)^ For all the products **5**, dr was >20:1, as determined by ^1^HNMR analysis.

Currently, efficient access to atropisomers containing five‐membered heterobiaryls remains as an intractable issue. Next, we explored a class of benzothiophene‐aryl type biaryls as substrates, and the reaction rate dropped seriously for the racemic **6a** when standard conditions were applied (**Table** **4**). Delightedly, a promising conversion rate was restored by increasing the reaction temperature, oxidized product **7a** was obtained in 43% yield with 93% ee and (*R*)−**3a** was recovered in 57% yield with 80% ee. With these encouraging results, the influence of substituent and substituted pattern was investigated. A range of analogues with C6, C7, and C8‐substituted 2‐naphthol moieties were also amenable substrates, and the kinetic resolution proceeded smoothly to give the sulfoxide products **7b**–**7j** with 83–98% ee as well as the intact **6b**–**6j** with 72–90% ee. Wherein the substrate **6i** bearing a cyano group at C6 position was not well compatible in this system, and the enantiopurity of product **7i** and intact **6i** was dropped to 83% ee and 72% ee, respectively. Unfortunately, as for benzofuran‐aryl and indol‐aryl type biaryl analogues only trace amounts of the required products were detected (**8**–**10**).

**Table 4:** Kinetic resolution of benzothiophene‐aryl substrates.

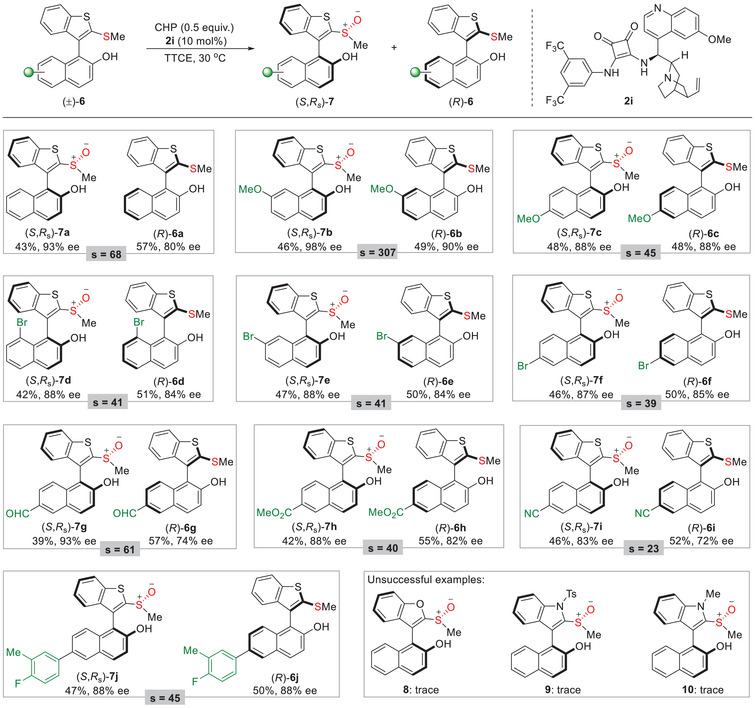 ^a)^, ^b)^

^a)^ Reactions were performed with (±)−**6** (0.20 mmol), CHP (0.10 mmol), and catalyst **2i** (0.02 mmol) in TTCE (1 mL) at 30 °C. Reported yields are for the isolated product, and ee values were determined by chiral‐phase HPLC analysis. The selectivity factor was calculated as *s* = ln[(1‐C)(1‐ee(**6**))]/ln[(1‐C)(1+ee(**6**))], C = ee(**6**)/(ee(**7**)+ee(**6**))

^b)^ For all the sulfoxide products **7**, dr was >20:1, as determined by ^1^HNMR analysis.

### 2.3 Synthetic Applications

**Scheme 1:**
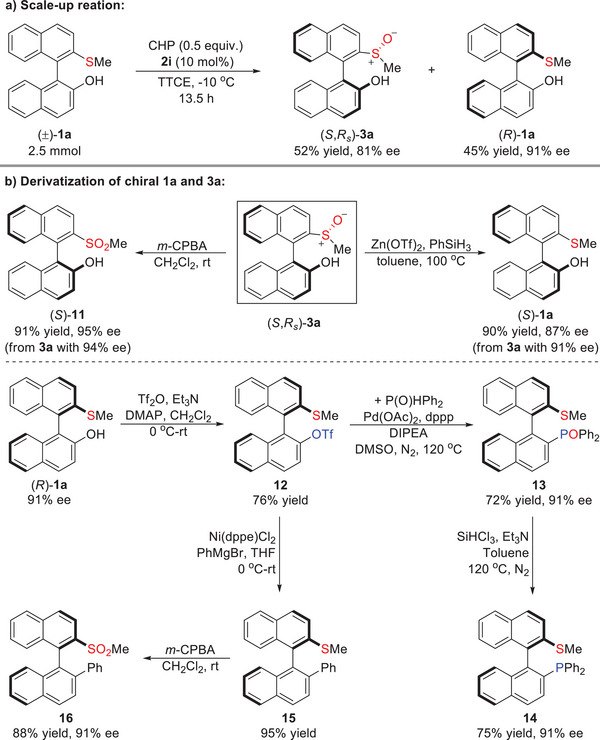
Scale‐up reaction and derivatization of chiral products.

To demonstrate the potential utility of this reaction, we performed a scale‐up reaction and derivatization of axially chiral products **1a** and **3a** (**Scheme** **1**). Gratifyingly, the protocol could be scaled up under the standard conditions and afforded product **3a** in 52% yield with 81% ee and recovered (*R*)−**1a** in 45% yield with 91% ee (Scheme 1a). Following this, the transformations of the obtained **3a** were carried out (Scheme 1b). Sulfone **11** was given by treating **3a** with *m*‐CPBA in 91% yield without the erosion of enantioselectivity. (*S*)−**1a** was also accessible from the (*S*,*R_s_*)−**3a** through high efficiency reduction of sulfoxide by PhSiH_3_. Additionally, starting from the remaining (*R*)−**1a**, the reaction with Tf_2_O could give triflate **12**. Compound **12** could undergo Kumada cross‐coupling with Grignard reagent to generate biaryl thioether **15** under the catalysis of Ni(dppe)_2_Cl_2_, which could be oxidized efficiently to furnish the chiral sulfone **16**. The compound **12** could also be transformed to **14**, a potential axially chiral (S,P)‐ligand,^[^ ^9e^ ^]^ by successive palladium‐mediated C‐P cross‐coupling and reduction of *P*‐oxide (Scheme 1b).

### 2.4 Mechanistic Studies

**Scheme 2:**
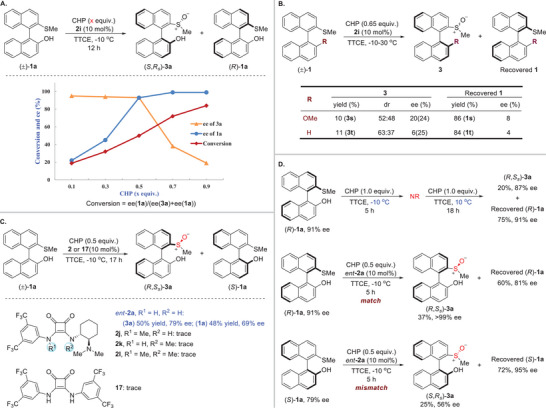
Mechanistic investigations.

To gain mechanistic insights into this organocatalytic system in the oxidative kinetic resolution of racemic biaryl thioethers, in particular the origin of the high diastereoselectivity of the transformation, a series of control experiments were performed (**Scheme** **2**). At first, the reaction of (±)−**1a** with different equivalents of CHP was monitored (Scheme 2A). It was found that the ee values of the recovered **1a** increases with increasing equivalents of CHP, indicating that one of the enantiomers of **1a** was consumed more rapidly, suggesting a kinetic resolution process. To our surprise, when the loading of CHP exceeded 0.5 equivalents, the enantioselectivities of product **3a**, which has both axial and central chirality, continued to decrease, yet always obtained as diastereomerically pure. It indicated that in the case of insufficient and excessive CHP, the absolute configuration of the S‐stereogenic center generated in sulfoxide **3a** is the opposite. We speculated that the sulfoxide group as a strong hydrogen‐bond acceptor for interaction with the hydroxyl group resulted in the formation of the inherently preferred diastereomer. This was also supported by control experiments that exhibited obviously low diastereoselectivity in the absence of a free hydroxyl group in substrate **1** (Scheme 2B).

**Figure 2:**
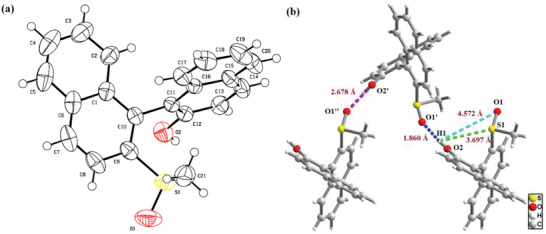
Description of the crystal structures of (*S*,*R_s_*)−**3a**. a) X‐ray crystal structure of (*S*,*R_s_*)−**3a**. b) Crystal‐packing diagram of three units of (*S*,*R_s_*)−**3a**.

We obtained single‐crystal of (*S*,*R_s_*)−**3a** for structure determination (**Figure** **2**a). Insight into the role of the hydrogen bonds in the origin of diastereoselectivity in the oxidation of **3a** may be obtained from the X‐ray crystallographic analysis below. As shown in Figure 2b, a significant hydrogen bonding interaction between adjacent molecules, that is, the hydrogen atom of the hydroxyl group of one molecule with the oxygen atom of the sulfoxide group of the next molecule. The typical hydrogen bond O2─H1···O1ʹ has a length of 1.860 Å. It is worth noting that Lucchi et al. once reported a similar case of (*R*)−**1a** being diastereospecifically oxidized by *m*‐CPBA.^[^ ^29^ ^]^ They attributed the stereochemistry of the oxidation product **3a** to internal hydrogen bonding. Interestingly, the crystal structure showed that (*S*,*R_s_*)−**3a** lacked intramolecular hydrogen bonding in its solid state. This may be due to the spatial repulsion that prevented the hydroxyl group from approaching the S─O bond located within the same molecule (Figure 2b).

Furthermore, we were interested in the activation mode of chiral tertiary amine‐squaramide catalysts on the substrates. The presence of the free hydroxy group in substrate **1** evidently plays a crucial role in both reactivity and stereoselectivity, possibly through hydrogen bonding with the catalyst (Scheme 2B). We then studied the effect of the functional groups of tertiary amine‐squaramide catalyst **2** on the observed reactivity (Scheme 2C). In the presence of *ent*‐**2a**, the kinetic resolution proceeded smoothly and exhibited good stereoselectivity. However, partial or complete methylation of the two NH groups of the squaramide in *ent*‐**2a** both led to reaction not occurring, confirming the cooperative catalysis of the NH moieties as hydrogen bonding donor. The power of the synergistic activation specifically by a bifunctional tertiary amine and squaramide was demonstrated when the squaramide **17** was also ineffective for the reaction.

Finally, we observed the matching characteristics between the substrates and the catalyst (Scheme 2D). (*R*)−**1a** with 91% ee conducted the oxidation process without a catalyst. In this case, no background reaction occurred. Then, under the optimal conditions, the enantiomers (*R*)‐ and (*S*)−**1a** were treated with catalyst *ent*‐**2a** for 5 h, respectively. The result indicated that the (*R*)−**1a** was chiral matched with the catalyst *ent*‐**2a** and was preferentially converted to generate the (*R*,*S_s_*)−**3a**. The (*R*)−**1a** was chiral mismatched with *ent*‐**2a**, with a slower oxidation rate compared to the (*S*)−**1a**, and was transformed to the opposite enantiomer (*S*,*R_s_*)−**3a**.

**Figure 3:**
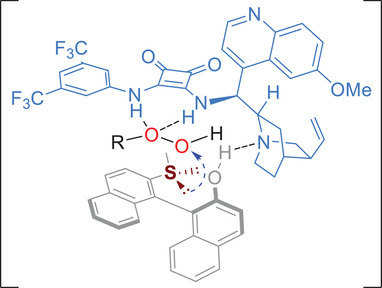
Plausible stereochemical model.

On the basis of the experimental results and previous reports on synergistic activation of bifunctional chiral tertiary amine‐squaramide, a plausible model was proposed (**Figure** **3**). The CHP was electrophilically activated by the squaramide group of the catalyst **2i** through double hydrogen bonds, while the phenolic proton on the naphthol unit was interacted with the tertiary amine unit of catalyst, thus facilitating a stereoselective nucleophilic attack of the sulfide on the CHP.

## 3 Conclusion

In summary, we have established an enantioselective oxidative kinetic resolution method to access a class of sulfur‐containing atropisomeric platform molecules. This strategy is based on a novel oxidation system by utilizing a chiral bifunctional squaramide catalyst and cumene hydroperoxide. This transformation resulted in biaryl sulfoxides bearing both axial and *S*‐central chirality, which have high atroposelectivities and excellent diastereoselectivities, as well as the remaining axially chiral thioethers, which have high enantiocontrol. Furthermore, the developed catalytic system is also applicable to the kinetic resolution of benzothiophene‐aryl type substrates. Importantly, the obtained products could be readily converted into valuable axially chiral compounds in simple steps, providing a handle for the synthesis of the chiral thioether and sulfoxide ligands and catalysts.

## Conflict of Interest

The authors declare no conflict of interest.

## Supporting information

Supporting Information

## Data Availability

The data that support the findings of this study are available in the supplementary material of this article.
