# Supplementary material for: Bifunctional Squaramide‐Catalyzed Oxidative Kinetic Resolution: Simultaneous Access to Axially Chiral Thioether and Sulfoxide
Source: Adv Sci (Weinh). 2024 May 15;11(28):2402429. doi: 10.1002/advs.202402429 (PMC11267355; doi:10.1002/advs.202402429)
Supplement: Supplementary file 1 — Supporting Information [file ADVS-11-2402429-s001.pdf]

ADVANCED SCIENCE
Open Access

Supporting Information

for *Adv. Sci.*, DOI 10.1002/advs.202402429

Bifunctional Squaramide-Catalyzed Oxidative Kinetic Resolution: Simultaneous Access to Axially Chiral Thioether and Sulfoxide

Wei Wen, Chang-Lin Yang, Zhu-Lian Wu, Dong-Rong Xiao and Qi-Xiang Guo**

Supporting Information

Bifunctional Squaramide-Catalyzed Oxidative Kinetic Resolution: Simultaneous Access to Axially Chiral Thioether and Sulfoxide

Wei Wen*[a], Chang-Lin Yang[b], Zhu-Lian Wu[b] Dong-Rong Xiao[b], Qi-Xiang Guo*[a]

wenwei1989@swu.edu.cn; qxguo@swu.edu.cn

[a] Key Laboratory of Applied Chemistry of Chongqing Municipality, and Chongqing Key Laboratory of Soft-Matter Material Chemistry and Function Manufacturing, School of Chemistry and Chemical Engineering, Southwest University, Chongqing, 400715.

[b] School of Chemistry and Chemical Engineering, Southwest University, Chongqing, 400715.

Table of Contents

1. General data S2
2. Reaction condition optimization for the kinetic resolution S3
3. General procedure for the kinetic resolution of (±)-**1** and (±)-**4** S8
4. General procedure for the kinetic resolution of (±)-**6** S40
5. Scale-Up Reaction and Derivatization of Chiral Products S53
6. Crystal data and structure of compound (*S*, R_s)-**3a** S60
7. Control experiment for mechanism investigation S61
8. References S66
9. Copies of ^{1}H NMR and ^{13}C NMR spectra S67

1. General data

Solvents for reactions were dried appropriately before use: toluene, THF and Et_2O were dried by refluxing with sodium and benzophenone as indicator, CH_2Cl_2 was dried by refluxing with CaH_2. All other reagents were directly used as purchased from Aladdin, Adamas-beta® and Energy Chemical.

Unless otherwise noted, commercial reagents were used as received and all reactions were carried out directly in air atmosphere. All reactions were monitored by TLC with silica gel coated plates. ^{1}H NMR and ^{13}C NMR spectra were recorded on Bruker Avance 600 or 400 MHz spectrometer. Chemical shifts (δ) are reported in ppm from tetramethylsilane (TMS) with the solvent resonance as the internal standard. Proton signal multiplicities are given as s(singlet), d (doublet), t (triplet), q (quartet), m (multiplet), br (broad) or a combination of them. *J*-values are in Hz. HRMS (ESI-Q-TOF) spectra were recorded on Bruker Impact-II mass spectrometer. Enantiomer ratios were determined by HPLC (Chiralpak IA-H, OJ-H, IC-H, OD-H columns were purchased from Daicel Chemical Industries, LTD). Optical rotations were determined at λ = 589 nm (sodium D line) by using a Rudolph-API automatic polarimeter. The biaryl substrates (±)-**1**, (±)-**4**, and (±)-**6**[1] and bifunctional squaramide catalysts **2d-2l**[2-6] were prepared according to the literature.

2. Reaction condition optimization for the kinetic resolution

2.1 Reaction condition optimization for the kinetic resolution catalyzed by transition metals

Table S1: Ligands screening[a]

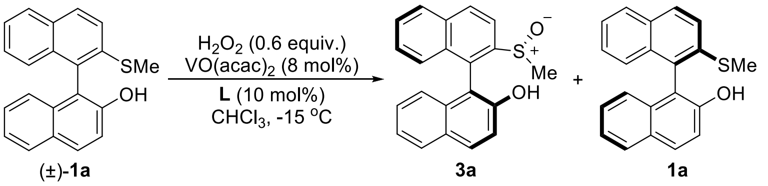

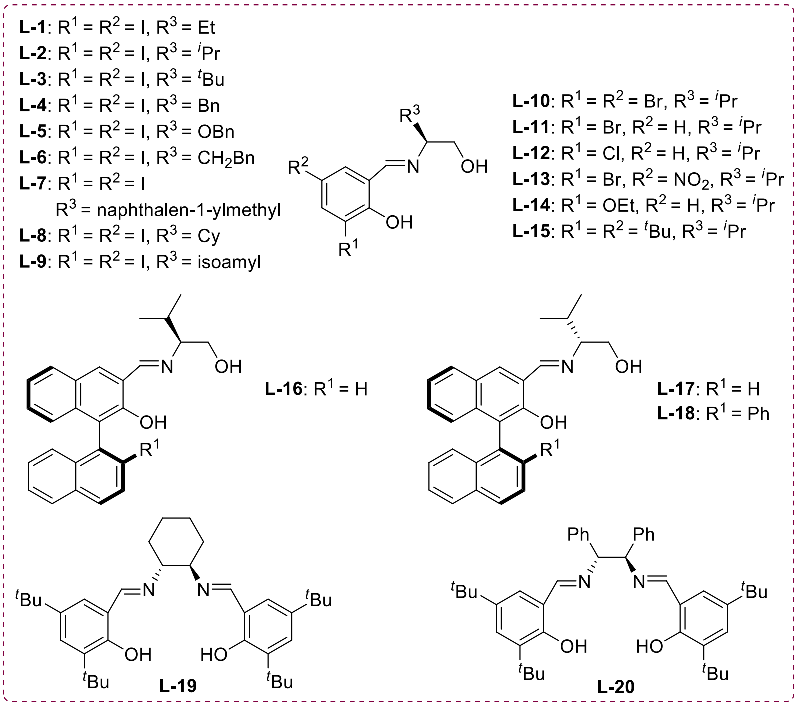

entry	L	time (h)	1a		3a	
			yield (%)[b]	ee (%)[c]	yield (%)[b]	ee (%)[c]
1	**L-1**	3	42	53	57	45
2	**L-2**	4	38	80	60	50
3	**L-3**	9	36	60	60	40
4	**L-4**	5	47	36	44	50
5	**L-5**	4	40	48	55	35
6	**L-6**	2.5	67	18	31	45
7	**L-7**	12	58	15	41	21
8	**L-8**	10	45	45	54	41
9	**L-9**	3	38	63	57	60
10	**L-10**	16	56	40	41	56
11	**L-11**	7	39	78	55	57

12	**L-12**	6.5	41	58	51	60
13	**L-13**	4	11	60	88	14
14	**L-14**	4	26	79	70	20
15	**L-15**	12	25	96	70	35
16	**L-16**	16	55	50	42	56
17	**L-17**	10	38	-66	58	-30
18	**L-18**	7	40	-53	56	-42
19	**L-19**	12	36	0	56	0
20	**L-20**	12	69	8	27	10

[a]Unless noted otherwise, reactions were performed with (±)-**1a** (0.1 mmol), VO(acac)$_2$ (0.008 mmol), **L** (0.01 mmol) and 30% aq. H_2O_2 (0.06 mmol) in $CHCl_3$ (1.0 mL) at -15 °C. [b]Isolated yield. [c]Determined by chiral HPLC analysis.

Table S2: Transition metals screening[a]

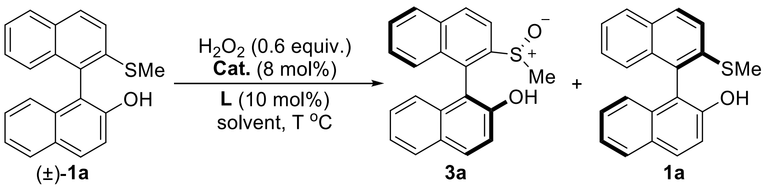

entry	L	Cat.	solvent	T (°C)	1a		3a	
					**yield (%)[b]**	**ee (%)[c]**	**yield (%)[b]**	**ee (%)[c]**
1	**L-15**	Fe(acac)$_3$	DCM	-15	66	6	30	28
2	**L-20**	Fe(acac)$_3$	DCM	0	80	0	10	0
3	**L-19**	$FeCl_3$	PhMe	r.t.	48	0	31	0
4	**L-15**	Mn(OTf)$_2$	DCM	-15	95	0	trace	-
5	**L-19**	Mn(OTf)$_2$	MeCN	r.t.	65	0	15	0
6	**L-20**	Ti(O^{i}Pr)$_4$	DCM	0	30	0	59	0

[a]Unless noted otherwise, reactions were performed with (±)-**1a** (0.1 mmol), **Cat.** (0.008 mmol), **L** (0.01 mmol) and 30% aq. H_2O_2 (0.06 mmol) in solvent (1.0 mL) at T °C. [b]Isolated yield. [c]Determined by chiral HPLC analysis.

2.2 Reaction condition optimization for the kinetic resolution catalyzed by chiral Brønsted acids

Table S3: Chiral Brønsted acid catalysts screening[a]

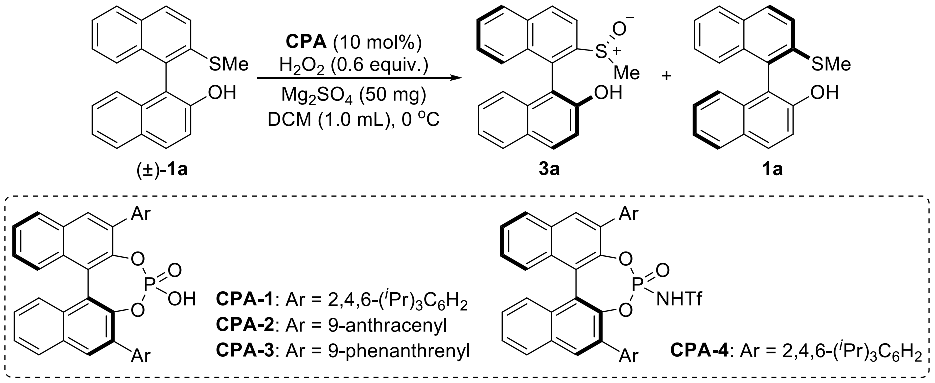

entry	CAP	time (h)	1a		3a	
			yield (%)[b]	ee (%)[c]	yield (%)[b]	ee (%)[c]
1	**CAP-1**	16.5	44	1	51	1
2	**CAP-2**	24	80	6	16	40
3	**CAP-3**	24	68	1	30	1
4	**CAP-4**	24	69	7	26	21

[a]Unless noted otherwise, reactions were performed with (±)-**1a** (0.1 mmol), **CAP** (0.01 mmol) and 30% aq. H_2O_2 (0.06 mmol) in DCM (1.0 mL) at 0 ℃. [b]Isolated yield. [c]Determined by chiral HPLC analysis.

2.3 Reaction condition optimization for the kinetic resolution catalyzed by bifunctional squaramides

Table S4: Optimization of reactant concentration and CHP equivalent[a]

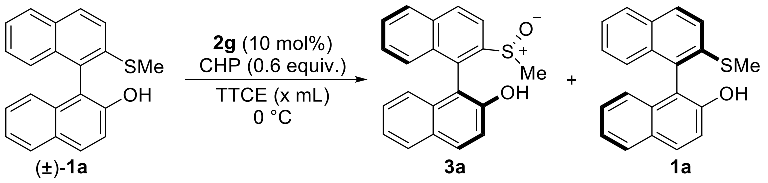

entry	TTCE (x mL)	time (h)	1a yield (%)[b]	1a ee (%)[c]	3a yield (%)[b]	3a ee (%)[c]
1	1	11.5	46	96	50	86
2	2	22	68	57	27	91
3	0.5	15.5	45	97	50	83
4[d]	0.5	7	51	91	46	91
5[d]	0.33	5	50	90	46	88

[a]Unless noted otherwise, reactions were performed with (±)-**1a** (0.10 mmol), **2g** (0.01 mmol) and CHP (0.06 mmol), in TTCE (x mL) at 0 ℃. [b]Isolated yield. [c]Determined by chiral HPLC analysis. [d]0.5 equiv. CHP.

Table S5: Reaction temperature screening[a]

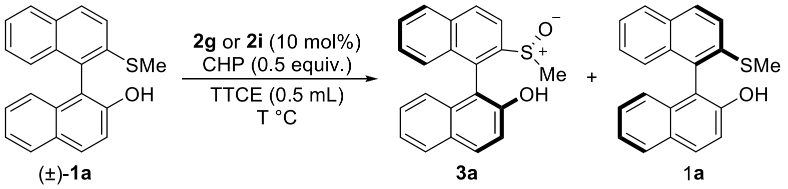

entry	T (℃)	time (h)	1a yield (%)[b]	1a ee (%)[c]	3a yield (%)[b]	3a ee (%)[c]
1	0	7	52	91	46	91
2	-10	10	55	84	41	94
3	-20	15	53	85	44	93
4	-30	16	49	88	47	91
5[d]	-10	11	48	93	47	93

[a]Unless noted otherwise, reactions were performed with (±)-**1a** (0.10 mmol), **2g** (0.01 mmol) and CHP (0.05 mmol) in TTCE (0.5 mL) at T ℃. [b]Isolated yield. [c]Determined by chiral HPLC analysis. [d]Using **2i**.

Table S6: Temperature optimization for the kinetic resolution of (±)-6a[a]

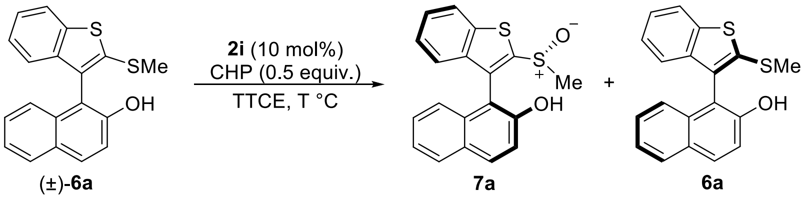

entry	T (°C)	time (h)	6a		7a	
			yield (%)[b]	ee (%)[c]	yield (%)[b]	ee (%)[c]
1	-10	17	69	44	28	92
2	0	18	64	63	35	93
3	10	20	59	69	40	93
4	30	24	54	81	43	93

[a]Unless noted otherwise, reactions were performed with (±)-**6a** (0.20 mmol), **2i** (0.02 mmol) and CHP (0.10 mmol) in TTCE (1.0 mL) at T °C. [b]Isolated yield. [c]Determined by chiral HPLC analysis.

3. General procedure for the kinetic resolution of (±)-1 and (±)-4

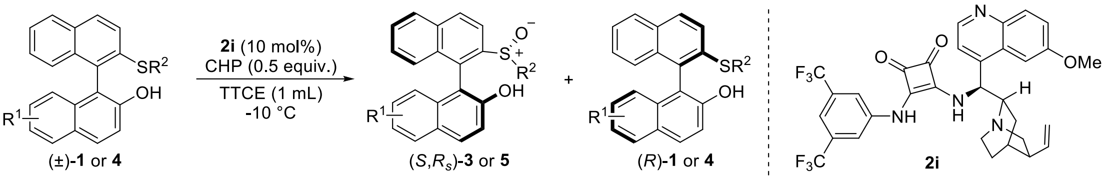

A dry Schlenk tube was charged with (±)-**1** (0.2 mmol), catalyst **2i** (12.6 mg, 0.02 mmol) and TTCE (1 mL). The mixture was cooled to -10 ℃ and stirred for 5 min, then the CHP (19 mg, 0.1 mmol) was added and the stirring was maintained at -10 ℃ for corresponding time. The reaction was quenched with saturated Na_2SO_3 solution (2 mL) and extracted with CH_2Cl_2 (5 mL × 3). The combined organic phase was washed with saturated NH_4Cl solution (5 mL × 2), dried over Na_2SO_4 and concentrated. The residue was purified by column chromatography on silica gel eluted with petroleum ether/ethyl acetate (5:1 to 1:2) to afford the pure product.

(*S*)-2'-((*R*)-methylsulfinyl)-1,1'-binaphthyl-2-ol (3a):

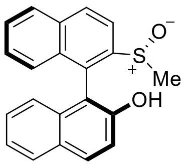

White solid (31.2 mg, 47%, -10 °C for 13 h); m.p. = 225-227 °C; R_f = 0.24 (petroleum ether/ethyl acetate = 1:2); the enantiomeric excess was determined to be 93% by HPLC analysis on Daicel Chirapak IA-H column (hexane/isopropanol = 90/10, flow rate 0.8 mL/min, T = 30 °C), UV 254 nm, t_R(major) 13.710 min, t_R(minor) 12.058 min; $[\alpha]_D^{25}$ = +221.26 (*c* = 0.356, $CHCl_3$); **^{1}H NMR (600 MHz, DMSO-d_6)** δ 10.02 (s, 1H), 8.33 (d, J = 8.7 Hz, 1H), 8.17 – 8.09 (m, 2H), 8.00 (d, J = 8.9 Hz, 1H), 7.93 (d, J = 8.0 Hz, 1H), 7.62 (t, J = 7.4 Hz, 1H), 7.40 (t, J = 7.7 Hz, 2H), 7.29 – 7.23 (m, 2H), 7.10 (d, J = 8.4 Hz, 1H), 6.63 (d, J = 8.4 Hz, 1H), 2.21 (s, 3H); **^{13}C NMR (151 MHz, DMSO-d_6)** δ 153.00, 143.74, 134.76, 134.13, 132.42, 132.27, 131.02, 130.06, 129.37, 128.98, 128.25, 127.94, 127.76, 127.49, 126.24, 123.80, 123.32, 119.66, 118.89, 113.65, 42.38; **HRMS(ESI)**: Calcd. for $C_{21}H_{15}O_2S[M-H]^-$ 331.0798; found 331.0795.

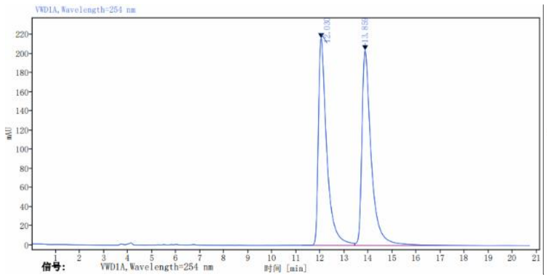

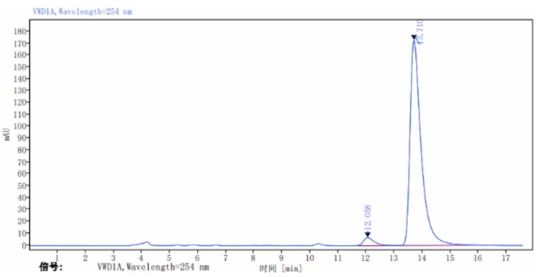

Peak	RetTime [min]	Type	Width[min]	Area[mAU*s]	Height[mAU]	Area%
	12.030	BV	2.2002	5253.3399	216.0845	48.7130
	13.859	VB	5.5531	5530.9245	203.5831	51.2870

Peak	RetTime [min]	Type	Width[min]	Area[mAU*s]	Height[mAU]	Area%
	12.058	BB	1.6372	184.4750	6.7072	3.7244
	13.710	BB	3.1450	4768.6051	172.7817	96.2756

(*R*)-2'-(methylthio)-[1,1'-binaphthalen]-2-ol (1a):

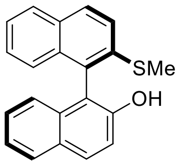

White semi-solid (30.3 mg, 48%, -10 ^{o}C for 13 h); R_f = 0.37 (petroleum ether/ethyl acetate = 5:1); the enantiomeric excess was determined to be 91% by HPLC analysis on Daicel Chirapak IA-H column (hexane/isopropanol = 70/30, flow rate 1 mL/min, T = 30 °C), UV 254 nm, t_R(major) 6.909 min, t_R(minor) 5.221 min; $[\alpha]_D^{25}$ = -5.70 (*c* = 0.480, $CHCl_3$); **^{1}H NMR (600 MHz, $CDCl_3$)** δ 8.01 (d, J = 8.8 Hz, 1H), 7.94 (d, J = 8.9 Hz, 1H), 7.89 (dd, J = 18.4, 8.1 Hz, 2H), 7.58 (d, J = 8.8 Hz, 1H), 7.43 (t, J = 7.4 Hz, 1H), 7.37 – 7.27 (m, 3H), 7.23 (d, J = 7.1 Hz, 1H), 7.15 (d, J = 8.5 Hz, 1H), 6.97 (d, J = 8.4 Hz, 1H), 4.84 (s, 1H), 2.42 (s, 3H); **^{13}C NMR (151 MHz, $CDCl_3$)** δ 150.99, 138.87, 133.37, 133.20, 131.48, 130.52, 129.84, 129.25, 128.29, 128.26, 127.57, 126.94, 126.86, 125.58, 124.83, 124.32, 123.59, 122.78, 117.61, 116.51, 15.41; **HRMS(ESI)**: Calcd. for $C_{21}H_{15}OS[M-H]^-$ 315.0849; found 315.0848.

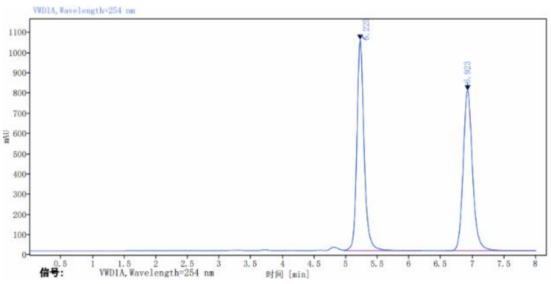

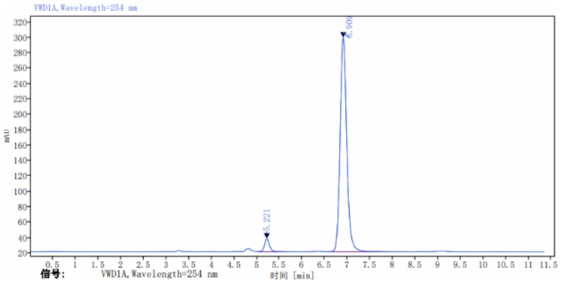

Peak	RetTime [min]	Type	Width[min]	Area[mAU*s]	Height[mAU]	Area%
	5.225	VB	1.2225	8337.0123	1042.9055	50.4335
	6.923	BBA	1.4365	8193.6818	792.2977	49.5665

Peak	RetTime [min]	Type	Width[min]	Area[mAU*s]	Height[mAU]	Area%
	5.221	VB	0.6843	135.7629	17.0517	4.5547
	6.909	BB	1.8400	2844.9503	278.2742	95.4453

(*S*)-8-methyl-2'-((*R*)-methylsulfinyl)-[1,1'-binaphthalen]-2-ol (3b):

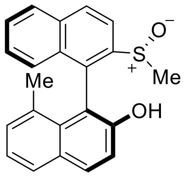

White solid (33.2 mg, 48%, -10 ^{o}C for 18 h); m.p. = 241-243 ^{o}C; R_f = 0.21 (petroleum ether/ethyl acetate = 1:2); the enantiomeric excess was determined to be 90% by HPLC analysis on Daicel Chirapak IA-H column (hexane/isopropanol = 90/10, flow rate 1 mL/min, T = 30 °C), UV 254 nm, t_R(major) 13.720 min, t_R(minor) 10.521 min; $[\alpha]_D^{25}$ = +160.56 (*c* = 0.60, $CHCl_3$); **^{1}H NMR (600 MHz, DMSO-*d_6*)** δ 9.87 (s,1H), 8.27 (d, J = 8.6 Hz, 1H), 8.07 (dd, J = 16.8, 8.4 Hz, 2H), 7.98 (d, J = 8.8 Hz, 1H), 7.80 (d, J = 8.0 Hz, 1H), 7.65 – 7.59 (m, 1H), 7.45 (d, J = 7.5 Hz, 1H), 7.37 (d, J = 8.8 Hz, 1H), 7.33 (s, 1H), 7.23 – 7.15 (m, 1H), 7.08 (d, J = 6.6 Hz, 1H), 2.32 (s, 3H), 1.38 (s, 3H); **^{13}C NMR (151

MHz, DMSO-d_6) δ 158.25, 147.45, 141.32, 138.85, 138.54, 138.33, 137.04, 136.73, 135.91, 134.58, 134.43, 133.63, 133.52, 132.61, 131.03, 127.93, 124.38, 124.27, 122.80, 118.79, 45.19, 27.50; **HRMS(ESI)**: Calcd. for $C_{22}H_{17}O_2S[M-H]^-$ 345.0955; found 345.0952.

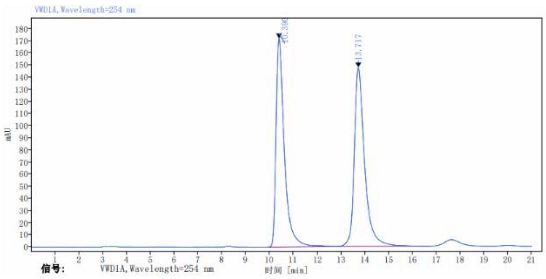

Peak	RetTime [min]	Type	Width[min]	Area[mAU^s]	Height[mAU]	Area%
	10.390	BB	2.9433	4189.0639	171.8118	48.5317
	13.717	BB	3.8950	4442.5436	147.2979	51.4683

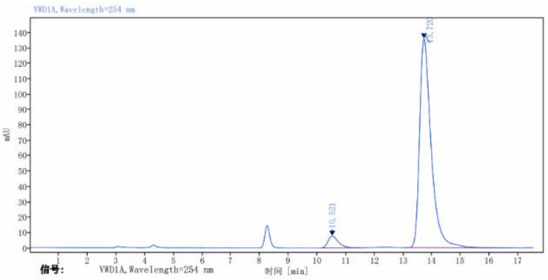

Peak	RetTime [min]	Type	Width[min]	Area[mAU^s]	Height[mAU]	Area%
	10.521	BB	1.5150	199.0922	7.8194	4.9586
	13.720	BB	3.9100	3815.9632	135.7978	95.0414

(*R*)-8-methyl-2'-(methylthio)-[1,1'-binaphthalen]-2-ol (1b):

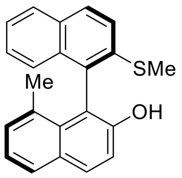

White semi-solid (31.1 mg, 47%, -10 °C for 18 h); R_f = 0.40 (petroleum ether/ethyl acetate = 5:1); the enantiomeric excess was determined to be 91% by HPLC analysis on Daicel Chirapak IA-H column (hexane/isopropanol = 70/30, flow rate 1 mL/min, T = 30 °C), UV 254 nm, t_R(major) 7.266 min, t_R(minor) 5.048 min; $[\alpha]_D^{25}$ = +21.18 (*c* = 0.76, $CHCl_3$); **^{1}H NMR (600 MHz, CDCl$_3$)** δ 8.03 (d, J = 8.7 Hz, 1H), 7.98 (d, J = 8.8 Hz, 1H), 7.92 (d, J = 8.1 Hz, 1H), 7.79 (d, J = 8.0 Hz, 1H), 7.55 (d, J = 8.7 Hz, 1H), 7.50 – 7.43 (m, 1H), 7.39 – 7.34 (m, 2H), 7.29 – 7.23 (m, 2H), 7.14 (d, J = 6.7 Hz, 1H), 4.87 (s, 1H), 2.50 (s, 3H), 1.72 (s, 3H); **^{13}C NMR (151 MHz, CDCl$_3$)** δ 151.41, 139.14, 134.07, 133.70, 132.45, 131.87, 131.00, 130.53, 130.13, 129.96, 129.82, 128.25, 127.93, 127.83, 125.59, 124.82, 123.38, 121.86, 117.05, 116.13, 22.27, 15.03; **HRMS(ESI)**: Calcd. for $C_{22}H_{17}OS[M-H]^-$ 329.1006; found 329.1004.

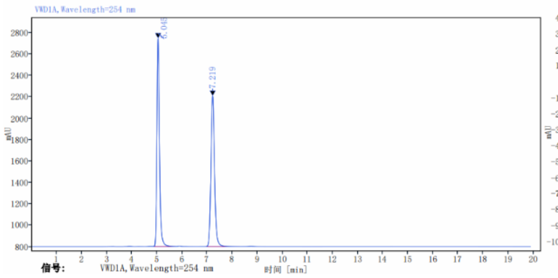

Peak	RetTime [min]	Type	Width[min]	Area[mAU^s]	Height[mAU]	Area%
	5.045	VV	0.8969	14108.7962	1944.5100	49.8924
	7.219	VB	1.4179	14169.6574	1407.5851	50.1076

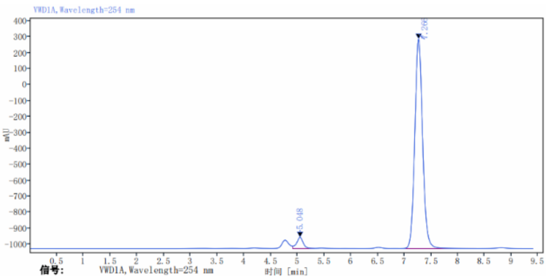

Peak	RetTime [min]	Type	Width[min]	Area[mAU^s]	Height[mAU]	Area%
	5.048	VV	0.4116	599.2410	70.0036	4.2797
	7.266	VB	1.4202	13402.8431	1313.3604	95.7203

(*S*)-8-bromo-2'-((*R*)-methylsulfinyl)-[1,1'-binaphthalen]-2-ol (3c):

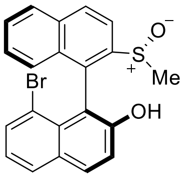

White solid (33.2 mg, 42%, -10 °C for 21 h); m.p. = 269-271 °C; R_f = 0.23 (petroleum ether/ethyl acetate = 1:2); the enantiomeric excess was determined to be 88% by HPLC analysis on Daicel Chirapak IA-H column (hexane/isopropanol = 90/10, flow rate 1 mL/min, T = 30 °C), UV 254 nm, t_R(major) 13.611 min, t_R(minor) 10.535 min; $[\alpha]_D^{25}$ = +84.82 (*c* = 0.56, $CHCl_3$); **^{1}H NMR (400 MHz, DMSO-*d*$_6$)** δ 10.35 (s, 1H), 8.24 (d, J = 8.7 Hz, 1H), 8.08 (d, J = 8.9 Hz, 1H), 8.06 – 7.91 (m, 3H), 7.63 (d, J = 7.4 Hz, 1H), 7.59 – 7.53 (m, 1H), 7.45 (d, J = 8.9 Hz, 1H), 7.38 (t, J = 7.6 Hz, 1H), 7.22 – 7.17 (m, 1H), 7.12 (d, J = 8.4 Hz, 1H), 2.47 (s, 3H); **^{13}C NMR (101 MHz, DMSO-*d*$_6$)** δ 159.58, 147.14, 139.70, 139.24, 139.18, 138.85, 137.22, 136.43, 135.47, 135.18, 134.82, 133.43, 132.38, 132.21, 130.91, 128.87, 124.52, 123.98, 121.19, 118.42, 45.40; **HRMS(ESI)**: Calcd. for $C_{21}H_{14}BrO_2S[M-H]^-$ 408.9903; found 408.9901.

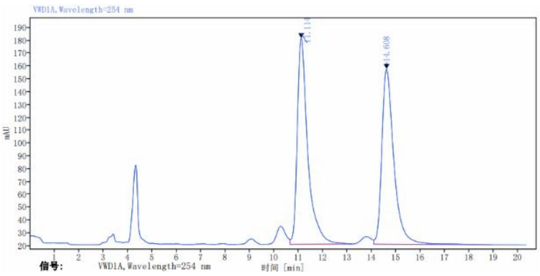

Peak	RetTime [min]	Type	Width[min]	Area[mAU^s]	Height[mAU]	Area%
	11.114	VB	2.5326	4797.8612	160.2532	50.6212
	14.608	VB	4.8360	4680.1086	136.2711	49.3788

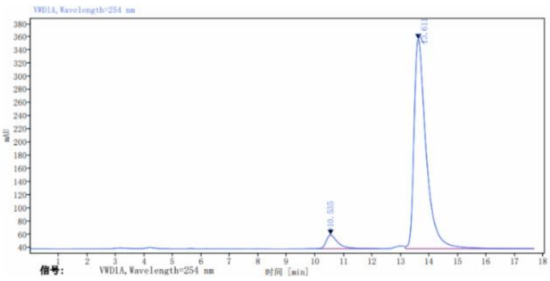

Peak	RetTime [min]	Type	Width[min]	Area[mAU^s]	Height[mAU]	Area%
	10.535	BB	2.2033	585.1747	20.7359	5.8676
	13.611	VBA	4.5162	9387.7663	318.2007	94.1324

(*R*)-8-bromo-2'-(methylthio)-[1,1'-binaphthalen]-2-ol (1c):

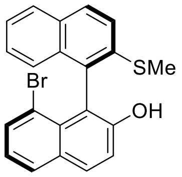

White semi-solid (41.8 mg, 53%, -10 °C for 21 h); R_f = 0.38 (petroleum ether/ ethyl acetate = 5:1); the enantiomeric excess was determined to be 84% by HPLC analysis on Daicel Chirapak IA-H column (hexane/isopropanol = 80/20, flow rate 0.8 mL/min, T = 30 °C), UV 254 nm, t_R(major) 7.167 min, t_R(minor) 6.617 min; $[\alpha]_D^{25}$ = +36.35 (*c* = 0.64, $CHCl_3$); **^{1}H NMR (400 MHz, $CDCl_3$)** δ 7.93 (d, J = 8.8 Hz, 1H), 7.87 (d, J = 8.9 Hz, 1H), 7.82 – 7.76 (m, 2H), 7.58 (dd, J = 7.5, 1.4 Hz, 1H), 7.44 (d, J = 8.8 Hz, 1H), 7.38 – 7.30 (m, 2H), 7.25 – 7.23 (m, 1H), 7.08 – 6.98 (m, 2H), 4.99 (s, 1H), 2.39 (s, 3H); **^{13}C NMR (101 MHz, $CDCl_3$)** δ 152.62, 139.59, 134.63, 134.22, 131.69, 131.40, 131.24, 130.01, 129.35, 128.24, 128.17, 127.53, 125.51, 124.73, 124.36, 123.92, 122.74, 118.23, 117.74, 116.04, 15.33; **HRMS(ESI)**: Calcd. for $C_{21}H_{14}BrOS[M-H]^-$ 392.9954; found 392.9952.

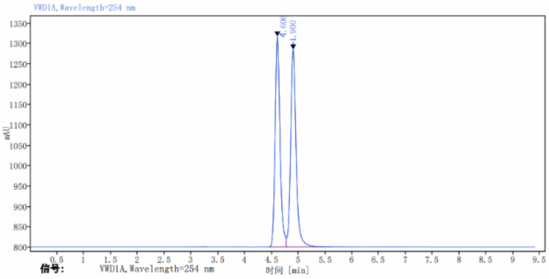

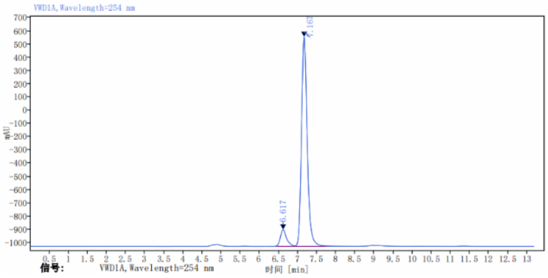

Peak	RetTime [min]	Type	Width[min]	Area[mAU^s]	Height[mAU]	Area%
	4.606	BV	0.6679	3317.3975	515.6058	49.1534
	4.900	VB	0.8426	3431.6731	483.6373	50.8466

Peak	RetTime [min]	Type	Width[min]	Area[mAU^s]	Height[mAU]	Area%
	6.617	BV	0.6058	1448.4674	126.2063	8.0198
	7.167	VB	1.5642	16612.5721	1581.5740	91.9802

(*S*)-7-methoxy-2'-((*R*)-methylsulfinyl)-[1,1'-binaphthalen]-2-ol (3d):

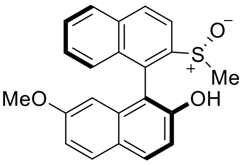

White solid (36.2 mg, 50%, -10 °C for 12 h); m.p. = 208-210 °C; R_f = 0.12 (petroleum ether/ethyl acetate = 5:4); the enantiomeric excess was determined to be 88% by HPLC analysis on Daicel Chirapak IA-H column (hexane/isopropanol = 90/10, flow rate 0.8 mL/min, T = 30 °C), UV 254 nm, t_R(major) 16.274 min, t_R(minor) 14.019 min; $[\alpha]_D^{25}$ = +141.67 (*c* = 0.30, $CHCl_3$); **^{1}H NMR (600 MHz, DMSO-*d*$_6$)** δ 9.96 (s, 1H), 8.32 (d, J = 8.7 Hz, 1H), 8.17 – 8.06 (m, 2H), 7.88 (dd, J = 18.7, 8.9 Hz, 2H), 7.65 – 7.59 (m, 1H), 7.41 (t, J = 7.7 Hz, 1H), 7.22 (d, J = 8.8 Hz, 1H), 7.14 (d, J = 8.5 Hz, 1H), 6.98 (d, J = 8.9, 1H), 5.90 (s, 1H), 3.37 (s, 3H), 2.24 (s, 3H); **^{13}C NMR (151 MHz, DMSO-*d*$_6$)** δ 158.56, 153.73, 143.81, 135.48, 134.74, 132.43, 132.04, 130.77, 130.75, 130.02, 129.00, 127.89, 127.69, 126.18, 123.64, 119.66, 116.32, 114.63, 112.80, 103.44, 55.20, 42.47; **HRMS(ESI)**: Calcd. for $C_{22}H_{17}O_3S[M-H]^-$ 361.0904; found 361.0903.

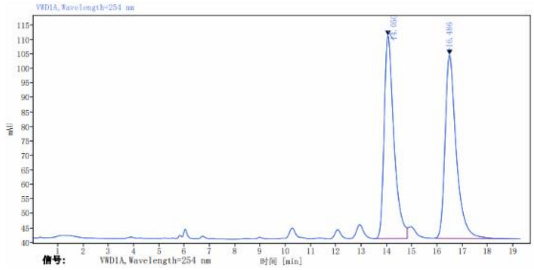

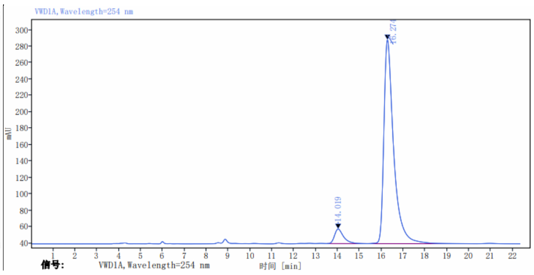

Peak	RetTime [min]	Type	Width[min]	Area[mAU^s]	Height[mAU]	Area%
	14.050	BV	1.3033	1879.1783	69.9228	49.0438
	16.486	BBA	3.3533	1952.4525	63.2640	50.9562

Peak	RetTime [min]	Type	Width[min]	Area[mAU^s]	Height[mAU]	Area%
	14.019	BB	1.8367	512.4741	17.7381	6.2161
	16.274	BB	5.0300	7731.8508	248.2723	93.7839

(*R*)-7-methoxy-2'-(methylthio)-[1,1'-binaphthalen]-2-ol (1d):

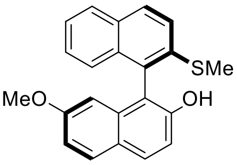

White semi-solid (31.1 mg, 45%, -10 °C for 12 h); R_f = 0.40 (petroleum ether/ethyl acetate = 5:1); the enantiomeric excess was determined to be 96% by HPLC analysis on Daicel Chirapak IA-H column

(hexane/isopropanol = 90/10, flow rate 1 mL/min, T = 30 °C), UV 254 nm, t_R(major) 16.601 min, t_R(minor) 10.093 min; $[\alpha]_D^{25}$ **=** -21.83 (*c* = 0.40, $CHCl_3$); **^{1}H NMR (400 MHz, $CDCl_3$)** δ 8.01 (d, J = 8.8 Hz, 1H), 7.88 (dd, J = 17.2, 8.5 Hz, 2H), 7.77 (d, J = 8.9 Hz, 1H), 7.59 (d, J = 8.8 Hz, 1H), 7.43 (d, J = 8.1, 1H), 7.37 – 7.28 (m, 2H), 7.22 – 7.17 (m, 2H), 7.00 (d, J = 8.9 Hz, 1H), 6.26 (d, J = 2.6 Hz, 1H), 3.51 (s, 3H), 2.44 (s, 3H); **^{13}C NMR (101 MHz, $CDCl_3$)** δ 158.57, 151.59, 138.79, 134.60, 133.21, 131.52, 130.22, 129.80, 128.22, 127.54, 126.70, 125.56, 124.84, 124.62, 124.36, 122.87, 115.72, 115.28, 115.05, 103.81, 55.05, 15.46; **HRMS(ESI)**: Calcd. for $C_{22}H_{17}O_2S[M-H]^-$ 345.0955; found 345.0951.

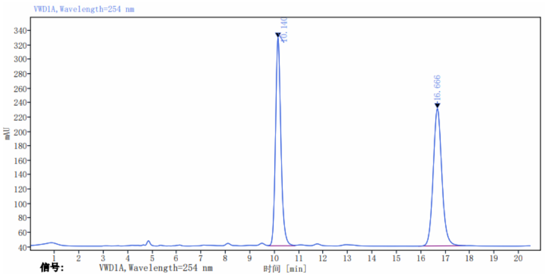

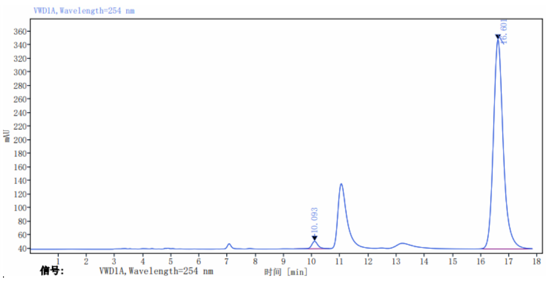

Peak	RetTime [min]	Type	Width[min]	Area[mAU*s]	Height[mAU]	Area%
	10.140	VV	1.1020	4455.2013	288.3920	48.5935
	16.666	BB	1.9406	4713.1145	190.5780	51.4065

Peak	RetTime [min]	Type	Width[min]	Area[mAU*s]	Height[mAU]	Area%
	10.093	BV	1.2594	171.7408	11.0035	2.2257
	16.601	BBA	2.0300	7544.5348	308.0548	97.7743

(*S*)-7-bromo-2'-((*R*)-methylsulfinyl)-[1,1'-binaphthalen]-2-ol (3e):

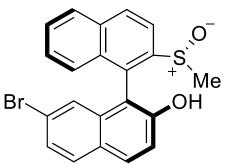

White solid (37.0 mg, 45%, -10 °C for 12.5 h); m.p. = 240-242 °C; R_f = 0.25 (petroleum ether/ethyl acetate = 1:2); the enantiomeric excess was determined to be 92% by HPLC analysis on Daicel Chirapak IA-H column (hexane/isopropanol = 90/10, flow rate 0.8 mL/min, T = 30 °C), UV 254 nm, t_R(major) 12.772 min, t_R(minor) 11.193 min; $[\alpha]_D^{25}$ **=** +154.59 (*c* = 0.66, $CHCl_3$); **^{1}H NMR (400 MHz, $CDCl_3$)** δ 9.28 (s, 1H), 8.04 (d, J = 8.7 Hz, 1H), 7.98 (d, J = 8.8 Hz, 1H), 7.86 – 7.77 (m, 2H), 7.69 (d, J = 8.7 Hz, 1H), 7.51 – 7.47 (m, 1H), 7.43 (d, J = 9.0 Hz, 1H), 7.36 – 7.29 (m, 2H), 7.22 (s, 1H), 6.89 (d, J = 2.0 Hz, 1H) , 2.35 (s, 3H); **^{13}C NMR (151 MHz, $CDCl_3$)** δ 153.75, 141.21, 135.39, 135.02, 132.13, 131.74, 130.71, 130.65, 130.27, 128.58, 127.86, 127.58, 126.74, 126.43, 126.28, 125.50, 121.55, 119.36, 118.91, 112.94, 41.92; **HRMS(ESI)**: Calcd. for $C_{21}H_{14}BrO_2S[M-H]^-$408.9903; found 408.9900.

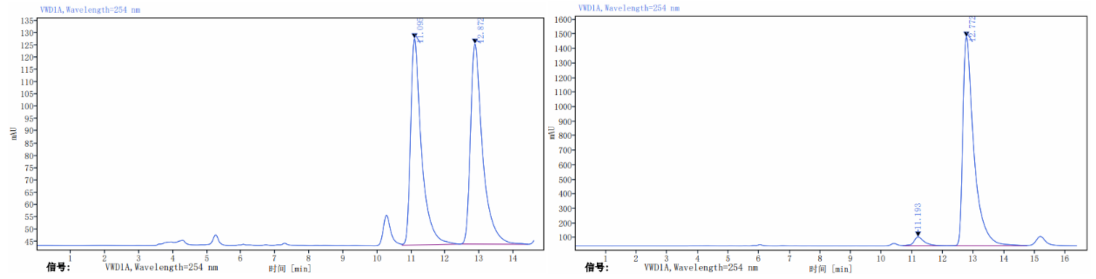

Peak	RetTime [min]	Type	Width[min]	Area[mAU*s]	Height[mAU]	Area%
	11.095	VB	1.6911	1880.9816	84.2790	48.1587
	12.872	BB	2.0067	2024.8158	81.7131	51.8413

Peak	RetTime [min]	Type	Width[min]	Area[mAU*s]	Height[mAU]	Area%
	11.193	VB	1.4463	1487.8092	61.2590	3.9569
	12.772	BV	2.6054	36112.5417	1435.2161	96.0431

(*R*)-7-bromo-2'-(methylthio)-[1,1'-binaphthalen]-2-ol (1e):

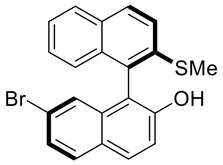

White solid (34.3 mg, 44%, -10 °C for 12.5 h); m.p. = 186-189 °C; R_f = 0.38 (petroleum ether/ethyl acetate = 5:1); the enantiomeric excess was determined to be 87% by HPLC analysis on Daicel Chirapak IA-H column (hexane/isopropanol = 90/10, flow rate 1.0 mL/min, T = 30 °C), UV 254 nm, t_R(major) 11.515 min, t_R(minor) 7.991 min; $[\alpha]_D^{25}$ = -47.67 (*c* = 0.70, $CHCl_3$); **^{1}H NMR (400 MHz, $CDCl_3$)** δ 8.03 (d, J = 8.8 Hz, 1H), 7.95 – 7.84 (m, 2H), 7.73 (d, J = 8.7 Hz, 1H), 7.58 (d, J = 8.8 Hz, 1H), 7.45 (d, J = 8.1 Hz, 1H), 7.40 (d, J = 8.7 Hz, 1H), 7.35 (d, J = 8.9 Hz, 1H), 7.34 – 7.28 (m, 1H), 7.14 – 7.08 (m, 2H), 4.91 (s, 1H), 2.44 (s, 3H); **^{13}C NMR (101 MHz, $CDCl_3$)** δ 151.89, 139.07, 134.51, 133.18, 131.51, 130.44, 130.17, 129.92, 128.38, 127.78, 127.69, 127.06, 126.37, 125.90, 125.69, 124.49, 122.81, 121.52, 118.09, 115.95, 15.38; **HRMS(ESI)**: Calcd. for $C_{21}H_{14}BrOS[M-H]^-$ 392.9954; found 392.9955.

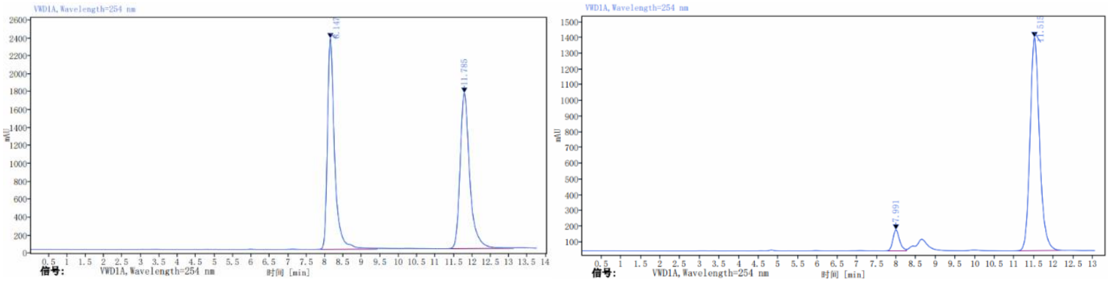

Peak	RetTime [min]	Type	Width[min]	Area[mAU*s]	Height[mAU]	Area%
	8.147	BV	1.7377	32077.8036	2350.8970	50.3324
	11.785	BV	1.8233	31654.0767	1734.1677	49.6676

Peak	RetTime [min]	Type	Width[min]	Area[mAU*s]	Height[mAU]	Area%
	7.991	BV	0.5978	1610.5463	134.2785	6.5879
	11.515	BB	1.3157	22836.5224	1357.2551	93.4121

(*S*)-6-bromo-2'-((*R*)-methylsulfinyl)-[1,1'-binaphthalen]-2-ol (3f):

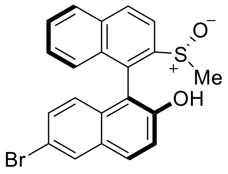

White solid (41.0 mg, 50%, -10 °C for 14 h); m.p. = 185-187 °C; R_f = 0.21 (petroleum ether/ethyl acetate = 1:2); the enantiomeric excess was determined to be 92% by HPLC analysis on Daicel Chirapak IA-H column

(hexane/isopropanol = 90/10, flow rate 1 mL/min, T = 30 °C), UV 254 nm, t_R(major) 10.820 min, t_R(minor) 9.577 min; $[\alpha]_D^{25}$ = +151.38 (*c* = 0.51, CH_3Cl); **^{1}H NMR (400 MHz, $CDCl_3$)** δ 8.96 (s, 1H), 8.11 (s, 2H), 7.99 (d, J = 2.0 Hz, 1H), 7.93 (d, J = 8.2 Hz, 1H), 7.78 (d, J = 8.9 Hz, 1H), 7.58 – 7.51 (m, 1H), 7.44 (d, J = 9.0 Hz, 1H), 7.33 (d, J = 8.2 Hz, 1H), 7.23 (dd, J = 5.5, 3.2 Hz, 2H), 6.63 (d, J = 8.9 Hz, 1H), 2.31 (s, 3H); **^{13}C NMR (101 MHz, $CDCl_3$)** δ 152.97, 141.59, 134.99, 132.45, 132.27, 131.61, 130.56, 130.52, 130.22, 129.90, 129.54, 128.54, 127.90, 127.58, 126.37, 125.47, 120.04, 118.98, 116.72, 113.70, 41.92; **HRMS(ESI)**: Calcd. for $C_{21}H_{14}BrO_2S[M-H]^-$ 408.9903; found 408.9899.

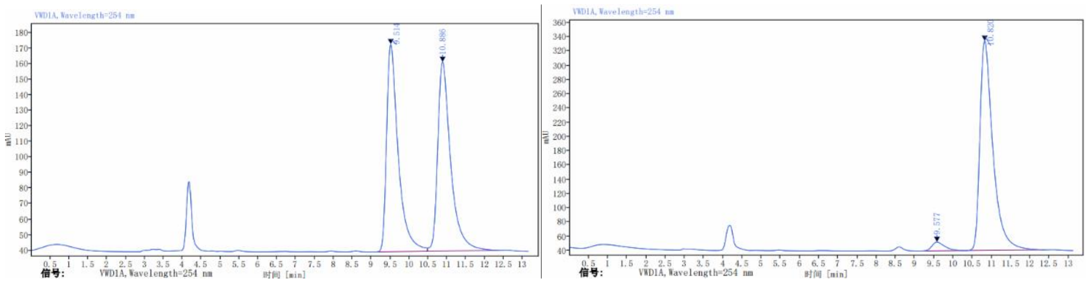

Peak	RetTime [min]	Type	Width[min]	Area[mAU^s]	Height[mAU]	Area%
	9.514	BV	1.3751	2854.5887	133.4140	49.5640
	10.886	VB	1.9057	2904.8071	121.5557	50.4360

Peak	RetTime [min]	Type	Width[min]	Area[mAU^s]	Height[mAU]	Area%
	9.577	BB	1.1417	282.5015	12.2292	3.9045
	10.820	BB	2.0977	6952.8475	294.1660	96.0955

(*R*)-6-bromo-2'-(methylthio)-[1,1'-binaphthalen]-2-ol (1f):

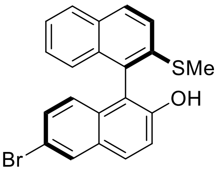

White solid (37.5 mg, 48%, -10 °C for 14 h); m.p. = 144-146 °C; R_f = 0.38 (petroleum ether/ethyl acetate = 5:1); the enantiomeric excess was determined to be 95% by HPLC analysis on Daicel Chirapak IA-H column (hexane/isopropanol = 70/30, flow rate 1 mL/min, T = 30 °C), UV 254 nm, t_R(major) 6.167 min, t_R(minor) 5.005 min; $[\alpha]_D^{25}$ = -28.54 (*c* = 0.8, $CHCl_3$); **^{1}H NMR (400 MHz, $CDCl_3$)** δ 8.01 (d, J = 8.8 Hz, 1H), 7.90 (d, J = 8.2 Hz, 2H), 7.83 (d, J = 8.9 Hz, 1H), 7.56 (d, J = 8.8 Hz, 1H), 7.47 (d, J = 7.9 Hz, 1H), 7.39 – 7.29 (m, 3H), 7.29 – 7.23 (m, 1H), 7.10 (d, J = 8.5 Hz, 1H), 6.83 (d, J = 8.9 Hz, 1H), 2.42 (s, 3H); **^{13}C NMR (101 MHz, $CDCl_3$)** δ 151.40, 138.93, 133.25, 131.77, 131.46, 130.39, 130.24, 130.10, 130.06, 129.56, 128.35, 128.24, 127.74, 126.21, 125.69, 124.56, 124.37, 122.75, 118.85, 117.40, 15.38; **HRMS(ESI)**: Calcd. for $C_{21}H_{14}BrOS[M-H]^-$ 392.9954; found 392.9951.

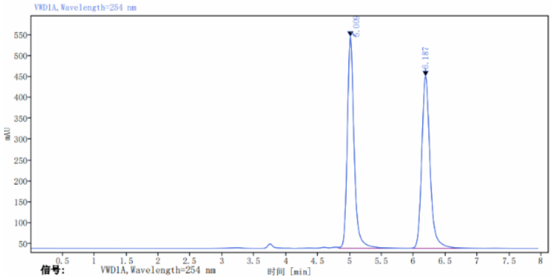

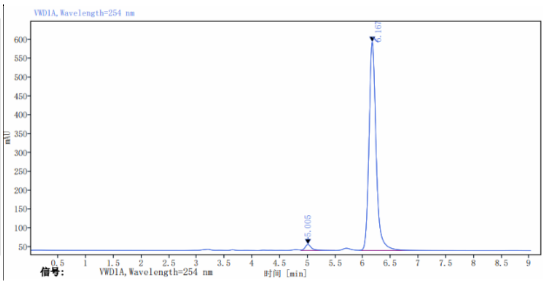

Peak	RetTime [min]	Type	Width[min]	Area[mAU^s]	Height[mAU]	Area%
	5.009	VB	1.0853	3755.0190	505.2267	50.1696
	6.187	BB	1.1617	3729.6351	410.7251	49.8304

Peak	RetTime [min]	Type	Width[min]	Area[mAU^s]	Height[mAU]	Area%
	5.005	VB	0.6175	129.7071	15.9016	2.5546
	6.167	VB	1.7018	4947.7181	554.0265	97.4454

(*S*)-2-hydroxy-2'-((*R*)-methylsulfinyl)-[1,1'-binaphthalene]-6-carbonitrile (3g):

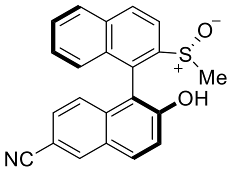

White solid (36.2 mg, 50%, -10 °C for 17 h); m.p. = 180-182 °C; R_f = 0.20 (petroleum ether/ethyl acetate = 1:2); the enantiomeric excess was determined to be 95% by HPLC analysis on Daicel Chirapak IA-H column (hexane/isopropanol = 90/10, flow rate 1 mL/min, T = 30 °C), UV 254 nm, t_R(major) 19.045 min, t_R(minor) 15.995 min; $[\alpha]_D^{25}$ = +236.62 (*c* = 0.74, $CHCl_3$); **^{1}H NMR (400 MHz, $CDCl_3$)** δ 9.86 (s, 1H), 8.14 (d, J = 1.7 Hz, 1H), 8.02 (s, 2H), 7.88 – 7.81 (m, 2H), 7.47 (dd, J = 11.4, 8.3 Hz, 2H), 7.25 (dd, J = 8.6, 6.9 Hz, 2H), 7.11 (d, J = 8.5 Hz, 1H), 6.75 (d, J = 8.7 Hz, 1H), 2.27 (s, 3H); **^{13}C NMR (101 MHz, $CDCl_3$)** δ 155.81, 141.13, 141.11, 135.70, 135.06, 134.65, 132.04, 131.39, 130.80, 128.63, 128.04, 127.73, 127.65, 127.13, 126.18, 124.65, 120.75, 119.33, 118.89, 114.08, 106.23, 41.86; **HRMS(ESI)**: Calcd. for $C_{22}H_{14}NO_2S[M-H]^-$ 356.0751; found 356.0749.

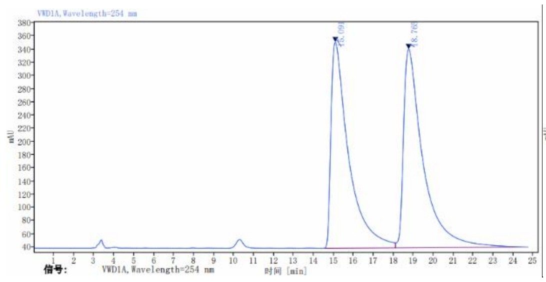

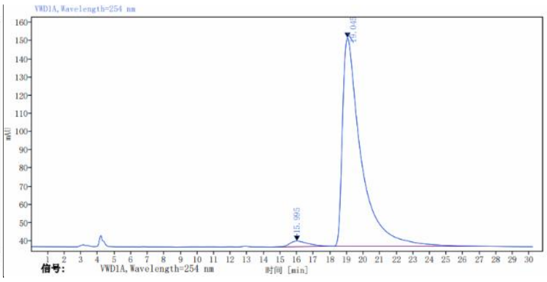

Peak	RetTime [min]	Type	Width[min]	Area[mAU^s]	Height[mAU]	Area%
	15.091	BV	3.6400	19230.6129	312.3499	48.4072
	18.765	VBA	6.6533	20496.1714	301.0121	51.5928

Peak	RetTime [min]	Type	Width[min]	Area[mAU^s]	Height[mAU]	Area%
	15.995	BB	3.6000	250.5639	3.0866	2.7181
	19.045	BB	9.3800	8967.9269	114.4576	97.2819

(*R*)-2-hydroxy-2'-(methylthio)-[1,1'-binaphthalene]-6-carbonitrile (1g):

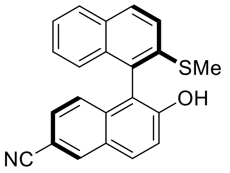

White solid (34.0 mg, 50%, -10 °C for 17 h); m.p. = 177-179 °C; R_f = 0.33 (petroleum ether/ethyl acetate = 3:1); the enantiomeric excess was determined to be 85% by HPLC analysis on Daicel Chirapak IA-H column (hexane/isopropanol = 70/30, flow rate 1 mL/min, T = 30 °C), UV 254 nm, t_R(major) 6.535 min,

t_R(minor) 5.075 min; $[\alpha]_D^{25}$ = -32.66 (*c* = 0.70, $CHCl_3$); **^{1}H NMR (400 MHz, $CDCl_3$)** δ 8.24 (d, J = 1.7 Hz, 1H), 8.04 (d, J = 8.8 Hz, 1H), 7.98 (d, J = 9.0 Hz, 1H), 7.92 (d, J = 8.2 Hz, 1H), 7.58 (d, J = 8.8 Hz, 1H), 7.50 – 7.41 (m, 2H), 7.37 – 7.29 (m, 2H), 7.04 (dd, J = 10.7, 8.5 Hz, 2H), 5.25 (s, 1H), 2.43 (s, 3H); **^{13}C NMR (101 MHz, $CDCl_3$)** δ 149.12, 134.34, 130.31, 129.63, 128.37, 126.74, 126.29, 125.61, 123.74, 123.38, 123.16, 122.75, 121.06, 120.82, 120.67, 119.48, 118.01, 114.92, 114.76, 112.39, 102.12, 10.61; **HRMS(ESI)**: Calcd. for $C_{22}H_{14}NOS[M-H]^-$ 340.0802; found 340.0800.

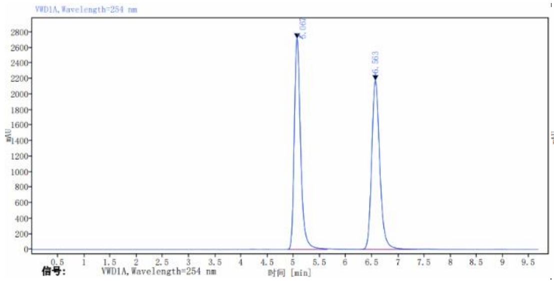

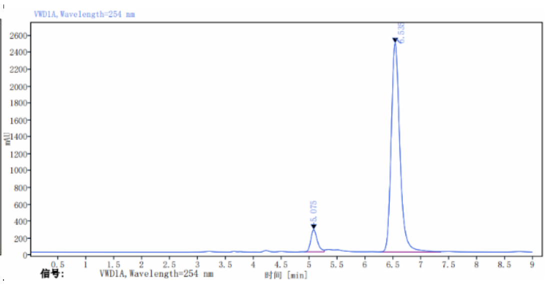

Peak	RetTime [min]	Type	Width[min]	Area[mAU*s]	Height[mAU]	Area%
	5.067	BV	0.8801	23037.1828	2715.5828	49.4752
	6.563	BV	1.0899	23525.8954	2174.4768	50.5248

Peak	RetTime [min]	Type	Width[min]	Area[mAU*s]	Height[mAU]	Area%
	5.075	BV	0.3566	2127.5256	260.8469	7.3441
	6.535	BV	1.0985	26841.7146	2469.3451	92.6559

(*S*)-2-hydroxy-2'-((*R*)-methylsulfinyl)-[1,1'-binaphthalene]-6-carbaldehyde (3h):

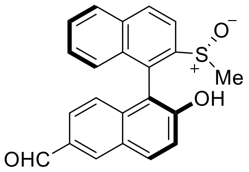

White solid (33.1 mg, 46%, -10 °C for 18 h); m.p. = 179-181 °C; R_f = 0.18 (petroleum ether/ethyl acetate = 1:2); the enantiomeric excess was determined to be 92% by HPLC analysis on Daicel Chirapak IA-H column (hexane/isopropanol = 90/10, flow rate 1 mL/min, T = 30 °C), UV 254 nm, t_R(major) 20.513 min, t_R(minor) 16.948 min; $[\alpha]_D^{25}$ = +183.89 (*c* = 0.36, $CHCl_3$); **^{1}H NMR (400 MHz, $CDCl_3$)** δ 10.00 (s, 1H), 9.57 (s, 1H), 8.26 (d, J = 1.7 Hz, 1H), 8.05 (s, 2H), 7.97 (d, J = 8.9 Hz, 1H), 7.86 (d, J = 8.2 Hz, 1H), 7.59 (d, J = 8.7 Hz, 1H), 7.52 – 7.43 (m, 2H), 7.30 – 7.24 (m, 1H), 7.16 (d, J = 8.6 Hz, 1H), 6.78 (d, J = 8.8 Hz, 1H), 2.28 (s, 3H); **^{13}C NMR (101 MHz, $CDCl_3$)** δ 191.70, 155.92, 141.31, 137.44, 135.15, 135.06, 132.59, 132.15, 131.73, 131.63, 130.75, 128.61, 128.02, 127.69, 127.36, 126.25, 124.57, 124.25, 120.04, 118.92, 114.31, 41.90; **HRMS(ESI)**: Calcd. for $C_{22}H_{15}O_3S[M-H]^-$ 359.0747; found 359.0745.

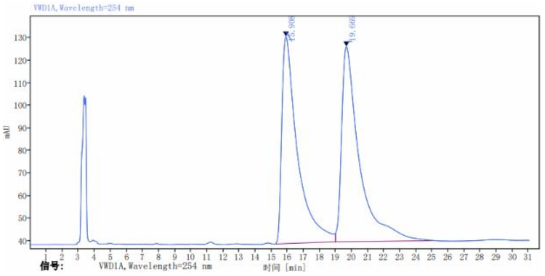

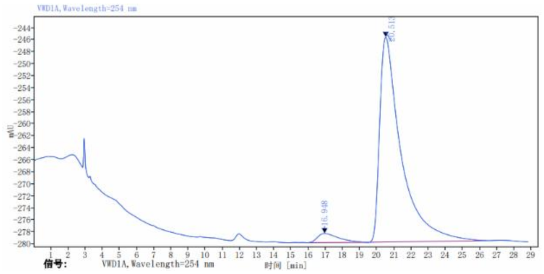

Peak	RetTime [min]	Type	Width[min]	Area[mAU*s]	Height[mAU]	Area%
	15.908	BM m	0.9624	6199.1908	91.7338	48.3148
	19.669	MB m	1.0867	6631.6462	86.4762	51.6852

Peak	RetTime [min]	Type	Width[min]	Area[mAU*s]	Height[mAU]	Area%
	16.948	MM m	1.0038	129.6791	1.5178	4.1936
	20.513	BB	6.9167	2962.6376	34.1276	95.8064

(*R*)-2-hydroxy-2'-(methylthio)-[1,1'-binaphthalene]-6-carbaldehyde (1h):

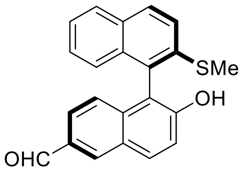

White solid (31.6 mg, 46%, -10 °C for 18 h); m.p. = 178-180 °C; R_f = 0.36 (petroleum ether/ethyl acetate = 3:1); the enantiomeric excess was determined to be 81% by HPLC analysis on Daicel Chirapak IA-H column (hexane/isopropanol = 70/30, flow rate 1 mL/min, T = 30 °C), UV 254 nm, t_R(major) 7.017 min, t_R(minor) 5.684 min; $[\alpha]_D^{25}$ **=** -72.13 (*c* = 0.36, $CHCl_3$); **^{1}H NMR (400 MHz, $CDCl_3$)** δ 9.98 (s, 1H), 8.28 (d, J = 1.8 Hz, 1H), 8.02 (d, J = 8.9 Hz, 1H), 7.95 (d, J = 8.8 Hz, 1H), 7.84 (d, J = 8.2 Hz, 1H), 7.62 (d, J = 8.7, Hz, 1H), 7.51 (d, J = 8.9 Hz, 1H), 7.42 – 7.33 (m, 2H), 7.25 – 7.20 (m, 1H), 7.00 (dd, J = 17.2, 8.6 Hz, 2H), 5.21 (s, 1H), 2.35 (s, 3H); **^{13}C NMR (101 MHz, $CDCl_3$)** δ 187.22, 149.33, 134.27, 132.11, 130.02, 128.42, 127.48, 126.75, 125.48, 123.69, 123.58, 123.48, 123.07, 121.17, 121.00, 120.66, 119.61, 119.38, 118.06, 114.23, 112.57, 10.65; **HRMS(ESI)**: Calcd. for $C_{22}H_{15}O_2S[M-H]^-$ 343.0798; found 343.0796.

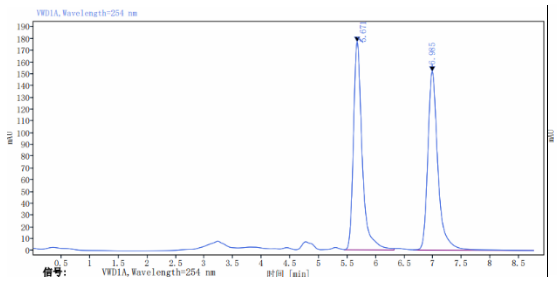

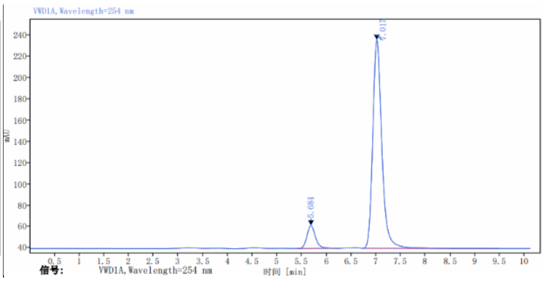

Peak	RetTime [min]	Type	Width[min]	Area[mAU*s]	Height[mAU]	Area%
	5.671	VV	0.8737	1884.4735	176.4100	50.1811
	6.985	VBA	2.1063	1870.8695	151.4753	49.8189

Peak	RetTime [min]	Type	Width[min]	Area[mAU*s]	Height[mAU]	Area%
	5.684	BB	0.8572	258.3045	21.5402	9.3001
	7.017	VB	2.7619	2519.1343	196.0426	90.6999

methyl (*S*)-2-hydroxy-2'-((*R*)-methylsulfinyl)-[1,1'-binaphthalene]-6-carboxylate (3i):

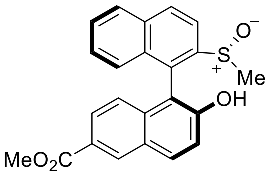

White solid (35.1 mg, 45%, -10 °C for 16.5 h); m.p. = 181-183 °C; R_f = 0.18 (petroleum ether/ethyl acetate = 1:2); the enantiomeric excess was determined to be 88% by HPLC analysis on Daicel Chirapak IA-H

column (hexane/isopropanol = 90/10, flow rate 1 mL/min, T = 30 °C), UV 254 nm, t_R(major) 14.543 min, t_R(minor) 12.583 min; $[\alpha]_D^{25}$ = +274.35 (c = 0.66, $CHCl_3$); **^{1}H NMR (400 MHz, DMSO-d_6)** δ 10.52 (s, 1H), 8.66 (d, J = 1.8 Hz, 1H), 8.35 (d, J = 8.7 Hz, 1H), 8.23 (d, J = 8.9 Hz, 1H), 8.20 – 8.11 (m, 2H), 7.76 – 7.71 (m, 1H), 7.67 – 7.61 (m, 1H), 7.50 (d, J = 8.9 Hz, 1H), 7.44 – 7.37 (m, 1H), 7.09 (d, J = 8.5 Hz, 1H), 6.75 (d, J = 8.9 Hz, 1H), 3.87 (s, 3H), 2.23 (s, 3H); **^{13}C NMR (151 MHz, DMSO-d_6)** δ 167.19, 155.05, 141.52, 136.57, 135.12, 132.34, 132.31, 132.25, 131.39, 130.18, 128.37, 127.64, 127.38, 127.19, 126.07, 125.79, 124.54, 123.65, 118.89, 118.44, 113.76, 51.23, 40.83; **HRMS(ESI)**: Calcd. for $C_{23}H_{17}O_4S[M-H]^-$ 389.0853; found 389.0851.

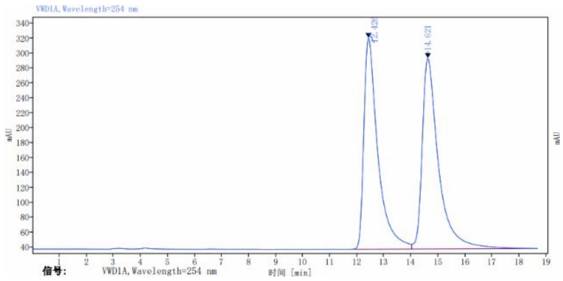

Peak	RetTime [min]	Type	Width[min]	Area[mAU^s]	Height[mAU]	Area%
	12.426	BV	2.2018	10189.4634	282.5851	48.6915
	14.621	VBA	4.6616	10737.1226	255.3372	51.3085

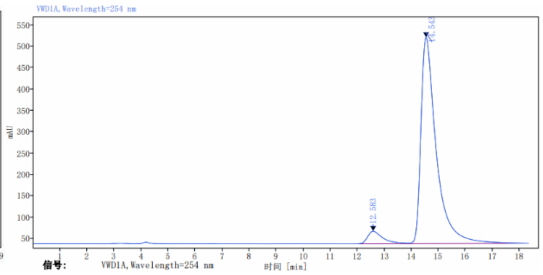

Peak	RetTime [min]	Type	Width[min]	Area[mAU^s]	Height[mAU]	Area%
	12.583	BV	2.0065	1176.0341	29.4755	5.8035
	14.543	VBA	4.4435	19088.3589	482.5907	94.1965

methyl (*R*)-2-hydroxy-2'-(methylthio)-[1,1'-binaphthalene]-6-carboxylate (1i):

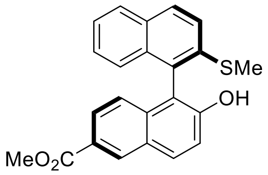

White semi-solid (36.6 mg, 49%, -10 °C for 16.5 h); R_f = 0.36 (petroleum ether/ethyl acetate = 3:1); the enantiomeric excess was determined to be 82% by HPLC analysis on Daicel Chirapak IA-H column (hexane/isopropanol = 70/30, flow rate 1 mL/min, T = 30 °C), UV 254 nm, t_R(major) 8.997 min, t_R(minor) 5.695 min; $[\alpha]_D^{25}$ = -50.83 (c = 0.68, $CHCl_3$); **^{1}H NMR (400 MHz, $CDCl_3$)** δ 8.66 (d, J = 1.8 Hz, 1H), 8.07 (dd, J = 8.9, 4.7 Hz, 2H), 7.94 (d, J = 8.2 Hz, 1H), 7.84 (d, J = 8.8 Hz, 1H), 7.62 (d, J = 8.8 Hz, 1H), 7.50 – 7.41 (m, 2H), 7.33 (d, J = 8.3 Hz, 1H), 7.13 (d, J = 8.5 Hz, 1H), 7.02 (d, J = 8.8 Hz, 1H), 5.08 (s, 1H), 3.96 (s, 3H), 2.46 (s, 3H); **^{13}C NMR (101 MHz, $CDCl_3$)** δ 162.58, 148.43, 134.24, 130.99, 128.48, 127.25, 126.74, 125.37, 123.61, 123.51, 122.98, 121.61, 121.44, 120.94, 120.50, 119.77, 119.76, 118.08, 113.81, 112.03, 47.32, 10.65; **HRMS(ESI)**: Calcd. for $C_{23}H_{17}O_3S[M-H]^-$ 373.0904; found 373.0900.

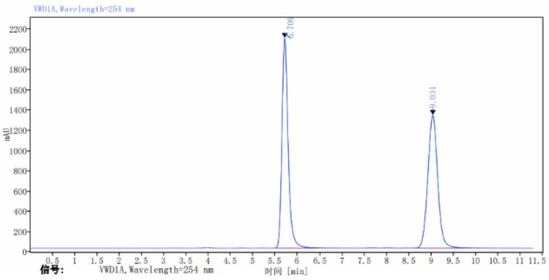

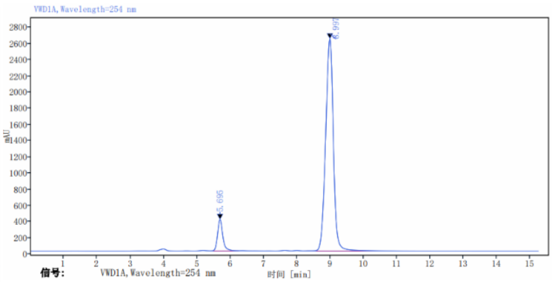

Peak	RetTime [min]	Type	Width[min]	Area[mAU*s]	Height[mAU]	Area%
	5.709	VB	1.8538	20228.8822	2075.6226	49.8493
	9.031	VBA	3.0238	20351.2248	1313.5636	50.1507

Peak	RetTime [min]	Type	Width[min]	Area[mAU*s]	Height[mAU]	Area%
	5.695	VV	0.8320	4261.8794	388.2684	9.1295
	8.997	VB	4.1525	42420.5996	2621.6932	90.8705

(*S*)-2'-((*R*)-methylsulfinyl)-6-phenyl-[1,1'-binaphthalen]-2-ol (3j):

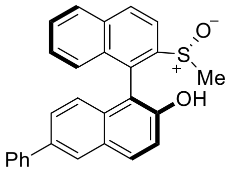

White solid (39.2 mg, 48%, -10 °C for 13.5 h); m.p. = 148-150 °C; R_f = 0.23 (petroleum ether/ethyl acetate = 1:2); the enantiomeric excess was determined to be 91% by HPLC analysis on Daicel Chirapak IA-H column (hexane/isopropanol = 90/10, flow rate 1 mL/min, T = 30 °C), UV 254 nm, t_R(major) 14.507 min, t_R(minor) 12.723 min; $[\alpha]_D^{25}$ = +271.94 (*c* = 0.60, $CHCl_3$); **^{1}H NMR (400 MHz, DMSO-*d*$_6$)** δ 10.10 (s, 1H), 8.37 – 8.32 (m, 1H), 8.24 (d, J = 2.0 Hz, 1H), 8.19 – 8.05 (m, 3H), 7.77 – 7.68 (m, 2H), 7.62 (d, J = 8.2 Hz, 1H), 7.56 (d, J = 8.8 Hz, 1H), 7.48 – 7.31 (m, 5H), 7.16 (d, J = 8.2 Hz, 1H), 6.72 (d, J = 8.8 Hz, 1H), 2.24 (s, 3H); **^{13}C NMR (101 MHz, DMSO-*d*$_6$)** δ 158.01, 148.63, 145.01, 139.69, 139.54, 138.16, 137.05, 137.02, 136.24, 134.87, 134.17, 133.76, 133.34, 132.70, 132.57, 132.45, 131.86, 131.29, 131.04, 129.33, 124.44, 124.13, 118.38, 47.27; **HRMS(ESI)**: Calcd. for $C_{27}H_{19}O_2S[M-H]^-$ 407.1111; found 407.1111.

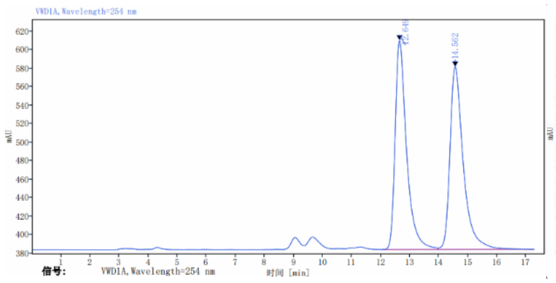

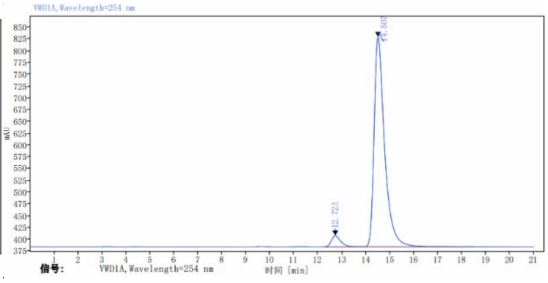

Peak	RetTime [min]	Type	Width[min]	Area[mAU*s]	Height[mAU]	Area%
	12.649	BV	1.9242	6159.9287	225.6828	49.4680
	14.562	VBA	3.3125	6292.4207	197.1659	50.5320

Peak	RetTime [min]	Type	Width[min]	Area[mAU*s]	Height[mAU]	Area%
	12.723	BB	1.7350	672.1753	23.7308	4.7173
	14.507	BB	4.6900	13576.9231	445.1193	95.2827

(*R*)-2'-(methylthio)-6-phenyl-[1,1'-binaphthalen]-2-ol (1j):

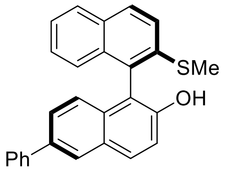

White solid solid (39.0 mg, 50%, -10 °C for 13.5 h); m.p. = 129-131 °C; R_f = 0.36 (petroleum ether/ethyl acetate = 5:1); the enantiomeric excess was determined to be 91% by HPLC analysis on Daicel Chirapak IA-H column

(hexane/isopropanol = 70/30, flow rate 1 mL/min, T = 30 °C), UV 254 nm, t_R(major) 7.508 min, t_R(minor) 6.068 min; $[\alpha]_D^{25}$ = -59.87 (*c* = 0.74, $CHCl_3$); **^{1}H NMR (400 MHz, $CDCl_3$)** δ 7.99 (d, J = 1.9 Hz, 1H), 7.97 – 7.86 (m, 2H), 7.82 (d, J = 8.2 Hz, 1H), 7.57 (d, J = 7.7 Hz, 2H), 7.50 (d, J = 8.8 Hz, 1H), 7.42 (d, J = 8.7 Hz, 1H), 7.43 – 7.27 (m, 3H), 7.30 (d, J = 8.9 Hz, 1H), 7.28 – 7.19 (m, 2H), 7.11 (d, J = 8.5 Hz, 1H), 6.96 (d, J = 8.8 Hz, 1H), 4.80 (s, 1H), 2.35 (s, 3H); **^{13}C NMR (101 MHz, $CDCl_3$)** δ 151.20, 141.15, 138.89, 136.39, 133.41, 132.45, 131.51, 130.85, 129.91, 129.53, 128.81, 128.31, 127.66, 127.26, 127.08, 126.91, 126.57, 126.30, 125.63, 124.91, 124.84, 122.80, 118.13, 116.50, 15.44; **HRMS(ESI)**: Calcd. for $C_{27}H_{19}OS[M-H]^-$ 391.1162; found 391.1160.

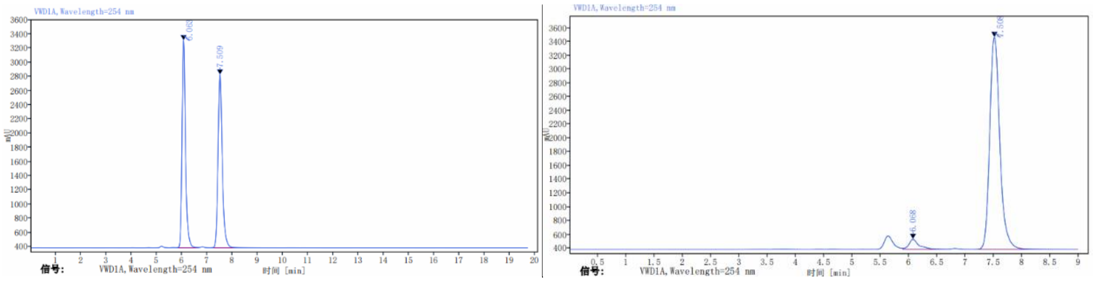

Peak	RetTime [min]	Type	Width[min]	Area[mAU^s]	Height[mAU]	Area%
	6.063	VV	0.8785	28374.0020	2923.2338	49.5067
	7.509	BB	1.9834	28939.4682	2439.3720	50.4933

Peak	RetTime [min]	Type	Width[min]	Area[mAU^s]	Height[mAU]	Area%
	6.068	VV	0.7545	1829.3008	145.8377	4.5512
	7.508	BBA	1.8539	38364.5261	3078.7989	95.4488

(*S*)-6-(4-(*tert*-butyl)phenyl)-2'-((*R*)-methylsulfinyl)-[1,1'-binaphthalen]-2-ol (3k):

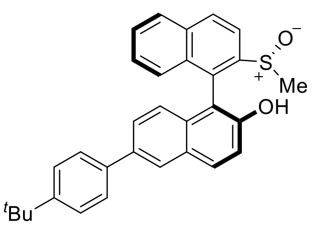

White solid (43.3 mg, 47%, -10 °C for 13.5 h); m.p. = 146-148 °C; R_f = 0.23 (petroleum ether/ethyl acetate = 1:2); the enantiomeric excess was determined to be 92% by HPLC analysis on Daicel Chirapak IA-H column (hexane/isopropanol = 90/10, flow rate 1 mL/min, T = 30 °C), UV 254 nm, t_R(major) 11.033 min, t_R(minor) 9.548 min; $[\alpha]_D^{25}$ = +89.85 (*c* = 0.90, $CHCl_3$); **^{1}H NMR (600 MHz, $CDCl_3$)** δ 8.62 (s, 1H), 8.09 – 8.03 (m, 2H), 7.95 (s, 1H), 7.85 (dd, J = 16.5, 8.6 Hz, 2H), 7.50 (d, J = 8.3 Hz, 2H), 7.49 – 7.43 (m, 1H), 7.37 (dd, J = 17.1, 8.6 Hz, 4H), 7.27 (s, 1H), 6.73 (d, J = 8.7 Hz, 1H), 2.24 (s, 3H), 1.28 (s, 9H); **^{13}C NMR (151 MHz, $CDCl_3$)** δ 152.59, 150.24, 137.83, 135.69, 135.01, 133.00, 132.51, 132.16, 131.11, 130.37, 128.78, 128.48, 127.80, 127.47, 126.73, 126.65, 126.61, 126.13, 125.82, 119.26, 119.08, 113.43, 42.04, 34.56, 31.38; **HRMS(ESI)**: Calcd. for $C_{31}H_{27}O_2S[M-H]^-$ 463.1737; found 463.1735.

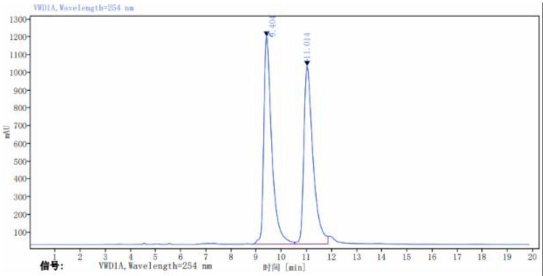

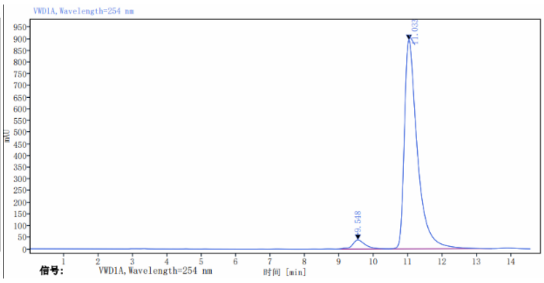

Peak	RetTime [min]	Type	Width[min]	Area[mAU*s]	Height[mAU]	Area%
	9.404	BV	1.6898	25843.4730	1167.1324	50.4226
	11.014	VV	1.3296	25410.3263	1000.1664	49.5774

Peak	RetTime [min]	Type	Width[min]	Area[mAU*s]	Height[mAU]	Area%
	9.548	VV	1.5090	1010.3043	38.5042	4.1197
	11.033	VB	3.0156	23513.5539	893.5023	95.8803

(*R*)-6-(4-(*tert*-butyl)phenyl)-2'-(methylthio)-[1,1'-binaphthalen]-2-ol (1k):

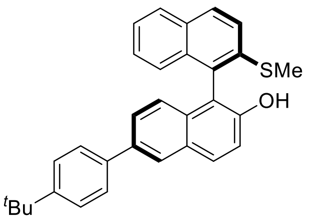

White semi-solid (42.1 mg, 47%, -10 ^{o}C for 13.5 h); R_f = 0.37 (petroleum ether/ethyl acetate = 5:1); the enantiomeric excess was determined to be 93% by HPLC analysis on Daicel Chirapak IA-H column (hexane/isopropanol = 70/30, flow rate 1 mL/min, T = 30 °C), UV 254 nm, t_R(major) 6.284 min, t_R(minor) 5.362 min; $[\alpha]_D^{25}$ = -0.24 (*c* = 0.880, $CHCl_3$); **^{1}H NMR (600 MHz, $CDCl_3$)** δ 8.10 (s, 1H), 8.07 (d, J = 8.8 Hz, 1H), 8.03 (d, J = 8.9 Hz, 1H), 7.95 (d, J = 8.2 Hz, 1H), 7.64 (d, J = 8.8 Hz, 3H), 7.57 – 7.46 (m, 4H), 7.42 (d, J = 8.9 Hz, 1H), 7.38 – 7.32 (m, 1H), 7.25 (d, J = 8.5 Hz, 1H), 7.07 (d, J = 8.7 Hz, 1H), 4.89 (s, 1H), 2.49 (s, 3H), 1.41 (s, 9H); **^{13}C NMR (151 MHz, $CDCl_3$)** δ 151.04, 150.04, 138.86, 138.24, 136.28, 133.40, 132.30, 131.51, 130.78, 129.88, 129.55, 128.27, 127.62, 126.98, 126.88, 126.59, 126.02, 125.74, 125.61, 124.87, 124.78, 122.81, 117.99, 116.49, 34.54, 31.40, 15.44; **HRMS(ESI)**: Calcd. for $C_{31}H_{27}OS[M-H]^-$ 447.1788; found 447.1785.

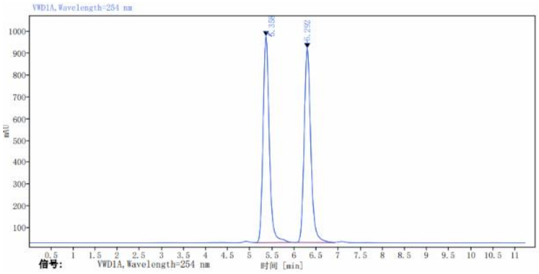

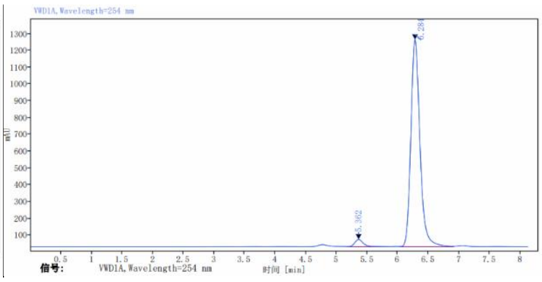

Peak	RetTime [min]	Type	Width[min]	Area[mAU*s]	Height[mAU]	Area%
	5.358	VB	0.9145	9355.3010	946.3549	49.9610
	6.292	BV	0.8913	9369.9226	890.3734	50.0390

Peak	RetTime [min]	Type	Width[min]	Area[mAU*s]	Height[mAU]	Area%
	5.362	VB	0.6888	438.1829	43.6888	3.3188
	6.284	VV	0.8848	12764.7511	1231.8163	96.6812

(*S*)-2'-((*R*)-methylsulfinyl)-6-(p-tolyl)-[1,1'-binaphthalen]-2-ol (3l):

White solid (40.6 mg, 48%, -10 ^{o}C for 13.5 h); m.p. = 189-191 ^{o}C; R_f = 0.23 (petroleum ether/ethyl acetate = 1:2); the enantiomeric excess was determined to be 80% by HPLC analysis

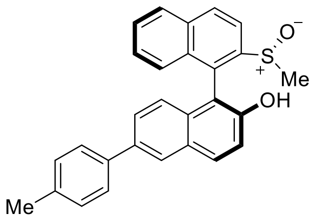

on Daicel Chirapak IA-H column (hexane/isopropanol = 90/10, flow rate 1 mL/min, T = 30 °C), UV 254 nm, t_R(major) 13.697 min, t_R(minor) 11.080 min; $[\alpha]_D^{25}$ = +233.94 (*c* = 0.44, $CHCl_3$); **^{1}H NMR (400 MHz, $CDCl_3$)** δ 8.63 (s, 1H), 8.13 – 8.01 (m, 2H), 7.94 (d, J = 1.9 Hz, 1H), 7.85 (dd, J = 11.7, 8.5 Hz, 2H), 7.49 – 7.43 (m, 3H), 7.38 – 7.33 (m, 2H), 7.24 – 7.30 (m, 2H) 7.19 – 7.15 (m, 2H), 6.73 (d, J = 8.8 Hz, 1H), 2.31 (s, 3H), 2.25 (s, 3H); **^{13}C NMR (101 MHz, $CDCl_3$)** δ 152.56, 141.80, 137.84, 136.97, 135.75, 135.00, 132.98, 132.50, 132.15, 131.09, 130.38, 129.60, 128.78, 128.48, 127.80, 127.48, 126.91, 126.64, 126.55, 126.06, 124.26, 119.28, 119.07, 113.40, 42.02, 21.11; **HRMS(ESI)**: Calcd. for $C_{28}H_{21}O_2S[M-H]^-$ 421.1268; found 421.1267.

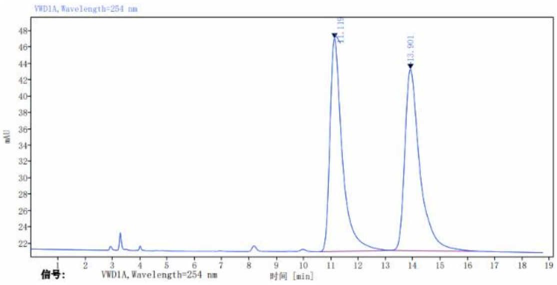

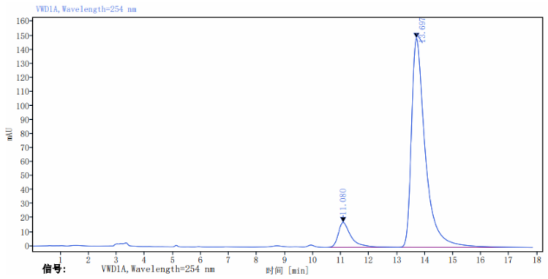

Peak	RetTime [min]	Type	Width[min]	Area[mAU*s]	Height[mAU]	Area%
	11.119	BB	2.6317	820.4925	26.0678	49.0348
	13.901	BB	3.3667	852.7938	22.1823	50.9652

Peak	RetTime [min]	Type	Width[min]	Area[mAU*s]	Height[mAU]	Area%
	11.080	BB	2.5750	566.8703	17.6776	9.9515
	13.697	BBA	4.8133	5129.4494	148.9388	90.0485

(*R*)-2'-(methylthio)-6-(p-tolyl)-[1,1'-binaphthalen]-2-ol (1l):

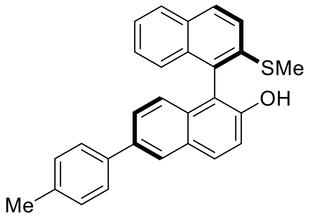

White semi-solid (39.9 mg, 49%, -10 °C for 13.5 h); R_f = 0.36 (petroleum ether/ethyl acetate = 5:1); the enantiomeric excess was determined to be 83% by HPLC analysis on Daicel Chirapak IA-H column (hexane/isopropanol = 70/30, flow rate 1 mL/min, T = 30 °C), UV 254 nm, t_R(major) 10.484 min, t_R(minor) 8.063 min; $[\alpha]_D^{25}$ = -49.95 (*c* = 0.42, $CHCl_3$); **^{1}H NMR (400 MHz, $CDCl_3$)** δ 8.07 – 7.96 (m, 3H), 7.92 (d, J = 8.2 Hz, 1H), 7.60 (d, J = 8.8 Hz, 3H), 7.51 – 7.42 (m, 3H), 7.39 – 7.29 (m, 2H), 7.24 – 7.18 (m, 2H), 7.02 (d, J = 8.7 Hz, 1H), 4.86 (s, 1H), 2.45 (s, 3H), 2.39 (s, 3H); **^{13}C NMR (101 MHz, $CDCl_3$)** δ 151.03, 138.85, 138.24, 136.79, 136.30, 133.38, 132.26, 131.48, 130.76, 129.87, 129.52, 128.27, 128.23, 127.62, 127.06, 126.51, 125.92, 125.60, 124.83, 124.79, 124.36, 122.76, 118.01, 116.45, 21.11, 15.42; **HRMS(ESI)**: Calcd. for $C_{28}H_{21}OS[M-H]^-$ 405.1319; found 405.1316.

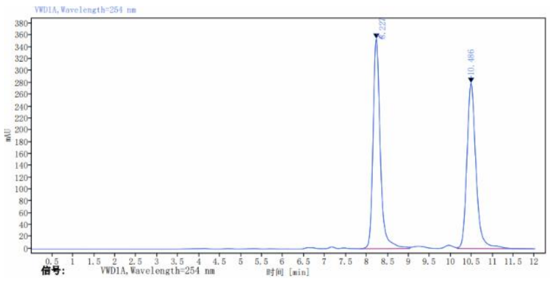

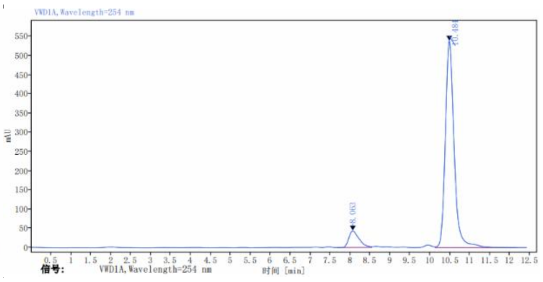

Peak	RetTime [min]	Type	Width[min]	Area[mAU*s]	Height[mAU]	Area%
	8.227	BV	1.1978	4269.1825	353.8928	50.3228
	10.486	VBA	1.8174	4214.4053	279.0360	49.6772

Peak	RetTime [min]	Type	Width[min]	Area[mAU*s]	Height[mAU]	Area%
	8.063	BV	0.8762	781.0020	43.4965	8.6391
	10.484	VB	2.0609	8259.3383	537.9897	91.3609

(*S*)-6-(4-fluorophenyl)-2'-((*R*)-methylsulfinyl)-[1,1'-binaphthalen]-2-ol (3m):

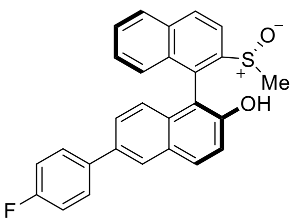

White solid (41 mg, 48%, -10 ^{o}C for 13.5 h); m.p. = 158-160 ^{o}C; R_f = 0.21 (petroleum ether/ethyl acetate = 1:2); the enantiomeric excess was determined to be 91% by HPLC analysis on Daicel Chirapak IA-H column (hexane/isopropanol = 90/10, flow rate 1 mL/min, T = 30 °C), UV 254 nm, t_R(major) 15.298 min, t_R(minor) 13.422 min; $[\alpha]_D^{25}$ = +359.42 (*c* = 0.80, $CHCl_3$); **^{1}H NMR (600 MHz, DMSO-*d*$_6$)** δ 10.09 (s, 1H), 8.34 (d, J = 8.7 Hz, 1H), 8.22 (s, 1H), 8.17 (d, J = 8.7 Hz, 1H), 8.13 (d, J = 8.2 Hz, 1H), 8.08 (d, J = 8.9 Hz, 1H), 7.78 – 7.74 (m, 2H), 7.65 – 7.59 (m, 1H), 7.53 (d, J = 8.8 Hz, 1H), 7.45 (d, J = 8.9 Hz, 1H), 7.43 – 7.36 (m, 1H), 7.33 – 7.21 (m, 2H), 7.16 (d, J = 8.5 Hz, 1H), 6.71 (d, J = 8.8 Hz, 1H), 2.24 (s, 3H); **^{13}C NMR (151 MHz, DMSO-*d*$_6$)** δ 163.04, 161.43, 153.30, 143.91, 136.77, 137.75, 134.80, 133.94, 133.35, 132.31, 132.24, 131.45, 130.14, 129.08, 129.02, 128.56, 127.94, 127.82, 126.46, 126.28, 124.62, 119.69, 119.47, 116.27, 116.13, 113.65, 42.53; **HRMS(ESI)**: Calcd. for $C_{27}H_{18}FO_2S[M-H]^-$ 425.1017; found 425.1014.

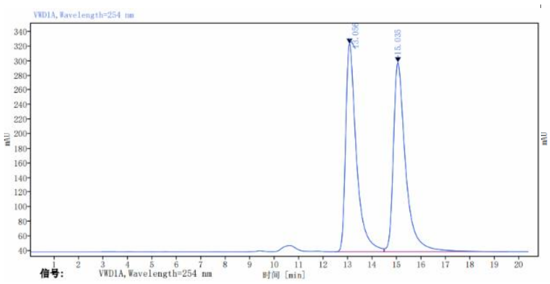

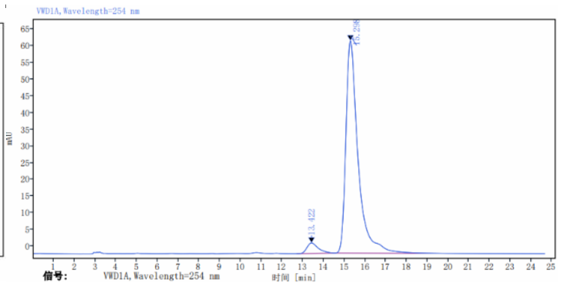

Peak	RetTime [min]	Type	Width[min]	Area[mAU*s]	Height[mAU]	Area%
	13.056	BV	2.0168	8694.0380	284.7842	49.0717
	15.035	VBA	5.0632	9022.9892	259.2339	50.9283

Peak	RetTime [min]	Type	Width[min]	Area[mAU*s]	Height[mAU]	Area%
	13.422	BB	1.8433	119.6933	3.1515	4.4003
	15.298	BB	5.1933	2600.4454	63.6430	95.5997

(*R*)-6-(4-fluorophenyl)-2'-(methylthio)-[1,1'-binaphthalen]-2-ol (1m):

White semi-solid (38.6 mg, 47%, -10 ^{o}C for 13.5 h); R_f = 0.32 (petroleum ether/ethyl acetate =

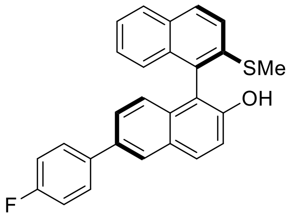

5:1); the enantiomeric excess was determined to be 88% by HPLC analysis on Daicel Chirapak IA-H column (hexane/isopropanol = 70/30, flow rate 1 mL/min, T = 30 °C), UV 254 nm, t_R(major) 7.671 min, t_R(minor) 6.182 min; $[\alpha]_D^{25}$ = -41.75(c = 0.78, $CHCl_3$); **^{1}H NMR (600 MHz, $CDCl_3$)** δ 7.92 (dd, J = 25.8, 9.9 Hz, 3H), 7.83 (d, J = 8.2 Hz, 1H), 7.53 – 7.50 (m, 3H), 7.35 (d, J = 8.3 Hz, 2H), 7.31 (d, J = 8.9 Hz, 1H), 7.23 (s, 1H), 7.11 (d, J = 8.5 Hz, 1H), 7.10 – 7.00 (m, 2H), 6.95 (d, J = 8.7 Hz, 1H), 4.82 (s, 1H), 2.36 (s, 3H); **^{13}C NMR (151 MHz, $CDCl_3$)** δ 151.22, 138.89, 137.27, 137.25, 135.39, 133.36, 132.35, 131.50, 130.75, 129.94, 129.46, 128.76, 128.70, 128.31, 127.66, 126.74, 126.37, 126.13, 125.63, 124.97, 124.76, 122.76, 118.23, 116.49, 115.72, 115.58, 15.41; **HRMS(ESI)**: Calcd. for $C_{27}H_{18}FOS[M-H]^-$ 409.1068; found 409.1066.

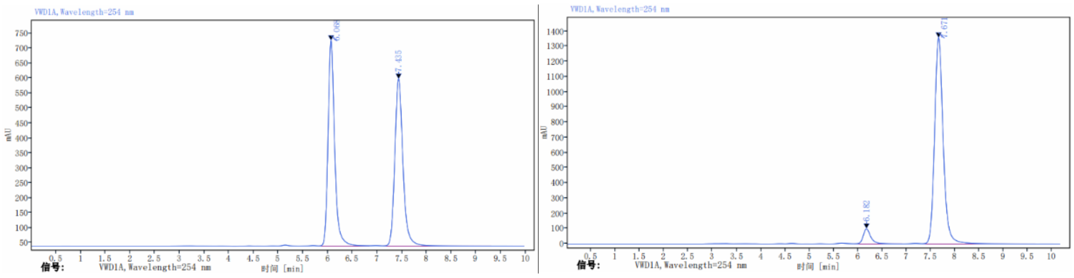

Peak	RetTime [min]	Type	Width[min]	Area[mAU*s]	Height[mAU]	Area%
	6.068	VV	1.0171	6407.9384	686.2282	50.0309
	7.435	VB	2.1840	6400.0332	557.9999	49.9691

Peak	RetTime [min]	Type	Width[min]	Area[mAU*s]	Height[mAU]	Area%
	6.182	VB	0.9815	1072.8573	99.1660	6.0017
	7.671	VB	2.3356	16803.0185	1357.5012	93.9983

(*S*)-6-(4-chlorophenyl)-2'-((*R*)-methylsulfinyl)-[1,1'-binaphthalen]-2-ol (3n):

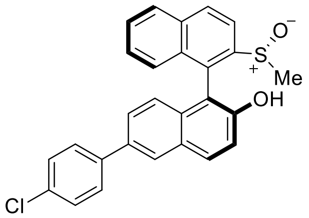

White solid (40.8 mg, 46%, -10 °C for 13 h); m.p. = 295-297 °C; R_f = 0.23 (petroleum ether/ethyl acetate = 1:2); the enantiomeric excess was determined to be 92% by HPLC analysis on Daicel Chirapak IA-H column (hexane/isopropanol = 90/10, flow rate 1 mL/min, T = 30 °C), UV 254 nm, t_R(major) 15.918 min, t_R(minor) 13.356 min; $[\alpha]_D^{25}$ = +244.68 (c = 0.84, $CHCl_3$); **^{1}H NMR (600 MHz, $CDCl_3$)** δ 9.01 (s, 1H), 8.09 – 9.98 (m, 2H), 7.91 (s, 1H), 7.86 – 7.80 (m, 2H), 7.50 – 7.43 (m, 3H), 7.38 (d, J = 8.9 Hz, 1H), 7.31 (d, J = 8.5 Hz, 3H), 7.23 (d, J = 3.8 Hz, 2H), 6.74 (d, J = 8.8 Hz, 1H), 2.25 (s, 3H); **^{13}C NMR (151 MHz, $CDCl_3$)** δ 152.79, 142.01, 139.19, 135.03, 134.61, 133.31, 133.22, 132.45, 131.73, 131.17, 130.54, 129.02, 128.70, 128.53, 128.29, 127.87, 127.56, 126.51, 126.37, 126.25, 124.50, 119.47, 119.08, 113.48, 42.08; **HRMS(ESI)**: Calcd. for $C_{27}H_{18}ClO_2S[M-H]^-$ 441.0722; found 441.0719.

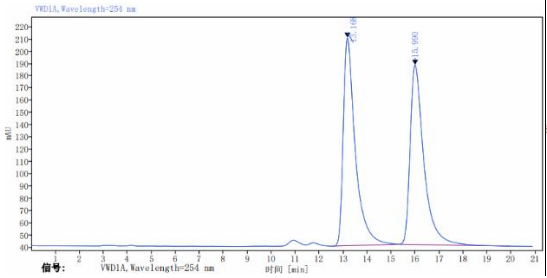

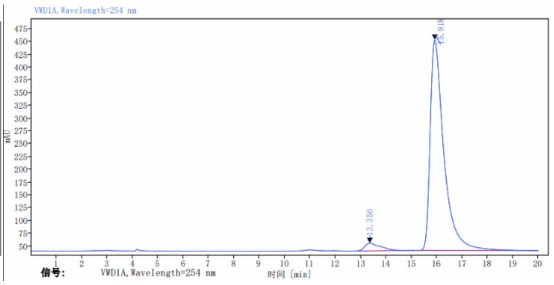

Peak	RetTime [min]	Type	Width[min]	Area[mAU^s]	Height[mAU]	Area%
	13.168	BB	2.7674	5922.4246	169.6138	50.3354
	15.990	BBA	4.6433	5843.5032	146.5097	49.6646

Peak	RetTime [min]	Type	Width[min]	Area[mAU^s]	Height[mAU]	Area%
	13.356	BB	2.0800	643.3479	14.9477	3.9768
	15.918	BBA	5.1633	15534.1097	412.0495	96.0232

(*R*)-6-(4-chlorophenyl)-2'-(methylthio)-[1,1'-binaphthalen]-2-ol (1n):

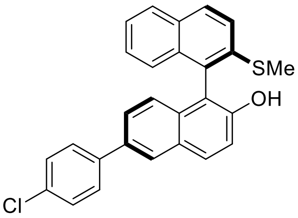

White semi-solid (41 mg, 48%, -10 °C for 13 h); R_f = 0.34 (petroleum ether/ethyl acetate = 5:1); the enantiomeric excess was determined to be 86% by HPLC analysis on Daicel Chirapak IA-H column (hexane/isopropanol = 70/30, flow rate 1 mL/min, T = 30 °C), UV 254 nm, t_R(major) 7.720 min, t_R(minor) 6.313 min; $[\alpha]_D^{25}$ = -49.58 (*c* = 0.79, $CHCl_3$); **^{1}H NMR (600 MHz, $CDCl_3$)** δ 7.92 (dd, J = 25.8, 9.9 Hz, 3H), 7.83 (d, J = 8.2 Hz, 1H), 7.53 – 7.50 (m, 3H), 7.35 (d, J = 8.3 Hz, 2H), 7.31 (d, J = 8.9 Hz, 1H), 7.23 (s, 1H), 7.11 (d, J = 8.5 Hz, 1H), 7.10 – 7.02 (m, 2H), 6.95 (d, J = 8.7 Hz, 1H), 4.82 (s, 1H), 2.36 (s, 3H); **^{13}C NMR (151 MHz, $CDCl_3$)** δ 151.39, 139.58, 138.89, 135.08, 133.36, 133.15, 132.55, 131.49, 130.83, 129.96, 129.44, 128.96, 128.44, 128.33, 127.68, 126.71, 126.23, 126.18, 125.64, 125.07, 124.74, 122.74, 118.33, 116.53, 15.41; **HRMS(ESI)**: Calcd. for $C_{27}H_{18}ClOS[M-H]^-$ 425.0772; found 425.0769.

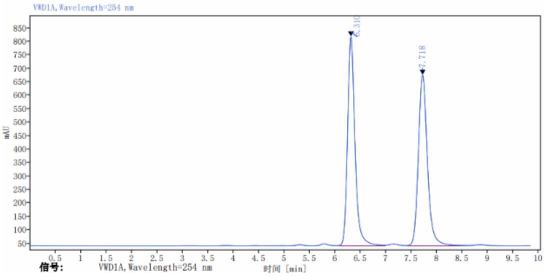

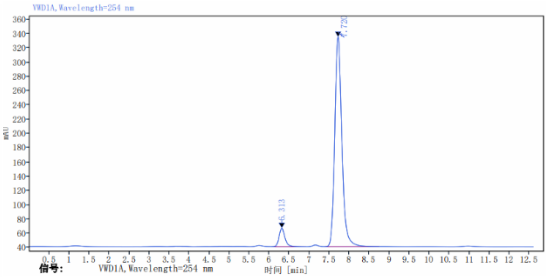

Peak	RetTime [min]	Type	Width[min]	Area[mAU^s]	Height[mAU]	Area%
	6.310	VV	0.9591	7890.3058	776.5924	50.1296
	7.718	VV	1.2195	7849.5003	633.8885	49.8704

Peak	RetTime [min]	Type	Width[min]	Area[mAU^s]	Height[mAU]	Area%
	6.313	VB	0.8587	271.6364	25.9837	6.9156
	7.720	VB	1.3644	3656.2297	293.8528	93.0844

(*S*)-2'-((*R*)-methylsulfinyl)-6-(4-(trifluoromethyl)phenyl)-[1,1'-binaphthalen]-2-ol (3o):

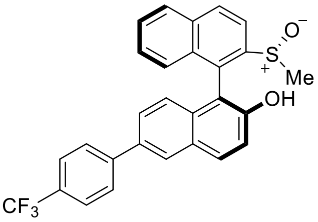

White solid (48.6 mg, 51%, -10 °C for 13.5 h); m.p. = 258-260 °C; R_f = 0.21 (petroleum ether/ethyl acetate = 1:2); the enantiomeric excess was determined to be 80% by HPLC analysis on Daicel

Chirapak IA-H column (hexane/isopropanol = 90/10, flow rate 1 mL/min, T = 30 °C), UV 254 nm, t_R(major) 13.650 min, t_R(minor) 11.986 min; $[\alpha]_D^{25}$ = +201.80 (*c* = 0.91, $CHCl_3$); **^{1}H NMR (600 MHz, $CDCl_3$)** δ 8.89 (s, 1H), 8.13 – 8.02 (m, 2H), 7.99 (s, 1H), 7.87 (d, J = 8.5 Hz, 2H), 7.66 (d, J = 8.1 Hz, 2H), 7.61 (d, J = 8.1 Hz, 2H), 7.50 – 7.44 (m, 1H), 7.40 (d, J = 8.9 Hz, 1H), 7.36 (d, J = 8.8 Hz, 1H), 7.26 (d, J = 7.0 Hz, 2H), 6.78 (d, J = 8.7 Hz, 1H), 2.27 (s, 3H); **^{13}C NMR (151 MHz, $CDCl_3$)** δ 153.28, 144.30, 141.67, 135.04, 134.23, 133.61, 132.42, 132.03, 131.26, 130.49, 129.54, 129.32, 129.11, 128.90, 128.57, 128.54, 127.86, 127.53, 127.30, 126.97, 126.54, 126.21, 125.81, 125.79, 125.23, 124.60, 123.43, 119.70, 119.04, 113.52, 42.02; **HRMS(ESI)**: Calcd. for $C_{28}H_{18}F_3O_2S[M-H]^-$ 475.0985; found 475.0980.

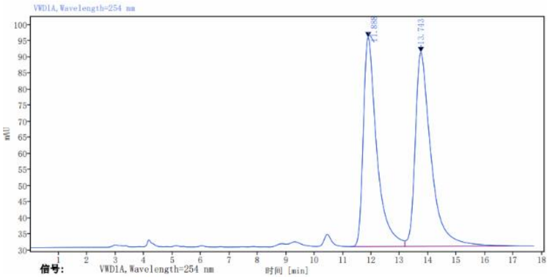

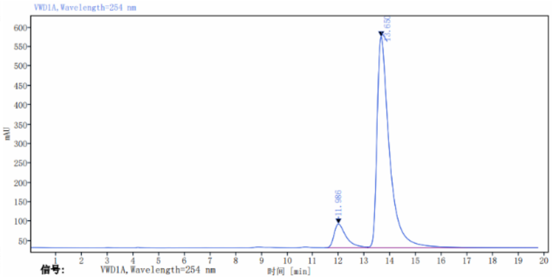

Peak	RetTime [min]	Type	Width[min]	Area[mAU^s]	Height[mAU]	Area%
	11.888	BV	1.9214	2138.1920	64.9969	47.7194
	13.743	VB	4.1369	2342.5684	60.3146	52.2806

Peak	RetTime [min]	Type	Width[min]	Area[mAU^s]	Height[mAU]	Area%
	11.986	BV	1.6874	2037.4023	61.4750	9.9453
	13.650	VBA	6.6359	18448.7641	543.9476	90.0547

(*R*)-2'-(methylthio)-6-(4-(trifluoromethyl)phenyl)-[1,1'-binaphthalen]-2-ol (1o):

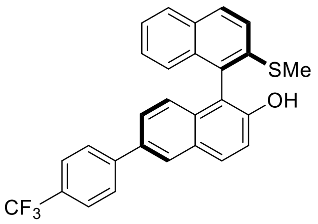

White semi-solid (39.6 mg, 43%, -10 °C for 13.5 h); R_f = 0.32 (petroleum ether/ethyl acetate = 5:1); the enantiomeric excess was determined to be 97% by HPLC analysis on Daicel Chirapak IA-H column (hexane/isopropanol = 70/30, flow rate 1 mL/min, T = 30 °C), UV 254 nm, t_R(major) 6.627 min, t_R(minor) 5.575 min; $[\alpha]_D^{25}$ = -47.74 (*c* = 0.86, $CHCl_3$); **^{1}H NMR (600 MHz, $CDCl_3$)** δ 8.09 (s, 1H), 8.02 (dd, J = 16.0, 8.9 Hz, 2H), 7.92 (d, J = 8.2 Hz, 1H), 7.75 (d, J = 8.1 Hz, 2H), 7.68 (d, J = 8.2 Hz, 2H), 7.60 (d, J = 8.8 Hz, 1H), 7.50 – 7.40 (m, 3H), 7.34 – 7.28 (m, 1H), 7.18 (d, J = 8.5 Hz, 1H), 7.07 (d, J = 8.7 Hz, 1H), 2.45 (s, 3H); **^{13}C NMR (151 MHz, $CDCl_3$)** δ 151.64, 144.66, 138.93, 134.82, 133.34, 132.88, 131.50, 130.95, 130.02, 129.38, 129.19, 128.98, 128.36, 128.25, 127.71, 127.43, 126.81, 126.54, 126.17, 125.78, 125.76, 125.73, 125.71, 125.67, 125.21, 124.68, 122.73, 118.47, 15.40; **HRMS(ESI)**: Calcd. for $C_{28}H_{18}F_3OS[M-H]^-$ 459.1047; found 459.1031.

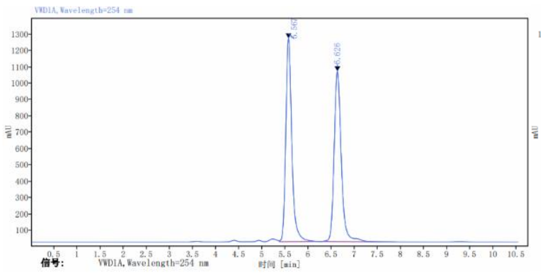

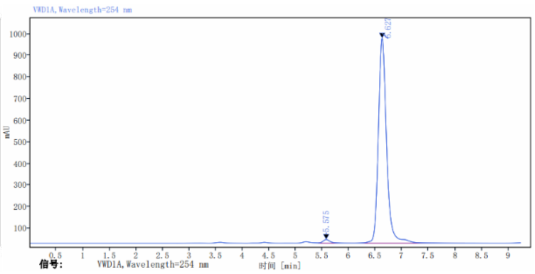

Peak	RetTime [min]	Type	Width[min]	Area[mAU*s]	Height[mAU]	Area%
	5.567	VB	0.8953	11280.5759	1240.2961	50.2000
	6.626	BB	1.6370	11190.6686	1038.7411	49.8000

Peak	RetTime [min]	Type	Width[min]	Area[mAU*s]	Height[mAU]	Area%
	5.575	VV	0.6649	163.0863	16.9735	1.5693
	6.627	VB	2.0970	10228.9214	948.4839	98.4307

4-((*S*)-2-hydroxy-2'-((*R*)-methylsulfinyl)-[1,1'-binaphthalen]-6-yl)benzonitrile (3p):

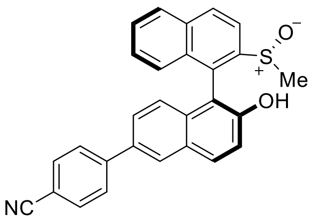

White solid (46 mg, 53%, -10 °C for 13.5 h); m.p. =175-177 °C; R_f = 0.18 (petroleum ether/ethyl acetate = 1:2); the enantiomeric excess was determined to be 71% by HPLC analysis on Daicel Chirapak IA-H column (hexane/isopropanol = 90/10, flow rate 1 mL/min, T = 30 °C), UV 254 nm, t_R(major) 34.774 min, t_R(minor) 27.193 min; $[\alpha]_D^{25}$ = +226.88 (*c* = 0.32, $CHCl_3$); **^{1}H NMR (600 MHz, $CDCl_3$)** δ 8.02 (s, 1H), 7.96 (dd, J = 20.2, 8.8 Hz, 2H), 7.85 (d, J = 8.2 Hz, 1H), 7.69 – 7.62 (m, 4H), 7.53 (d, J = 8.8 Hz, 1H), 7.41 – 7.33 (m, 3H), 7.27 – 7.20 (m, 1H), 7.09 (d, J = 8.6 Hz, 1H), 7.00 (d, J = 8.7 Hz, 1H), 4.90 (s, 1H), 2.38 (s, 3H); **^{13}C NMR (151 MHz, $CDCl_3$)** δ 153.46, 145.22, 141.74, 135.05, 133.81, 133.63, 132.69, 132.37, 131.78, 131.38, 130.60, 128.58, 128.52, 127.93, 127.60, 127.58, 127.19, 126.45, 125.92, 124.76, 119.81, 119.04, 118.96, 113.56, 110.71, 42.02; **HRMS(ESI)**: Calcd. for $C_{28}H_{18}NO_2S[M-H]^-$ 432.1064; found 432.1061.

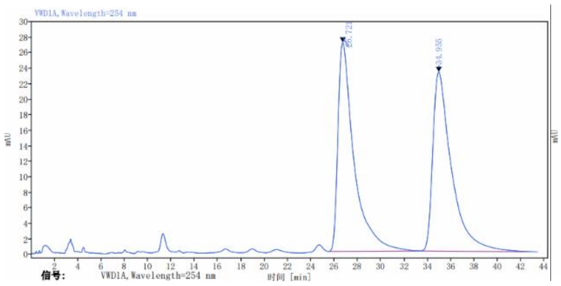

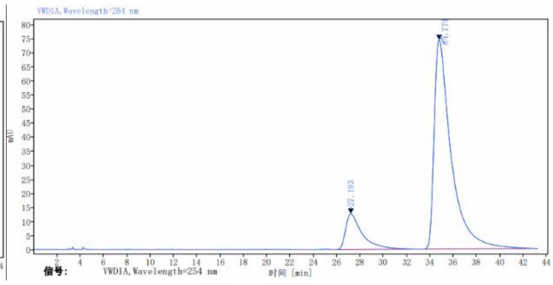

Peak	RetTime [min]	Type	Width[min]	Area[mAU*s]	Height[mAU]	Area%
	26.721	BB	7.8761	2578.4708	26.9055	49.9416
	34.955	BBA	10.0500	2584.5033	23.0726	50.0584

Peak	RetTime [min]	Type	Width[min]	Area[mAU*s]	Height[mAU]	Area%
	27.193	BB	7.2950	1272.6360	12.6598	14.4429
	34.774	BBA	9.9133	7538.8737	74.1726	85.5571

(*R*)-4-(2-hydroxy-2'-(methylthio)-[1,1'-binaphthalen]-6-yl)benzonitrile (1p):

White semi-solid (35.9 mg, 43%, -10 °C for 13.5 h); R_f = 0.22 (petroleum ether/ethyl acetate = 5:1); the enantiomeric excess was determined to be 89% by HPLC analysis on Daicel Chirapak

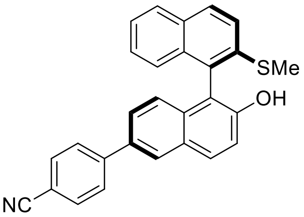

IA-H column (hexane/isopropanol = 70/30, flow rate 1 mL/min, T = 30 °C), UV 254 nm, t_R(major) 10.866 min, t_R(minor) 8.524 min; $[\alpha]_D^{25}$ = -71.67 (*c* = 0.26, $CHCl_3$); **^{1}H NMR (600 MHz, $CDCl_3$)** δ7.99 (s, 1H), 7.92 (t, J = 8.6 Hz, 2H), 7.82 (d, J = 8.1 Hz, 1H), 7.68 – 7.63 (m, 4H), 7.53 (d, J = 8.8 Hz, 1H), 7.41 – 7.32 (m, 3H), 7.26 – 7.20 (m, 1H), 7.15 – 7.09 (m, 1H), 6.96 (d, J = 8.7 Hz, 1H), 4.80 (s, 1H), 2.35 (s, 3H); **^{13}C NMR (151 MHz, $CDCl_3$)** δ 151.91, 145.62, 138.93, 134.18, 133.30, 133.12, 132.64, 131.50, 131.03, 130.08, 129.32, 128.39, 127.73, 127.70, 127.00, 126.37, 125.83, 125.69, 125.39, 124.60, 122.73, 119.05, 118.65, 116.62, 110.58, 15.40; **HRMS(ESI)**: Calcd. for $C_{28}H_{18}NOS[M-H]^-$ 416.1115; found 416.1111.

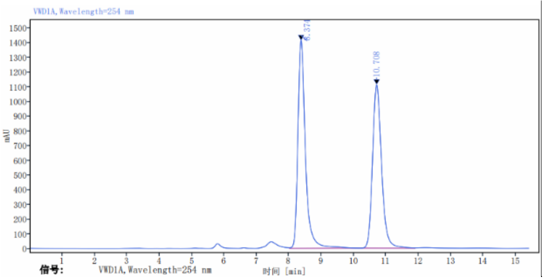

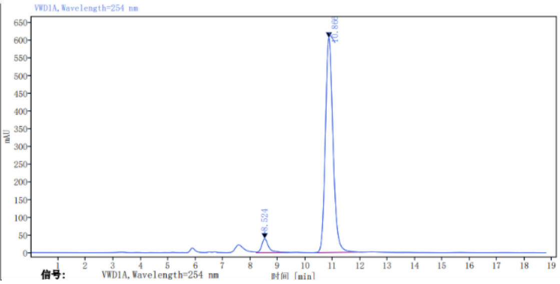

Peak	RetTime [min]	Type	Width[min]	Area[mAU^s]	Height[mAU]	Area%
	8.374	VB	2.1759	22908.5507	1415.4101	51.4882
	10.708	BV	1.7124	21584.2449	1112.7948	48.5118

Peak	RetTime [min]	Type	Width[min]	Area[mAU^s]	Height[mAU]	Area%
	8.524	VB	1.2160	682.4336	38.8683	5.4762
	10.866	BB	1.7928	11779.3105	606.2960	94.5238

(*S*)-6-(4-fluoro-3-methylphenyl)-2'-((*R*)-methylsulfinyl)-[1,1'-binaphthalen]-2-ol (3q):

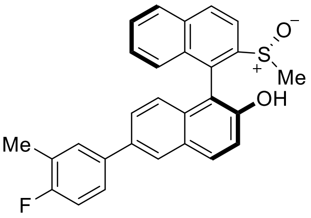

White solid (42.2 mg, 47%, -10 °C for 13 h); m.p. = 241-243 °C; R_f = 0.20 (petroleum ether/ethyl acetate = 1:2); the enantiomeric excess was determined to be 90% by HPLC analysis on Daicel Chirapak IA-H column (hexane/isopropanol = 90/10, flow rate 1 mL/min, T = 30 °C), UV 254 nm, t_R(major) 12.964 min, t_R(minor) 11.722 min; $[\alpha]_D^{25}$ = +177.83 (*c* = 0.66, $CHCl_3$); **^{1}H NMR (600 MHz, DMSO-*d*$_6$)** δ10.09 (s, 1H), 8.34 (d, J = 8.6 Hz, 1H), 8.21 (s, 1H), 8.15 (dd, J = 18.4, 8.5 Hz, 2H), 8.07 (d, J = 8.8 Hz, 1H), 7.69 – 7.60 (m, 2H), 7.58 – 7.51 (m, 2H), 7.45 – 7.38 (m, 2H), 7.24 – 7.18 (m, 1H), 7.15 (d, J = 8.4 Hz, 1H), 6.70 (d, J = 8.7 Hz, 1H), 2.30 (s, 3H), 2.24 (s, 3H); **^{13}C NMR (151 MHz, DMSO-*d*$_6$)** δ 161.61, 160.00, 153.23, 143.88, 136.49, 134.79, 134.13, 133.29, 132.24, 131.41, 130.41, 130.13, 129.02, 128.55, 127.95, 127.83, 126.50, 126.39, 126.34, 126.28, 125.19, 125.07, 124.54, 119.68, 119.42, 115.82, 113.61, 42.51, 14.77; **HRMS(ESI)**: Calcd. for $C_{28}H_{20}FO_2S[M-H]^-$ 439.1174; found 439.1170.

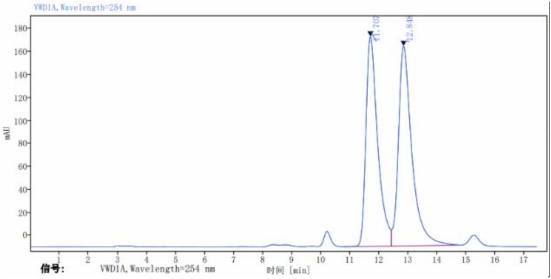

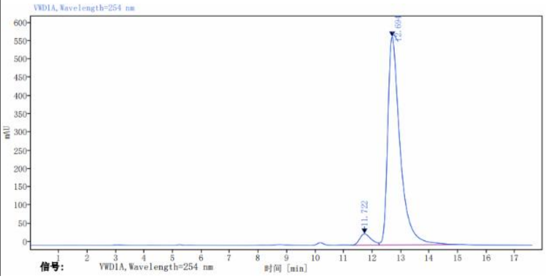

Peak	RetTime [min]	Type	Width[min]	Area[mAU*s]	Height[mAU]	Area%
	11.707	BV	1.6231	5140.2550	182.6395	47.8185
	12.848	VB	2.3877	5609.2596	173.9907	52.1815

Peak	RetTime [min]	Type	Width[min]	Area[mAU*s]	Height[mAU]	Area%
	11.722	VV	0.9987	871.0488	30.9169	4.8230
	12.694	VB	2.7660	17189.2556	569.8657	95.1770

(*R*)-6-(4-fluoro-3-methylphenyl)-2'-(methylthio)-[1,1'-binaphthalen]-2-ol (1q):

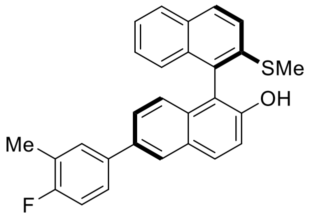

White semi-solid (40.7 mg, 47%, -10 °C for 13 h); R_f = 0.34 (petroleum ether/ethyl acetate = 5:1); the enantiomeric excess was determined to be 95% by HPLC analysis on Daicel Chirapak IA-H column (hexane/isopropanol = 70/30, flow rate 1 mL/min, T = 30 °C), UV 254 nm, t_R(major) 7.145 min, t_R(minor) 5.618 min; $[\alpha]_D^{25}$ = -42.81 (*c* = 0.76, $CHCl_3$); **^{1}H NMR (600 MHz, $CDCl_3$)** δ 7.98 – 7.88 (m, 3H), 7.84 (d, J = 8.2 Hz, 1H), 7.52 (d, J = 8.7 Hz, 1H), 7.39 – 7.29 (m, 5H), 7.25 – 7.21 (m, 1H), 7.11 (d, J = 8.5 Hz, 1H), 7.01 – 6.92 (m, 2H), 4.82 (s, 1H), 2.36 (s, 3H), 2.26 (s, 3H); **^{13}C NMR (151 MHz, $CDCl_3$)** δ 161.78, 160.16, 151.16, 138.87, 136.99, 135.62, 133.37, 132.30, 131.49, 130.74, 130.32, 129.92, 129.47, 128.30, 127.65, 126.44, 126.05, 125.64, 125.63, 124.89, 124.79, 124.37, 122.76, 118.18, 116.48, 115.35, 115.20, 31.76, 15.42; **HRMS(ESI)**: Calcd. for $C_{28}H_{20}FOS[M-H]^-$ 423.1224; found 423.1221.

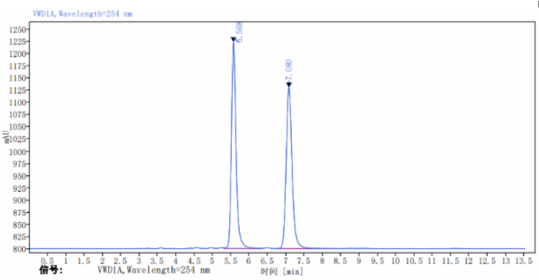

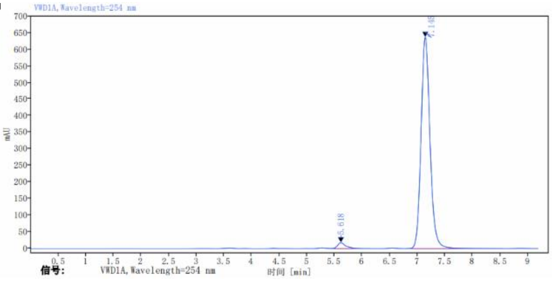

Peak	RetTime [min]	Type	Width[min]	Area[mAU*s]	Height[mAU]	Area%
	5.568	VV	1.0338	3720.4416	421.0053	50.0972
	7.080	BB	1.6267	3706.0108	328.4747	49.9028

Peak	RetTime [min]	Type	Width[min]	Area[mAU*s]	Height[mAU]	Area%
	5.618	VB	0.5497	179.3190	17.5770	2.4396
	7.145	BB	1.7800	7170.9990	636.4282	97.5604

(*S*)-3-bromo-2'-((*R*)-methylsulfinyl)-[1,1'-binaphthalen]-2-ol (3r):

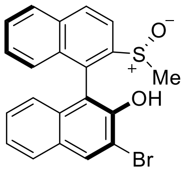

White solid (40.1 mg, 49%, -10 °C for 24 h); m.p. = 236-238 °C; R_f = 0.3 (petroleum ether/ethyl acetate = 1:2); the enantiomeric excess was determined to be 70% by HPLC analysis on Daicel Chirapak OJ-H column

(hexane/isopropanol = 90/10, flow rate 1 mL/min, T = 30 °C), UV 254 nm, t_R(major) 14.252 min, t_R(minor) 10.757 min; $[\alpha]_D^{25}$ = +185.00 (*c* = 0.78, $CHCl_3$); **^{1}H NMR (600 MHz, $CDCl_3$)** δ 8.17 (s, 1H), 8.05 (d, J = 5.9 Hz, 2H), 7.92 (d, J = 8.3 Hz, 1H), 7.72 (d, J = 8.2 Hz, 1H), 7.50 (d, J = 8.1 Hz, 1H), 7.31 – 7.22 (m, 2H), 7.14 (dd, J = 8.6, 5.2 Hz, 2H), 6.71 (s, 1H), 6.68 – 6.64 (m, 1H), 2.14 (s, 3H); **^{13}C NMR (151 MHz, $CDCl_3$)** δ 147.81, 142.93, 134.87, 133.14, 132.89, 132.17, 130.68, 130.66, 129.29, 128.60, 127.78, 127.71, 127.59, 127.57, 126.15, 124.59, 124.26, 119.36, 115.55, 113.30, 42.23; **HRMS(ESI)**: Calcd. for $C_{21}H_{14}BrO_2S[M-H]^-$ 408.9903; found 408.9900.

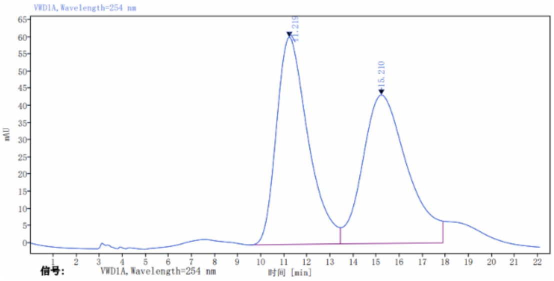

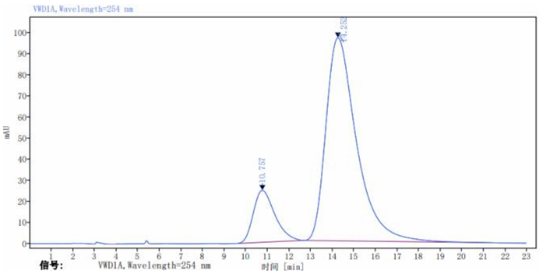

Peak	RetTime [min]	Type	Width[min]	Area[mAU^s]	Height[mAU]	Area%
	11.219	MM m	1.4451	5739.1853	60.3691	49.6885
	15.210	MM m	1.9769	5811.1395	43.2689	50.3115

Peak	RetTime [min]	Type	Width[min]	Area[mAU^s]	Height[mAU]	Area%
	10.757	BB	3.1494	1699.9094	24.5766	15.2095
	14.252	BBA	9.1867	9476.6854	96.1312	84.7905

(*R*)-3-bromo-2'-(methylthio)-[1,1'-binaphthalen]-2-ol (1r):

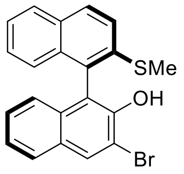

White semi-solid (40.5 mg, 51%, -10 °C for 24 h); R_f = 0.65 (petroleum ether/ethyl acetate = 3:1); the enantiomeric excess was determined to be 75% by HPLC analysis on Daicel Chirapak IA-H column (hexane/isopropanol = 70/30, flow rate 1 mL/min, T = 30 °C), UV 254 nm, t_R(major) 12.042 min, t_R(minor) 5.754 min; $[\alpha]_D^{25}$ = +8.99 (*c* = 0.76, $CHCl_3$); **^{1}H NMR (600 MHz, $CDCl_3$)** δ 8.22 (s, 1H), 8.01 (d, J = 8.8 Hz, 1H), 7.90 (d, J = 8.2 Hz, 1H), 7.79 (d, J = 8.2 Hz, 1H), 7.59 (d, J = 8.8 Hz, 1H), 7.45 – 7.39 (m, 1H), 7.37 – 7.32 (m, 1H), 7.32 – 7.26 (m, 1H), 7.25 (s, 1H), 7.10 (d, J = 8.5 Hz, 1H), 6.96 (d, J = 8.3 Hz, 1H), 5.40 (s, 1H), 2.43 (s, 3H); **^{13}C NMR (151 MHz, $CDCl_3$)** δ 147.37, 137.93, 132.99, 132.77, 132.32, 131.54, 129.77, 129.69, 128.27, 128.21, 127.44, 127.31, 127.19, 125.53, 124.77, 124.72, 124.57, 123.25, 118.66, 112.11, 15.69; **HRMS(ESI)**: Calcd. for $C_{21}H_{14}BrOS[M-H]^-$ 392.9954; found 392.9951.

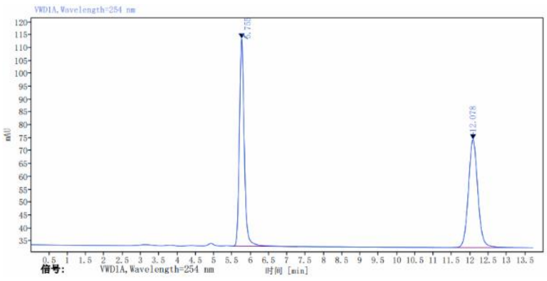

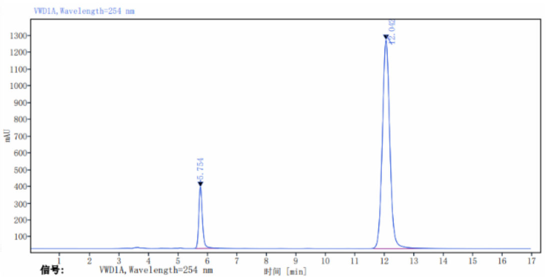

Peak	RetTime [min]	Type	Width[min]	Area[mAU*s]	Height[mAU]	Area%
	5.755	BB	1.7833	750.8278	80.5213	50.0908
	12.078	BB	1.6733	748.1048	41.8710	49.9092

Peak	RetTime [min]	Type	Width[min]	Area[mAU*s]	Height[mAU]	Area%
	5.754	BB	0.8745	3114.6557	365.3180	12.3888
	12.042	BB	4.1150	22026.2806	1243.1849	87.6112

4-methyl-2-((*S*)-2-((*R*)-methylsulfinyl)naphthalen-1-yl)phenol (5a):

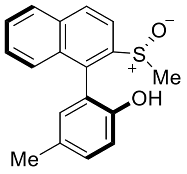

White solid (26.1 mg, 44%, -10 °C for 12.5 h); m.p. = 236-238 °C; R_f = 0.25 (petroleum ether/ethyl acetate = 1:2); the enantiomeric excess was determined to be 77% by HPLC analysis on Daicel Chirapak IA-H column (hexane/isopropanol = 90/10, flow rate 1 mL/min, T = 30 °C), UV 254 nm, t_R(major) 10.260 min, t_R(minor) 8.912 min; $[\alpha]_D^{25}$ = +88.56 (*c* = 0.3, $CHCl_3$); **^{1}H NMR (400 MHz, DMSO-*d*$_6$)** δ 9.39 (s, 1H), 8.21 (d, J = 8.7 Hz, 1H), 8.05 (dd, J = 8.5, 5.5 Hz, 2H), 7.63 (d, J = 8.1 Hz, 1H), 7.52 (d, J = 8.2 Hz, 1H), 7.43 (d, J = 8.6 Hz, 1H), 7.14 (d, J = 8.3 Hz, 1H), 6.95 – 6.89 (m, 2H), 2.56 (s, 3H), 2.26 (s, 3H); **^{13}C NMR (101 MHz, DMSO-*d*$_6$)** δ 157.33, 147.54, 139.49, 139.36, 137.43, 136.90, 135.64, 134.27, 133.47, 132.78, 132.53, 132.31, 131.23, 126.42, 121.01, 47.55, 25.25; **HRMS(ESI)**: Calcd. for $C_{18}H_{15}O_2S[M-H]^-$ 295.0798; found 295.0797.

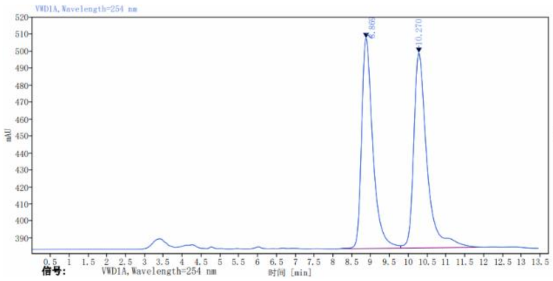

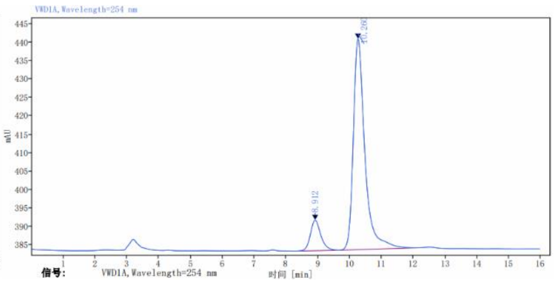

Peak	RetTime [min]	Type	Width[min]	Area[mAU*s]	Height[mAU]	Area%
	8.869	BV	1.7203	2582.2644	123.7969	47.9642
	10.270	VB	2.2013	2801.4651	114.8161	52.0358

Peak	RetTime [min]	Type	Width[min]	Area[mAU*s]	Height[mAU]	Area%
	8.912	BB	1.3417	189.1780	8.2709	11.5826
	10.260	BB	2.4728	1444.1150	57.1161	88.4174

(*R*)-4-methyl-2-(2-(methylthio)naphthalen-1-yl)phenol (4a):

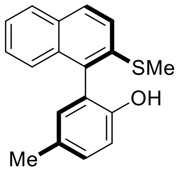

White solid (29.6 mg, 53%, -10 °C for 12.5 h); m.p. = 100-102 °C; R_f = 0.32 (petroleum ether/ethyl acetate = 5:1); the enantiomeric excess was determined to be 87% by HPLC analysis on Daicel Chirapak IA-H column (hexane/isopropanol = 70/30, flow rate 1 mL/min, T = 30 °C), UV 254 nm, t_R(major) 6.525 min,

t_R(minor) 4.820 min; $[\alpha]_D^{25}$ = -40.63 (*c* = 0.48, $CHCl_3$); **^{1}H NMR (400 MHz, $CDCl_3$)** δ 7.90 (d, J = 8.8 Hz, 1H), 7.84 (d, J = 7.6 Hz, 1H), 7.48 (d, J = 8.8 Hz, 1H), 7.44 – 7.37 (m, 3H), 7.20 (d, J = 8.3 Hz, 1H), 7.02 – 6.94 (m, 2H), 4.56 (s, 1H), 2.47 (s, 3H), 2.34 (s, 3H); **^{13}C NMR (101 MHz, $CDCl_3$)** δ 150.89, 137.08, 133.03, 131.71, 131.35, 130.68, 130.21, 130.15, 129.28, 128.11, 127.26, 125.41, 125.00, 123.76, 122.81, 115.81, 20.62, 15.70; **HRMS(ESI)**: Calcd. for $C_{18}H_{15}OS[M-H]^-$ 279.0849; found 279.0846.

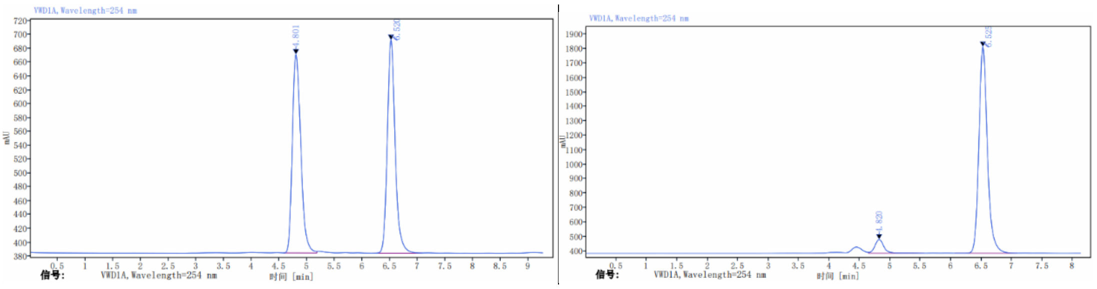

Peak	RetTime [min]	Type	Width[min]	Area[mAU*s]	Height[mAU]	Area%
	4.801	BV	0.6025	3050.6038	286.3348	50.0720
	6.520	BV	0.8975	3041.8271	307.5076	49.9280

Peak	RetTime [min]	Type	Width[min]	Area[mAU*s]	Height[mAU]	Area%
	4.820	VB	0.9157	1003.7792	94.4237	6.7372
	6.525	BV	1.0213	13895.2134	1426.7362	93.2628

4-(*tert*-butyl)-2-((*S*)-2-((*R*)-methylsulfinyl)naphthalen-1-yl)phenol (5b):

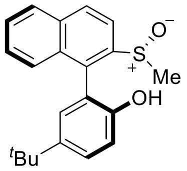

White solid (30.4 mg, 45%, -10 °C for 12 h); m.p. = 194-196 °C; R_f = 0.23 (petroleum ether/ethyl acetate = 1:2); the enantiomeric excess was determined to be 74% by HPLC analysis on Daicel Chirapak IA-H column (hexane/isopropanol = 90/10, flow rate 0.8 mL/min, T = 30 °C), UV 254 nm, t_R(major) 10.047 min, t_R(minor) 8.864 min; $[\alpha]_D^{25}$ = +149.19 (*c* = 0.58, $CHCl_3$); **^{1}H NMR (400 MHz, DMSO-*d_6*)** δ 9.54 (s, 1H), 8.21 (d, J = 8.7 Hz, 1H), 8.05 (dd, J = 8.6, 3.0 Hz, 2H), 7.61 (d, J = 8.2 Hz, 1H), 7.51 (d, J = 8.2 Hz, 1H), 7.42 (d, J = 8.5 Hz, 1H), 7.34 (d, J = 8.5 Hz, 1H), 7.03 (s, 1H), 6.95 (d, J = 8.5 Hz, 1H), 2.56 (s, 3H), 1.24 (s, 9H); **^{13}C NMR (101 MHz, DMSO-*d_6*)** δ 150.58, 143.68, 137.27, 133.00, 131.37, 129.22, 128.62, 128.13, 127.29, 126.79, 126.43, 125.40, 125.02, 123.04, 115.29, 114.75, 31.59, 31.54, 15.79; **HRMS(ESI)**: Calcd. for $C_{21}H_{21}O_2S[M-H]^-$ 337.1268; found 337.1268.

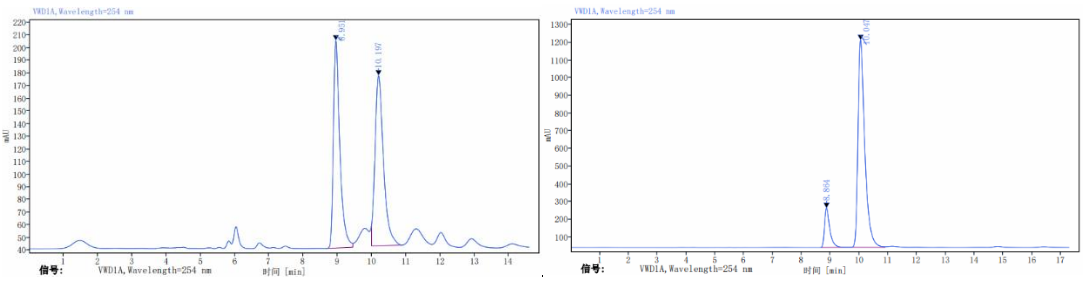

Peak	RetTime [min]	Type	Width[min]	Area[mAU^s]	Height[mAU]	Area%
	8.951	BV	0.7734	2146.7087	163.7457	48.0970
	10.197	MB m	0.2603	2316.5842	134.2372	51.9030

Peak	RetTime [min]	Type	Width[min]	Area[mAU^s]	Height[mAU]	Area%
	8.864	BB	1.0342	2814.7792	221.7579	12.7710
	10.047	BV	1.2560	19225.5338	1170.4626	87.2290

(*R*)-4-(*tert*-butyl)-2-(2-(methylthio)naphthalen-1-yl)phenol (4b):

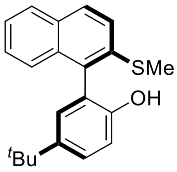

White semi-solid (33.0 mg, 51%, -10 °C for 12 h); R_f = 0.34 (petroleum ether/ethyl acetate = 5:1); the enantiomeric excess was determined to be 89% by HPLC analysis on Daicel Chirapak IA-H column (hexane/isopropanol = 90/10, flow rate 1 mL/min, T = 30 °C), UV 254 nm, t_R(major) 9.662 min, t_R(minor) 6.037 min; $[\alpha]_D^{25}$ = +0.24 (*c* = 0.56, $CHCl_3$); **^{1}H NMR (400 MHz, $CDCl_3$)** δ 7.91 (d, J = 8.8 Hz, 1H), 7.88 – 7.82 (m, 1H), 7.51 (d, J = 8.8 Hz, 1H), 7.47 – 7.34 (m, 3H), 7.24 (d, J = 5.3 Hz, 1H), 7.17 (d, J = 2.5 Hz, 1H), 7.03 (d, J = 8.5 Hz, 1H), 6.80 – 6.66 (m, 1H), 2.46 (s, 3H), 1.32 (s, 9H); **^{13}C NMR (101 MHz, $CDCl_3$)** δ 153.21, 150.58, 143.69, 143.47, 137.25, 133.01, 131.37, 130.76, 129.21, 128.63, 128.13, 127.29, 126.79, 126.41, 125.40, 125.03, 123.33, 123.05, 115.31, 114.77, 31.59, 31.54, 15.79; **HRMS(ESI)**: Calcd. for $C_{21}H_{21}OS[M-H]^-$ 321.1319; found 321.1322.

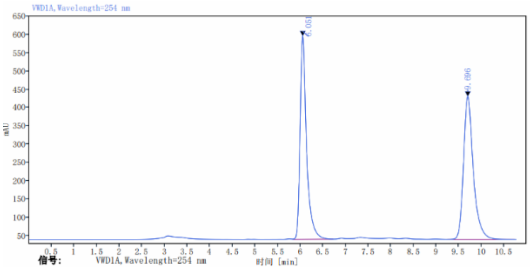

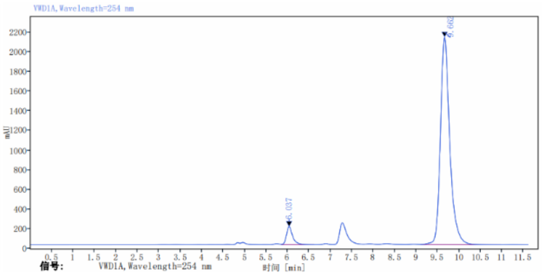

Peak	RetTime [min]	Type	Width[min]	Area[mAU^s]	Height[mAU]	Area%
	6.051	VB	0.8964	5507.3247	553.7188	48.4465
	9.696	BBA	1.4833	5860.5222	389.2491	51.5535

Peak	RetTime [min]	Type	Width[min]	Area[mAU^s]	Height[mAU]	Area%
	6.037	VB	0.7617	1858.7107	183.3913	5.4792
	9.662	VB	1.8706	32064.1681	2106.8377	94.5208

(*S*)-2-((*R*)-methylsulfinyl)-[1,2'-binaphthalen]-1'-ol (5c):

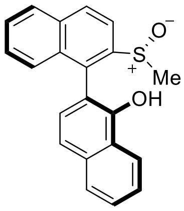

White solid (28.6 mg, 43%, -10 °C for 12 h); m.p. = 235-237 °C; R_f = 0.23 (petroleum ether/ethyl acetate = 1:2); the enantiomeric excess was determined to be 71% by HPLC analysis on Daicel Chirapak IA-H column (hexane/isopropanol = 92/8, flow rate 0.6 mL/min, T = 30 °C), UV 254 nm, t_R(major) 21.375 min, t_R(minor) 19.7615 min; $[\alpha]_D^{25}$ = +126.86 (*c* = 0.52, $CHCl_3$); **^{1}H NMR (400 MHz, DMSO-*d*$_6$)** δ 9.56 (s, 1H), 8.41 – 8.25 (m, 2H), 8.10 (d, J = 8.6 Hz, 2H), 7.96 (d, J = 7.2 Hz, 1H), 7.68 – 7.54 (m, 4H), 7.52 – 7.47 (m, 1H), 7.40 (d, J = 8.5 Hz, 1H), 7.20 (d, J = 8.3 Hz, 1H), 2.51 (s, 3H); **^{13}C NMR (101 MHz, DMSO-*d*$_6$)** δ 150.10, 143.44, 134.93, 134.82, 134.26, 132.53, 129.93, 129.79, 128.87, 128.26, 127.83, 127.68, 127.21, 126.38, 125.97, 125.74, 123.06, 119.85,

119.38, 116.08, 42.74; **HRMS(ESI)**: Calcd. for $C_{21}H_{15}O_2S[M-H]^-$ 331.0798; found 331.0798.

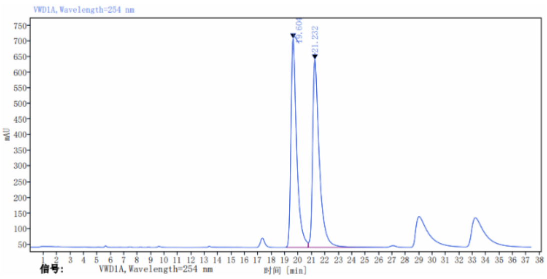

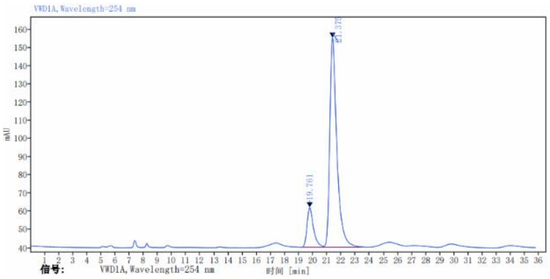

Peak	RetTime [min]	Type	Width[min]	Area[mAU*s]	Height[mAU]	Area%
	19.604	BV	1.8333	20070.4188	668.5368	48.7787
	21.232	VB	5.6017	21075.4293	601.0476	51.2213

Peak	RetTime [min]	Type	Width[min]	Area[mAU*s]	Height[mAU]	Area%
	19.761	BV	1.6003	694.3361	21.7973	14.7287
	21.375	VB	3.1613	4019.8501	115.1843	85.2713

(*R*)-2-(methylthio)-[1,2'-binaphthalen]-1'-ol (4c):

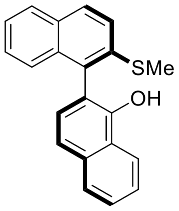

White semi-solid (32.9 mg, 53%, -10 °C for 12 h); R_f = 0.34 (petroleum ether/ethyl acetate = 5:1); the enantiomeric excess was determined to be 54% by HPLC analysis on Daicel Chirapak IA-H column (hexane/isopropanol = 70/30, flow rate 1 mL/min, T = 30 °C), UV 254 nm, t_R(major) 8.466 min, t_R(minor) 5.374 min; $[\alpha]_D^{25}$ = +8.17 (*c* = 0.60, $CHCl_3$); **^{1}H NMR (400 MHz, $CDCl_3$)** δ 8.39 – 8.27 (m, 1H), 7.96 (d, J = 8.8 Hz, 1H), 7.91 – 7.84 (m, 2H), 7.61 – 7.50 (m, 4H), 7.46 – 7.40 (m, 1H), 7.36 (dd, J = 3.6, 1.1 Hz, 2H), 7.24 (s, 1H), 5.17 (s, 1H), 2.47 (s, 3H); **^{13}C NMR (101 MHz, $CDCl_3$)** δ 148.71, 137.84, 134.84, 133.09, 131.44, 129.66, 129.58, 128.46, 128.23, 127.67, 127.45, 126.73, 125.52, 125.44, 124.94, 124.52, 122.81, 122.68, 120.57, 117.14, 15.61; **HRMS(ESI)**: Calcd. for $C_{21}H_{15}OS[M-H]^-$ 315.0849; found 315.0847.

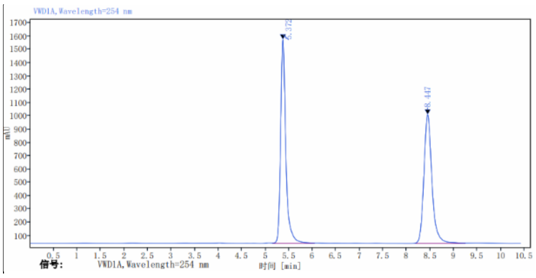

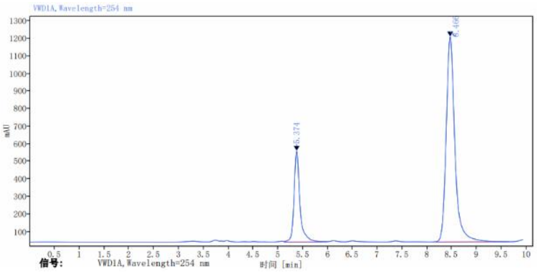

Peak	RetTime [min]	Type	Width[min]	Area[mAU*s]	Height[mAU]	Area%
	5.372	VV	0.8918	11936.5154	1533.2741	50.6897
	8.447	BV	1.3074	11611.6769	969.1624	49.3103

Peak	RetTime [min]	Type	Width[min]	Area[mAU*s]	Height[mAU]	Area%
	5.374	VV	0.8604	4280.2168	517.4317	23.1244
	8.466	BB	1.5539	14229.3450	1168.3711	76.8756

(*S*)-2'-((*R*)-ethylsulfinyl)-[1,1'-binaphthalen]-2-ol (5d):

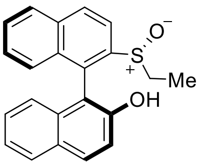

White semi-solid (33.2 mg, 48%, -10 °C for 16 h); R_f = 0.24 (petroleum ether/ethyl acetate = 1:2); the enantiomeric excess was determined to be 85% by HPLC analysis on Daicel Chirapak IA-H column (hexane/isopropanol =

90/10, flow rate 1 mL/min, T = 30 °C), UV 254 nm, t_R(major) 10.173 min, t_R(minor) 7.725 min; $[\alpha]_D^{25}$ = +209.90 (*c* = 0.66, $CHCl_3$); **^{1}H NMR (400 MHz, DMSO-d_6)** δ 10.01 (s, 1H), 8.29 (d, J = 8.7 Hz, 1H), 8.11 (d, J = 8.2 Hz, 1H), 8.05 (d, J = 8.7 Hz, 1H), 7.99 (d, J = 8.9 Hz, 1H), 7.92 (d, J = 8.0 Hz, 1H), 7.63 – 7.58 (m, 1H), 7.42 – 7.35 (m, 2H), 7.33 – 7.24 (m, 2H), 7.09 (d, J = 8.5 Hz, 1H), 6.63 (d, J = 8.3 Hz, 1H), 2.34 – 7.25 (m, 1H), 2.16 – 2.05 (m, 1H), 0.79 (t, J = 7.3 Hz, 3H); **^{13}C NMR (101 MHz, DMSO-d_6)** δ 157.77, 145.80, 139.47, 139.04, 137.56, 137.21, 135.77, 133.97, 133.73, 133.70, 132.94, 132.59, 132.38, 132.16, 130.93, 128.50, 128.06, 125.72, 123.72, 118.30, 51.64, 10.40; **HRMS(ESI)**: Calcd. for $C_{22}H_{17}O_2S[M-H]^-$ 345.0955; found 345.0951.

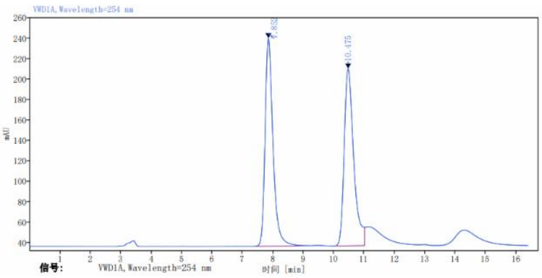

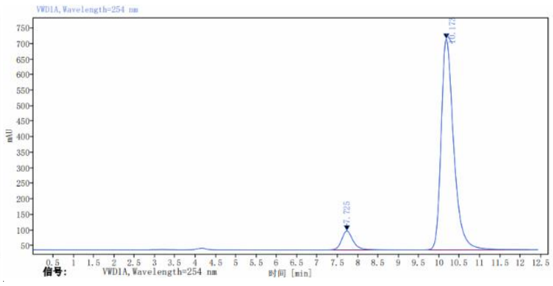

Peak	RetTime [min]	Type	Width[min]	Area[mAU^s]	Height[mAU]	Area%
	7.852	BB	1.8450	3686.9053	202.8902	49.5285
	10.475	BV	1.0404	3757.0952	172.8584	50.4715

Peak	RetTime [min]	Type	Width[min]	Area[mAU^s]	Height[mAU]	Area%
	7.725	BB	2.3967	1227.9803	61.6336	7.7414
	10.173	BB	2.6350	14634.5946	678.6787	92.2586

(*R*)-2'-(ethylthio)-[1,1'-binaphthalen]-2-ol (4d):

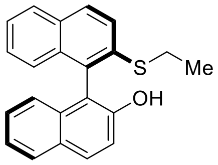

White semi-solid (31.9 mg, 49%, -10 °C for 16 h); R_f = 0.35 (petroleum ether/ethyl acetate = 5:1); the fenantiomeric excess was determined to be 85% by HPLC analysis on Daicel Chirapak IA-H column (hexane/isopropanol = 70/30, flow rate 1 mL/min, T = 30 °C), UV 254 nm, t_R(major) 6.494 min, t_R(minor) 4.903 min; $[\alpha]_D^{25}$ = -32.05 (*c* = 0.60, $CHCl_3$); **^{1}H NMR (400 MHz, $CDCl_3$)** δ 8.01 (dd, J = 12.4, 8.9 Hz, 2H), 7.96 – 7.89 (m, 2H), 7.68 (d, J = 8.8 Hz, 1H), 7.47 (d, J = 8.1 Hz, 1H), 7.44 – 7.36 (m, 1H), 7.35 (dd, J = 4.2, 1.3 Hz, 2H), 7.34 – 7.25 (m, 1H), 7.20 (d, J = 8.5 Hz, 1H), 7.03 (d, J = 8.4 Hz, 1H), 4.90 (s, 1H), 3.12 – 2.88 (m, 2H), 1.29 (t, J = 7.4 Hz, 3H); **^{13}C NMR (101 MHz, $CDCl_3$)** δ 146.15, 132.95, 128.87, 128.60, 126.99, 125.64, 124.89, 124.46, 123.65, 123.53, 123.47, 122.74, 122.04, 120.99, 120.36, 119.74, 119.59, 118.79, 112.90, 112.17, 21.55, 9.49; **HRMS(ESI)**: Calcd. for $C_{22}H_{17}OS[M-H]^-$ 329.1006; found 329.1005.

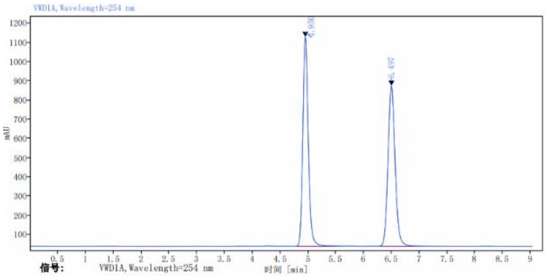

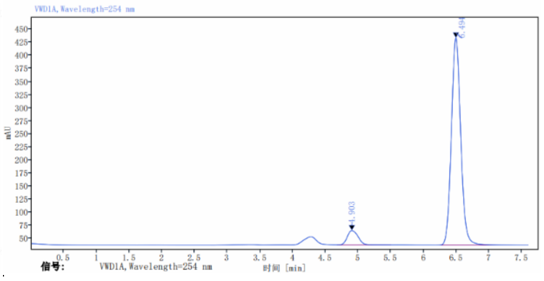

Peak	RetTime [min]	Type	Width[min]	Area[mAU*s]	Height[mAU]	Area%
	4.950	BV	0.8651	7476.5437	1091.3614	50.0761
	6.497	BB	1.5833	7453.8184	836.9032	49.9239

Peak	RetTime [min]	Type	Width[min]	Area[mAU*s]	Height[mAU]	Area%
	4.903	VB	0.8232	322.4712	27.6888	7.5347
	6.494	BBA	1.4067	3957.3510	396.0936	92.4653

(*S*)-2'-((*R*)-propylsulfinyl)-[1,1'-binaphthalen]-2-ol (5e):

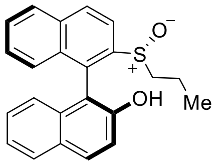

White solid (34.8 mg, 48%, -10 °C for 17.5 h); m.p. = 231-233 °C; R_f = 0.23 (petroleum ether/ethyl acetate = 1:2); the enantiomeric excess was determined to be 89% by HPLC analysis on Daicel Chirapak IA-H column (hexane/isopropanol = 90/10, flow rate 1 mL/min, T = 30 °C), UV 254 nm, t_R(major) 10.349 min, t_R(minor) 7.970 min; $[\alpha]_D^{25}$ = +287.36 (*c* = 0.44, $CHCl_3$); **^{1}H NMR (400 MHz, DMSO-*d*$_6$)** δ 10.02 (s, 1H), 8.29 (d, J = 8.7 Hz, 1H), 8.09 (dd, J = 19.6, 8.4 Hz, 2H), 7.99 (d, J = 8.9 Hz, 1H), 7.92 (d, J = 8.0 Hz, 1H), 7.64 – 7.58 (m, 1H), 7.39 (dd, J = 7.9, 4.8 Hz, 2H), 7.32 – 7.26 (m, 1H), 7.26 – 7.19 (m, 1H), 7.14 (d, J = 8.4 Hz, 1H), 6.63 (d, J = 8.3 Hz, 1H), 2.37 – 2.20 (m, 1H), 2.19 – 2.07 (m, 1H), 7.65 – 7.59 (m, 1H), 1.43 – 1.30 (m, 1H), 0.32 (t, J = 7.4 Hz, 3H); **^{13}C NMR (101 MHz, DMSO-*d*$_6$)** δ 153.07, 141.80, 134.68, 134.17, 132.59, 132.46, 131.00, 129.44, 128.99, 128.92, 128.29, 127.83, 127.67, 127.34, 126.17, 123.82, 123.28, 120.55, 118.93, 113.52, 56.64, 15.47, 12.67; **HRMS(ESI)**: Calcd. for $C_{23}H_{19}O_2S[M-H]^-$ 359.1111; found 359.1109.

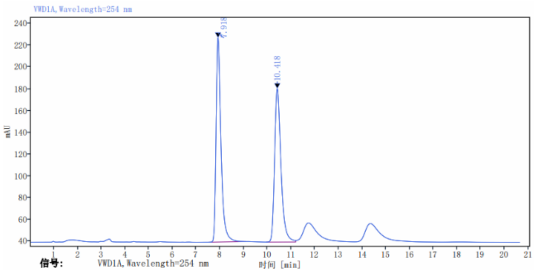

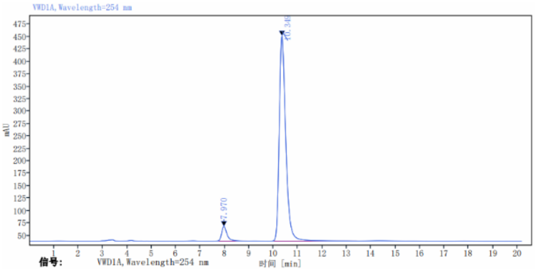

Peak	RetTime [min]	Type	Width[min]	Area[mAU*s]	Height[mAU]	Area%
	7.918	BB	1.5633	2720.0664	187.7738	50.7516
	10.418	BV	1.2512	2639.5052	141.0342	49.2484

Peak	RetTime [min]	Type	Width[min]	Area[mAU*s]	Height[mAU]	Area%
	7.970	BB	1.5067	450.9304	29.3130	5.5293
	10.349	BB	3.6100	7704.3477	412.5408	94.4707

(*R*)-2'-(propylthio)-[1,1'-binaphthalen]-2-ol (4e):

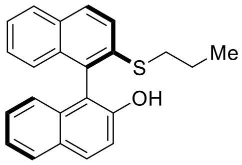

White semi-solid (34.8 mg, 50%, -10 °C for 17.5 h); R_f = 0.35 (petroleum ether/ethyl acetate = 5:1); the enantiomeric excess was determined to be

86% by HPLC analysis on Daicel Chirapak IA-H column (hexane/isopropanol = 70/30, flow rate 1 mL/min, T = 30 °C), UV 254 nm, t_R(major) 5.701 min, t_R(minor) 4.835 min; $[\alpha]_D^{25}$ = -42.50 (*c* = 0.60, $CHCl_3$); **^{1}H NMR (400 MHz, $CDCl_3$)** δ 7.99 (dd, J = 13.7, 8.9 Hz, 2H), 7.96 – 7.87 (m, 2H), 7.68 (d, J = 8.8 Hz, 1H), 7.49 – 7.43 (m, 1H), 7.42 – 7.30 (m, 3H), 7.30 – 7.24 (m, 1H), 7.18 (d, J = 8.5 Hz, 1H), 7.01 (d, J = 8.4 Hz, 1H), 4.87 (s, 1H), 3.00 – 2.78 (m, 2H), 1.73 – 1.51 (m, 2H), 0.95 (t, J = 7.3 Hz, 3H); **^{13}C NMR (101 MHz, $CDCl_3$)** δ 150.88, 137.82, 133.59, 133.36, 131.74, 130.33, 129.59, 129.18, 128.52, 128.23, 128.19, 127.46, 126.74, 125.72, 125.11, 124.62, 124.48, 123.50, 117.62, 116.97, 34.33, 22.56, 13.46; **HRMS(ESI)**: Calcd. for $C_{23}H_{19}OS[M-H]^-$ 343.1162; found 343.1160.

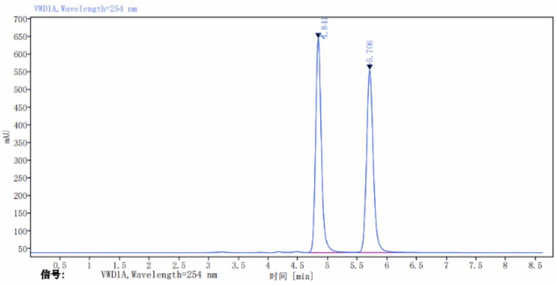

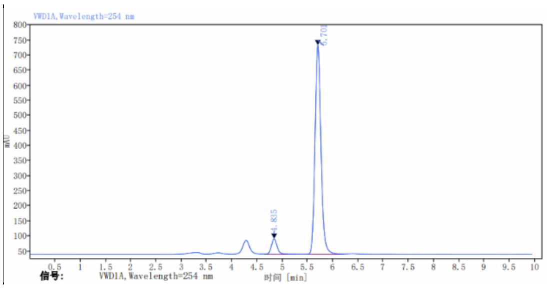

Peak	RetTime [min]	Type	Width[min]	Area[mAU*s]	Height[mAU]	Area%
	4.841	BB	0.7783	4098.2037	606.4647	50.1960
	5.706	BB	0.8733	4066.2064	516.8178	49.8040

Peak	RetTime [min]	Type	Width[min]	Area[mAU*s]	Height[mAU]	Area%
	4.835	VB	0.8137	419.3229	51.3647	6.8327
	5.701	BV	0.7509	5717.6513	693.7383	93.1673

(*S*)-2'-((*R*)-butylsulfinyl)-[1,1'-binaphthalen]-2-ol (5f):

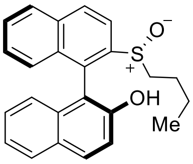

White solid (37.4 mg, 50%, -10 °C for 14 h); m.p. = 231-233 °C; R_f = 0.22 (petroleum ether/ethyl acetate = 1:2); the enantiomeric excess was determined to be 82% by HPLC analysis on Daicel Chirapak IA-H column (hexane/isopropanol = 90/10, flow rate 1 mL/min, T = 30 °C), UV 254 nm, t_R(major) 10.403 min, t_R(minor) 8.114 min; $[\alpha]_D^{25}$ = +100.69 (*c* = 0.68, $CHCl_3$); **^{1}H NMR (400 MHz, DMSO-d_6)** δ 9.77 (s, 1H), 8.07 (d, J = 8.7 Hz, 1H), 7.90 (d, J = 8.2 Hz, 1H), 7.84 (d, J = 8.7 Hz, 1H), 7.76 (d, J = 8.9 Hz, 1H), 7.69 (d, J = 7.8 Hz, 1H), 7.42 – 7.36 (m, 1H), 7.20 – 7.13 (m, 2H), 7.09 – 7.03 (m, 1H), 7.01 (d, J = 8.2 Hz, 1H), 6.90 (d, J = 8.4 Hz, 1H), 6.41 (d, J = 8.3 Hz, 1H), 2.07 – 1.92 (m, 2H), 1.14 – 1.03 (m, 1H), 0.97 – 0.86 (m, 1H), 0.69 – 0.61 (m, 1H), 0.54– 0.45 (m, 1H), 0.16 (t, J = 7.3 Hz, 3H); **^{13}C NMR (101 MHz, DMSO-d_6)** δ 153.04, 141.90, 134.69, 134.20, 132.59, 132.46, 131.00, 129.45, 128.99, 128.93, 128.32, 127.82, 127.67, 127.37, 126.18, 123.85, 123.27, 120.53, 118.95, 113.53, 54.13, 23.71, 20.91, 13.29; **HRMS(ESI)**: Calcd. for $C_{24}H_{21}O_2S[M-H]^-$ 373.1268; found 373.1265.

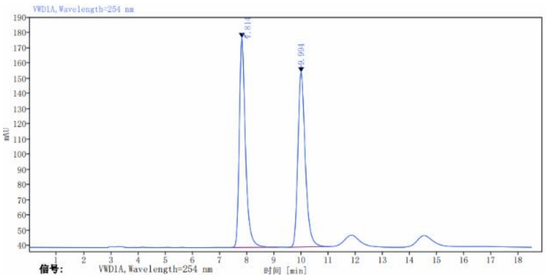

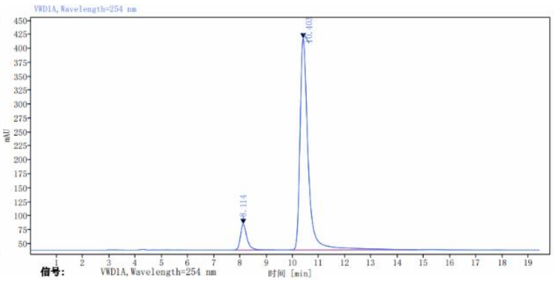

Peak	RetTime [min]	Type	Width[min]	Area[mAU^s]	Height[mAU]	Area%
	7.814	BB	1.7650	2183.4807	137.4959	49.9940
	9.994	BB	1.4967	2184.0027	114.7672	50.0060

Peak	RetTime [min]	Type	Width[min]	Area[mAU^s]	Height[mAU]	Area%
	8.114	BB	1.6606	796.6998	46.1200	8.7816
	10.403	BB	4.8883	8275.7201	379.0975	91.2184

(*R*)-2'-(butylthio)-[1,1'-binaphthalen]-2-ol (4f):

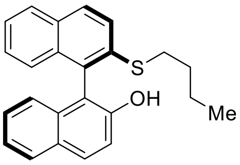

White semi-solid (33.6 mg, 47%, -10 ^{o}C for 14 h); R_f = 0.37 (petroleum ether/ethyl acetate = 5:1); the enantiomeric excess was determined to be 94% by HPLC analysis on Daicel Chirapak IA-H column (hexane/isopropanol = 70/30, flow rate 1 mL/min, T = 30 °C), UV 254 nm, t_R(major) 6.210 min, t_R(minor) 4.888 min; $[\alpha]_D^{25}$ = -36.67 (*c* = 0.66, $CHCl_3$); **^{1}H NMR (400 MHz, $CDCl_3$)** δ 7.87 (dd, J = 15.0, 8.8 Hz, 2H), 7.83 – 7.75 (m, 2H), 7.55 (d, J = 8.8 Hz, 1H), 7.37 – 7.31 (m, 1H), 7.27 (d, J = 8.9 Hz, 1H), 7.24 – 7.18 (m, 2H), 7.18 – 7.13 (m, 1H), 7.06 (d, J = 8.5 Hz, 1H), 6.88 (d, J = 8.4 Hz, 1H), 4.77 (s, 1H), 2.82 – 2.73 (m, 2H), 1.48 – 1.42 (m, 2H), 1.30 – 1.19 (m, 2H), 0.75 (t, J = 7.3 Hz, 3H); **^{13}C NMR (101 MHz, $CDCl_3$)** δ 150.91, 137.89, 133.60, 133.37, 131.75, 130.32, 129.58, 129.19, 128.55, 128.23, 128.19, 127.45, 126.72, 125.71, 125.13, 124.64, 124.50, 123.49, 117.64, 116.98, 32.08, 31.25, 21.99, 13.59; **HRMS(ESI)**: Calcd. for $C_{24}H_{21}OS[M-H]^-$ 357.1319; found 357.1316.

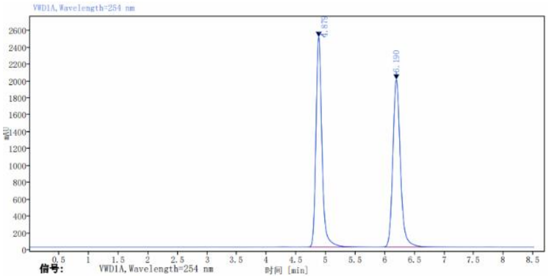

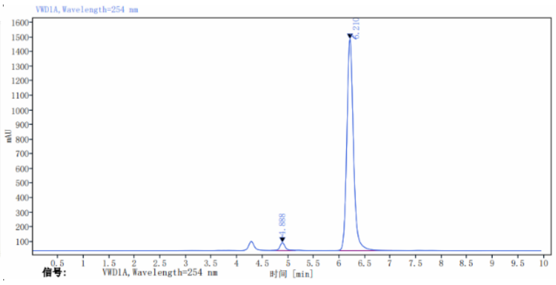

Peak	RetTime [min]	Type	Width[min]	Area[mAU^s]	Height[mAU]	Area%
	4.879	VB	1.2399	17556.4086	2483.3335	49.8790
	6.190	BB	1.1125	17641.5569	1979.5076	50.1210

Peak	RetTime [min]	Type	Width[min]	Area[mAU^s]	Height[mAU]	Area%
	4.888	BV	0.4792	384.6710	52.9405	2.8770
	6.210	BB	1.1139	12985.9979	1447.6313	97.1230

4. General procedure for the kinetic resolution of (±)-6

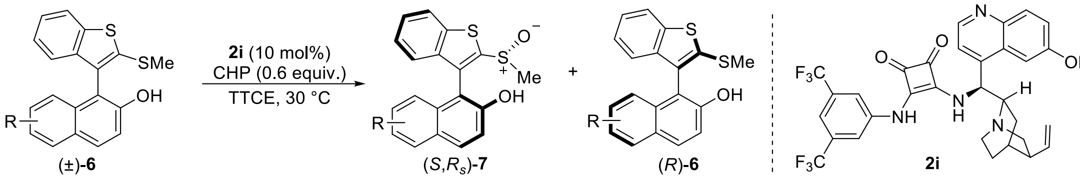

A dry Schlenk tube was charged with (±)-**6** (0.2 mmol), catalyst **2i** (12.6 mg, 0.02 mmol) and TTCE (1 mL). The mixture was stirred for 5 min at rt, then the CHP (23.0 mg, 0.12 mmol) was added and the stirring was maintained at 30 ℃ for corresponding time. The reaction was quenched with saturated Na_2SO_3 solution (2 mL) and extracted with CH_2Cl_2 (5 mL × 3). The combined organic phase was washed with saturated NH_4Cl solution (5 mL × 2), dried over Na_2SO_4 and concentrated. The residue was purified by column chromatography on silica gel eluted with petroleum ether/ethyl acetate (5:1 to 1:2) to afford the pure product.

1-((*S*)-2-((*R*)methylsulfinyl)benzo[b]thiophen-3-yl)naphthalen-2-ol (7a):

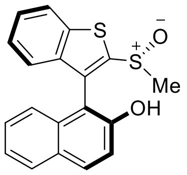

White solid (29.1 mg, 43%, 30 ^{o}C for 24 h); m.p. = 238-240 ^{o}C; R_f = 0.24 (petroleum ether/ethyl acetate = 1:2); the enantiomeric excess was determined to be 93% by HPLC analysis on Daicel Chirapak OD-H column (hexane/isopropanol = 90/10, flow rate 1 mL/min, T = 30 °C), UV 254 nm, t_R(major) 16.910 min, t_R(minor) 11.179 min; $[\alpha]_D^{25}$ = +79.36 (*c* = 0.457, $CHCl_3$); **^{1}H NMR (600 MHz, DMSO-*d$_6$*)** δ 10.08 (s, 1H), 8.20 (d, J = 8.1 Hz, 1H), 7.97 (s, 1H), 7.95 – 7.85 (m, 1H), 7.52 (d, J = 7.6 Hz, 1H), 7.38 (d, J = 8.9 Hz, 1H), 7.35 – 7.26 (m, 3H), 7.06 (d, J = 8.1 Hz, 1H), 6.98 – 6.92 (m, 1H), 2.64 (s, 3H); **^{13}C NMR (151 MHz, DMSO-*d$_6$*)** δ 153.47, 148.16, 139.91, 139.59, 134.01, 133.35, 131.11, 128.85, 128.25, 127.53, 126.74, 125.62, 124.22, 123.95, 123.70, 123.40, 118.90, 111.14, 43.88; **HRMS(ESI)**: Calcd. for $C_{19}H_{13}O_2S_2$[M-H]$^-$ 337.0362; found 337.0361.

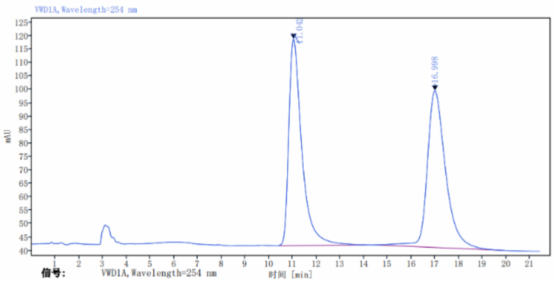

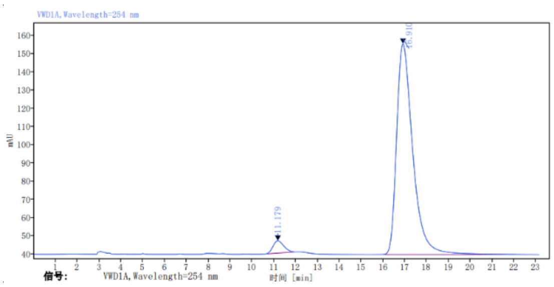

Peak	RetTime [min]	Type	Width[min]	Area[mAU^s]	Height[mAU]	Area%
	11.042	BB	3.7683	2945.2043	76.9474	48.4813
	16.998	BB	5.5900	3129.7294	58.3985	51.5187

Peak	RetTime [min]	Type	Width[min]	Area[mAU^s]	Height[mAU]	Area%
	11.179	BB	1.4317	232.6389	6.7594	3.7513
	16.910	BB	7.2433	5968.9592	115.8773	96.2487

(*R*)-1-(2-(methylthio)benzo[b]thiophen-3-yl)naphthalen-2-ol (6a):

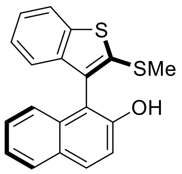

White semi-solid (36.5 mg, 57%, 30 °C for 24 h); R_f = 0.37 (petroleum ether/ethyl acetate = 5:1); the enantiomeric excess was determined to be 80% by HPLC analysis on Daicel Chirapak IA-H column (hexane/isopropanol = 80/20, flow rate 0.8 mL/min, T = 30 °C), UV 254 nm, t_R(major) 7.031 min, t_R(minor) 6.436 min; $[\alpha]_D^{25}$ **= -25.06** (*c* = 0.64, $CHCl_3$); **^{1}H NMR (600 MHz, $CDCl_3$)** δ 7.82 (d, J = 8.9 Hz, 1H), 7.77 (d, J = 7.9 Hz, 2H), 7.29 – 7.21 (m, 4H), 7.17 – 7.11 (m, 2H), 7.00 (d, J = 8.0 Hz, 1H), 5.06 (s, 1H), 2.37 (s, 3H); **^{13}C NMR (151 MHz, $CDCl_3$)** δ 151.61, 140.45, 139.96, 133.33, 130.69, 129.16, 128.30, 126.82, 125.04, 124.64, 124.57, 123.57, 122.68, 121.99, 117.60, 112.91, 19.05; **HRMS(ESI)**: Calcd. for $C_{19}H_{13}OS_2[M-H]^-$ 321.0413; found 321.0411.

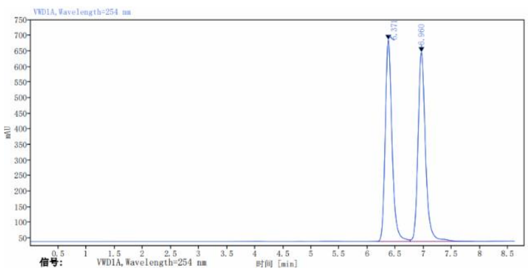

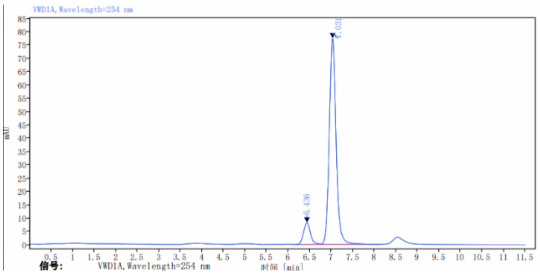

Peak	RetTime [min]	Type	Width[min]	Area[mAU*s]	Height[mAU]	Area%
	6.371	BV	0.7503	5459.3337	646.2072	49.4536
	6.960	VB	1.1425	5579.9794	606.6235	50.5464

Peak	RetTime [min]	Type	Width[min]	Area[mAU*s]	Height[mAU]	Area%
	6.436	BV	0.5977	92.2136	8.1902	9.7614
	7.031	VB	1.3939	852.4652	77.2997	90.2386

7-methoxy-1-((*S*)-2-((*R*)methylsulfinyl)benzo[b]thiophen-3-yl)naphthalen-2-ol (7b):

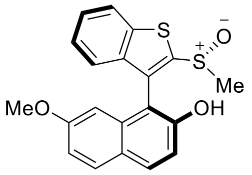

White solid (33.9 mg, 46%, 30 °C for 27 h); m.p. = 225-227 °C; R_f = 0.23 (petroleum ether/ethyl acetate = 1:2); the enantiomeric excess was determined to be 98% by HPLC analysis on Daicel Chirapak IA-H column (hexane/isopropanol = 90/10, flow rate 1 mL/min, T = 30 °C), UV 254 nm, t_R(major) 14.437 min, t_R(minor) 11.857 min; $[\alpha]_D^{25}$ **=** +22.56 (*c* = 0.7, $CHCl_3$); **^{1}H NMR (600 MHz, DMSO-*d_6*)** δ 10.04 (s, 1H), 8.20 (d, J = 8.1 Hz, 1H), 7.87 (d, J = 8.8 Hz, 1H), 7.83 (d, J = 8.9 Hz, 1H), 7.54 – 7.48 (m, 1H), 7.37 – 7.31 (m, 1H), 7.20 (d, J = 8.8 Hz, 1H), 7.09 (d, J = 8.0 Hz, 1H), 7.00 (d, J = 8.9 Hz, 1H), 6.23 (s, 1H), 3.48 (s, 3H), 2.66 (s, 3H); **^{13}C NMR (151 MHz, DMSO-*d_6*)** δ 158.62, 154.11, 148.25, 139.92, 139.34, 135.36, 133.29, 130.89, 130.64, 126.69, 125.54, 124.20, 123.97, 123.66, 116.26, 114.88, 110.34, 103.25, 55.31, 43.98; **HRMS(ESI)**: Calcd. for $C_{20}H_{15}O_3S_2[M-H]^-$ 367.0468; found 367.0465.

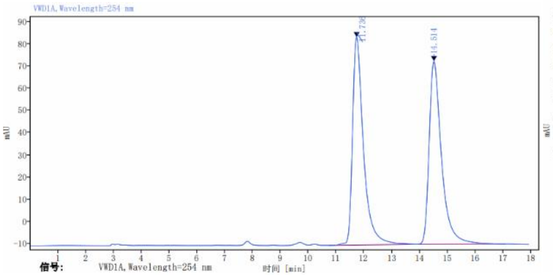

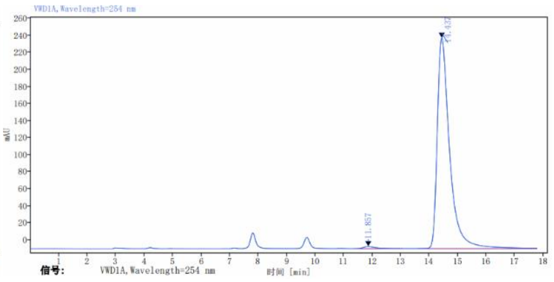

Peak	RetTime [min]	Type	Width[min]	Area[mAU^s]	Height[mAU]	Area%
	11.736	BB	3.1567	2566.7443	93.6352	50.1252
	14.514	BB	3.1900	2553.9258	82.4352	49.8748

Peak	RetTime [min]	Type	Width[min]	Area[mAU^s]	Height[mAU]	Area%
	11.857	BB	1.7283	71.6572	2.3699	0.9484
	14.437	BBA	4.1900	7484.1811	247.3323	99.0516

(*R*)-7-methoxy-1-(2-(methylthio)benzo[b]thiophen-3-yl)naphthalen-2-ol (6b):

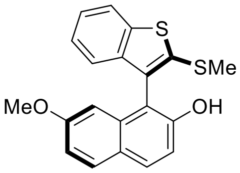

White semi-solid (34.5 mg, 49%, 30 °C for 27 h); R_f = 0.36 (petroleum ether/ethyl acetate = 5:1); the enantiomeric excess was determined to be 90% by HPLC analysis on Daicel Chirapak IA-H column (hexane/isopropanol = 70/30, flow rate 1 mL/min, T = 30 °C), UV 254 nm, t_R(major) 5.083 min, t_R(minor) 4.617 min; $[\alpha]_D^{25}$ = -11.97 (*c* = 0.66, $CHCl_3$); **^{1}H NMR (600 MHz, $CDCl_3$)** δ 7.89 (d, J = 7.9 Hz, 1H), 7.85 (d, J = 8.7 Hz, 1H), 7.79 (d, J = 8.8 Hz, 1H), 7.40 – 7.34 (m, 1H), 7.30 – 7.26 (m, 1H), 7.21 (d, J = 8.8 Hz, 1H), 7.17 (d, J = 7.9 Hz, 1H), 7.05 (d, J = 8.8 Hz, 1H), 6.56 (s, 1H), 5.15 (s, 1H), 3.64 (s, 3H), 2.51 (s, 3H); **^{13}C NMR (151 MHz, $CDCl_3$)** δ 158.57, 152.20, 140.38, 139.96, 139.64, 134.72, 130.42, 129.87, 128.36, 125.01, 124.64, 124.52, 122.73, 122.00, 115.47, 115.03, 112.10, 103.94, 55.17, 19.08; **HRMS(ESI)**: Calcd. for $C_{20}H_{15}O_2S_2$[M-H]$^-$ 351.0519; found 351.0516.

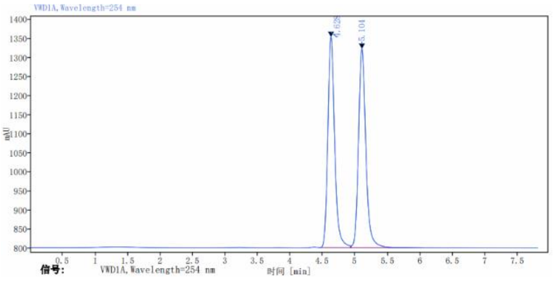

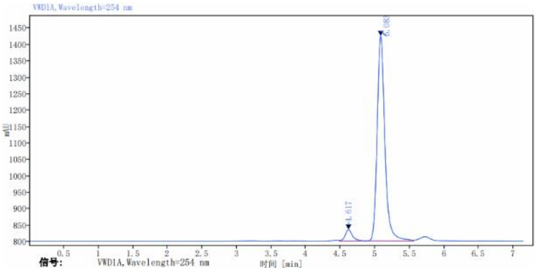

Peak	RetTime [min]	Type	Width[min]	Area[mAU^s]	Height[mAU]	Area%
	4.628	BV	0.4796	4039.4235	551.6821	49.4476
	5.104	VB	1.1255	4129.6777	520.9250	50.5524

Peak	RetTime [min]	Type	Width[min]	Area[mAU^s]	Height[mAU]	Area%
	4.617	VB	0.3936	257.1823	34.6525	5.0336
	5.083	BV	0.6771	4852.1080	624.0539	94.9664

6-methoxy-1-((*S*)-2-((*R*)methylsulfinyl)benzo[b]thiophen-3-yl)naphthalen-2-ol (7c):

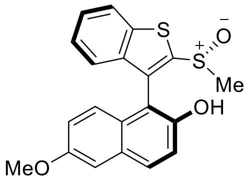

White semi-solid (35.3 mg, 48%, 30 °C for 24 h); R_f = 0.23 (petroleum ether/ethyl acetate = 1:2); the enantiomeric excess was determined to be 88% by HPLC analysis on Daicel Chirapak IA-H column

(hexane/isopropanol = 90/10, flow rate 1 mL/min, T = 30 °C), UV 254 nm, t_R(major) 19.168 min, t_R(minor) 16.791 min; $[\alpha]_D^{25}$ = +8.97 (c = 0.680, $CHCl_3$); **^{1}H NMR (600 MHz, $CDCl_3$)** δ 7.99 (d, J = 8.2 Hz, 1H), 7.80 (d, J = 8.9 Hz, 1H), 7.51 – 7.45 (m, 1H), 7.38 – 7.32 (m, 2H), 7.32 – 7.24 (m, 2H), 7.22 – 7.18 (m, 2H), 6.98 – 6.93 (m, 1H), 3.91 (s, 3H), 2.81 (s, 3H); **^{13}C NMR (151 MHz, $CDCl_3$)** δ 156.21, 150.58, 147.19, 145.07, 140.59, 139.37, 133.96, 130.12, 129.96, 128.73, 126.99, 125.47, 124.80, 123.15, 119.61, 119.54, 112.18, 107.04, 55.41, 42.49; **HRMS(ESI)**: Calcd. for $C_{20}H_{15}O_3S_2$[M-H]$^-$ 367.0468; found 367.0468.

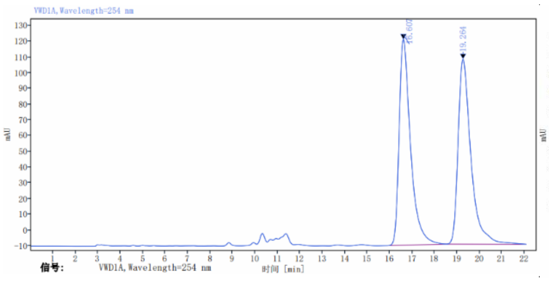

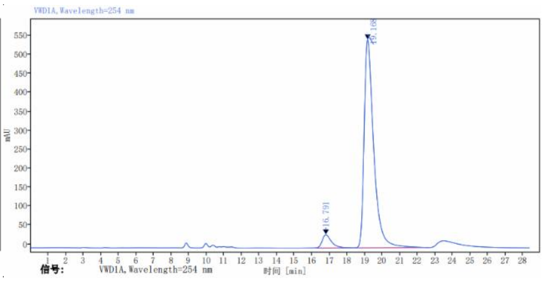

Peak	RetTime [min]	Type	Width[min]	Area[mAU^s]	Height[mAU]	Area%
	16.607	BB	2.6537	4631.3804	130.7220	49.7591
	19.264	BBA	3.5600	4676.2194	117.4877	50.2409

Peak	RetTime [min]	Type	Width[min]	Area[mAU^s]	Height[mAU]	Area%
	16.791	BB	3.2550	1285.2239	34.8858	5.8194
	19.168	BB	4.2423	20799.8720	548.9067	94.1806

(*R*)-6-methoxy-1-(2-(methylthio)benzo[b]thiophen-3-yl)naphthalen-2-ol (6c):

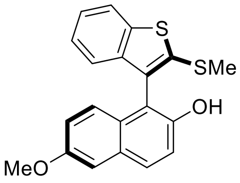

White semi-solid (33.7 mg, 48%, 30 °C for 24 h); R_f = 0.35 (petroleum ether/ethyl acetate = 5:1); the enantiomeric excess was determined to be 88% by HPLC analysis on Daicel Chirapak IA-H column (hexane/isopropanol = 70/30, flow rate 1 mL/min, T = 30 °C), UV 254 nm, t_R(major) 5.711 min, t_R(minor) 5.404 min; $[\alpha]_D^{25}$ = -26.09 (c = 0.640, $CHCl_3$); **^{1}H NMR (600 MHz, $CDCl_3$)** δ 7.89 – 7.83 (m, 2H), 7.40 – 7.32 (m, 2H), 7.30 – 7.24 (m, 1H), 7.22 (s, 1H), 7.14 (dd, J = 18.6, 8.5 Hz, 2H), 7.03 (d, J = 9.1 Hz, 1H), 5.04 (s, 1H), 3.93 (s, 3H), 2.49 (s, 3H); **^{13}C NMR (151 MHz, $CDCl_3$)** δ 156.07, 150.01, 140.27, 139.96, 139.93, 130.06, 129.33, 128.51, 128.45, 126.13, 125.04, 124.63, 122.66, 121.99, 119.25, 118.03, 113.22, 106.77, 55.35, 19.03; **HRMS(ESI)**: Calcd. for $C_{20}H_{15}O_2S_2$[M-H]$^-$ 351.0519; found 351.0514.

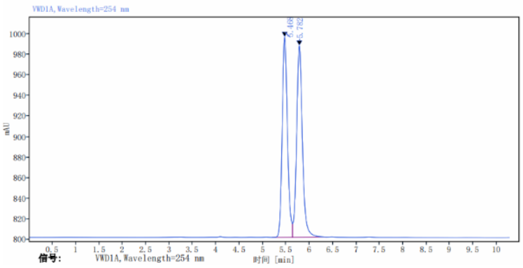

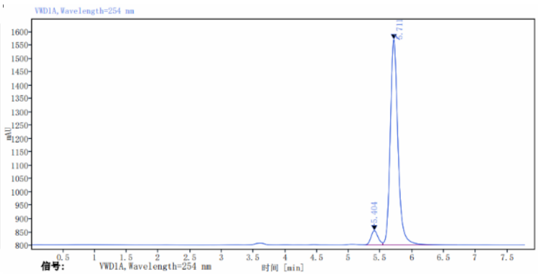

Peak	RetTime [min]	Type	Width[min]	Area[mAU*s]	Height[mAU]	Area%
	5.468	BV	0.4405	1528.8600	194.7959	48.6172
	5.782	VB	0.7474	1615.8312	186.3043	51.3828

Peak	RetTime [min]	Type	Width[min]	Area[mAU*s]	Height[mAU]	Area%
	5.404	VV	0.3010	425.7884	53.9931	6.1125
	5.711	VB	1.1708	6540.0722	769.9294	93.8875

8-bromo-1-((*S*)-2-((*R*)methylsulfinyl)benzo[b]thiophen-3-yl)naphthalen-2-ol (7d):

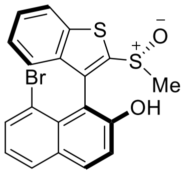

White solid (35.3 mg, 42%, 30 °C for 36 h); m.p. = 223-225 °C; R_f = 0.24 (petroleum ether/ethyl acetate = 1:2); the enantiomeric excess was determined to be 88% by HPLC analysis on Daicel Chirapak IA-H column (hexane/isopropanol = 90/10, flow rate 1 mL/min, T = 30 °C), UV 254 nm, t_R(major) 18.778 min, t_R(minor) 13.066 min; $[\alpha]_D^{25}$ = -19.30 (c = 0.475, $CHCl_3$); **^{1}H NMR (600 MHz, $CDCl_3$)** δ 7.80 (d, J = 8.0 Hz, 1H), 7.80 – 7.71 (m, 2H), 7.57 (d, J = 7.2 Hz, 1H), 7.37 (d, J = 8.8 Hz, 1H), 7.36 – 7.30 (m, 1H), 7.16 (d, J = 7.4 Hz, 1H), 7.09 – 7.03 (m, 1H), 7.02 (d, J = 7.9 Hz, 1H), 2.90 (s, 3H); **^{13}C NMR (151 MHz, $CDCl_3$)** δ 154.81, 143.42, 141.00, 139.61, 136.65, 134.30, 132.02, 131.63, 130.79, 129.38, 126.77, 125.21, 124.44, 123.64, 123.03, 119.31, 117.11, 110.65, 41.10; **HRMS(ESI)**: Calcd. for $C_{19}H_{12}BrO_2S_2$[M-H]$^-$ 414.9468; found 414.9464.

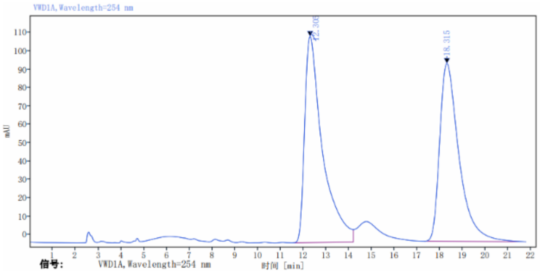

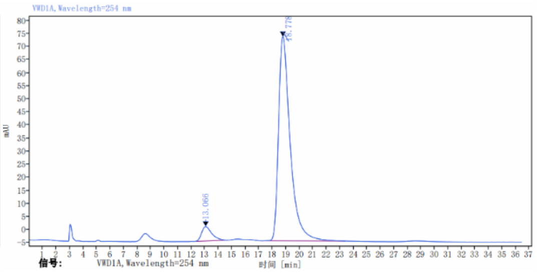

Peak	RetTime [min]	Type	Width[min]	Area[mAU*s]	Height[mAU]	Area%
	12.305	BV	2.6641	6125.1723	112.2220	51.7755
	18.315	BBA	4.4800	5705.0894	96.7382	48.2245

Peak	RetTime [min]	Type	Width[min]	Area[mAU*s]	Height[mAU]	Area%
	13.066	BB	2.4650	289.7280	5.4877	5.8124
	18.778	BB	6.2100	4694.9131	78.0038	94.1876

(*R*)-8-bromo-1-(2-(methylthio)benzo[b]thiophen-3-yl)naphthalen-2-ol (6d):

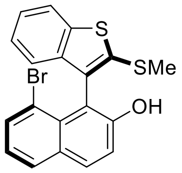

White semi-solid (40.9 mg, 51%, 30 °C for 36 h); R_f = 0.34 (petroleum ether/ethyl acetate = 5:1); the enantiomeric excess was determined to be 84% by HPLC analysis on Daicel Chirapak IA-H column (hexane/isopropanol = 80/20, flow rate 0.8 mL/min, T = 30 °C), UV 254 nm, t_R(major) 7.167 min, t_R(minor) 6.617 min; $[\alpha]_D^{25}$ = +55.58 (*c* = 0.8, $CHCl_3$); **^{1}H NMR (600 MHz, $CDCl_3$)** δ 7.94 (d, J = 8.8 Hz, 1H), 7.90 – 7.84 (m, 2H), 7.74 (d, J = 7.3 Hz, 1H), 7.41 (d, J = 8.8 Hz, 1H), 7.40 – 7.34 (m, 1H), 7.29 (d, J = 7.1 Hz, 1H), 7.22 – 7.16 (m, 1H), 7.09 (d, J = 7.9 Hz, 1H), 5.42 (s, 1H), 2.55 (s, 3H); **^{13}C NMR (151 MHz, $CDCl_3$)** δ 153.39, 141.52, 140.65, 139.40, 134.34, 131.85, 131.35, 131.16, 129.25, 128.96, 125.14, 124.61, 123.96, 122.41, 121.98, 118.07, 117.69, 112.18, 18.44; **HRMS(ESI)**:

Calcd. for $C_{19}H_{12}BrOS_2[M-H]^-$ 398.9518; found 398.9515.

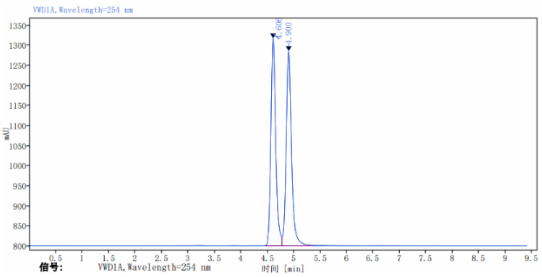
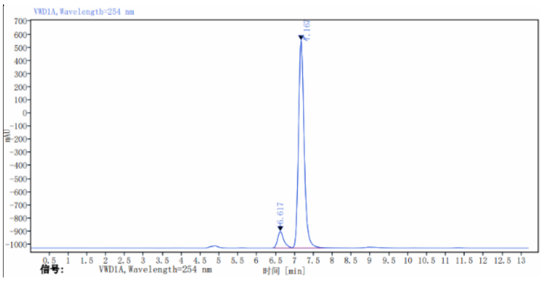

Peak	RetTime [min]	Type	Width[min]	Area[mAU*s]	Height[mAU]	Area%
	4.606	BV	0.6679	3317.3975	515.6058	49.1534
	4.900	VB	0.8426	3431.6731	483.6373	50.8466

Peak	RetTime [min]	Type	Width[min]	Area[mAU*s]	Height[mAU]	Area%
	6.617	BV	0.6058	1448.4674	126.2063	8.0198
	7.167	VB	1.5642	16612.5721	1581.5740	91.9802

7-bromo-1-((*S*)-2-((*R*)methylsulfinyl)benzo[b]thiophen-3-yl)naphthalen-2-ol (7e):

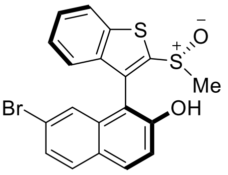

White solid (39.2 mg, 47%, 30 °C for 23 h); m.p. = 248-250 °C; R_f = 0.28 (petroleum ether/ethyl acetate = 1:2); the enantiomeric excess was determined to be 88% by HPLC analysis on Daicel Chirapak IA-H column (hexane/isopropanol = 90/10, flow rate 1 mL/min, T = 30 °C), UV 254 nm, t_R(major) 10.189 min, t_R(minor) 8.812 min; $[\alpha]_D^{25}$ = +24.65 (c = 0.66, $CHCl_3$); **^{1}H NMR (400 MHz, $CDCl_3$)** δ 8.46 (s, 1H), 7.82 (d, J = 8.1 Hz, 1H), 7.71 (d, J = 8.9 Hz, 1H), 7.61 (d, J = 8.7 Hz, 1H), 7.37 – 7.28 (m, 3H), 7.22 – 7.15 (m, 1H), 7.14 – 7.05 (m, 2H), 2.81 (s, 3H); **^{13}C NMR (101 MHz, $CDCl_3$)** δ 153.87, 145.10, 140.69, 138.60, 135.08, 134.91, 130.86, 130.11, 127.09, 126.95, 126.59, 125.83, 125.38, 124.87, 123.16, 121.41, 119.47, 110.75, 42.65; **HRMS(ESI)**: Calcd. for $C_{19}H_{12}BrO_2S_2[M-H]^-$ 414.9468; found 414.9464.

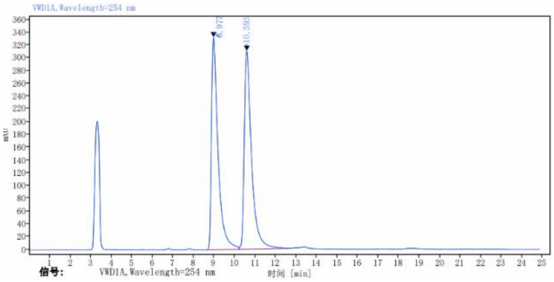
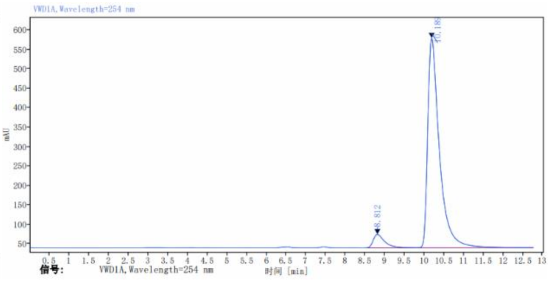

Peak	RetTime [min]	Type	Width[min]	Area[mAU*s]	Height[mAU]	Area%
	8.977	BV	1.6170	7453.1572	332.6590	49.2962
	10.593	VB	2.4235	7665.9685	310.3453	50.7038

Peak	RetTime [min]	Type	Width[min]	Area[mAU*s]	Height[mAU]	Area%
	8.812	BV	1.3650	735.3899	34.9814	6.1768
	10.189	VBA	2.9483	11170.2331	539.4040	93.8232

(*R*)-7-bromo-1-(2-(methylthio)benzo[b]thiophen-3-yl)naphthalen-2-ol (6e):

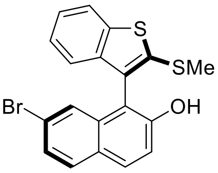

White semi-solid (39.9 mg, 50%, 30 °C for 23 h); R_f = 0.34 (petroleum ether/ethyl acetate = 5:1); the enantiomeric excess was determined to be 84% by HPLC analysis on Daicel Chirapak IA-H column (hexane/isopropanol =

80/20, flow rate 0.8 mL/min, T = 30 °C), UV 254 nm, t_R(major) 6.538 min, t_R(minor) 5.916 min; $[\alpha]_D^{25}$ = +6.99 (*c* = 0.62, $CHCl_3$); **^{1}H NMR (400 MHz, $CDCl_3$)** δ 8.04 (s, 1H), 7.86 (dd, J = 22.1, 8.5 Hz, 2H), 7.37 (d, J = 8.9 Hz, 3H), 7.28 (d, J = 5.9 Hz, 1H), 7.10 (dd, J = 12.4, 8.6 Hz, 2H), 5.20 (s, 1H), 2.50 (s, 3H); **^{13}C NMR (101 MHz, $CDCl_3$)** δ 151.95, 140.88, 139.92, 139.75, 131.84, 130.30, 130.25, 130.06, 129.72, 127.35, 126.42, 125.17, 124.74, 122.43, 122.07, 118.80, 117.37, 113.19, 18.99; **HRMS(ESI)**: Calcd. for $C_{19}H_{12}BrOS_2$[M-H]$^-$ 398.9518; found 398.9514.

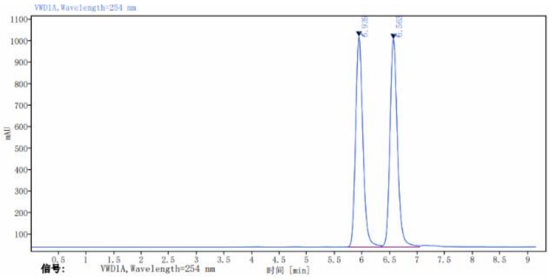

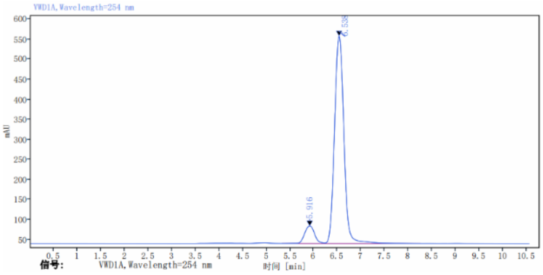

Peak	RetTime [min]	Type	Width[min]	Area[mAU*s]	Height[mAU]	Area%
	5.939	BV	0.6519	9038.8863	979.8976	49.7507
	6.563	VV	0.6837	9129.4886	968.4144	50.2493

Peak	RetTime [min]	Type	Width[min]	Area[mAU*s]	Height[mAU]	Area%
	5.916	BV	0.9062	631.4323	43.2589	8.1424
	6.538	VB	2.0271	7123.4309	517.4369	91.8576

6-bromo-1-((*S*)-2-((*R*)methylsulfinyl)benzo[b]thiophen-3-yl)naphthalen-2-ol (7f):

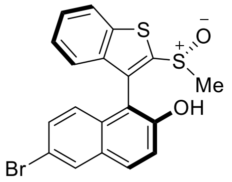

White solid (38.4 mg, 46%, 30 °C for 23 h); m.p. = 194-196 °C; R_f = 0.26 (petroleum ether/ethyl acetate = 1:2); the enantiomeric excess was determined to be 87% by HPLC analysis on Daicel Chirapak IA-H column (hexane/isopropanol = 90/10, flow rate 1 mL/min, T = 30 °C), UV 254 nm, t_R(major) 12.727 min, t_R(minor) 10.710 min; $[\alpha]_D^{25}$ = +79.83 (c = 0.60, $CHCl_3$); **^{1}H NMR (400 MHz, $CDCl_3$)** δ 7.98 – 7.90 (m, 2H), 7.73 (d, J = 8.9 Hz, 1H), 7.43 (t, J = 7.9 Hz, 2H), 7.30 (dd, J = 8.9, 2.1 Hz, 1H), 7.26 – 7.22 (m, 1H), 7.15 (d, J = 8.1 Hz, 1H), 6.92 (d, J = 9.0 Hz, 1H), 2.82 (s, 3H), 2.04 (s, 1H); **^{13}C NMR (101 MHz, $CDCl_3$)** δ 153.49, 145.37, 140.45, 139.13, 134.26, 132.53, 130.05, 129.89, 129.87, 129.58, 126.49, 125.28, 124.97, 123.94, 122.92, 119.10, 116.34, 111.18, 42.12; **HRMS(ESI)**: Calcd. for $C_{19}H_{12}BrO_2S_2$[M-H]$^-$ 414.9468; found 414.9464.

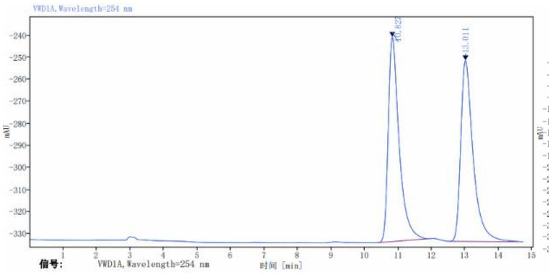

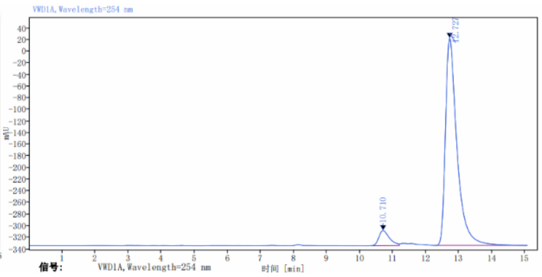

Peak	RetTime [min]	Type	Width[min]	Area[mAU^s]	Height[mAU]	Area%
	10.827	BB	1.5075	2093.6731	92.7147	49.7914
	13.011	BBA	2.1900	2111.2166	82.0594	50.2086

Peak	RetTime [min]	Type	Width[min]	Area[mAU^s]	Height[mAU]	Area%
	10.710	BV	0.9682	583.7768	26.1629	6.3084
	12.727	BBA	2.8000	8670.1692	356.2451	93.6916

(*R*)-6-bromo-1-(2-(methylthio)benzo[b]thiophen-3-yl)naphthalen-2-ol (6f):

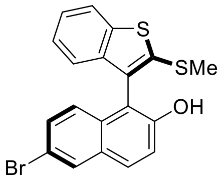

White semi-solid solid (39.9 mg, 50%, 30 °C for 23 h); R_f = 0.36 (petroleum ether/ethyl acetate = 5:1); the enantiomeric excess was determined to be 85% by HPLC analysis on Daicel Chirapak IA-H column (hexane/isopropanol = 90/10, flow rate 0.8 mL/min, T = 30 °C), UV 254 nm, t_R(major) 10.295 min, t_R(minor) 9.296 min; $[\alpha]_D^{25}$ = -27.68 (*c* = 0.66, $CHCl_3$); **^{1}H NMR (400 MHz, $CDCl_3$)** δ 7.89 (dd, J = 8.4, 5.2 Hz, 2H), 7.74 (d, J = 8.6 Hz, 1H), 7.45 (dd, J = 8.6, 1.8 Hz, 1H), 7.42 – 7.32 (m, 3H), 7.32 – 7.26 (m, 1H), 7.11 (d, J = 8.0 Hz, 1H), 5.21 (s, 1H), 2.53 (s, 3H); **^{13}C NMR (101 MHz, $CDCl_3$)** δ 152.50, 141.16, 139.99, 139.59, 134.58, 130.61, 129.95, 127.60, 127.04, 126.61, 125.24, 124.77, 122.36, 122.11, 121.67 – 120.89 (m), 118.07, 112.34, 19.02; **HRMS(ESI)**: Calcd. for $C_{19}H_{12}BrOS_2[M-H]^-$ 398.9518; found 398.9514.

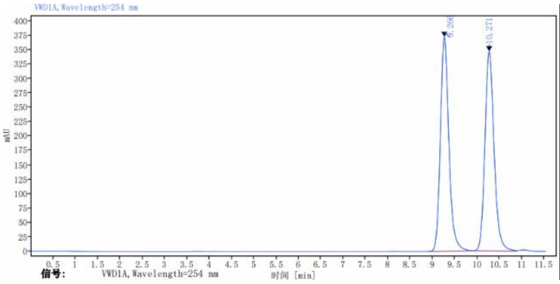

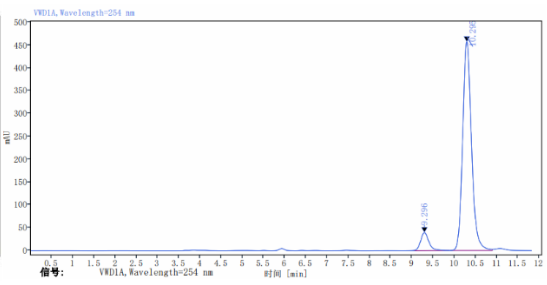

Peak	RetTime [min]	Type	Width[min]	Area[mAU^s]	Height[mAU]	Area%
	9.266	BB	1.0294	4941.1502	371.9536	50.0273
	10.271	BV	0.9774	4935.7546	346.2066	49.9727

Peak	RetTime [min]	Type	Width[min]	Area[mAU^s]	Height[mAU]	Area%
	9.296	BB	1.0205	536.3659	39.3730	7.4946
	10.295	BV	0.9953	6620.3652	456.6699	92.5054

6-hydroxy-5-((*S*)-2-((*R*)methylsulfinyl)benzo[b]thiophen-3-yl)-2-naphthaldehyde (7g):

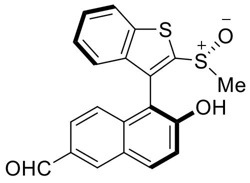

White solid (28.5 mg, 39%, 30 °C for 24 h); m.p. = 162-164 °C; R_f = 0.19 (petroleum ether/ethyl acetate = 1:2); the enantiomeric excess was determined to be 93% by HPLC analysis on Daicel Chirapak IA-H column (hexane/isopropanol = 90/10, flow rate 1 mL/min, T = 30 °C), UV 254 nm, t_R(major) 23.912 min, t_R(minor) 20.725 min; $[\alpha]_D^{25}$ = +94.39 (c = 0.60, $CHCl_3$); **^{1}H NMR (600 MHz, DMSO-d_6)** δ 10.71 (s, 1H), 10.07 (s, 1H), 8.57 (s, 1H), 8.23 (d, J = 8.6 Hz, 2H), 7.72 (d, J = 8.7 Hz, 1H), 7.52 (dd, J = 19.9, 8.2 Hz, 2H), 7.38 – 7.32 (m, 1H), 7.09 (dd, J = 15.2, 8.4 Hz, 2H), 2.68 (s, 3H); **^{13}C NMR (151 MHz, DMSO-d_6)** δ 192.79, 156.61, 148.47, 139.97, 139.33, 137.39, 135.54, 133.21, 132.67, 131.84, 127.32, 126.92, 125.78, 124.78, 124.29, 124.11, 124.04, 120.00,

111.99, 43.90; **HRMS(ESI)**: Calcd. for $C_{20}H_{13}O_3S_2$[M-H]$^-$ 365.0312; found 365.0309.

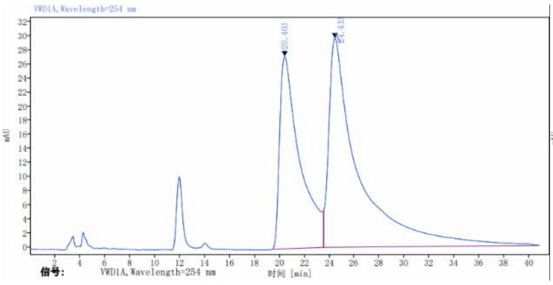

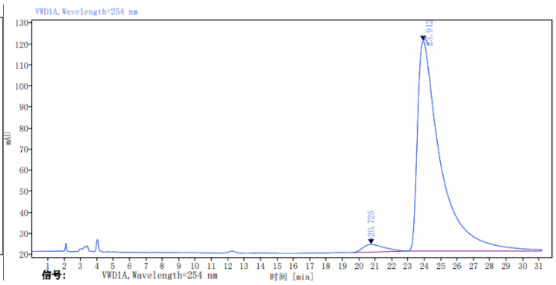

Peak	RetTime [min]	Type	Width[min]	Area[mAU^s]	Height[mAU]	Area%
	20.403	BM m	1.5458	3051.8885	27.3306	38.0627
	24.433	MM m	2.1992	4966.1624	29.6933	61.9373

Peak	RetTime [min]	Type	Width[min]	Area[mAU^s]	Height[mAU]	Area%
	20.725	BB	3.3967	327.5981	3.5537	3.2639
	23.912	BBA	8.2467	9709.4152	99.6077	96.7361

(*R*)-6-hydroxy-5-(2-(methylthio)benzo[b]thiophen-3-yl)-2-naphthaldehyde (6g):

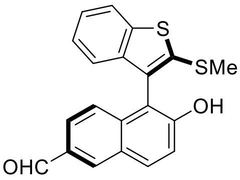

White solid (39.9 mg, 57%, 30 °C for 24 h); m.p. = 181-183 °C; R_f = 0.36 (petroleum ether/ethyl acetate = 5:1); the enantiomeric excess was determined to be 74% by HPLC analysis on Daicel Chirapak IA-H column (hexane/isopropanol = 90/10, flow rate 1 mL/min, T = 30 °C), UV 254 nm, t_R(major) 13.153 min, t_R(minor) 10.254 min; $[\alpha]_D^{25}$ = +29.66 (*c* = 0.68, $CHCl_3$); **^{1}H NMR (600 MHz, $CDCl_3$)** δ 9.98 (s, 1H), 8.26 (s, 1H), 7.97 (d, J = 8.8 Hz, 1H), 7.78 (d, J = 8.1 Hz, 1H), 7.68 (d, J = 8.7 Hz, 1H), 7.35 (d, J = 8.8 Hz, 1H), 7.29 – 7.23 (m, 1H), 7.22 (d, J = 8.7 Hz, 1H), 7.18 – 7.13 (m, 1H), 6.97 (d, J = 8.0 Hz, 1H), 5.55 (s, 1H), 2.39 (s, 3H); **^{13}C NMR (151 MHz, $CDCl_3$)** δ 191.97, 154.63, 141.23, 139.97, 139.65, 136.94, 134.74, 132.39, 132.20, 128.24, 126.99, 125.63, 125.24, 124.80, 124.12, 122.30, 122.14, 118.97, 113.74, 19.02; **HRMS(ESI)**: Calcd. for $C_{20}H_{13}O_2S_2$[M-H]$^-$ 349.0362; found 349.0359.

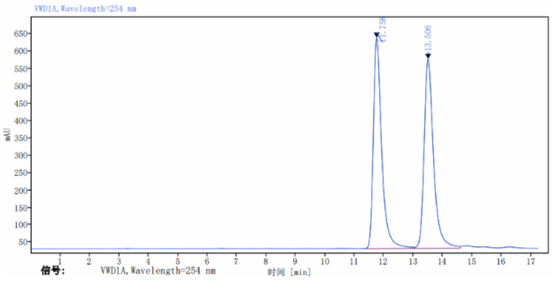

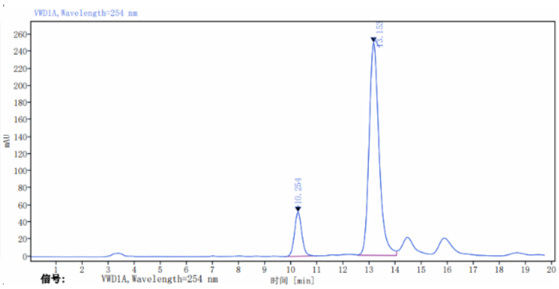

Peak	RetTime [min]	Type	Width[min]	Area[mAU^s]	Height[mAU]	Area%
	11.758	BV	1.7781	11994.9710	607.5549	49.7221
	13.506	VV	1.5856	12129.0604	545.3739	50.2779

Peak	RetTime [min]	Type	Width[min]	Area[mAU^s]	Height[mAU]	Area%
	10.254	BB	1.2728	1003.1921	51.4743	13.1902
	13.153	VV	1.5061	6602.3739	249.1230	86.8098

Methyl 6-hydroxy-5-((*S*)-2-((*R*)methylsulfinyl)benzo[b]thiophen-3-yl)-2-naphthoate (7h):

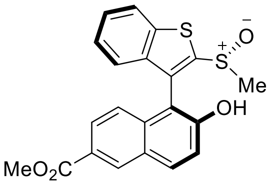

White solid (33.3 mg, 42%, 30 °C for 25.5 h); m.p. = 152-154 °C; R_f = 0.20 (petroleum ether/ethyl acetate = 1:2); the enantiomeric excess was determined to be 88% by HPLC analysis on Daicel Chirapak IA-H column (hexane/isopropanol = 90/10, flow rate 1 mL/min, T = 30 °C), UV 254 nm, t_R(major) 17.376 min, t_R(minor) 15.617 min; $[\alpha]_D^{25}$ = +85.83 (c = 0.76, $CHCl_3$); **^{1}H NMR (600 MHz, $CDCl_3$)** δ 8.49 (s, 1H), 7.88 – 7.80 (m, 2H), 7.74 (d, J = 8.9 Hz, 1H), 7.39 (d, J = 9.0 Hz, 2H), 7.19 – 7.12 (m, 1H), 7.07 (d, J = 8.2 Hz, 1H), 6.99 (d, J = 8.8 Hz, 1H), 3.86 (s, 3H), 2.75 (s, 3H); **^{13}C NMR (151 MHz, $CDCl_3$)** δ 167.21, 155.16, 145.21, 140.68, 138.76, 136.18, 134.82, 132.42, 131.58, 127.51, 127.02, 126.33, 125.31, 124.95, 124.82, 124.01, 123.16, 119.85, 111.53, 52.15, 42.78; **HRMS(ESI)**: Calcd. for $C_{21}H_{15}O_4S_2[M-H]^-$ 395.0417; found 395.0413.

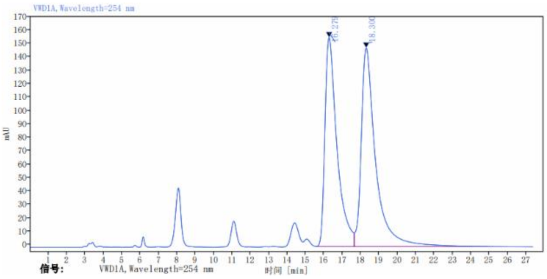

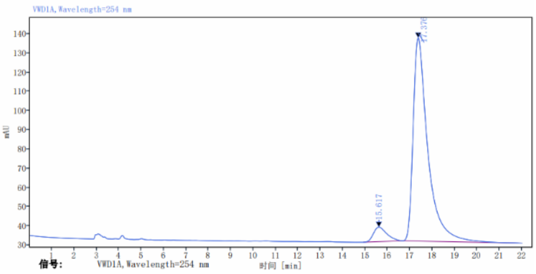

Peak	RetTime [min]	Type	Width[min]	Area[mAU*s]	Height[mAU]	Area%
	16.279	BV	2.0683	7073.7474	155.8396	46.6918
	18.300	VB	7.3206	8076.1082	148.0623	53.3082

Peak	RetTime [min]	Type	Width[min]	Area[mAU*s]	Height[mAU]	Area%
	15.617	BB	1.7513	314.4566	7.5816	6.1197
	17.376	BBA	5.3467	4823.9418	105.7289	93.8803

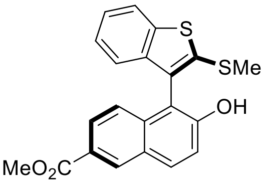

(*R*)-Methyl 6-hydroxy-5-(2-(methylthio)benzo[b]thiophen-3-yl)-2-naphthoate (6h):

White solid (41.8 mg, 55%, 30 °C for 25.5 h); m.p. = 179-181 °C; R_f = 0.32 (petroleum ether/ethyl acetate = 5:1); the enantiomeric excess was determined to be 82% by HPLC analysis on Daicel Chirapak IA-H column (hexane/isopropanol = 70/30, flow rate 1 mL/min, T = 30 °C), UV 254 nm, t_R(major) 5.542 min, t_R(minor) 4.937 min; $[\alpha]_D^{25}$ = -8.81 (*c* = 0.84, $CHCl_3$); **^{1}H NMR (600 MHz, $CDCl_3$)** δ 8.54 (s, 1H), 7.94 (d, J = 8.6 Hz, 1H), 7.81 (s, 2H), 7.32 (d, J = 8.6 Hz, 1H), 7.31 – 7.25 (m, 1H), 7.18 (d, J = 4.7 Hz, 2H), 6.99 (d, J = 7.7 Hz, 1H), 5.29 (s, 1H), 3.87 (s, 3H), 2.40 (s, 3H); **^{13}C NMR (151 MHz, $CDCl_3$)** δ 167.30, 153.73, 140.99, 139.97, 139.70, 135.81, 132.17, 131.48, 128.19, 127.33, 126.33, 125.26, 125.18, 124.76, 122.43, 122.09, 118.54, 113.19, 52.13, 19.03; **HRMS(ESI)**: Calcd. for $C_{21}H_{15}O_3S_2[M-H]^-$ 379.0468; found 379.0465.

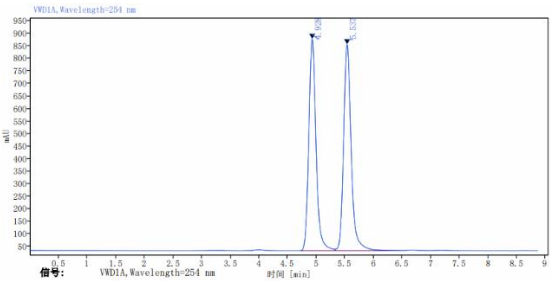

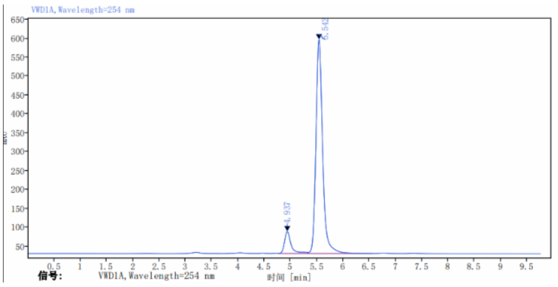

Peak	RetTime [min]	Type	Width[min]	Area[mAU^s]	Height[mAU]	Area%
	4.928	BV	0.7237	7460.1398	845.7597	49.4132
	5.537	VV	1.1359	7637.3093	824.1802	50.5868

Peak	RetTime [min]	Type	Width[min]	Area[mAU^s]	Height[mAU]	Area%
	4.937	VV	0.5551	507.1951	58.0516	9.1619
	5.542	VB	1.2360	5028.7294	565.9984	90.8381

6-hydroxy-5-((*S*)-2-((*R*)methylsulfinyl)benzo[b]thiophen-3-yl)-2-naphthonitrile (7i):

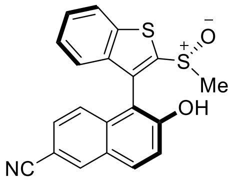

White solid (33.4 mg, 46%, 30 ^{o}C for 26 h); m.p. = 159-161 ^{o}C; R_f = 0.20 (petroleum ether/ethyl acetate = 1:2); the enantiomeric excess was determined to be 83% by HPLC analysis on Daicel Chirapak IA-H column (hexane/isopropanol = 90/10, flow rate 1 mL/min, T = 30 °C), UV 254 nm, t_R(major) 23.470 min, t_R(minor) 19.016 min; $[\alpha]_D^{25}$ = +87.02 (c = 0.56, $CHCl_3$); **^{1}H NMR (600 MHz, DMSO-*d$_6$*)** δ 8.56 (s, 1H), 8.22 (d, J = 8.2 Hz, 1H), 8.13 (d, J = 8.9 Hz, 1H), 7.57 (d, J = 8.8 Hz, 1H), 7.52 (d, J = 8.4 Hz, 3H), 7.38 – 7.32 (m, 1H), 7.10 (d, J = 8.7 Hz, 1H), 7.06 (d, J = 8.0 Hz, 1H), 2.68 (s, 3H); **^{13}C NMR (151 MHz, DMSO-*d$_6$*)** δ 156.52, 148.66, 139.97, 139.25, 135.83, 135.22, 132.26, 132.12, 128.09, 127.18, 126.94, 125.82, 125.12, 124.07, 124.04, 120.64, 119.79, 111.76, 105.57, 43.91; **HRMS(ESI)**: Calcd. for $C_{20}H_{12}NO_2S_2[M-H]^-$ 362.0315; found 362.0312.

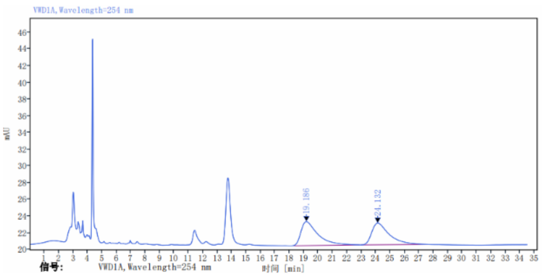

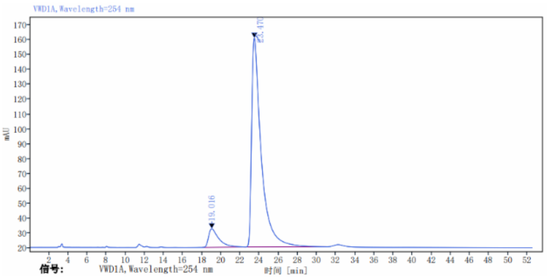

Peak	RetTime [min]	Type	Width[min]	Area[mAU^s]	Height[mAU]	Area%
	19.186	BB	4.7483	235.4645	2.8707	51.5297
	24.132	BB	4.7417	221.4850	2.5956	48.4703

Peak	RetTime [min]	Type	Width[min]	Area[mAU^s]	Height[mAU]	Area%
	19.016	BB	4.5117	929.0026	12.4736	8.6291
	23.470	BB	8.4800	9836.8713	140.2946	91.3709

(*R*)-6-hydroxy-5-(2-(methylthio)benzo[b]thiophen-3-yl)-2-naphthonitrile (6i):

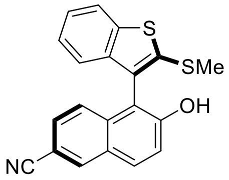

White solid (36.2 mg, 52%, 30 ^{o}C for 26 h); m.p. = 188-190 ^{o}C; R_f = 0.32 (petroleum ether/ethyl acetate = 5:1); the enantiomeric excess was determined to be 72% by HPLC analysis on Daicel Chirapak IA-H column (hexane/isopropanol = 90/10, flow rate 1 mL/min, T = 30 °C), UV 254 nm, t_R(major) 12.017 min,

t_R(minor) 10.249 min; $[\alpha]_D^{25}$ = -19.39 (*c* = 0.60, $CHCl_3$); **^{1}H NMR (600 MHz, $CDCl_3$)** δ 8.16 (s, 1H), 7.88 (d, J = 8.9 Hz, 1H), 7.80 (d, J = 8.0 Hz, 1H), 7.40 – 7.33 (m, 2H), 7.33 – 7.26 (m, 1H), 7.22 – 7.14 (m, 2H), 6.95 (d, J = 7.9 Hz, 1H), 5.40 (s, 1H), 2.42 (s, 3H); **^{13}C NMR (151 MHz, $CDCl_3$)** δ 154.37, 141.54, 139.91, 139.54, 135.10, 134.38, 131.21, 128.05, 127.50, 126.22, 125.78, 125.32, 124.84, 122.18, 122.16, 119.61, 119.44, 113.54, 106.92, 18.93; **HRMS(ESI)**: Calcd. for $C_{20}H_{12}NOS_2$[M-H]$^-$ 346.0366; found 346.0362.

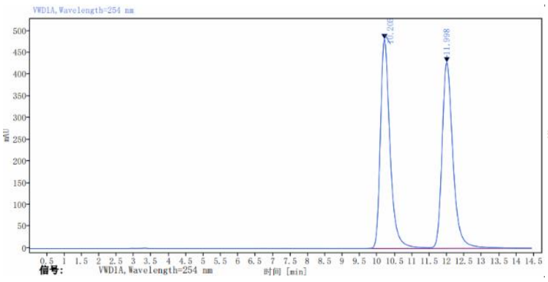

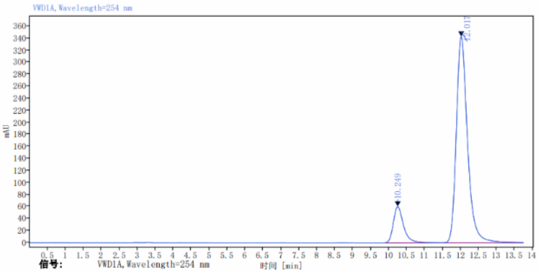

Peak	RetTime [min]	Type	Width[min]	Area[mAU*s]	Height[mAU]	Area%
	10.205	BV	1.7725	9265.2184	481.0769	49.6863
	11.998	VBA	2.9575	9382.2165	427.6392	50.3137

Peak	RetTime [min]	Type	Width[min]	Area[mAU*s]	Height[mAU]	Area%
	10.249	BB	1.6903	1207.8530	60.4666	13.9420
	12.017	BBA	2.2800	7455.5486	343.0430	86.0580

6-(4-fluoro-3-methylphenyl)-1-((*S*)-2-((*R*)methylsulfinyl)benzo[b]thiophen-3-yl)naphthalen-2-ol (7j):

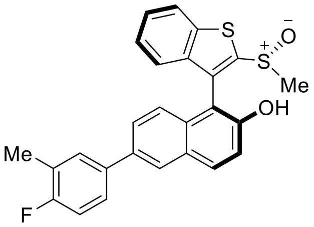

White solid (41.9 mg, 47%, 30 °C for 28 h); m.p. = 195-198 °C; R_f = 0.20 (petroleum ether/ethyl acetate = 1:2); the enantiomeric excess was determined to be 88% by HPLC analysis on Daicel Chirapak IA-H column (hexane/isopropanol = 90/10, flow rate 1 mL/min, T = 30 °C), UV 254 nm, t_R(major) 14.910 min, t_R(minor) 12.957 min; $[\alpha]_D^{25}$ = +52.85 (c = 0.48, $CHCl_3$); **^{1}H NMR (600 MHz, DMSO-d_6)** δ 10.18 (s, 1H), 8.21 (d, J = 8.1 Hz, 1H), 8.18 (s, 1H), 8.04 (d, J = 8.8 Hz, 1H), 7.67 (d, J = 6.5 Hz, 1H), 7.61 (d, J = 8.7 Hz, 1H), 7.57 (s, 1H), 7.55 – 7.49 (m, 1H), 7.40 (d, J = 8.9 Hz, 1H), 7.38 – 7.32 (m, 1H), 7.25 – 7.19 (m, 1H), 7.10 (d, J = 7.9 Hz, 1H), 7.02 (d, J = 8.6 Hz, 1H), 2.67 (s, 3H), 2.31 (s, 3H); **^{13}C NMR (151 MHz, DMSO-d_6)** δ 161.62, 160.01, 153.65, 148.18, 139.92, 139.56, 136.49, 134.24, 133.25, 133.15, 131.51, 130.36, 128.54, 126.80, 126.54, 126.30, 125.68, 125.21, 125.09, 124.42, 124.24, 123.98, 119.38, 115.76, 111.08, 43.92, 14.78; **HRMS(ESI)**: Calcd. for $C_{26}H_{18}FO_2S_2$[M-H]$^-$ 445.0738; found 445.0736.

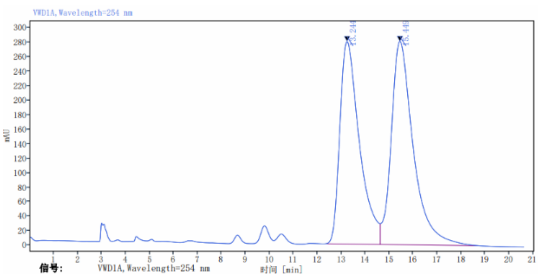

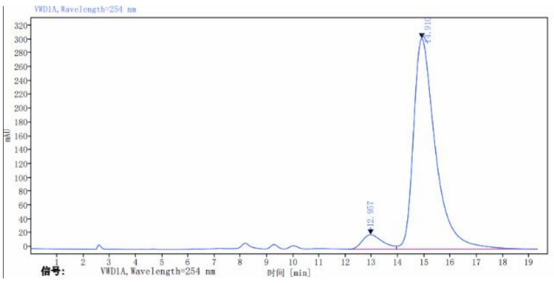

Peak	RetTime [min]	Type	Width[min]	Area[mAU*s]	Height[mAU]	Area%
	13.244	BV	2.2760	15869.3309	279.5127	46.9650
	15.449	VBA	4.1073	17920.3525	280.6473	53.0350

Peak	RetTime [min]	Type	Width[min]	Area[mAU*s]	Height[mAU]	Area%
	12.957	BV	1.8353	1134.4603	20.8639	6.0030
	14.910	VBA	5.3814	17763.8050	304.1964	93.9970

(*R*)-6-(4-fluoro-3-methylphenyl)-1-(2-(methylthio)benzo[b]thiophen-3-yl)naphthalen-2-ol (6j):

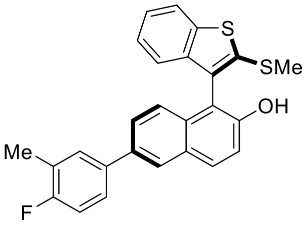

White semi-solid (42.9 mg, 50%, 30 °C for 28 h); R_f = 0.355 (petroleum ether/ethyl acetate = 5:1); the enantiomeric excess was determined to be 88% by HPLC analysis on Daicel Chirapak IA-H column (hexane/isopropanol = 70/30, flow rate 1 mL/min, T = 30 °C), UV 254 nm, t_R(major) 5.728 min, t_R(minor) 5.034 min; $[\alpha]_D^{25}$ = -24.06 (*c* = 0.46, $CHCl_3$); **^{1}H NMR (600 MHz, $CDCl_3$)** δ 7.91 (s, 1H), 7.87 (d, J = 8.8 Hz, 1H), 7.80 (d, J = 8.1 Hz, 1H), 7.46 – 7.38 (m, 2H), 7.35 (s, 1H), 7.30 – 7.27 (m, 2H), 7.20 – 7.15 (m, 2H), 7.05 (d, J = 8.0 Hz, 1H), 7.03 – 6.96 (m, 1H), 5.10 (s, 1H), 2.41 (s, 3H), 2.27 (s, 3H); **^{13}C NMR (151 MHz, $CDCl_3$)** δ 161.82, 160.20, 151.73, 140.57, 139.92, 136.85, 135.62, 132.38, 130.89, 130.31, 129.38, 128.25, 128.04, 126.37, 126.03, 125.15, 124.68, 124.37, 122.64, 122.04, 118.14, 115.40, 115.25, 112.87, 31.76, 19.06; **HRMS(ESI)**: Calcd. for $C_{26}H_{18}FO_2S_2$[M-H]$^-$ 429.0789; found 429.0786.

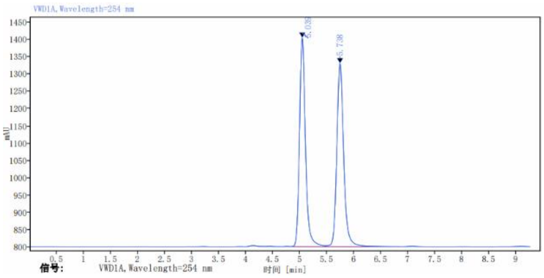

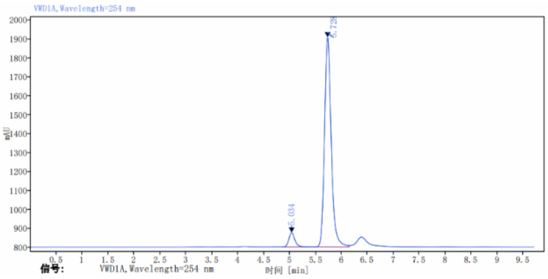

Peak	RetTime [min]	Type	Width[min]	Area[mAU*s]	Height[mAU]	Area%
	5.039	BV	0.6393	4651.6965	603.2697	49.6890
	5.738	VB	1.3890	4709.9190	529.4488	50.3110

Peak	RetTime [min]	Type	Width[min]	Area[mAU*s]	Height[mAU]	Area%
	5.034	VB	0.5842	622.3908	75.0352	5.8746
	5.728	BV	0.7070	9972.2110	1105.0129	94.1254

5. Scale-Up Reaction and Derivatization of Chiral Products

5.1 General procedure for the scale-up reaction

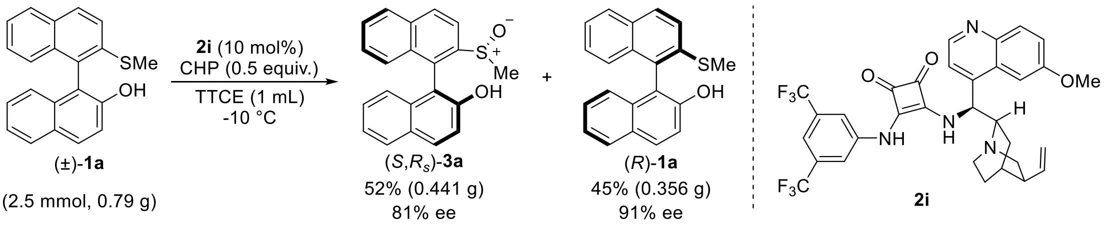

To a stirred solution of (±)-**1a** (790 mg, 2.5 mmol), catalyst **2i** (157.1 mg, 0.25 mmol) in TTCE (12.5 mL) cooled to -10 ℃ was added CHP (237.5 mg, 1.25 mmol) and the stirring was maintained at -10 ℃ for 13.5 h. The reaction was quenched with saturated Na_2SO_3 solution (10 mL) and extracted with CH_2Cl_2 (3 × 15 mL). The combined organic phase was washed with saturated NH_4Cl solution (2 × 15 mL), dried over Na_2SO_4 and concentrated. The residue was purified by column chromatography on silica gel eluted with petroleum ether/ethyl acetate (5:1 to 1:2) to afford the pure products. The enantiomeric excess of **3a** was determined to be 81%, and the enantiomeric excess of **1a** was determined to be 91%.

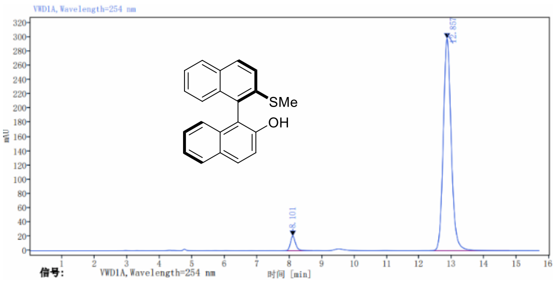

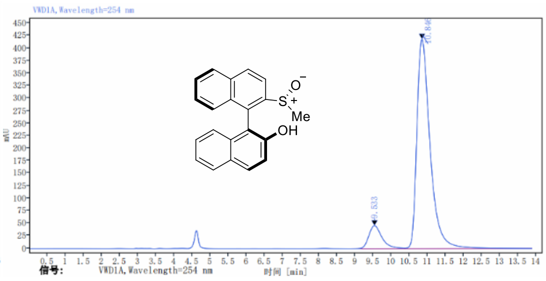

Peak	RetTime [min]	Type	Width[min]	Area[mAU*s]	Height[mAU]	Area%
	8.101	BB	0.9167	230.1507	20.4845	4.3072
	12.857	BB	2.4500	5113.1933	298.2754	95.6928

Peak	RetTime [min]	Type	Width[min]	Area[mAU*s]	Height[mAU]	Area%
	9.533	BV	1.4685	1168.6990	45.7823	9.8927
	10.846	VBA	3.5182	10645.0626	418.5971	90.1073

5.2 Procedures for the synthesis of compounds (*S*)-1a and 11-16

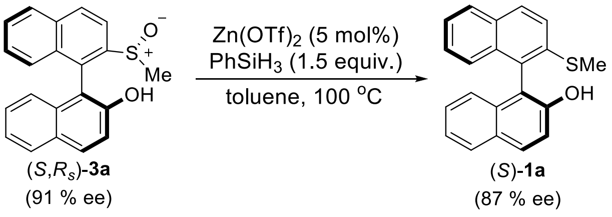

The procedure for the synthesis of (*S*)-1a: To a 10 mL Schlenk tube with (*S*,*R*$_s$)-**3a** (0.1 mmol), $PhSiH_3$ (0.15 mmol) and $Zn(OTf)_2$ (0.005 mmol) was added 0.3 mL toluene, and the mixture was stirred at 100 ^{o}C for 36 h. After the reaction is completed, the solvent was removed by rotary

evaporation, and the residue is purified by flash chromatography column on silica gel with petroleum ether/ethyl acetate (5:1) to obtain the (*S*)-**1a** (28.3 mg, 90% yield). The enantiomeric excess of (*S*)-**1a** was determined to be 87%.

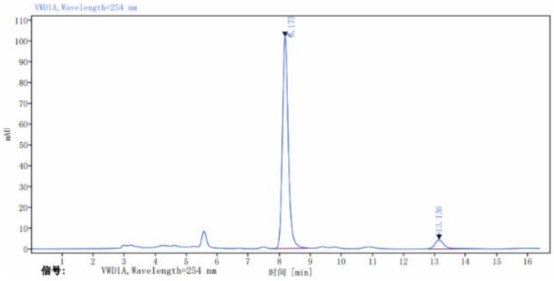

Peak	RetTime [min]	Type	Width[min]	Area[mAU^s]	Height[mAU]	Area%
	8.175	BB	1.2700	1345.0785	101.7332	93.4677
	13.136	BB	2.2917	94.0056	4.3372	6.5323

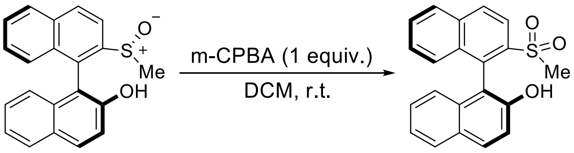

(*S*,R_s)-**3a** 94% ee → (*S*)-**11** 91% yield, 95% ee

The procedure for the synthesis of compound 11: To a solution of (*S*,R_s)-**3a** (0.1 mmol) in DCM (1 mL) was added *m*-CPBA (0.15 mmol), and the mixture was stirred at room temperature for 8 h. After the reaction is completed, the solvent was removed by rotary evaporation, and the residue was purified by flash chromatography column on silica gel with petroleum ether/ethyl acetate (1:1).

(*S*)-2'-(methylsulfonyl)-[1,1'-binaphthalen]-2-ol (11):

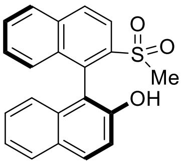

Colorless solid (31.6 mg, 91%); R_f = 0.33 (petroleum ether/ethyl acetate = 1:1); the enantiomeric excess was determined to be 95% by HPLC analysis on Daicel Chirapak IA-H column (hexane/isopropanol = 80/20, flow rate 1 mL/min, T = 30 °C), UV 254 nm, t_R(major) 10.130 min, t_R(minor) 8.366 min; $[\alpha]_D^{25}$ = +55.55 (*c* = 0.60, $CHCl_3$); **^{1}H NMR (600 MHz, DMSO-*d*$_6$)** δ 9.89 (s, 1H), 8.30 (d, J = 8.9 Hz, 1H), 8.22 (d, J = 8.9 Hz, 1H), 8.15 (d, J = 8.2 Hz, 1H), 7.97 (d, J = 8.9 Hz, 1H), 7.88 (s, 1H), 7.71 – 7.65 (m, 1H), 7.45 – 7.39 (m, 1H), 7.35 (d, J = 8.9 Hz, 1H), 7.26 (d, J = 7.0 Hz, 1H), 7.18 (s, 1H), 7.09 (d, J = 8.5 Hz, 1H), 6.67 (d, J = 8.4 Hz, 1H), 2.95 (s, 3H); **^{13}C NMR (151 MHz, DMSO-*d*$_6$)** δ 152.88, 138.22, 136.84, 135.54, 134.93, 133.09, 130.62, 129.43, 129.28, 128.77, 128.31, 128.15, 128.06, 127.28, 126.81, 125.01, 124.52, 123.12, 118.51, 115.52, 43.76; **HRMS(ESI)**: Calcd. for

$C_{21}H_{15}O_3S$[M-H]$^-$ 347.0747; found 347.0743.

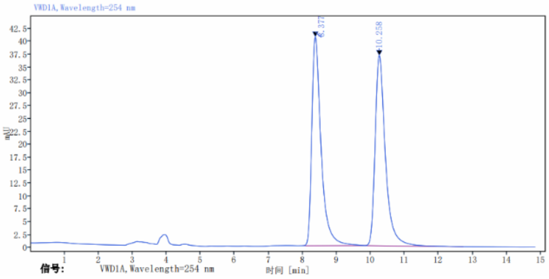

Peak	RetTime [min]	Type	Width[min]	Area[mAU^s]	Height[mAU]	Area%
	8.377	BB	1.8383	770.4675	40.4553	49.7686
	10.258	BB	2.8967	777.6335	36.8540	50.2314

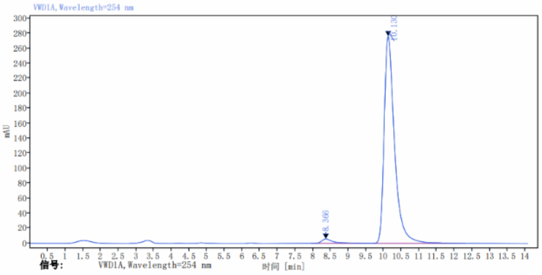

Peak	RetTime [min]	Type	Width[min]	Area[mAU^s]	Height[mAU]	Area%
	8.366	BB	1.6633	142.3675	5.8178	2.4555
	10.130	BB	2.6967	5655.4319	276.2282	97.5445

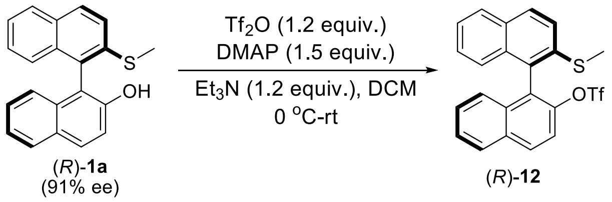

The procedure for the synthesis of compound 12: Added (*R*)-**1a** (379.2 mg, 1.2 mmol), DMAP (219.6 mg, 1.8 mmol), Et_3N (0.2 mL, 1.44 mmol) and CH_2Cl_2 (5 mL) to a dry round-bottom flask. Stired the reaction mixture and cool to 0 ℃. Added Tf_2O (0.24 mL, 1.44 mmol) dropwise to the stirring reaction mixture at 0 ℃. Leaved the reaction mixture to warm to room temperature overnight and quenched with water (5 mL). Separated the phases and extracted the aqueous fraction with CH_2Cl_2 (3 × 10 mL). Wash the combined organic phases with brine then dry with anhydrous Na_2SO_4. Finally, the solvent was removed by rotary evaporation, and the residue was purified by flash chromatography column on silica gel (petroleum ether/ethyl acetate = 20:1).

(*R*)-2'-(methylthio)-[1,1'-binaphthalen]-2-yl trifluoromethanesulfonate (12):

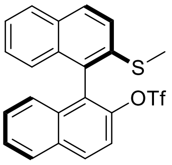

Colorless solid (394.9 mg, 76%); R_f = 0.60 (petroleum ether/ethyl acetate = 10:1); **^{1}H NMR (600 MHz, $CDCl_3$)** δ 8.00 (d, J = 9.1 Hz, 1H), 7.96 – 7.88 (m, 2H), 7.81 (d, J = 8.2 Hz, 1H), 7.52 (dd, J = 15.4, 8.9 Hz, 2H), 7.51 – 7.42 (m, 1H), 7.37 – 7.31 (m, 1H), 7.31 – 7.25 (m, 1H), 7.23 – 7.17 (m, 1H), 7.15 (d, J = 8.5 Hz, 1H), 6.95 (d, J = 8.5 Hz, 1H), 2.36 (s, 3H); **^{13}C NMR (151 MHz, $CDCl_3$)** δ 145.08, 137.21, 133.30, 132.90, 132.56, 131.33, 130.83, 129.74, 128.70, 128.38, 128.08, 127.98, 127.81, 127.11, 127.03, 126.47, 125.46, 125.31, 123.87, 121.34, 119.61, 119.22, 117.09, 114.97, 16.31; **HRMS(ESI)**: Calcd. for $C_{22}H_{16}F_3O_3S_2$ [M+H]$^+$ 449.0487; found 449.0486.

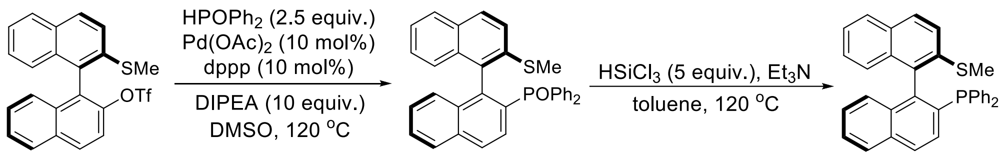

The procedure for the synthesis of compound 13: To a 10 mL dry Schlenk tube charged with (*R*)-**12** (0.1 mmol), $HPOPh_2$ (0.25 mmol), $Pd(OAc)_2$ (0.01 mmol) , DIPEA (1 mmol) and dppp (0.01 mmol) was added DMSO (2 mL), and the mixture was stirred under nitrogen atmosphere at 120 °C for 18 h. After the reaction is completed, water and DCM are added to the reaction system for extraction. Wash the combined organic phases with H_2O (3 × 3 mL). Then dry the organic phase with anhydrous Na_2SO_4. Finally, the solvent was removed by rotary evaporation, and the residue was purified by flash chromatography column on silica gel (petroleum ether/ethyl acetate = 2:3).

The procedure for the synthesis of compound 14: To a 10 mL dry Schlenk tube charged with (*R*)-**12** (0.1 mmol), $HSiCl_3$ (0.5 mmol) and Et_3N (2.4 mmol) was added 2 mL toluene, and the mixture was stirred under nitrogen atmosphere at room temperature for 18 h. After the reaction is completed, the solvent was removed by rotary evaporation, and the residue was purified by flash chromatography column on silica gel (petroleum ether/ethyl acetate = 20:1).

(*R*)-(2'-(methylthio)-[1,1'-binaphthalen]-2-yl)diphenylphosphine oxide (13):

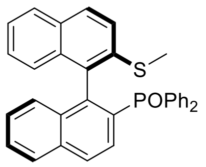

Colorless solid (35.8 mg, 72%); R_f = 0.25 (petroleum ether/ethyl acetate = 4:1); the enantiomeric excess was determined to be 91% by HPLC analysis on Daicel Chirapak IA-H column (hexane/isopropanol = 80/20, flow rate 1 mL/min, T = 30 °C), UV 254 nm, t_R(major) 20.657 min, t_R(minor) 11.924 min; $[\alpha]_D^{25}$ = +1.675 (*c* = 0.870, $CHCl_3$); **^{1}H NMR (600 MHz, DMSO-*d*$_6$)** δ 8.13 (d, J = 8.5 Hz, 1H), 8.07 (s, 1H), 7.85 – 7.76 (m, 2H), 7.66 – 7.58 (m, 2H), 7.47 – 7.39 (m, 2H), 7.34 (ddd, J = 15.4, 8.8, 5.9 Hz, 9H), 7.33 – 7.24 (m, 2H), 7.13 – 7.07 (m, 1H), 6.89 (d, J = 8.6 Hz, 1H), 6.72 (d, J = 8.5 Hz, 1H), 2.27 (s, 3H); **^{13}C NMR (151 MHz, DMSO-*d*$_6$)** δ 142.00, 136.82, 134.81, 133.40, 133.22, 132.64, 132.56, 131.80, 131.75, 131.71, 131.65, 131.61, 131.55, 131.01, 130.49, 129.37, 129.29, 129.20, 128.74, 128.68, 128.50, 128.42, 128.39, 128.29, 128.22, 128.19, 127.85, 126.64, 126.34, 125.45, 125.14, 123.87, 15.76; **HRMS(ESI)**: Calcd. for $C_{33}H_{26}OPS[M+H]^+$ 501.1437; found 501.1433.

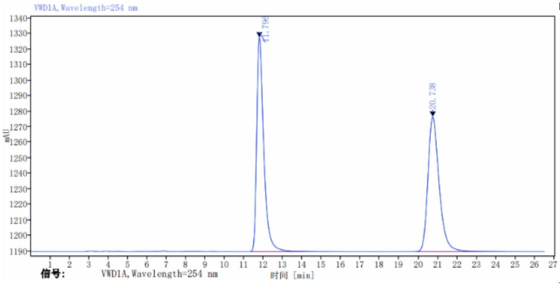

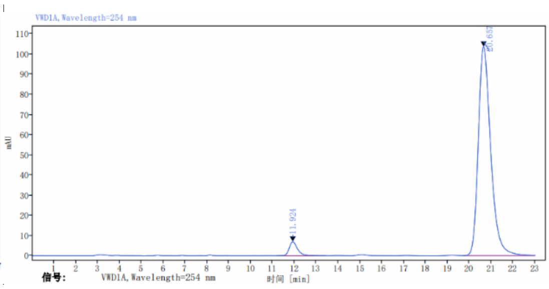

Peak	RetTime [min]	Type	Width[min]	Area[mAU*s]	Height[mAU]	Area%
	11.796	BB	3.5217	3501.7042	137.8955	49.9243
	20.738	BB	4.8283	3512.3165	86.7879	50.0757

Peak	RetTime [min]	Type	Width[min]	Area[mAU*s]	Height[mAU]	Area%
	11.924	BB	2.2833	186.6424	6.9131	4.3544
	20.657	BBA	3.3167	4099.6735	103.4317	95.6456

(*R*)-(2'-(methylthio)-[1,1'-binaphthalen]-2-yl)diphenylphosphane (14):

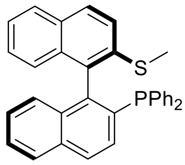

Colorless solid (36.1 mg, 75%); R_f = 0.56 (petroleum ether/ethyl acetate = 10:1); the enantiomeric excess was determined to be 91% by HPLC analysis on Daicel Chirapak IA-H column (hexane/isopropanol = 90/10, flow rate 1 mL/min, T = 30 °C), UV 254 nm, t_R(major) 4.053 min, t_R(minor) 4.522 min; $[\alpha]_D^{25}$ = +4.24 (*c* = 0.267, $CHCl_3$); **^{1}H NMR (600 MHz, $CDCl_3$)** δ 7.89 (d, J = 8.7 Hz, 1H), 7.82 (dd, J = 8.2, 5.6 Hz, 2H), 7.75 (d, J = 8.2 Hz, 1H), 7.50 (d, J = 8.8 Hz, 1H), 7.42 – 7.37 (m, 2H), 7.24 – 7.18 (m, 6H), 7.13 – 7.05 (m, 3H), 7.05 – 7.01 (m, 2H), 6.99 – 6.93 (m, 2H), 6.88 - 6.84 (m, 1H), 6.62 (d, J = 8.5 Hz, 1H), 2.23 (s, 3H); **^{13}C NMR (151 MHz, $CDCl_3$)** δ 143.53, 136.40, 134.03, 133.90, 133.82, 133.45, 133.21, 133.09, 132.04, 131.71, 131.64, 130.97, 130.52, 130.06, 128.77, 128.44, 128.42, 128.33, 128.25, 128.16, 128.12, 128.07, 127.95, 127.79, 127.66, 127.55, 127.46, 127.38, 126.94, 126.74, 126.68, 126.31, 126.25, 125.74, 125.01, 124.83, 123.25, 15.98.

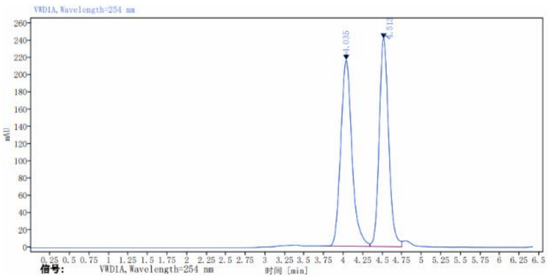

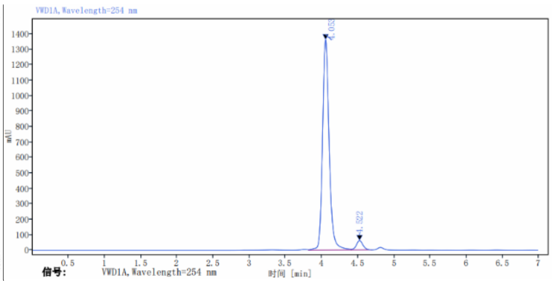

Peak	RetTime [min]	Type	Width[min]	Area[mAU*s]	Height[mAU]	Area%
	4.035	BV	0.6358	2160.9185	215.7815	51.7170
	4.513	VV	0.4085	2017.4316	240.9121	48.2830

Peak	RetTime [min]	Type	Width[min]	Area[mAU*s]	Height[mAU]	Area%
	4.053	VV	0.5947	8686.4007	1361.3335	95.7249
	4.522	VB	0.2932	387.9370	60.6484	4.2751

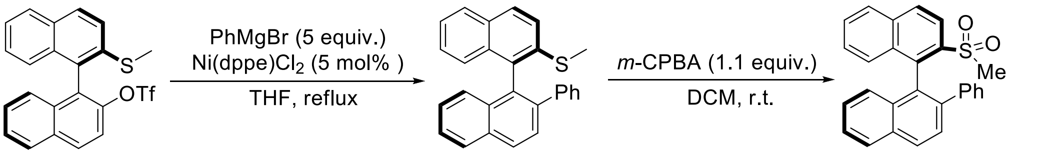

(*R*)-**12** (*R*)-**15** (*R*)-**16**

The procedure for the synthesis of compound 15: Under nitrogen protection, (*R*)-**12** (0.1 mmol,

43.2 mg) and $NiCl_2$(dppe) (0.005 mmol, 2.64 mg) were dissolved in 0.5 mL of redistilled THF. The Grignard reagent (0.5 mL, 1 M) was added dropwise at 0 ℃, and heated to reflux for 12 h. After the reaction is complete, quench with 1 mL saturated NH_4Cl solution and extract the aqueous phase with ethyl acetate (3 × 3 mL). Then dry the organic phase with anhydrous Na_2SO_4. Finally, the solvent was removed by rotary evaporation, and the residue was purified by flash chromatography column on silica gel (petroleum ether/ethyl acetate = 20:1).

The procedure for the synthesis of compound 16: To a 10 mL dry Schlenk tube charged with (*R*)-**15** (0.1 mmol) and *m*-CPBA (0.11 mmol) was added 1 mL DCM, and the mixture was stirred at room temperature for 8 h. After the reaction is completed, the solvent was removed by rotary evaporation, and the residue was purified by flash chromatography column on silica gel (petroleum ether/ethyl acetate = 1:1).

(*R*)-2'-(methylthio)-[1,1'-binaphthalen]-2-yl trifluoromethanesulfonate (15):

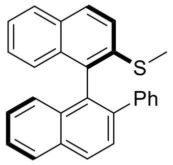

Colorless solid (36.1 mg, 95%); R_f = 0.56 (petroleum ether/ethyl acetate = 10:1); $[\alpha]_D^{25}$ = +60.57 (*c* = 0.64, $CHCl_3$); **^{1}H NMR (600 MHz, $CDCl_3$)** δ 7.96 (d, J = 8.5 Hz, 1H), 7.88 (d, J = 8.2 Hz, 1H), 7.71 (dd, J = 14.1, 8.5 Hz, 2H), 7.57 (d, J = 8.5 Hz, 1H), 7.41 – 7.35 (m, 1H), 7.30 (d, J = 8.7 Hz, 1H), 7.28 – 7.22 (m, 1H), 7.21 – 7.17 (m, 1H), 7.17 – 7.11 (m, 1H), 7.10 – 7.05 (m, 4H), 6.93 (d, J = 6.7 Hz, 3H), 2.20 (s, 3H); **^{13}C NMR (151 MHz, $CDCl_3$)** δ 140.51, 138.85, 135.54, 132.85, 132.68, 132.27, 131.83, 131.49, 129.86, 127.84, 127.44, 127.29, 127.07, 126.91, 126.20, 125.64, 125.52, 125.44, 125.30, 124.83, 124.80, 123.85, 122.23, 15.00; **HRMS(ESI)**: Calcd. for $C_{27}H_{20}NaS[M+Na]^+$ 399.1178; found 399.1178.

(*R*)-2-(methylsulfonyl)-2'-phenyl-1,1'-binaphthalene (16):

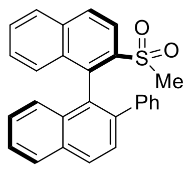

Colorless solid (36.1 mg, 88%); R_f = 0.33 (petroleum ether/ethyl acetate = 2:1); the enantiomeric excess was determined to be 91% by HPLC analysis on Daicel Chirapak IA-H column (hexane/isopropanol = 90/10, flow rate 1 mL/min, T = 30 °C), UV 254 nm, t_R(major) 11.981 min, t_R(minor) 20.410 min; $[\alpha]_D^{25}$ = +60.57 (*c* = 0.640, $CHCl_3$); **^{1}H NMR (600 MHz, $CDCl_3$)** δ 8.11 – 8.00 (m, 3H), 7.96 (dd, J = 17.6, 8.2 Hz, 2H), 7.69 (d, J = 8.5 Hz, 1H), 7.64 – 7.58 (m, 1H), 7.49 – 7.43 (m, 1H), 7.43 – 7.35 (m, 2H), 7.24 (d, J = 7.3 Hz, 1H), 7.10 (d, J = 6.8 Hz, 2H), 7.06 – 6.98 (m, 3H), 6.95 (d, J = 8.5 Hz, 1H), 2.43 (s, 3H); **^{13}C NMR (151 MHz, $CDCl_3$)** δ 141.26, 139.83, 138.63, 136.82, 134.71, 134.23, 132.91,

132.43, 130.76, 129.36, 129.00, 128.77, 128.56, 128.48, 128.39, 128.24, 127.89, 127.59, 126.75, 126.68, 126.33, 125.82, 124.37, 43.92.; **HRMS(ESI)**: Calcd. for $C_{27}H_{21}O_2S[M+H]^+$ 409.1257; found 409.1254.

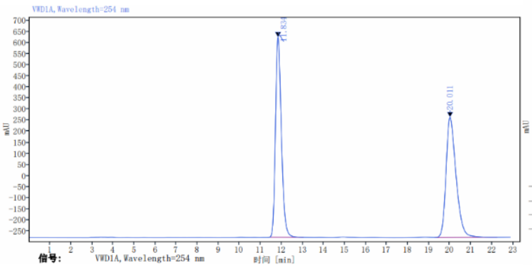

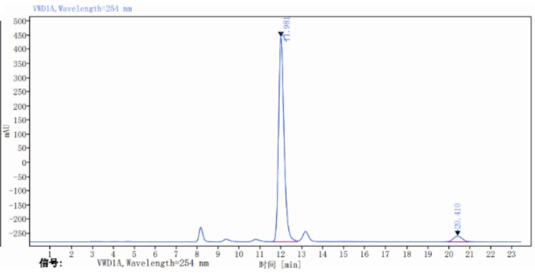

Peak	RetTime [min]	Type	Width[min]	Area[mAU^s]	Height[mAU]	Area%
	11.834	MM m	0.3012	17645.2247	902.0882	49.6189
	20.011	VB	2.9895	17916.2411	542.4053	50.3811

Peak	RetTime [min]	Type	Width[min]	Area[mAU^s]	Height[mAU]	Area%
	11.981	VV	1.2973	13260.7337	723.5634	95.3993
	20.410	BB	2.4033	639.5053	20.8257	4.6007

6. Crystal data and structure of compound (*S*, *R*$_s$)-3a

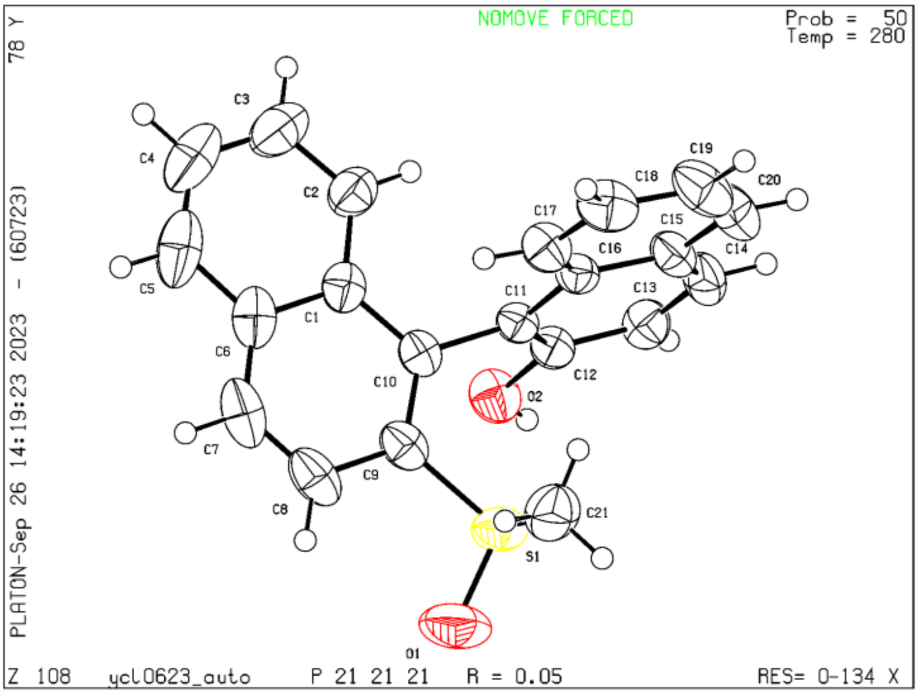

Figure S1. X-ray crystal structure of (*S*,*R*$_s$)-3a (CCDC 2330721)**.**

Chemical Formula	$C_{21}H_{16}O_2S$
Formula weight	332.40
Temperature	280 K
Wavelength	1.54184
Crystal system, space group	Orthorhombic, P21 21 21
a, Ǻ	10.1877(3)
b, Ǻ	11.4503(3)
c, Ǻ	14.7540(3)
α, °	90
β, °	90
γ, °	90
V, Ǻ^3	1721.10(7)
F (000)	696.0
Z, Calculated density	4, 1.283 g/cm^3

7. Control experiment for mechanism investigation

7.1 Investigation of the loading of the oxidant

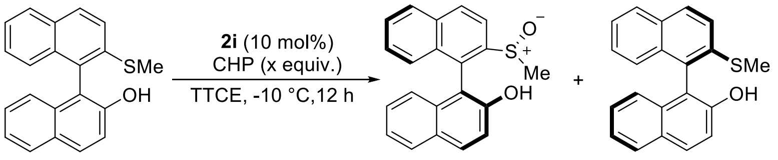

(±)-**1a** (*S*,*R*s)-**3a** (*R*)-**1a**

entry	x (equiv.)	1a		3a	
		yield (%)[b]	ee (%)[c]	yield (%)[b]	ee (%)[c]
1	0.1	77	22	23	95
2	0.3	63	45	37	94
3	0.5	48	93	47	93
4	0.7	37	99	63	38
5	0.9	22	99	78	19

[a]Unless noted otherwise, reactions were performed with (±)-**1a** (0.1 mmol), **2i** (0.01 mmol), CHP (x equiv.), in TTCE (0.5 mL) at -10 ℃. [b]Isolated yield. [c]Determined by chiral HPLC analysis.

7.2 Investigation of the structure of substrates

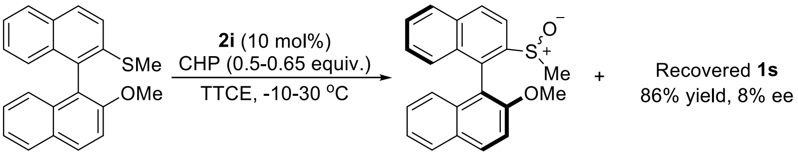

(±)-**1s**

3s
10% yield
dr = 52:48, 20% ee (24% ee)

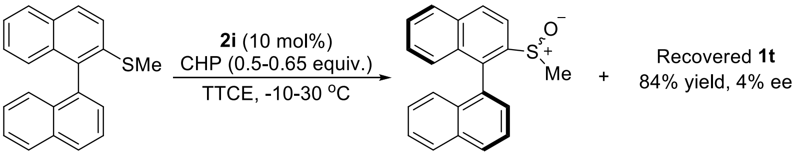

(±)-**1t**

3t
11% yield
dr=63:37, 6% ee (25% ee)

A dry Schlenk tube was charged with (±)-**1s** or (±)-**1t** (0.1 mmol), catalyst **2i** (6.3 mg, 0.01 mmol) and TTCE (0.5 mL). The mixture was cooled to -10 ℃ and stirred for 5 min, then the CHP (9.5 mg, 0.05 mmol) was added and the stirring was maintained at -10 ℃ for 24 h. Through TCl monitoring, no reaction occurred. Then, added 0.15 equiv. CHP to the mixture and stirred at 30 ^{o}C for an additional 24 h. The reaction was quenched with saturated Na_2SO_3 solution (2 mL) and extracted with CH_2Cl_2 (3 × 5 mL). The combined organic phase was washed with saturated NH_4Cl solution (2 × 5 mL), dried over Na_2SO_4 and concentrated. The residue was purified by column

chromatography on silica gel eluted with petroleum ether/ethyl acetate (5:1 to 1:2) to afford the pure product.

(2'-methoxy-[1,1'-binaphthalen]-2-yl)(methyl)sulfane (1s):

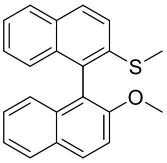

White semi-solid; R_f = 0.47 (petroleum ether/ethyl acetate = 5:1); **^{1}H NMR (600 MHz, $CDCl_3$)** δ7.92 (d, J = 9.0 Hz, 1H), 7.85 (d, J = 8.7 Hz, 1H), 7.78 (d, J = 8.1 Hz, 2H), 7.49 (d, J = 8.7 Hz, 1H), 7.37 (d, J = 9.0 Hz, 1H), 7.32 – 7.26 (m, 1H), 7.26 – 7.20 (m, 1H), 7.15 – 7.12 (m, 2H), 7.02 (d, J = 8.5 Hz, 1H), 6.92 (s, 1H), 3.69 (s, 3H), 2.30 (s, 3H); **^{13}C NMR (151 MHz, $CDCl_3$)** δ155.00, 136.36, 133.48, 133.25, 131.64, 131.42, 130.09, 129.27, 128.39, 128.09, 128.05, 126.73, 126.62, 125.39, 125.00, 124.84, 123.78, 123.51, 120.97, 114.10, 56.90, 15.98; **HRMS(ESI)**: Calcd. for $C_{22}H_{19}OS[M+H]^+$ 331.1151; found 331.1147.

The dr of **3s** was determined to be 52:48, and the enantiomeric excess of **3s** was determined to be 24% and 20%, respectively, by HPLC analysis on Daicel Chirapak OD-H column (hexane/isopropanol = 70/30, flow rate 1 mL/min, T = 30 °C), UV 254 nm, t_{R1}(major) 7.577 min, t_{R1}(minor) 6.691 min, t_{R2}(major) 9.465 min, t_{R1}(minor) 5.994 min.

The enantiomeric excess of recovered **1s** was determined to be 8% by HPLC analysis on Daicel Chirapak OD-H column (hexane/isopropanol = 70/30, flow rate 1 mL/min, T = 30 °C), UV 254 nm, t_R(major) 6.904 min, t_R(minor) 6.331 min.

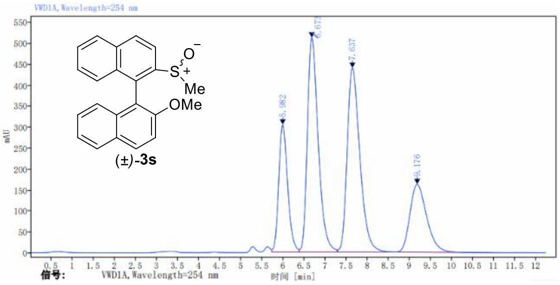

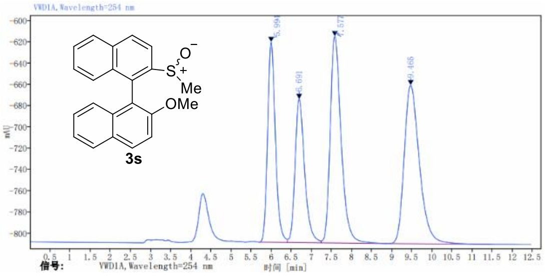

Peak	RetTime [min]	Type	Width[min]	Area[mAU*s]	Height[mAU]	Area%
	5.982	VV	0.6531	4483.5293	303.5333	16.4077
	6.673	VV	0.9054	9155.3141	511.0565	33.5043
	7.637	VB	1.3631	9201.5701	438.8728	33.6735
	9.176	BB	2.3104	4485.3971	161.7951	16.4145

Peak	RetTime [min]	Type	Width[min]	Area[mAU*s]	Height[mAU]	Area%
	5.994	BV	0.7135	2531.2712	187.7007	20.5151
	6.691	VV	0.8351	2226.6774	134.4826	18.0465
	7.577	VB	1.6612	3664.5350	193.8187	29.6998
	9.465	BB	2.5733	3916.0891	148.5774	31.7386

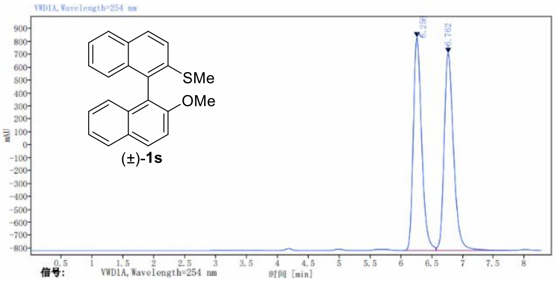

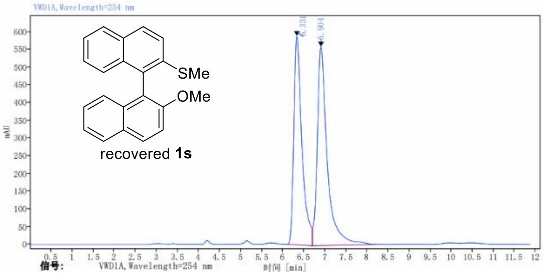

Peak	RetTime [min]	Type	Width[min]	Area[mAU^s]	Height[mAU]	Area%
	6.256	BV	0.5424	15546.1601	1646.2029	48.9288
	6.762	VV	1.1882	16226.8582	1525.0721	51.0712

Peak	RetTime [min]	Type	Width[min]	Area[mAU^s]	Height[mAU]	Area%
	6.331	BM m	0.1985	7890.8149	588.1404	45.9813
	6.904	MB m	0.2410	9270.0918	560.3181	54.0187

[1,1'-binaphthalen]-2-yl(methyl)sulfane (1t):

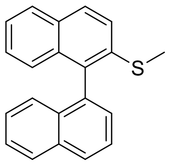

White semi-solid; R_f = 0.52 (petroleum ether/ethyl acetate = 4:1); **^{1}H NMR (600 MHz, $CDCl_3$)** δ7.97 (d, J = 8.3 Hz, 1H), 7.95 – 7.90 (m, 2H), 7.85 (d, J = 8.2 Hz, 1H), 7.64 – 7.58 (m, 1H), 7.55 (d, J = 8.7 Hz, 1H), 7.47 – 7.43 (m, 1H), 7.41 (d, J = 6.9 Hz, 1H), 7.39 – 7.33 (m, 1H), 7.28 – 7.24 (m, 1H), 7.23 – 7.18 (m, 2H), 7.09 (d, J = 8.6 Hz, 1H), 2.36 (s, 3H); **^{13}C NMR (151 MHz, $CDCl_3$)** δ 136.16, 136.01, 134.88, 133.75, 133.35, 132.33, 131.14, 128.45, 128.44, 128.36, 128.29, 127.84, 126.61, 126.22, 125.95, 125.75, 125.72, 125.63, 125.01, 123.14, 16.07; **HRMS(ESI)**: Calcd. for $C_{21}H_{17}S[M+H]^+$ 301.1045; found 301.1043.

The dr of **3t** was determined to be 63:37, and the enantiomeric excess of **3t** was determined to be 6% and 25%, respectively, by HPLC analysis on Daicel Chirapak AS-H column (hexane/isopropanol = 90/10, flow rate 1 mL/min, T = 30 °C), UV 254 nm, t_{R1}(major) 11.184 min, t_{R1}(minor) 12.112 min, t_{R2}(major) 13.673 min, t_{R1}(minor) 10.358 min.

The enantiomeric excess of recovered **1t** was determined to be 4% by HPLC analysis on Daicel Chirapak OD-H column (hexane/isopropanol = 90/10, flow rate 1 mL/min, T = 30 °C), UV 254 nm, t_R(major) 13.855 min, t_R(minor) 13.194 min.

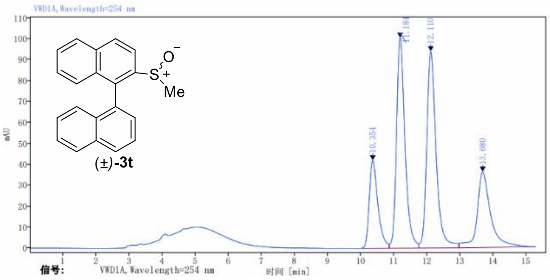

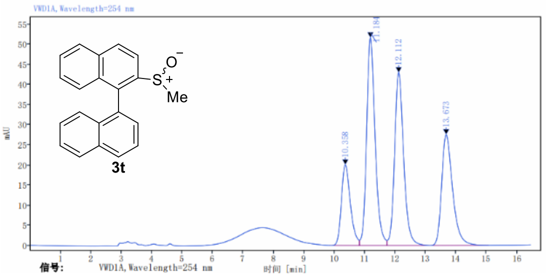

Peak	RetTime [min]	Type	Width[min]	Area[mAU^s]	Height[mAU]	Area%
	10.354	BM m	0.2751	765.2772	41.9050	14.1040
	11.184	MM m	0.2690	1781.1766	100.3709	32.8269
	12.110	MM m	0.2978	1848.6597	93.8479	34.0706
	13.680	MB m	0.4154	1030.8469	36.0758	18.9984

Peak	RetTime [min]	Type	Width[min]	Area[mAU^s]	Height[mAU]	Area%
	10.358	MM m	0.3130	410.5852	20.0435	13.6984
	11.184	VV	0.9068	1004.6835	51.6772	33.5194
	12.112	VB	1.4146	891.8419	42.9816	29.7546
	13.673	BB	1.9233	690.2087	27.5486	23.0275

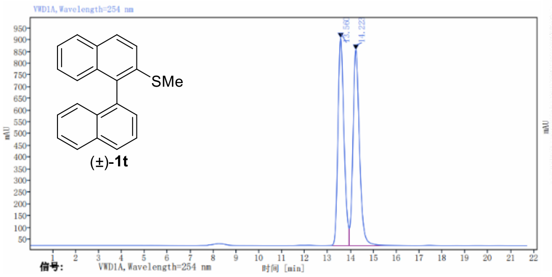

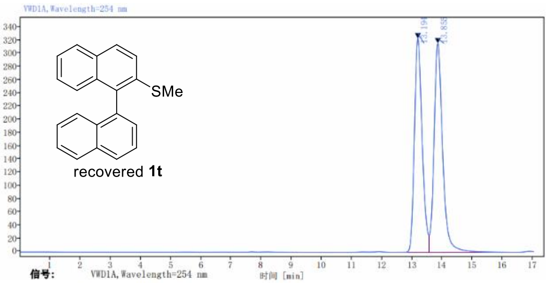

Peak	RetTime [min]	Type	Width[min]	Area[mAU^s]	Height[mAU]	Area%
	13.560	BV	1.1185	16271.2699	884.0704	49.4843
	14.223	VB	2.9148	16610.4236	833.7731	50.5157

Peak	RetTime [min]	Type	Width[min]	Area[mAU^s]	Height[mAU]	Area%
	13.194	BV	0.8352	5763.5618	323.4496	47.8173
	13.855	VB	2.8948	6289.7314	316.0379	52.1827

7.3 Investigation of the chiral matching

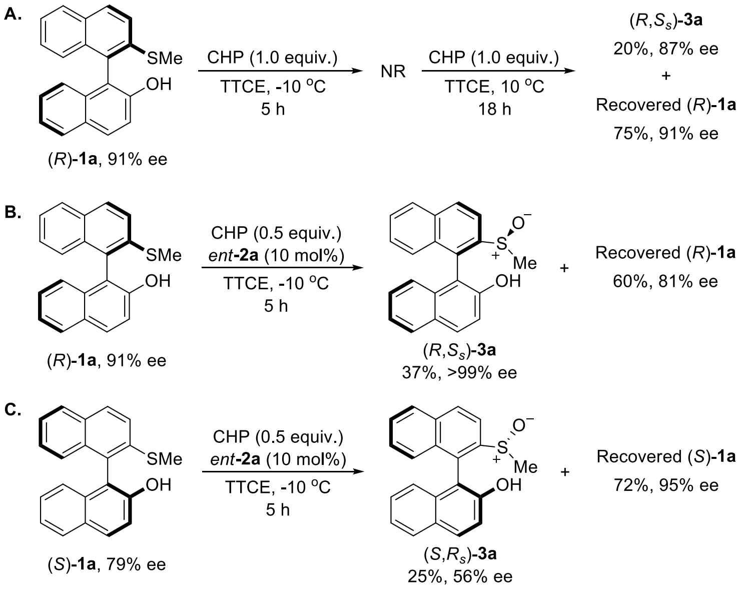

Condition A: An oven-dried 10 mL Schlenk tube equipped with a stir bar was charged with (*R*)-**1a** (31.6 mg, 0.1 mmol) and DCE (0.5 mL), the mixture was stirred in for about 5 min at -10 ℃, then the CHP (19.0 mg, 0.1 mmol) was added and the stirring was maintained at -10 ℃ for 5 h. Through TCl monitoring, no reaction occurred. The solution was stirred for an additional 18 h at 30 ^{o}C. The reaction was quenched with saturated Na_2SO_3 solution (0.5 mL) and extracted with CH_2Cl_2 (3 × 2 mL). The combined organic phase was washed with saturated NH_4Cl solution (2 × 2 mL), dried over Na_2SO_4 and concentrated. The residue was purified by column chromatography on silica gel eluted with petroleum ether/ethyl acetate (5:1 to 1:2) to afford the pure products. The generated **3a** and recovered **1a** were analyzed by HPLC to determine the enantiomeric excess.

Condition B: An oven-dried 10 mL Schlenk tube equipped with a stir bar was charged with (*R*)-**1a** (31.6 mg, 0.1 mmol), *ent*-**2a** (4.5 mg, 0.01 mmol) and DCE (0.5 mL), the mixture was stirred in for about 5 min at -10 ℃, then the CHP (19.0 mg, 0.1 mmol) was added and the stirring was maintained at -10 ℃ for 5 h. The reaction was quenched with saturated Na_2SO_3 solution (0.5 mL) and extracted with CH_2Cl_2 (3 × 2 mL). The combined organic phase was washed with saturated NH_4Cl solution (2 × 2 mL), dried over Na_2SO_4 and concentrated. The residue was purified by column chromatography on silica gel eluted with petroleum ether/ethyl acetate (5:1 to 1:2) to

afford the pure products. The generated **3a** and recovered **1a** were analyzed by HPLC to determine the enantiomeric excess.

Condition C: An oven-dried 10 mL Schlenk tube equipped with a stir bar was charged with (*S*)-**1a** (31.6 mg, 0.1 mmol), *ent*-**2a** (4.5 mg, 0.01 mmol) and DCE (0.5 mL), the mixture was stirred in for about 5 min at -10 ℃, then the CHP (19.0 mg, 0.1 mmol) was added and the stirring was maintained at -10 ℃ for 5 h. The reaction was quenched with saturated Na_2SO_3 solution (0.5 mL) and extracted with CH_2Cl_2 (3 × 2 mL). The combined organic phase was washed with saturated NH_4Cl solution (2 × 2 mL), dried over Na_2SO_4 and concentrated. The residue was purified by column chromatography on silica gel eluted with petroleum ether/ethyl acetate (5:1 to 1:2) to afford the pure products. The generated **3a** and recovered **1a** were analyzed by HPLC to determine the enantiomeric excess.

7.4 Investigation of the hydrogen bonding of 3a

The ^{1}H NMR spectra of **3a** with different enantiomeric excess (in $CDCl_3$).

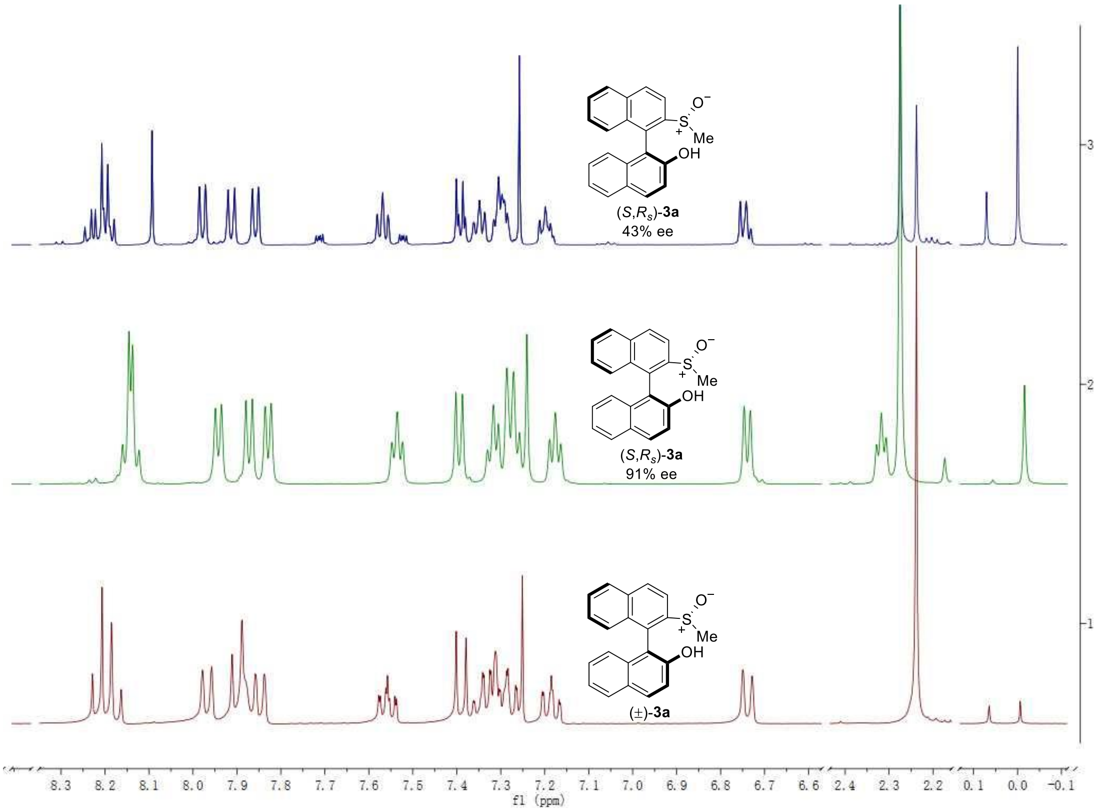

8. References

[1] Yanagi, T.; Otsuka, S.; Kasuga, Y.; Fujimoto, K.; Murakami, K.; Nogi, K.; Osuka, A. Metal-Free Approach to Biaryls from Phenols and Aryl Sulfoxides by Temporarily Sulfur-Tethered Regioselective C–H/C–H Coupling. *J. Am. Chem. Soc.* **2016**, *138*, 14582–14585.

[2] Vakulya, B.; Varga, S.; Csámpai, A.; Soós, T. Highly Enantioselective Conjugate Addition of Nitromethane to Chalcones Using Bifunctional Cinchona Organocatalysts. *Org. Lett.* **2005**, *7*, 1967–1969.

[3] Konishi, H.; Lam, T. Y.; Malerich, J. P.; Rawal, V. H. Enantioselective α-Amination of 1,3-Dicarbonyl Compounds Using Squaramide Derivatives as Hydrogen Bonding Catalysts. *Org. Lett.* **2010**, *12*, 2028–2031.

[4] Miyaji, R.; Asano, K.; Matsubara, S. Bifunctional Organocatalysts for the Enantioselective Synthesis of Axially Chiral Isoquinoline *N*-Oxides. *J. Am. Chem. Soc*. **2015**, *137*, 6766–6769.

[5] Badiola, E.; Fiser, B.; Gómez-Bengoa, E.; Mielgo, A.; Olaizola, I.; Urruzuno, I.; García, J. M.; Odriozola, J. M.; Razkin, J.; Oiarbide, M.; Palomo, C. Enantioselective Construction of Tetrasubstituted Stereogenic Carbons through Brønsted Base Catalyzed Michael Reactions: α′-Hydroxy Enones as Key Enoate Equivalent. *J. Am. Chem. Soc*. **2014**, *136*, 17869–17881.

[6] Hu, Q.; He, Z.; Peng, L.; Guo, C. Combining Nickel and Squaramide Catalysis for the Stereodivergent α-Propargylation of Oxindoles. *Nat. Synth*. **2022**, *1*, 322–331.

9. Copies of ^{1}H NMR and ^{13}C NMR spectra

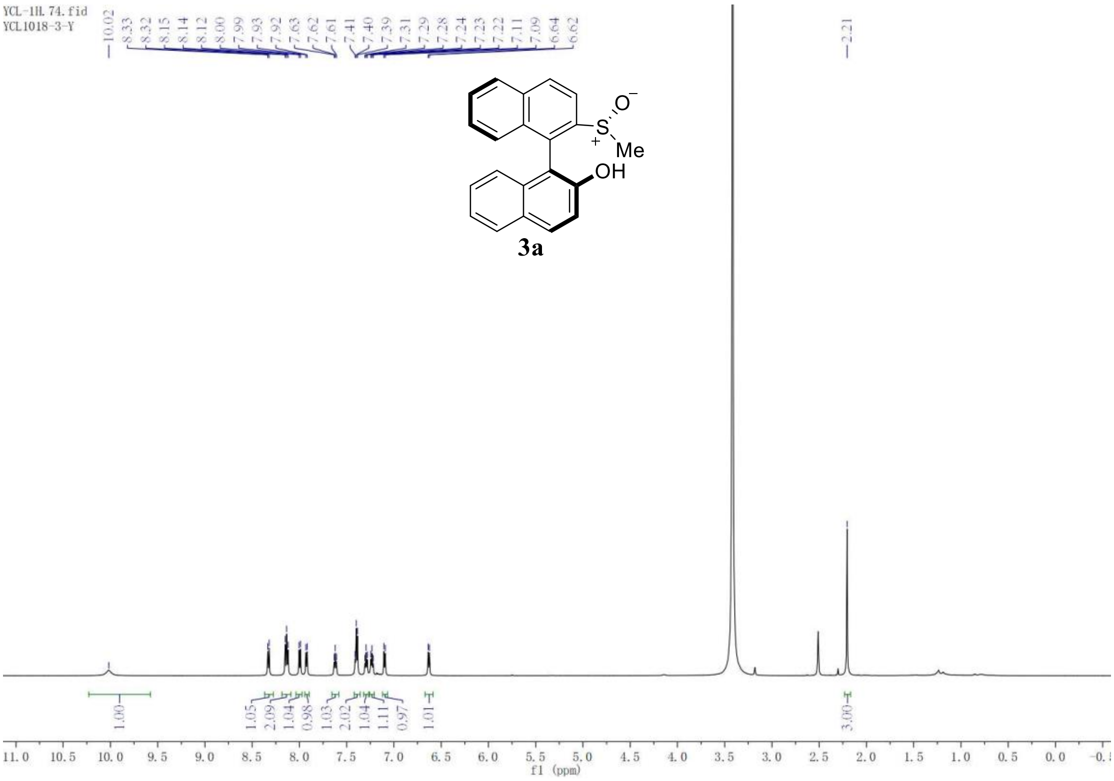

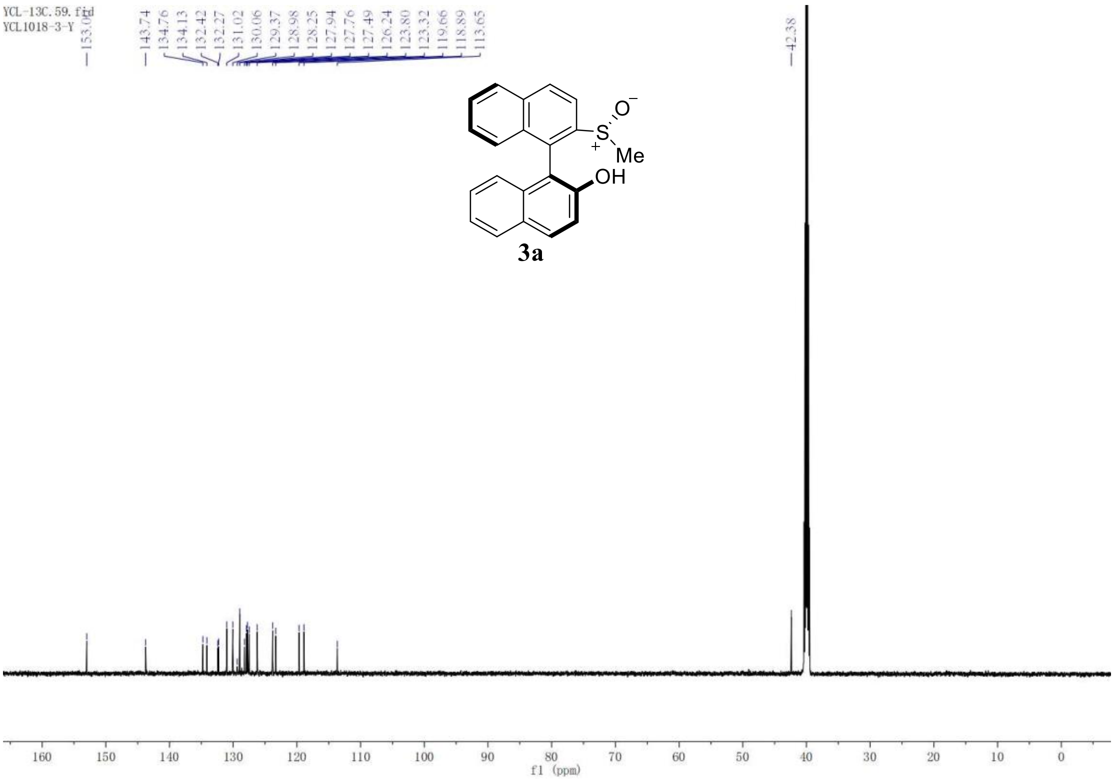

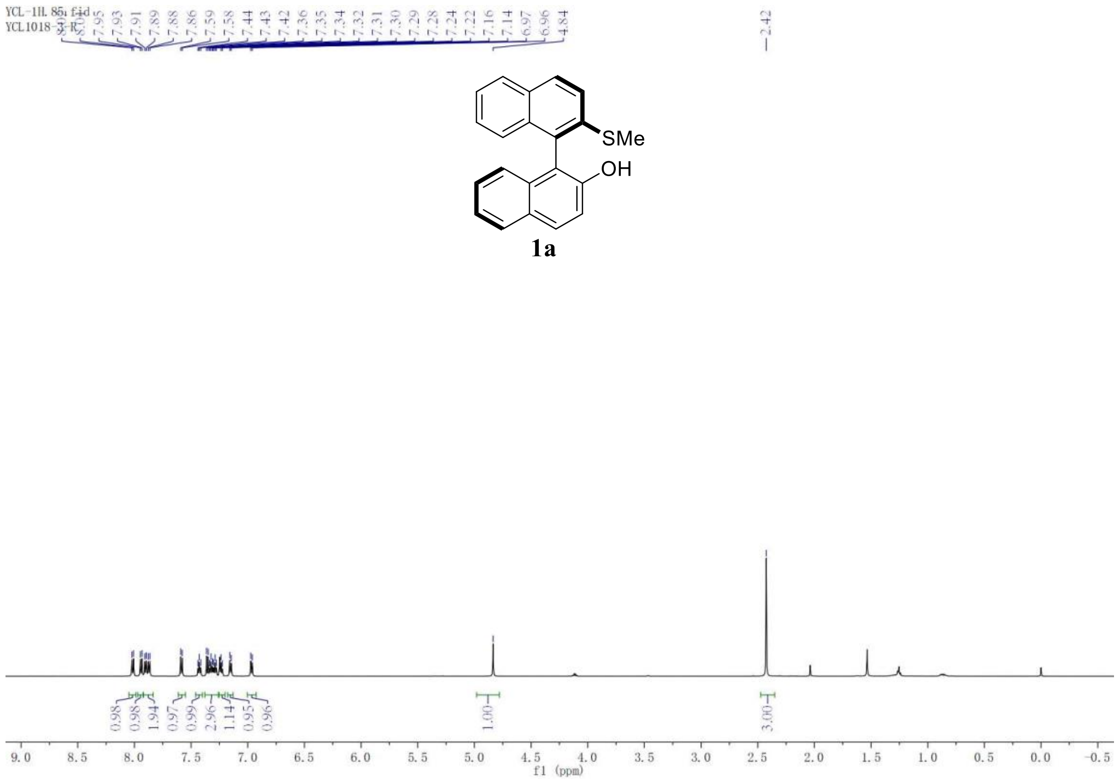

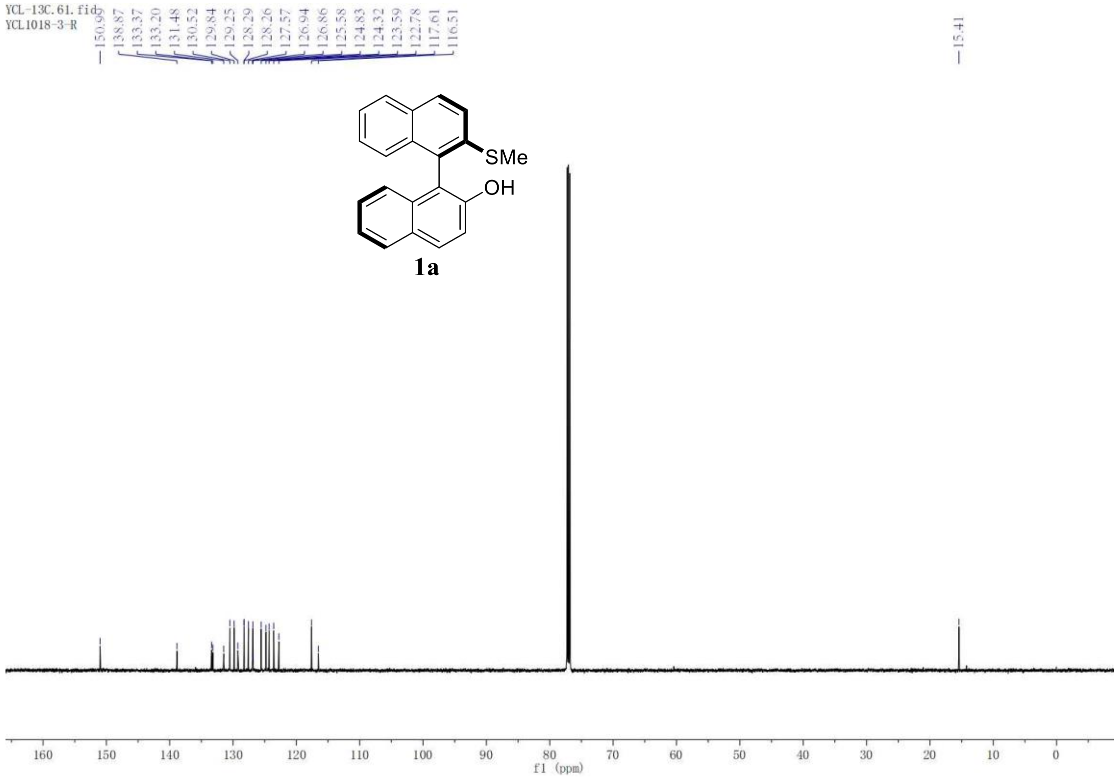

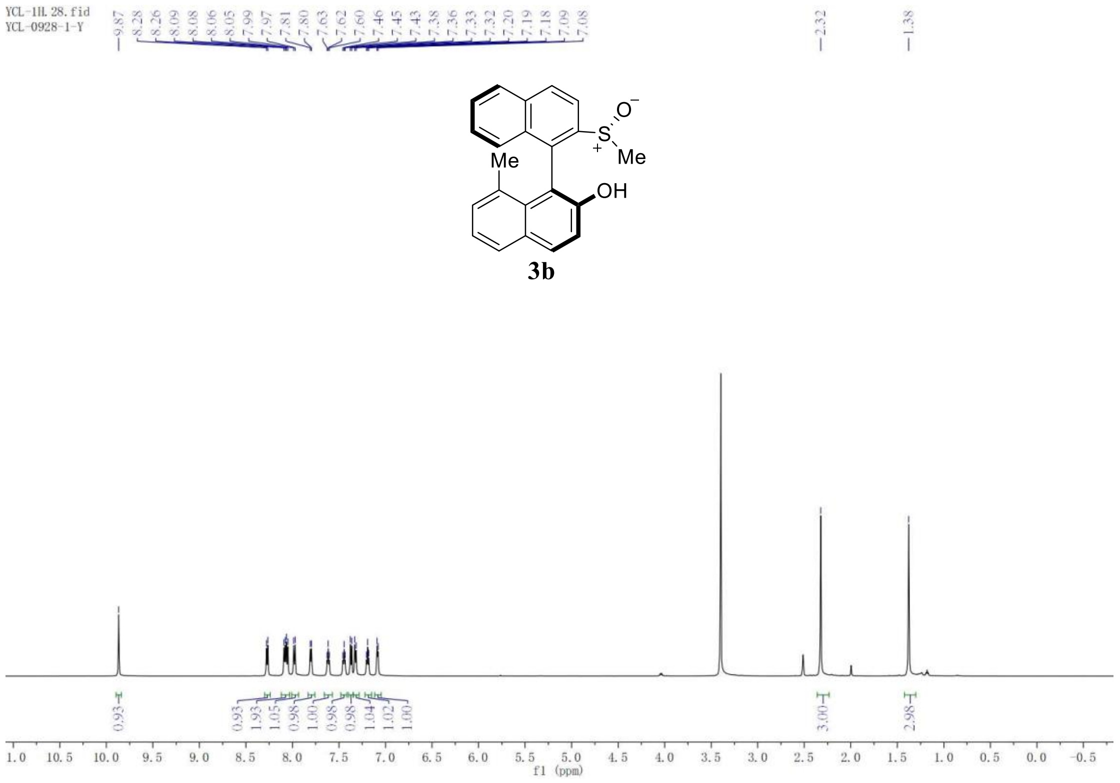
YCL-1H.28.fid
YCL-0928-1-Y
9.87
8.28
8.26
8.09
8.08
8.06
8.05
7.99
7.97
7.81
7.80
7.63
7.62
7.60
7.46
7.45
7.43
7.38
7.36
7.33
7.32
7.20
7.19
7.18
7.09
7.08
2.32
1.38
O
S
+
Me
Me
OH
3b
0.93
0.93
1.93
1.05
0.98
1.00
0.98
0.98
1.04
1.02
1.00
3.00
2.98
f1 (ppm)

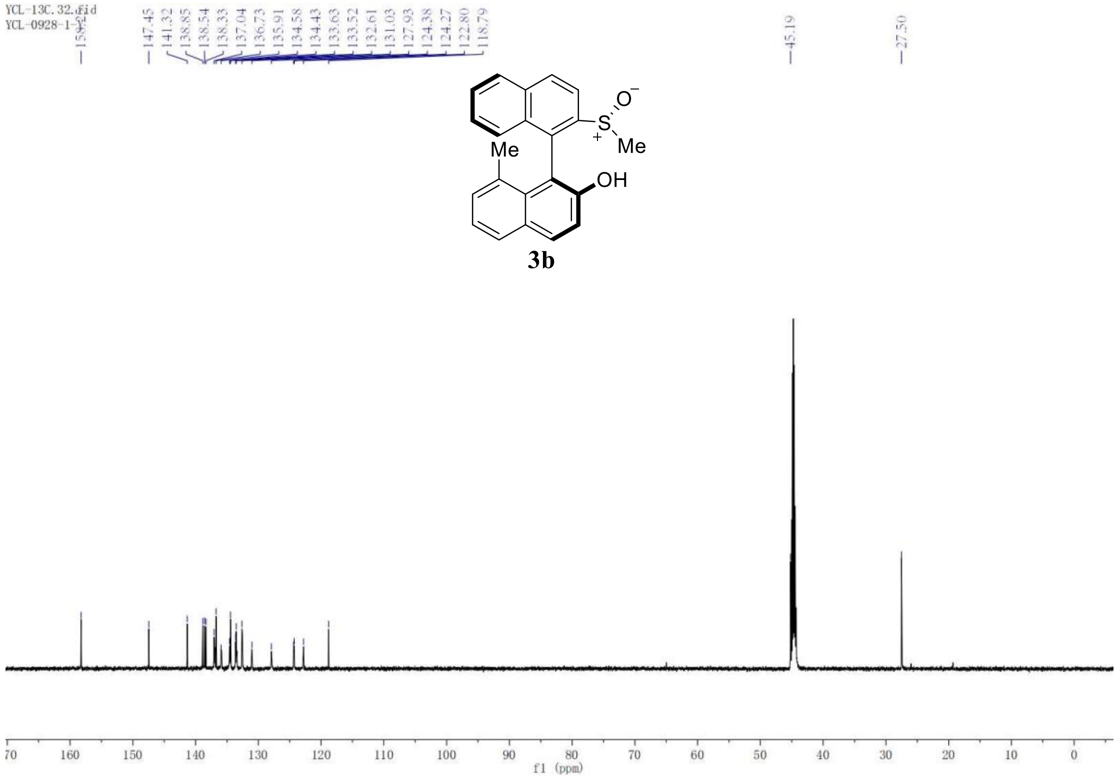
YCL-13C.32.fid
YCL-0928-1-Y
147.45
141.32
138.85
138.54
138.33
137.04
136.73
135.91
134.58
134.43
133.63
133.52
132.61
131.03
127.93
124.38
124.27
122.80
118.79
45.19
27.50
O
S
+
Me
Me
OH
3b
f1 (ppm)

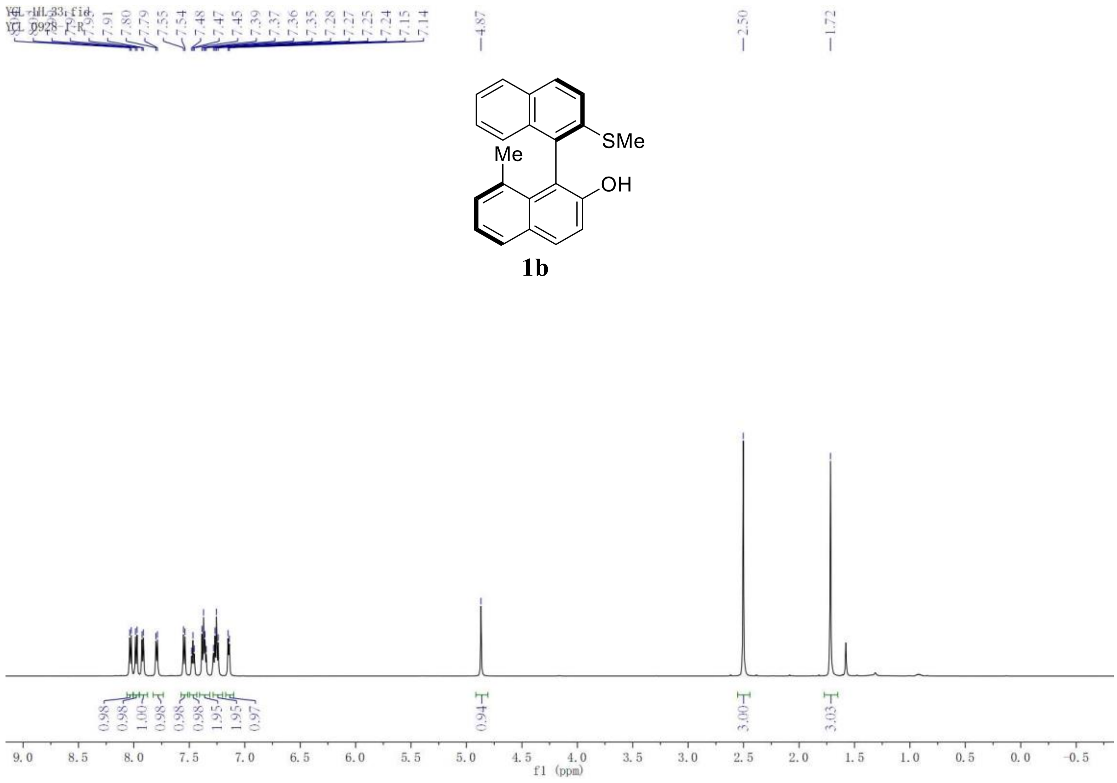
SMe
Me
OH
1b
4.87
2.50
1.72
0.94
3.00
3.03
f1 (ppm)

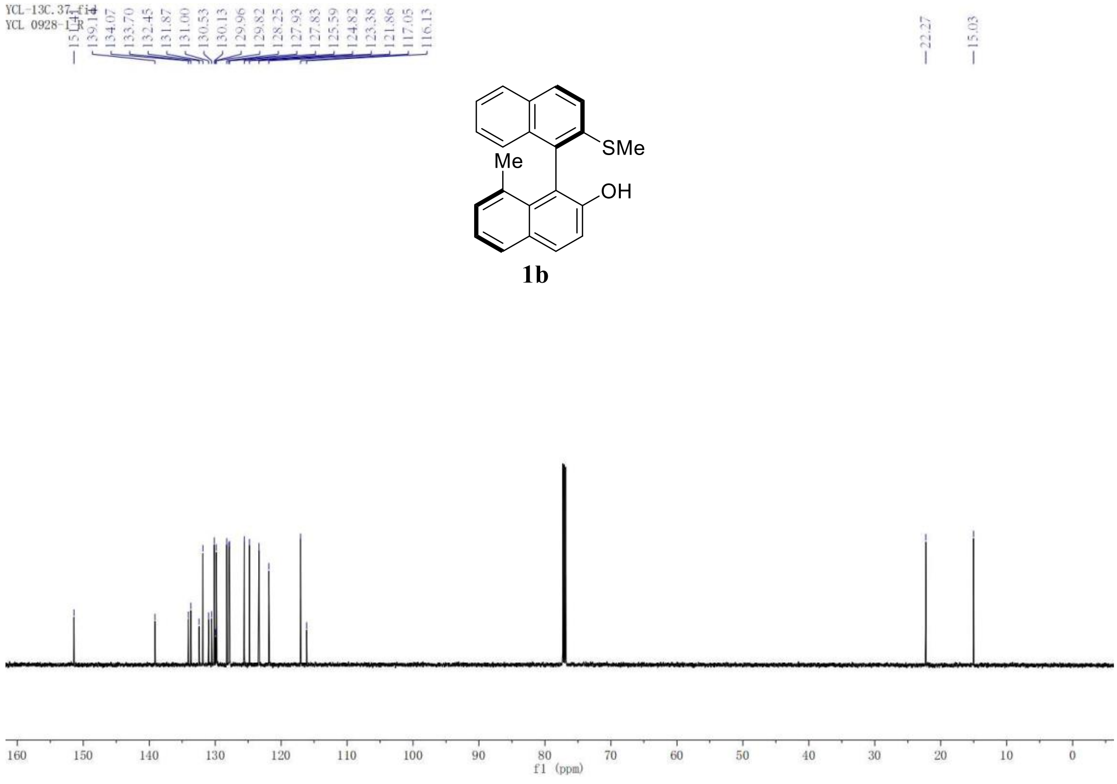
SMe
Me
OH
1b
22.27
15.03
f1 (ppm)

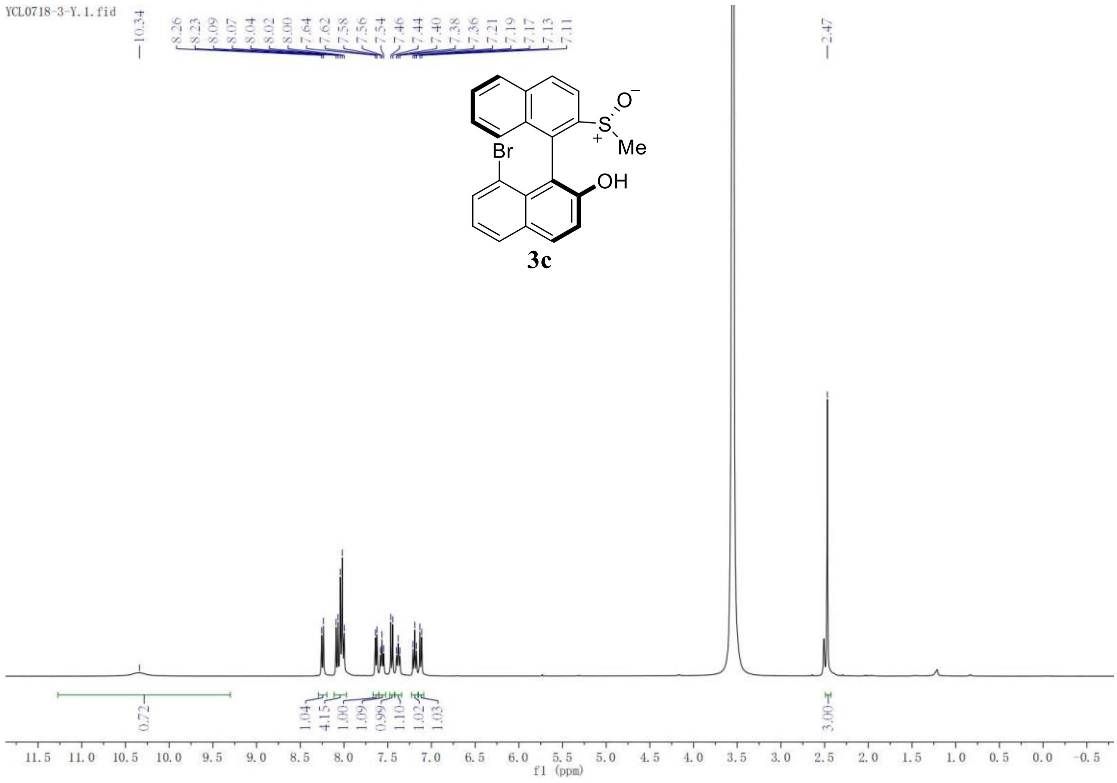
YCL0718-3-Y.1.fid
10.34
8.26 8.23 8.09 8.07 8.04 8.02 8.00 7.64 7.62 7.58 7.56 7.54 7.46 7.44 7.40 7.38 7.36 7.21 7.19 7.17 7.13 7.11
2.47
Br
OH
Me
3c
0.72
1.04 4.15 1.00 1.09 0.99 1.10 1.02 1.03
3.00
f1 (ppm)

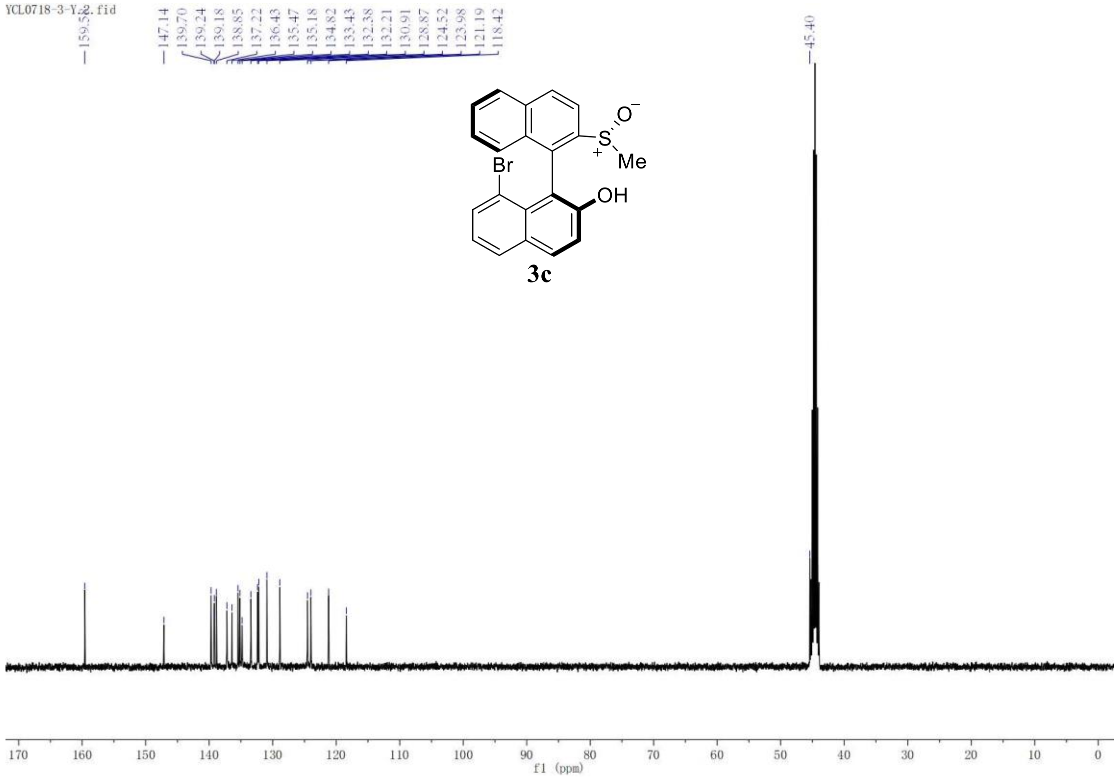
YCL0718-3-Y.2.fid
147.14
139.70 139.24 139.18 138.85 137.22 136.43 135.47 135.18 134.82 133.43 132.38 132.21 130.91 128.87 124.52 123.98 121.19 118.42
45.40
Br
OH
Me
3c
f1 (ppm)

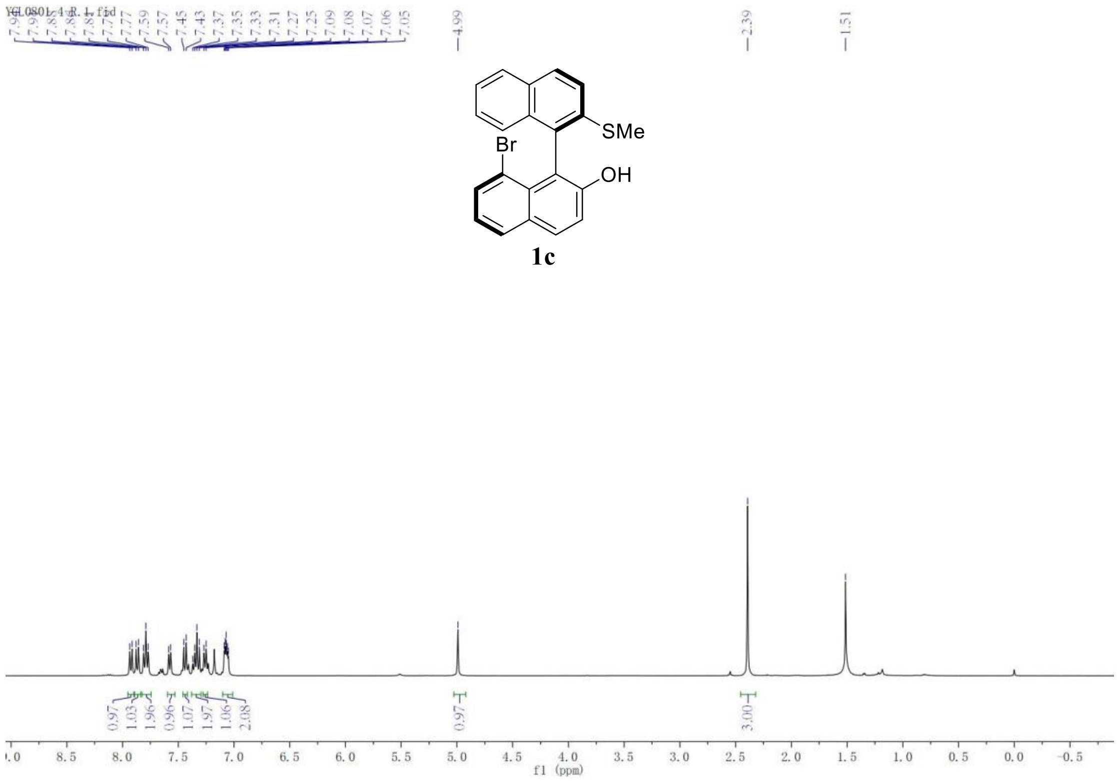
YCL0801-4-R.1.fid
4.99
2.39
1.51
SMe
Br
OH
1c
0.97
1.03
1.96
0.96
1.07
1.97
1.06
2.08
0.97
3.00
f1 (ppm)

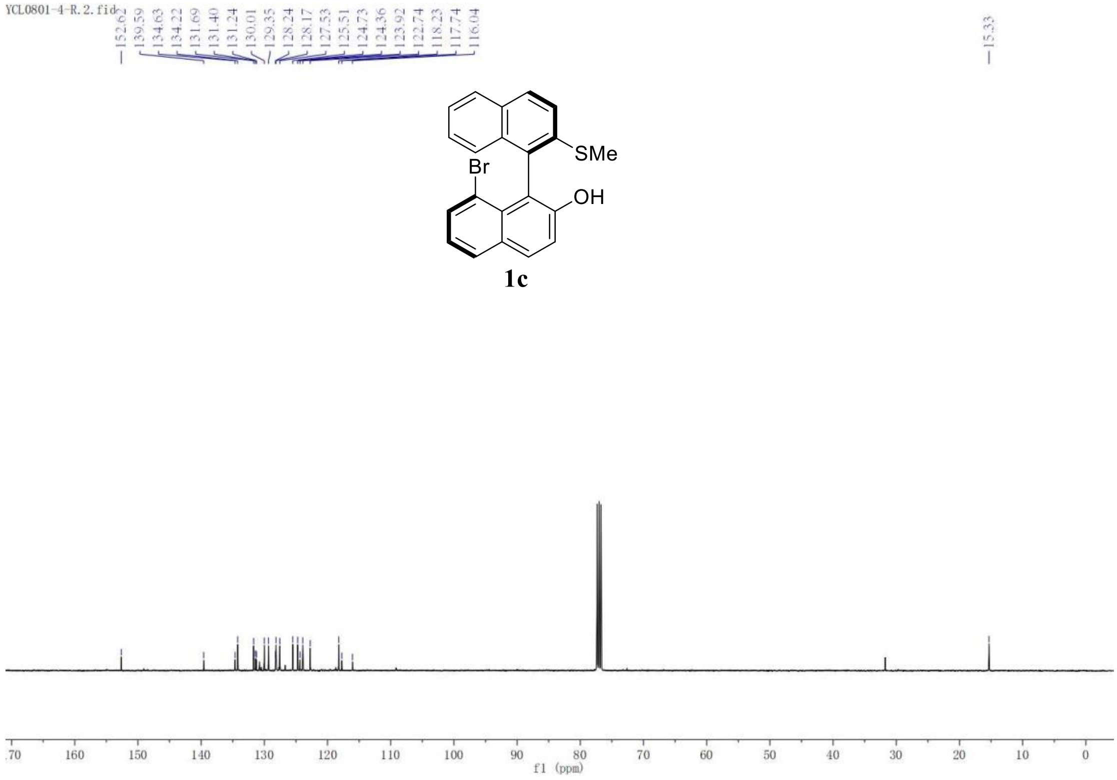
YCL0801-4-R.2.fid
152.62
139.59
134.63
134.22
131.69
131.40
131.24
130.01
129.35
128.24
128.17
127.53
125.51
124.73
124.36
123.92
122.74
118.23
117.74
116.04
15.33
SMe
Br
OH
1c
f1 (ppm)

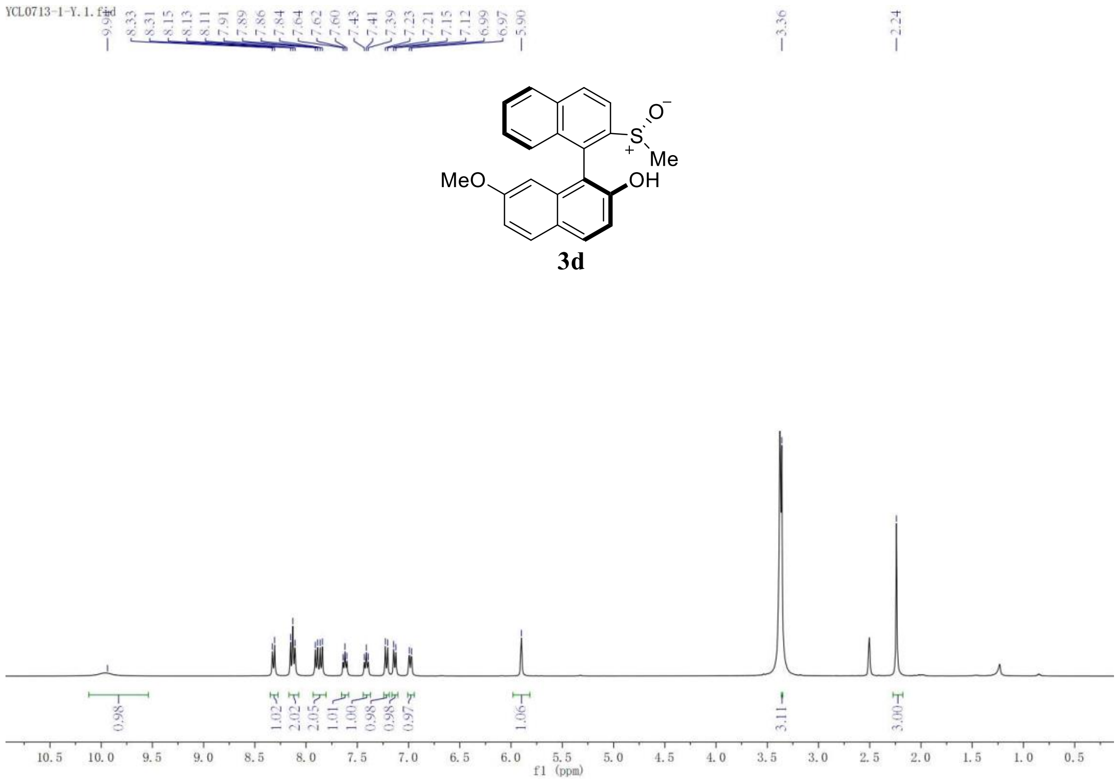
YCL0713-1-Y.1.fid
8.33
8.31
8.15
8.13
8.11
7.91
7.89
7.86
7.84
7.64
7.62
7.60
7.43
7.41
7.39
7.23
7.21
7.15
7.12
6.99
6.97
5.90
3.36
2.24
O
S
+
Me
MeO
OH
3d
0.98
1.02
2.02
2.05
1.01
1.00
0.98
0.98
0.97
1.06
3.11
3.00
f1 (ppm)

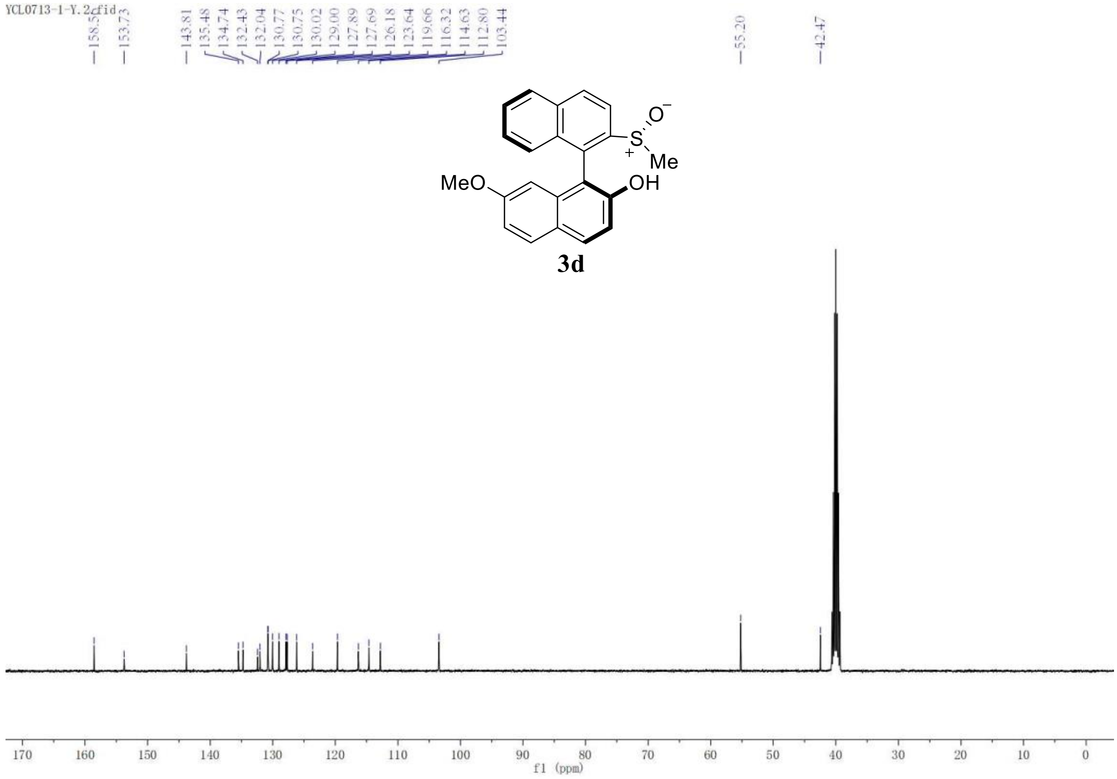
YCL0713-1-Y.2.fid
153.73
143.81
135.48
134.74
132.43
132.04
130.77
130.75
130.02
129.00
127.89
127.69
126.18
123.64
119.66
116.32
114.63
112.80
103.44
55.20
42.47
O
S
+
Me
MeO
OH
3d
f1 (ppm)

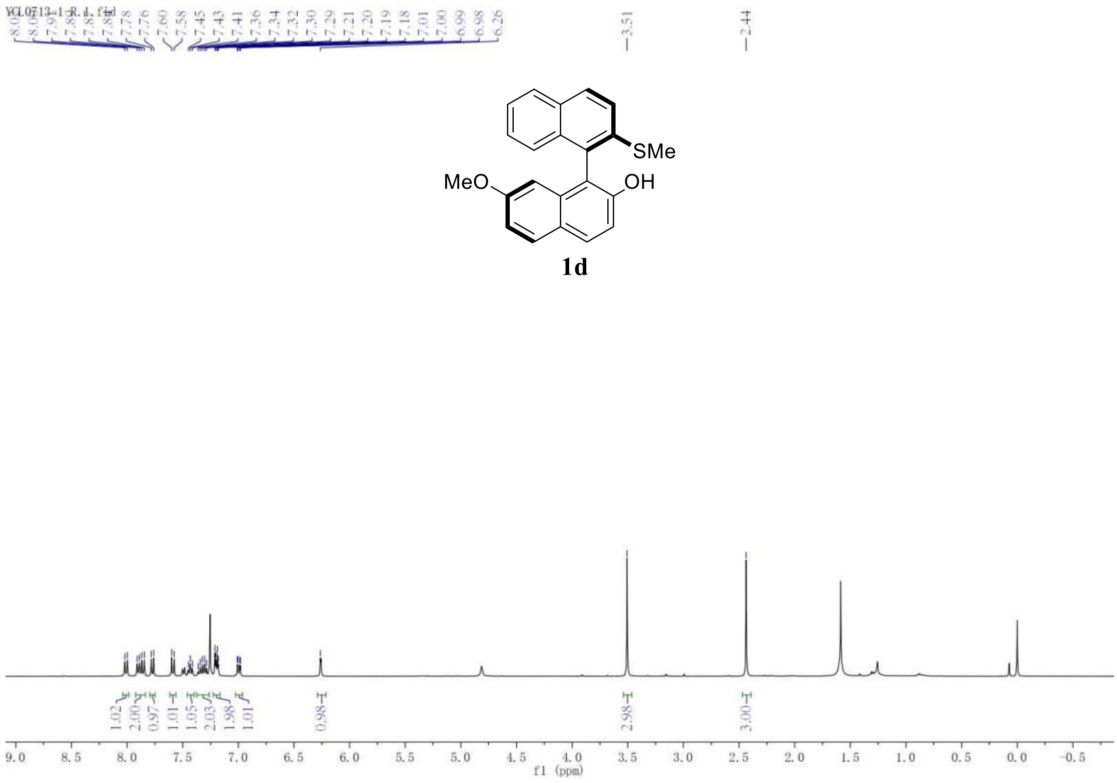
YCL0713-1-R.1.fid
7.78 7.76 7.60 7.58 7.45 7.43 7.41 7.36 7.34 7.32 7.30 7.29 7.21 7.20 7.19 7.18 7.01 7.00 6.99 6.98 6.26
3.51
2.44
SMe
MeO
OH
1d
1.02 2.00 0.97 1.01 1.05 2.03 1.98 1.01 0.98 2.98 3.00
9.0 8.5 8.0 7.5 7.0 6.5 6.0 5.5 5.0 4.5 4.0 3.5 3.0 2.5 2.0 1.5 1.0 0.5 0.0 -0.5
f1 (ppm)

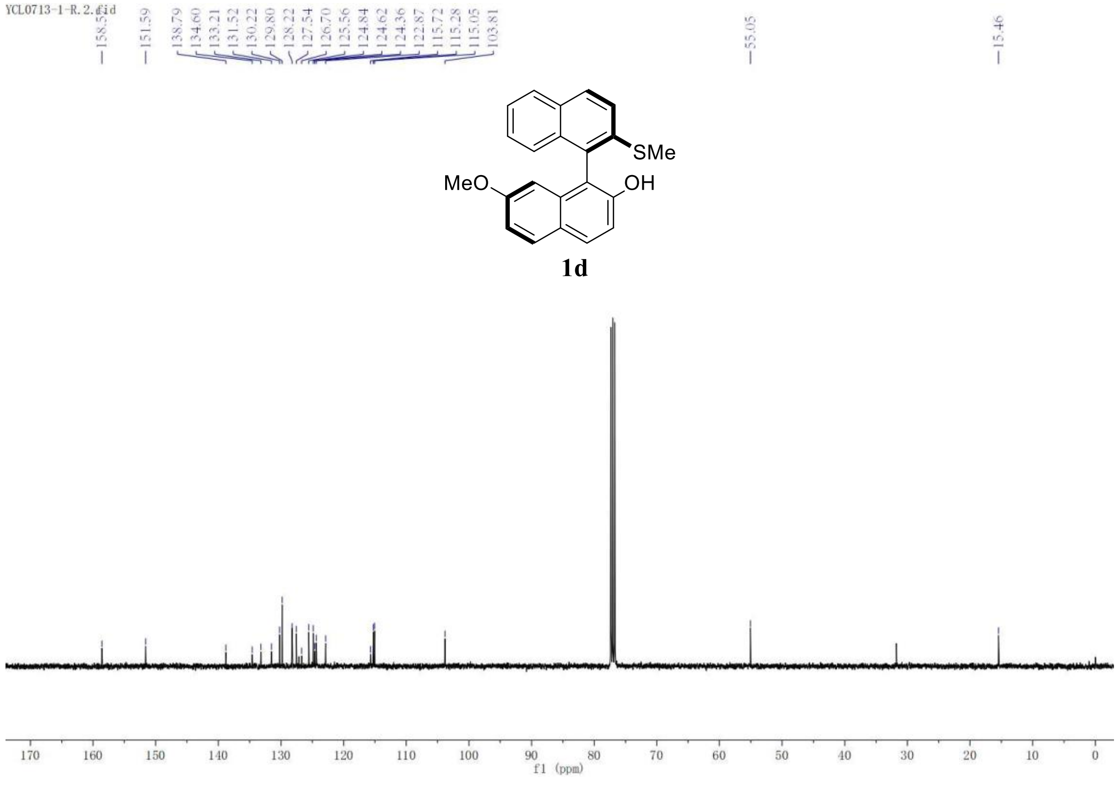
YCL0713-1-R.2.fid
151.59
138.79 134.60 133.21 131.52 130.22 129.80 128.22 127.54 126.70 125.56 124.84 124.62 124.36 122.87 115.72 115.28 115.05 103.81
55.05
15.46
SMe
MeO
OH
1d
170 160 150 140 130 120 110 100 90 80 70 60 50 40 30 20 10 0
f1 (ppm)

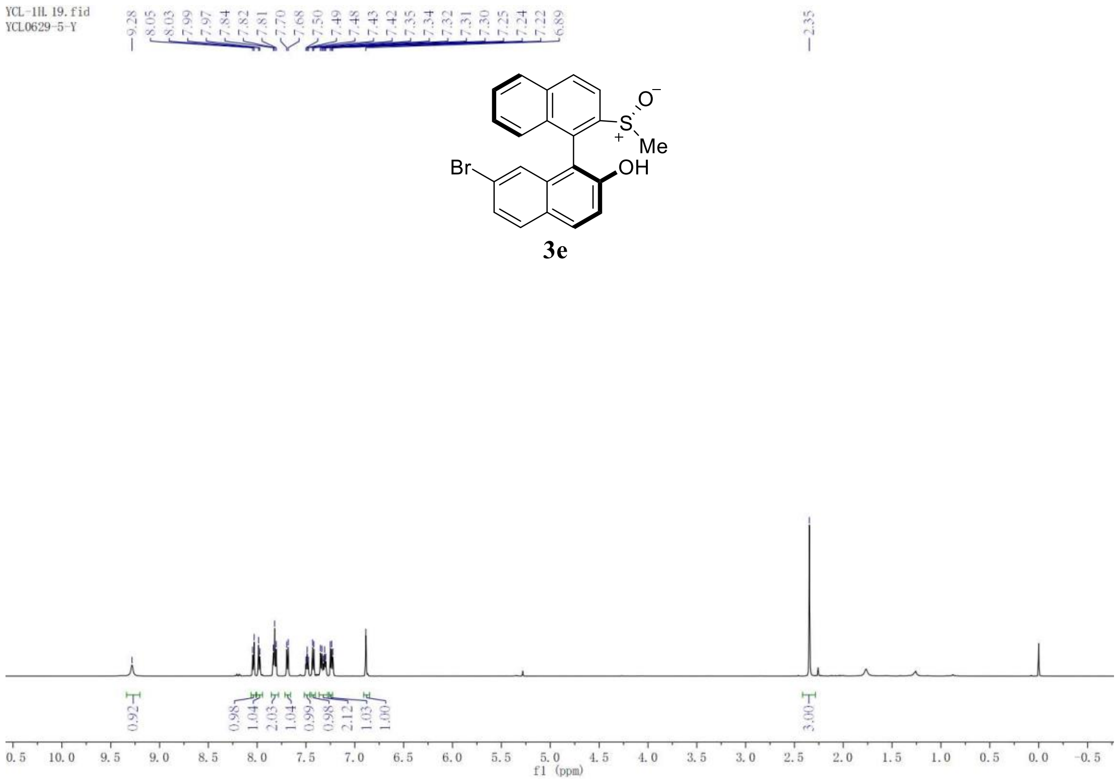
YCL-1H.19.fid
YCL0629-5-Y
9.28 8.05 8.03 7.99 7.97 7.84 7.82 7.81 7.70 7.68 7.50 7.49 7.48 7.43 7.42 7.35 7.34 7.32 7.31 7.30 7.25 7.24 7.22 6.89
2.35
Br
OH
S
O
Me
3e
0.92 0.98 1.04 2.03 1.04 0.99 0.98 2.12 1.03 1.00
3.00
f1 (ppm)

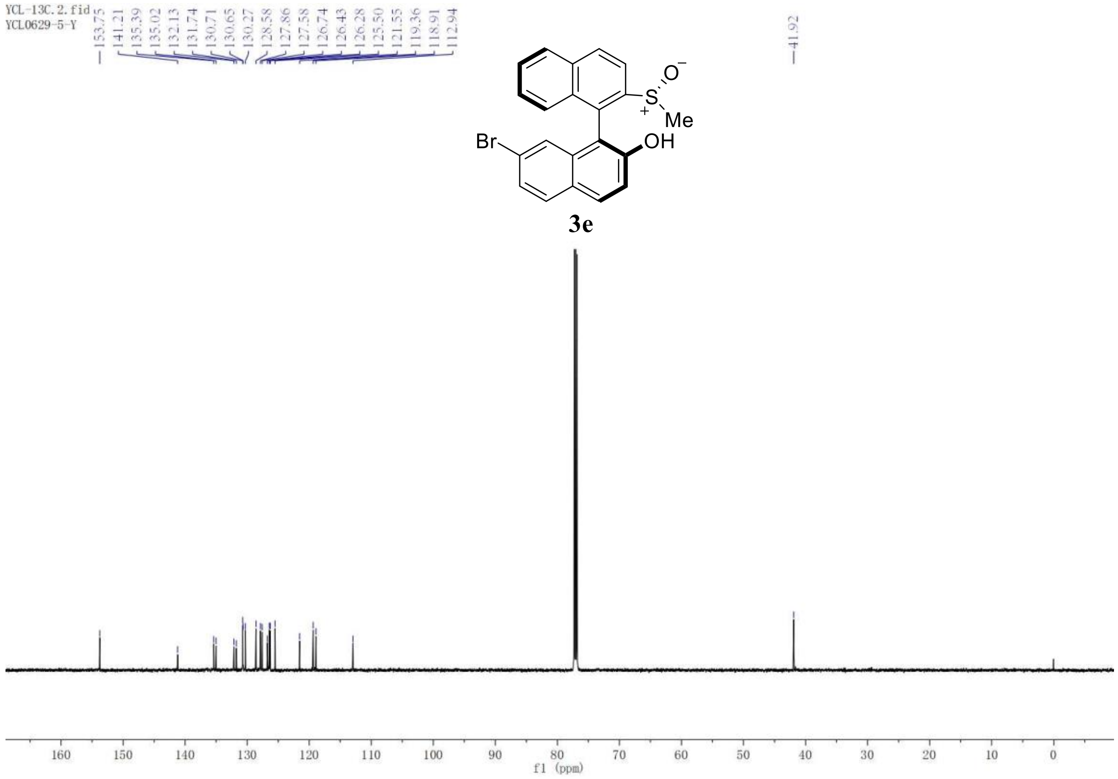
YCL-13C.2.fid
YCL0629-5-Y
153.75 141.21 135.39 135.02 132.13 131.74 130.71 130.65 130.27 128.58 127.86 127.58 126.74 126.43 126.28 125.50 121.55 119.36 118.91 112.94
41.92
Br
OH
S
O
Me
3e
f1 (ppm)

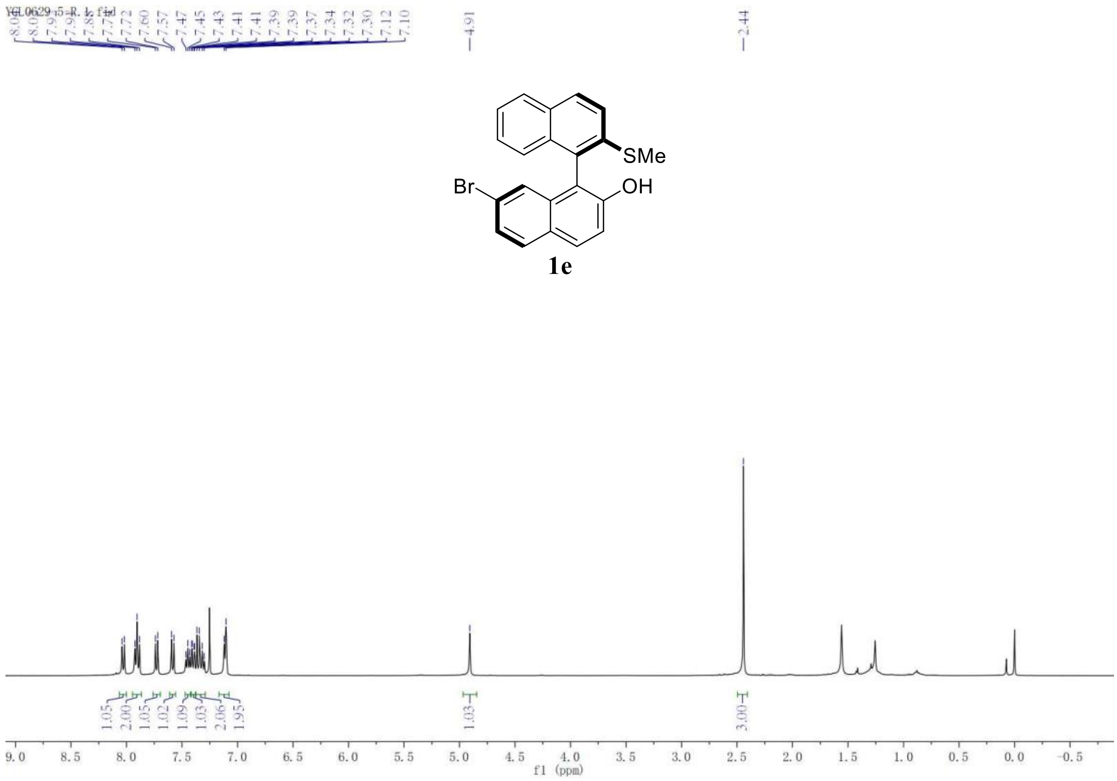
YCL0629-5-R.1.fid
7.60
7.57
7.47
7.45
7.43
7.41
7.39
7.39
7.37
7.34
7.32
7.30
7.12
7.10
4.91
2.44
SMe
OH
Br
1e
1.05
2.00
1.05
1.02
1.09
1.03
2.06
1.95
1.03
3.00
f1 (ppm)

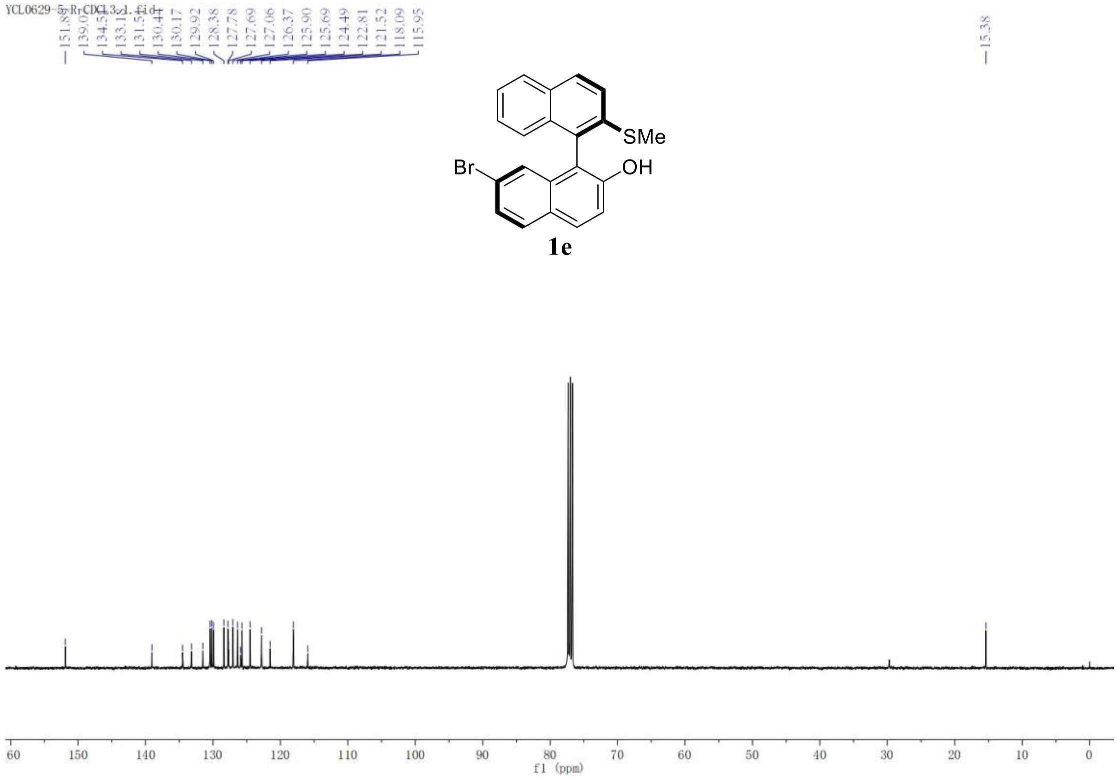
130.17
129.92
128.38
127.78
127.69
127.06
126.37
125.90
125.69
124.49
122.81
121.52
118.09
115.95
15.38
SMe
OH
Br
1e
f1 (ppm)

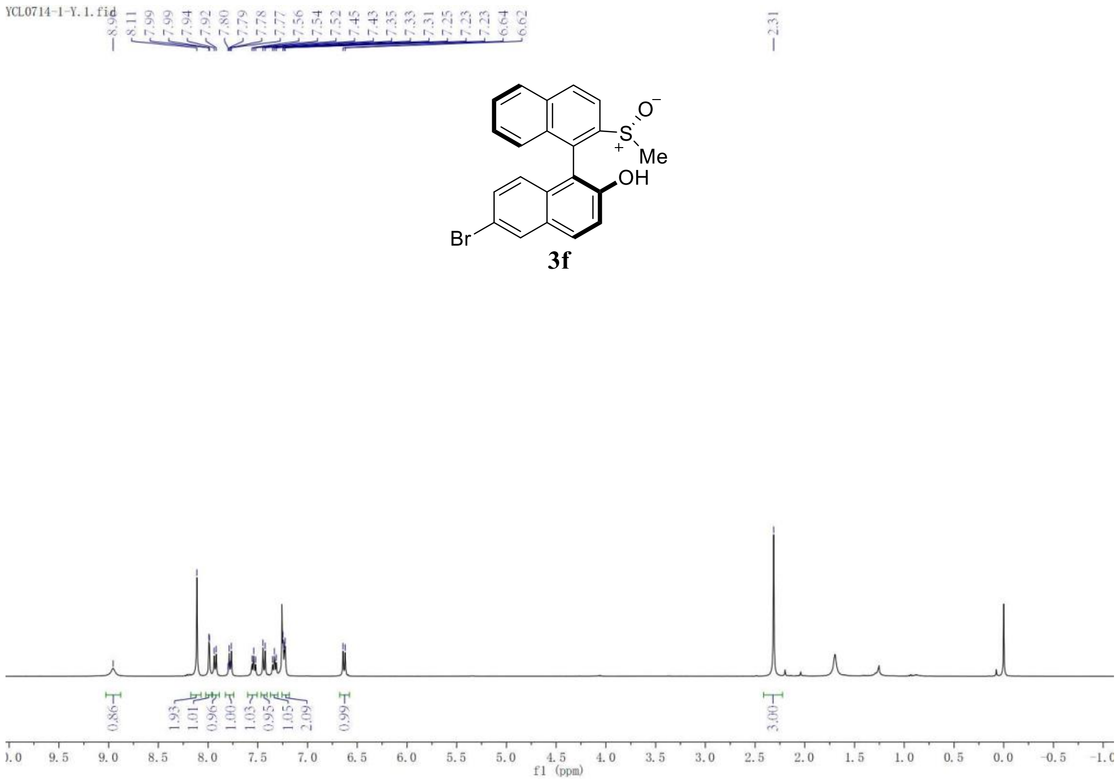
YCL0714-1-Y.1.fid
8.94 8.11 7.99 7.99 7.94 7.92 7.80 7.79 7.78 7.77 7.56 7.54 7.52 7.45 7.43 7.35 7.33 7.31 7.25 7.23 7.23 6.64 6.62
2.31
Br
OH
S
O
Me
3f
0.86 1.93 1.01 0.96 1.00 1.03 0.95 1.05 2.09 0.99 3.00
f1 (ppm)

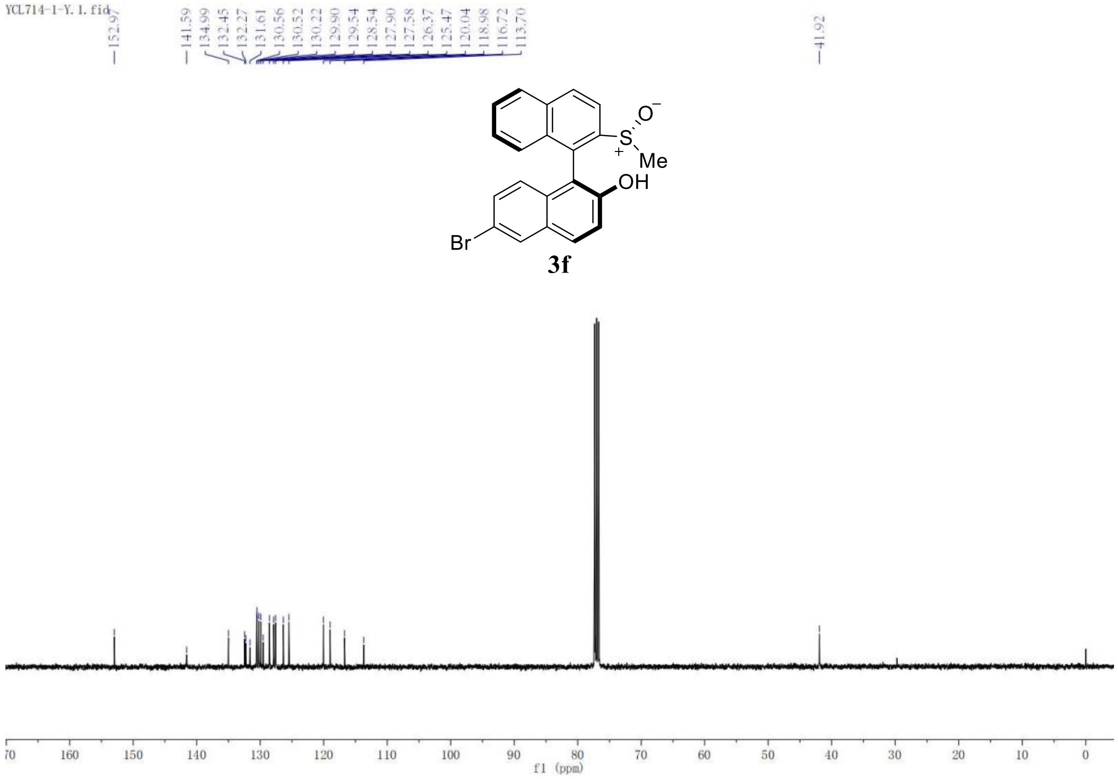
YCL714-1-Y.1.fid
152.97 141.59 134.99 132.45 132.27 131.61 130.56 130.52 130.22 129.90 129.54 128.54 127.90 127.58 126.37 125.47 120.04 118.98 116.72 113.70
41.92
Br
OH
S
O
Me
3f
f1 (ppm)

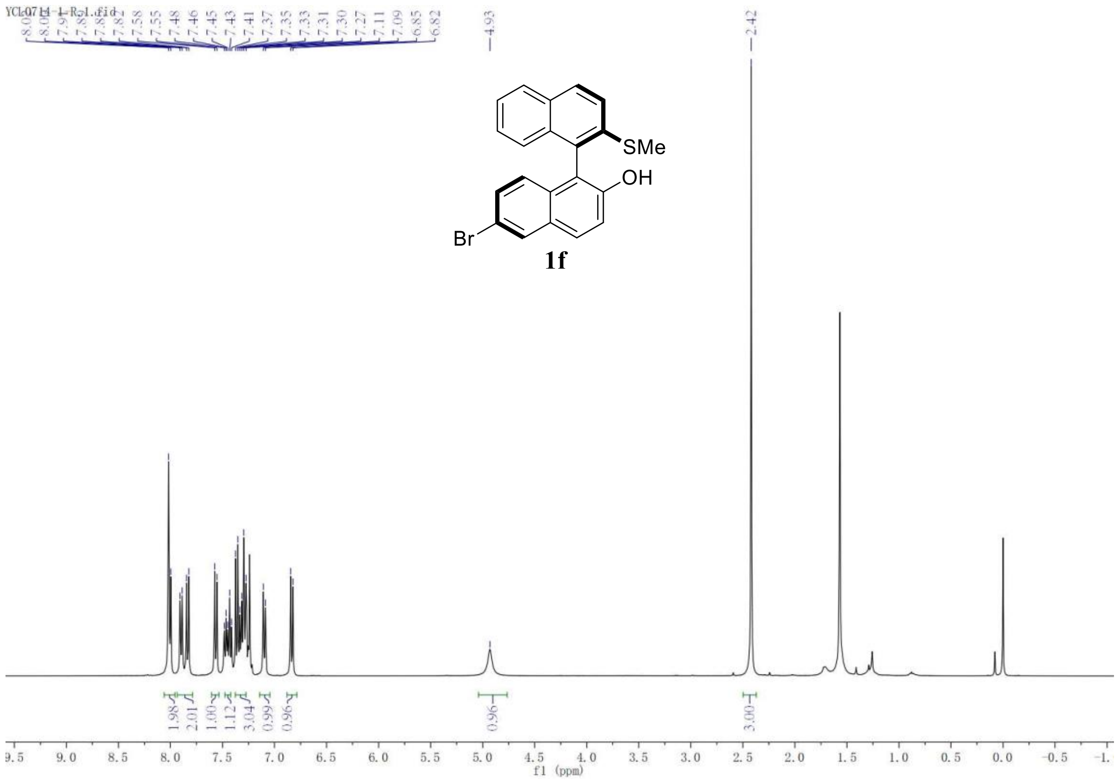
YCL0714-1-R.1.fid
7.58
7.55
7.48
7.46
7.45
7.43
7.41
7.37
7.35
7.33
7.31
7.30
7.27
7.11
7.09
6.85
6.82
4.93
2.42
SMe
OH
Br
1f
1.98
2.01
1.00
1.12
3.04
0.99
0.96
0.96
3.00
f1 (ppm)

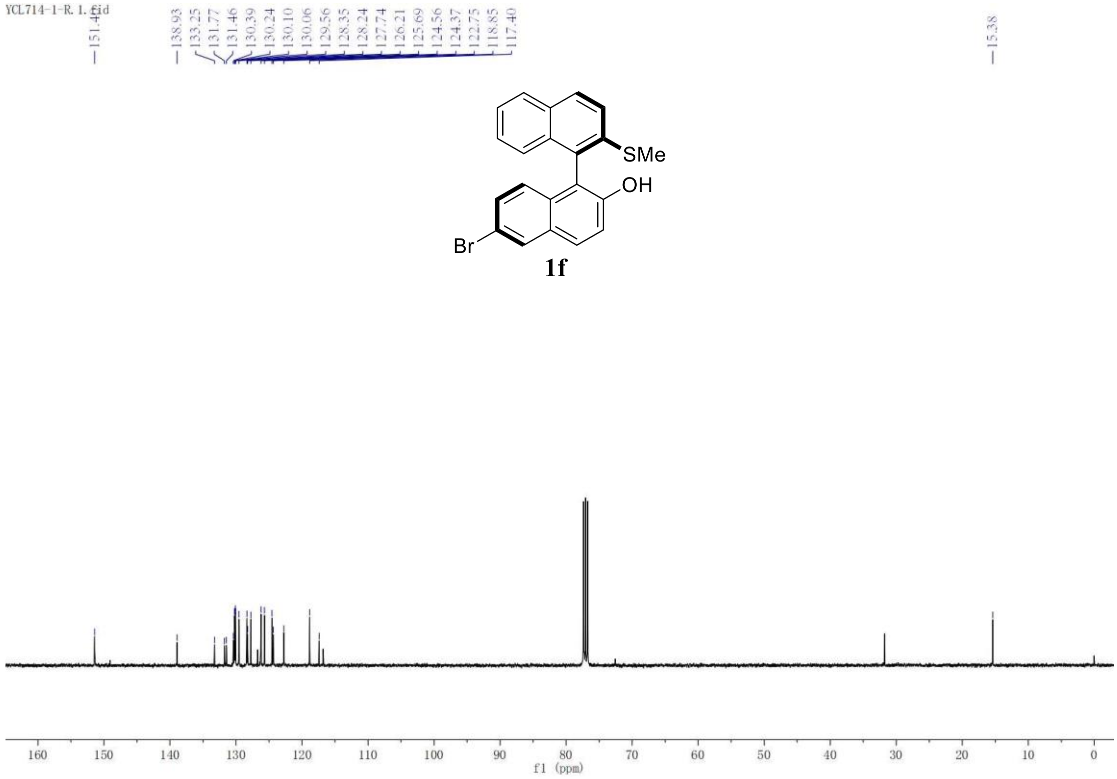
YCL714-1-R.1.fid
138.93
133.25
131.77
131.46
130.39
130.24
130.10
130.06
129.56
128.35
128.24
127.74
126.21
125.69
124.56
124.37
122.75
118.85
117.40
15.38
SMe
OH
Br
1f
f1 (ppm)

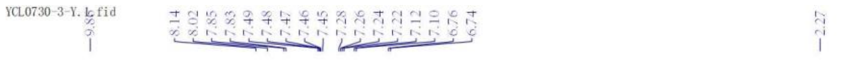
YCL0730-3-Y.1.fid
8.14 8.02 7.85 7.83 7.49 7.48 7.47 7.46 7.45 7.28 7.26 7.24 7.22 7.12 7.10 6.76 6.74
2.27

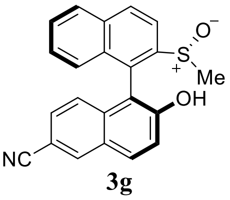
O
S
+
Me
OH
NC

3g

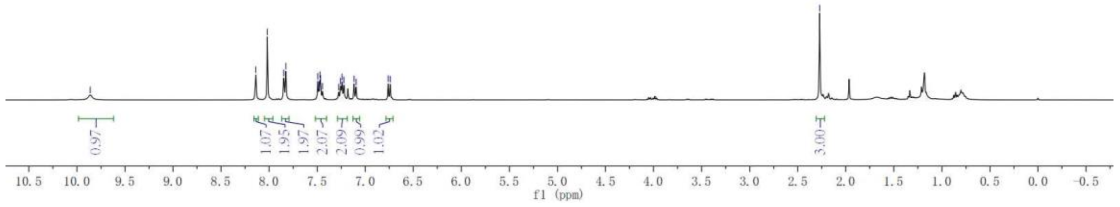
0.97
1.07
1.95
1.97
2.07
2.09
0.99
1.02
3.00
10.5 10.0 9.5 9.0 8.5 8.0 7.5 7.0 6.5 6.0 5.5 5.0 4.5 4.0 3.5 3.0 2.5 2.0 1.5 1.0 0.5 0.0 -0.5
f1 (ppm)

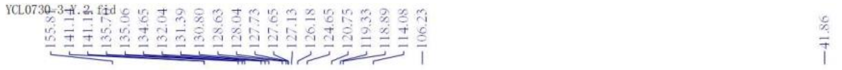
135.06 134.65 132.04 131.39 130.80 128.63 128.04 127.73 127.65 127.13 126.18 124.65 120.75 119.33 118.89 114.08 106.23
41.86

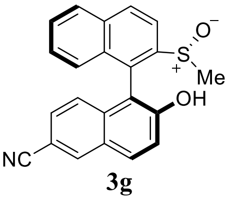
O
S
+
Me
OH
NC

3g

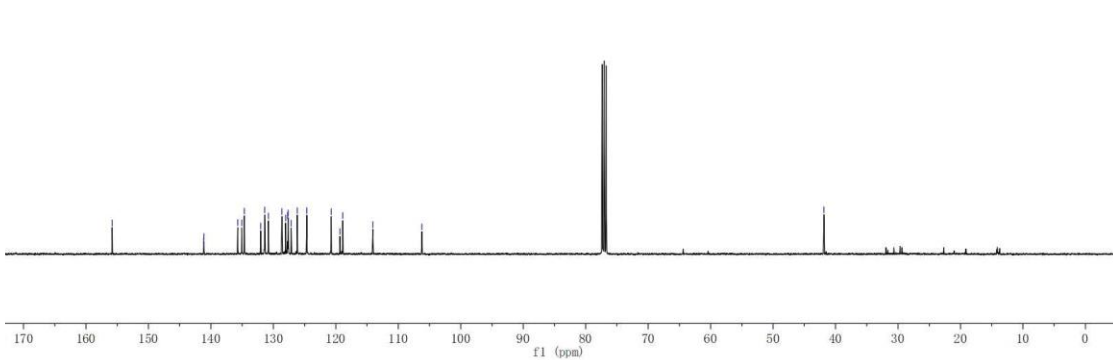
170 160 150 140 130 120 110 100 90 80 70 60 50 40 30 20 10 0
f1 (ppm)

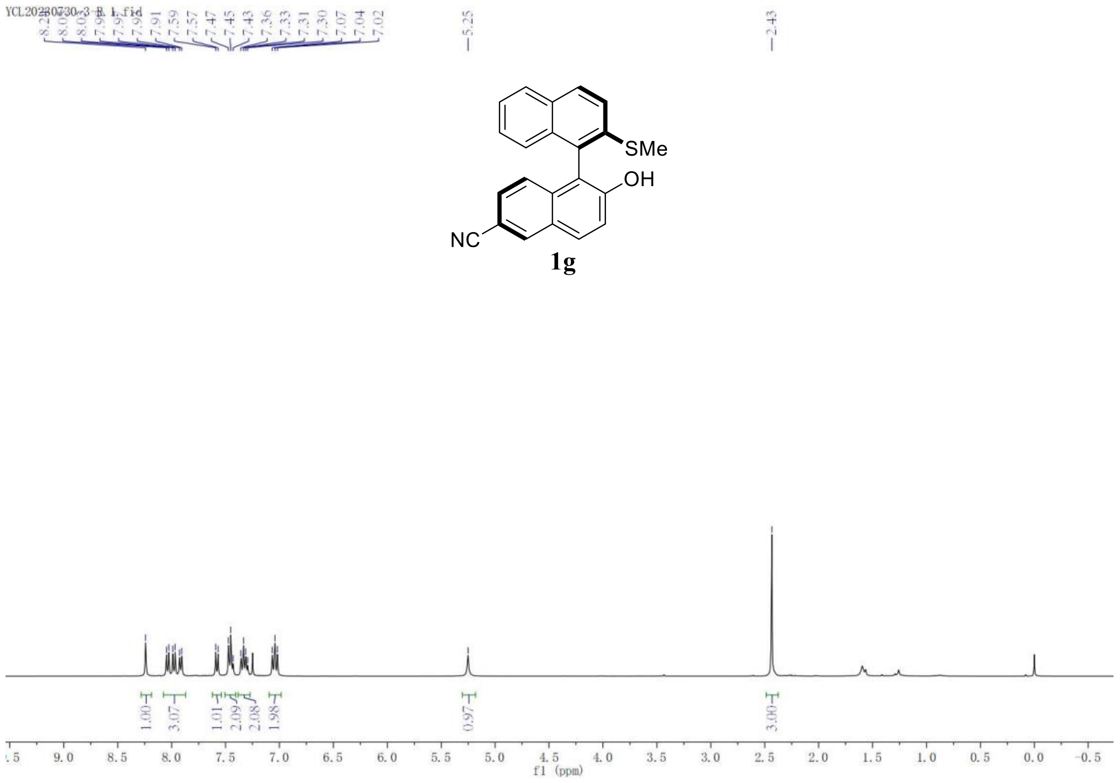
YCL20230730-3-R 1.fid
5.25
2.43
SMe
OH
NC
1g
1.00
3.07
1.01
2.09
2.08
1.98
0.97
3.00
f1 (ppm)

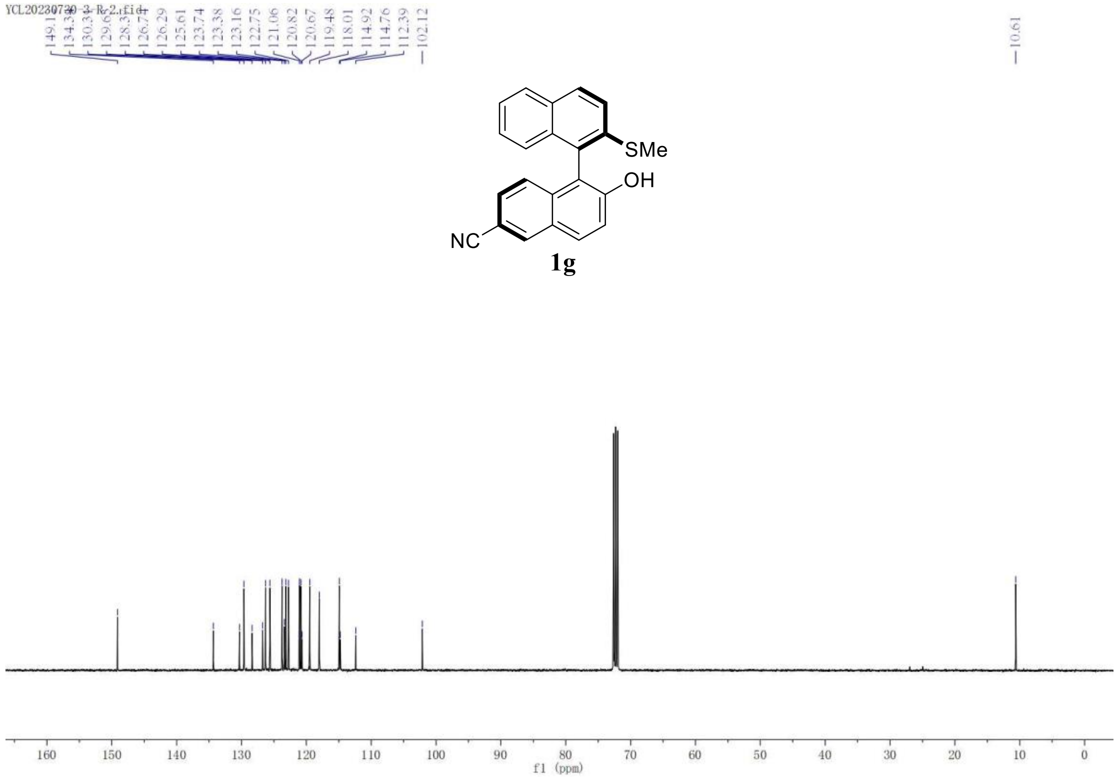
YCL20230730-3-R 2.fid
126.29
125.61
123.74
123.38
123.16
122.75
121.06
120.82
120.67
119.48
118.01
114.92
114.76
112.39
102.12
10.61
SMe
OH
NC
1g
f1 (ppm)

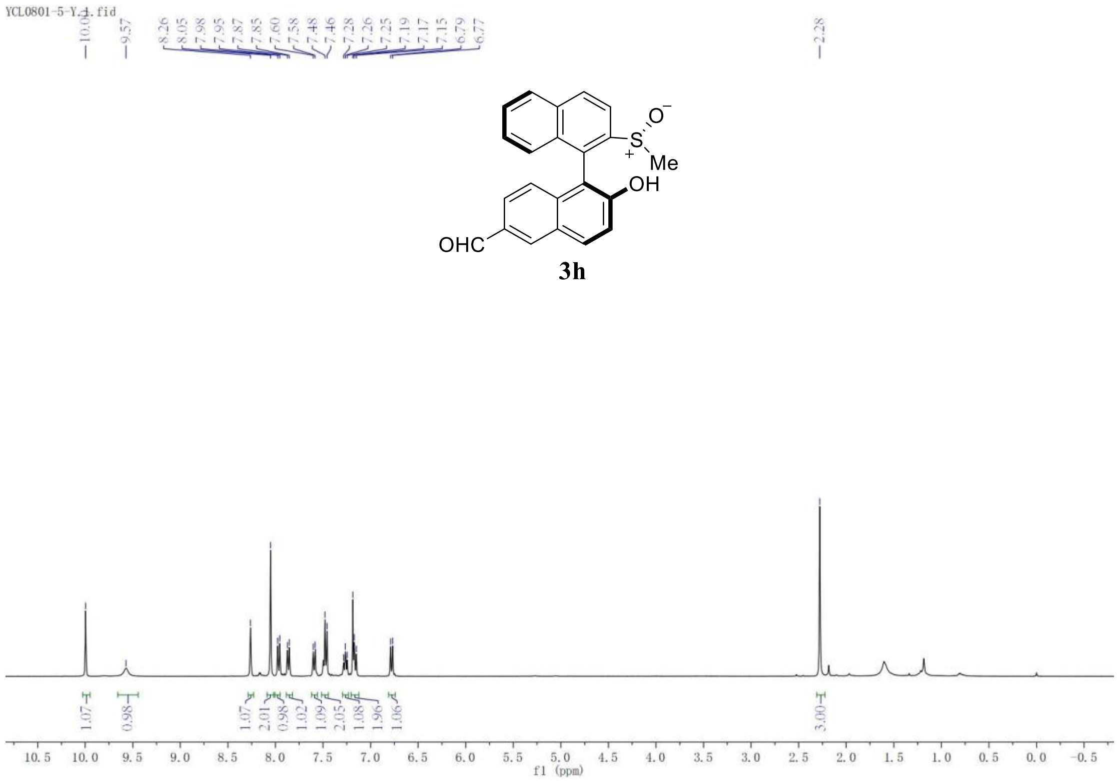
YCL0801-5-Y.1.fid
10.0
9.57
8.26
8.05
7.98
7.95
7.87
7.85
7.60
7.58
7.48
7.46
7.28
7.26
7.25
7.19
7.17
7.15
6.79
6.77
2.28
O
S
+
Me
OH
OHC
3h
1.07
0.98
1.07
2.01
0.98
1.02
1.09
2.05
1.08
1.96
1.06
3.00
f1 (ppm)

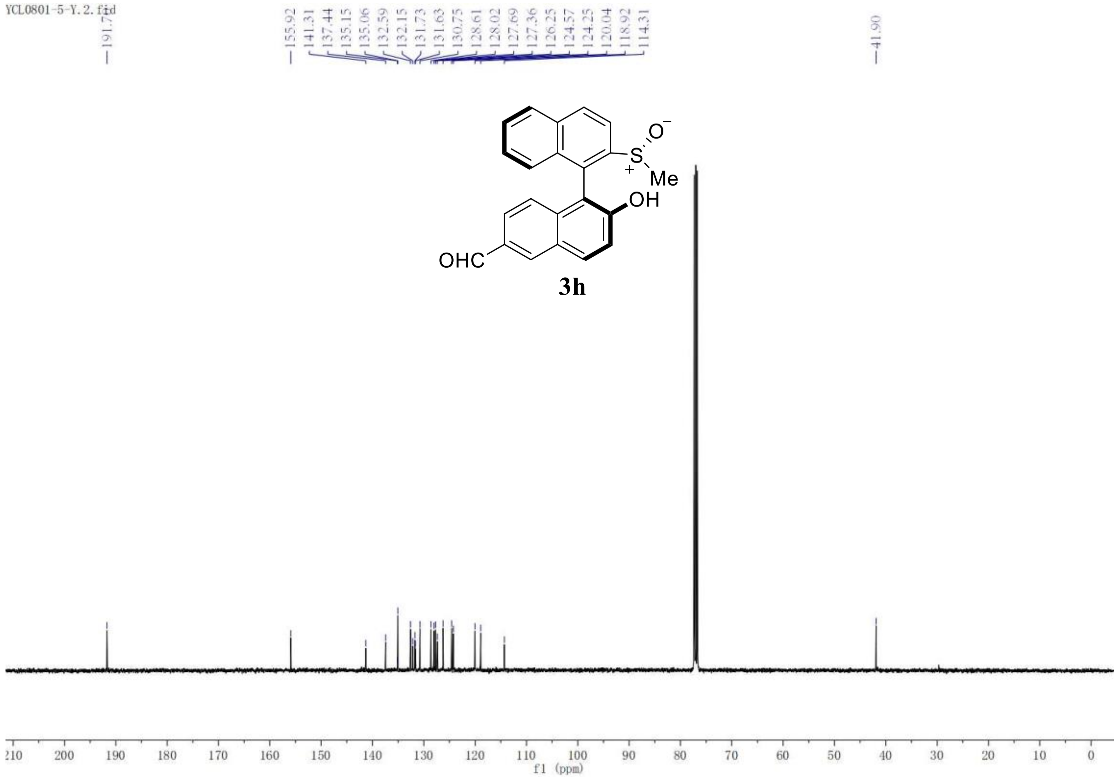
YCL0801-5-Y.2.fid
191.7
155.92
141.31
137.44
135.15
135.06
132.59
132.15
131.73
131.63
130.75
128.61
128.02
127.69
127.36
126.25
124.57
124.25
120.04
118.92
114.31
41.90
O
S
+
Me
OH
OHC
3h
f1 (ppm)

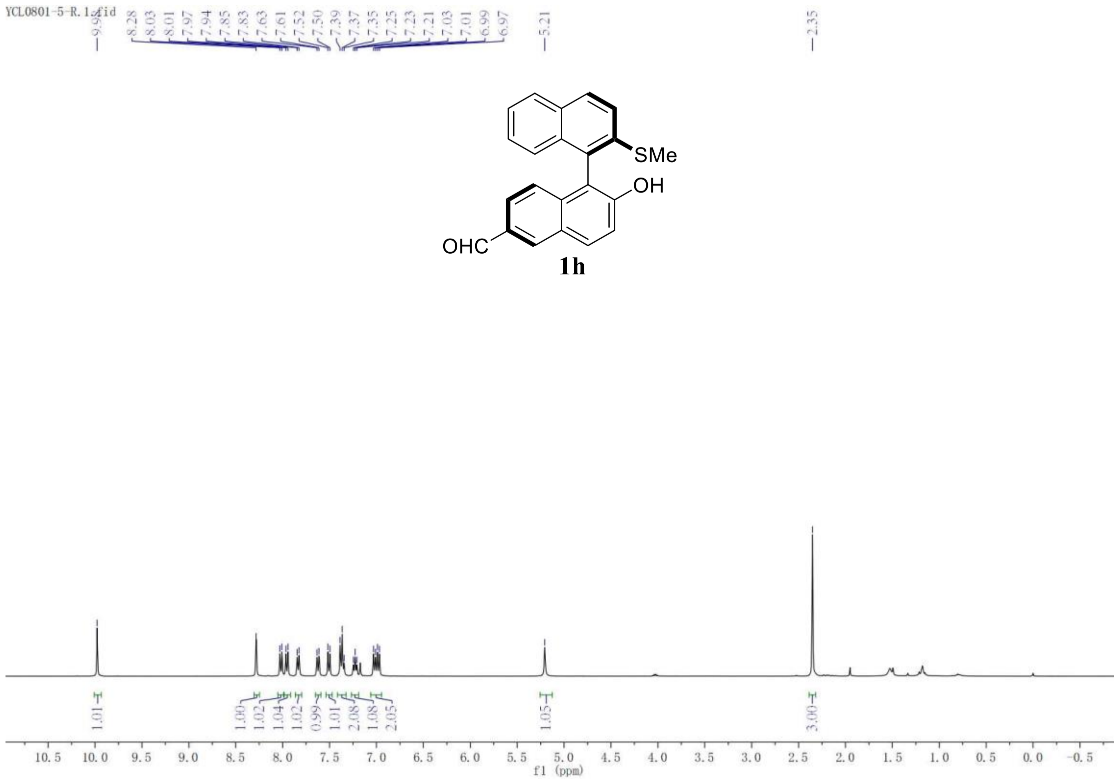
YCL0801-5-R.1.fid
9.98
8.28
8.03
8.01
7.97
7.94
7.85
7.83
7.63
7.61
7.52
7.50
7.39
7.37
7.35
7.25
7.23
7.21
7.03
7.01
6.99
6.97
5.21
2.35
SMe
OH
OHC
1h
1.01
1.00
1.02
1.04
1.02
0.99
1.01
2.08
1.08
2.05
1.05
3.00
f1 (ppm)

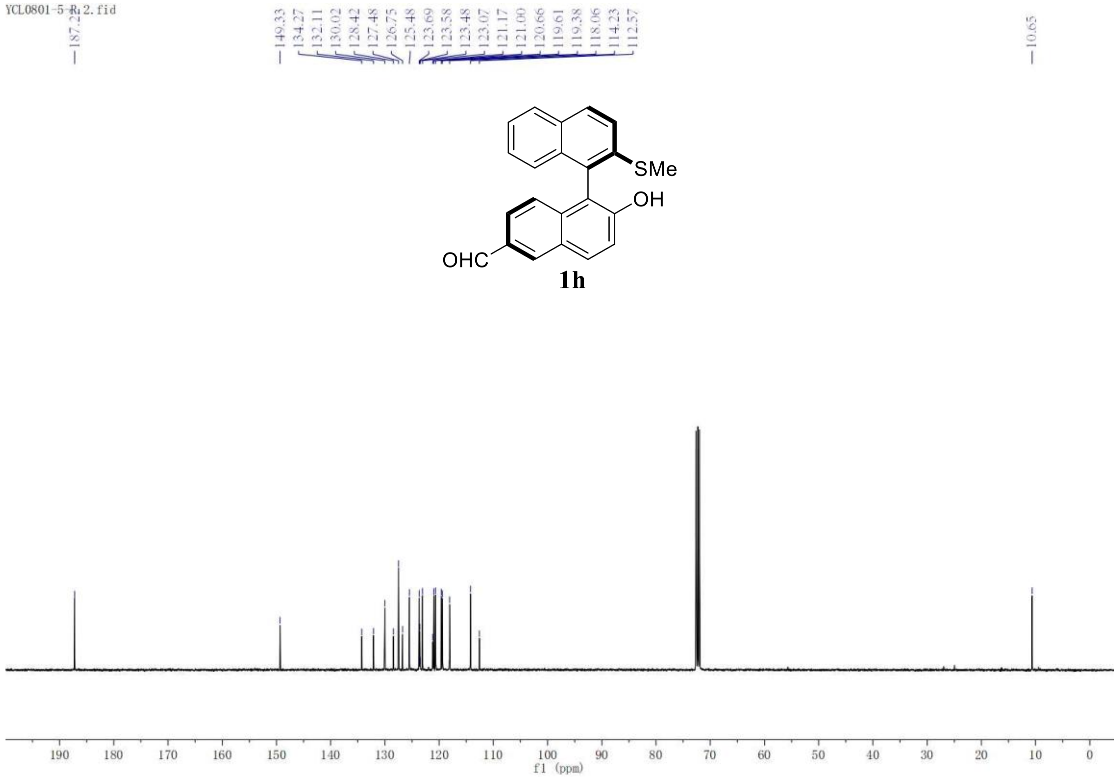
YCL0801-5-R.2.fid
187.2
149.33
134.27
132.11
130.02
128.42
127.48
126.75
125.48
123.69
123.58
123.48
123.07
121.17
121.00
120.66
119.61
119.38
118.06
114.23
112.57
10.65
SMe
OH
OHC
1h
f1 (ppm)

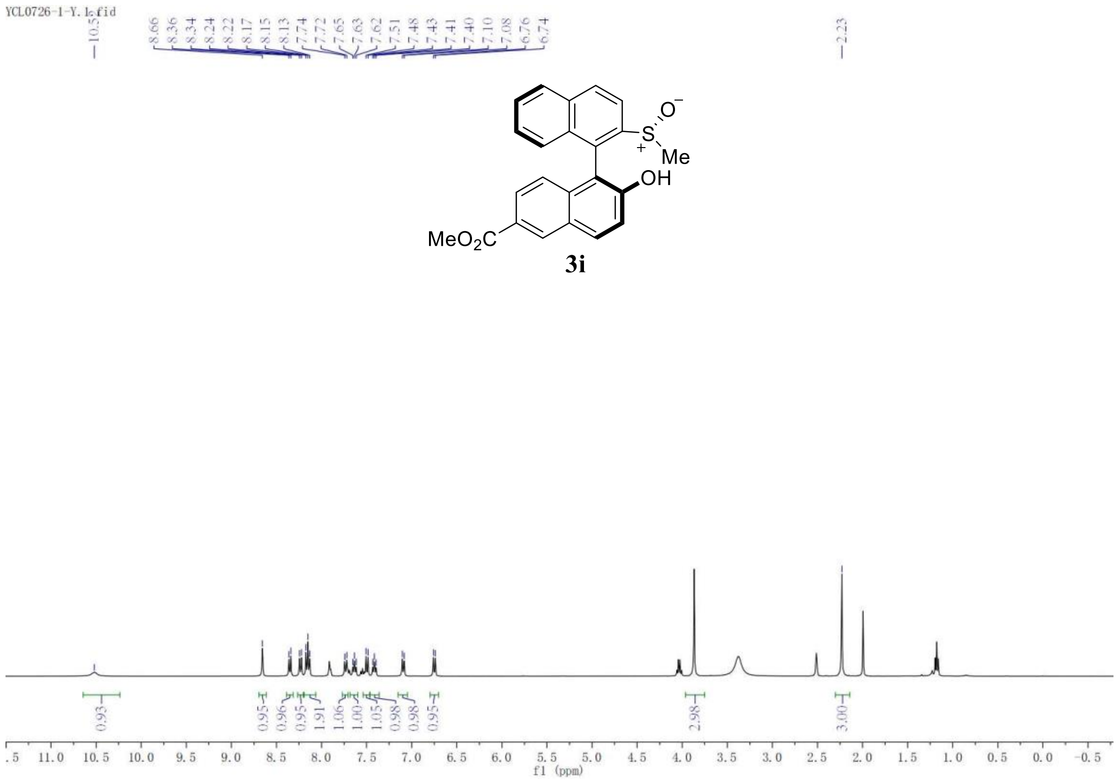
YCL0726-1-Y.1.fid
MeO2C
3i
f1 (ppm)

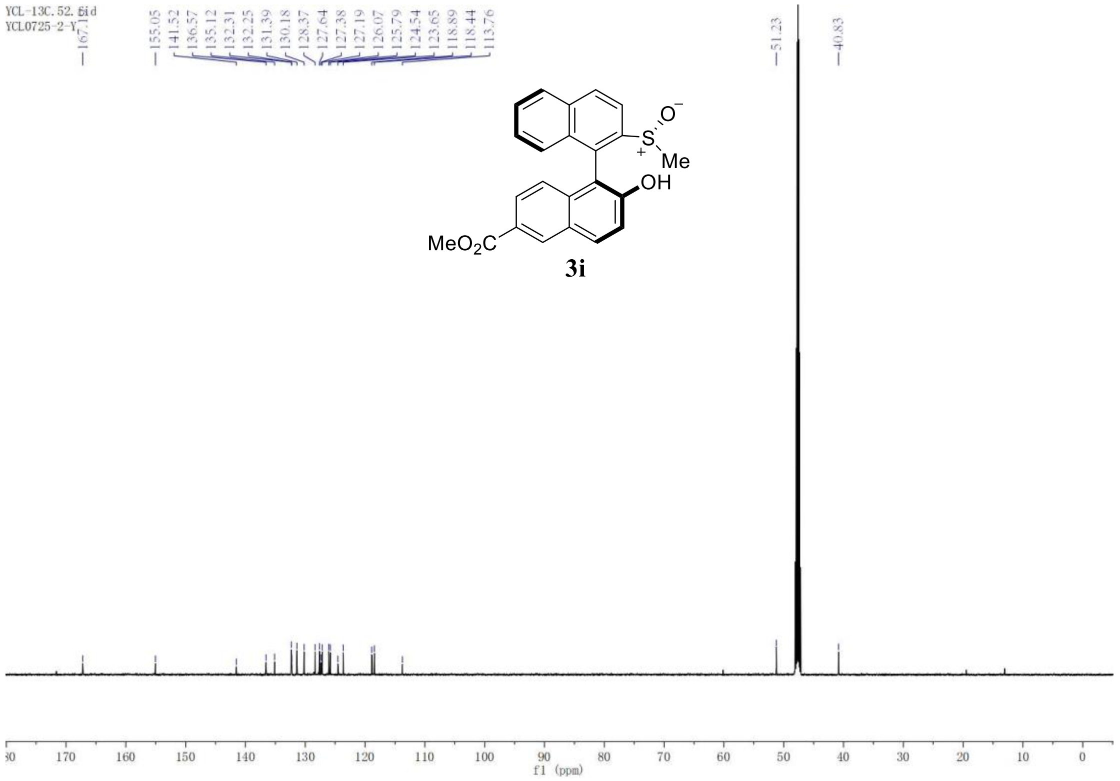
YCL-13C.52.fid
YCL0725-2-Y
MeO2C
3i
f1 (ppm)

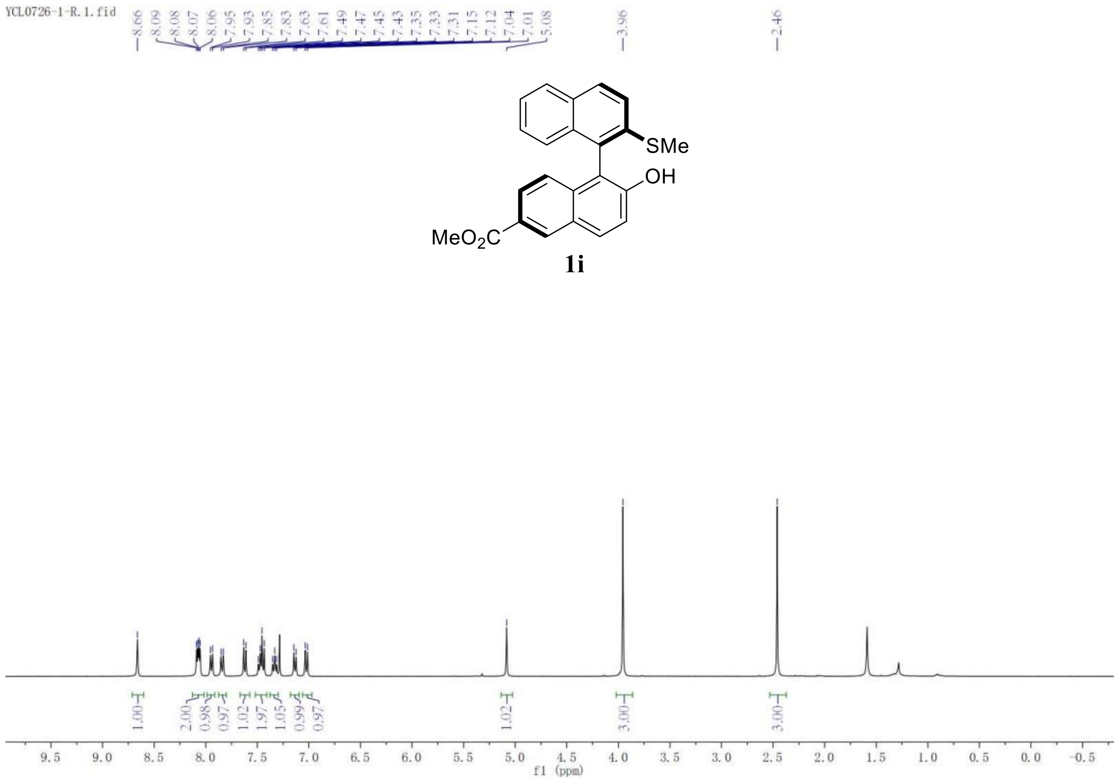
YCL0726-1-R.1.fid
8.66 8.09 8.08 8.07 8.06 7.95 7.93 7.85 7.83 7.63 7.61 7.49 7.47 7.45 7.43 7.35 7.33 7.31 7.15 7.12 7.04 7.01 5.08
3.96
2.46
SMe
OH
MeO2C
1i
1.00 2.00 0.98 0.97 1.02 1.97 1.05 0.99 0.97 1.02 3.00 3.00
f1 (ppm)

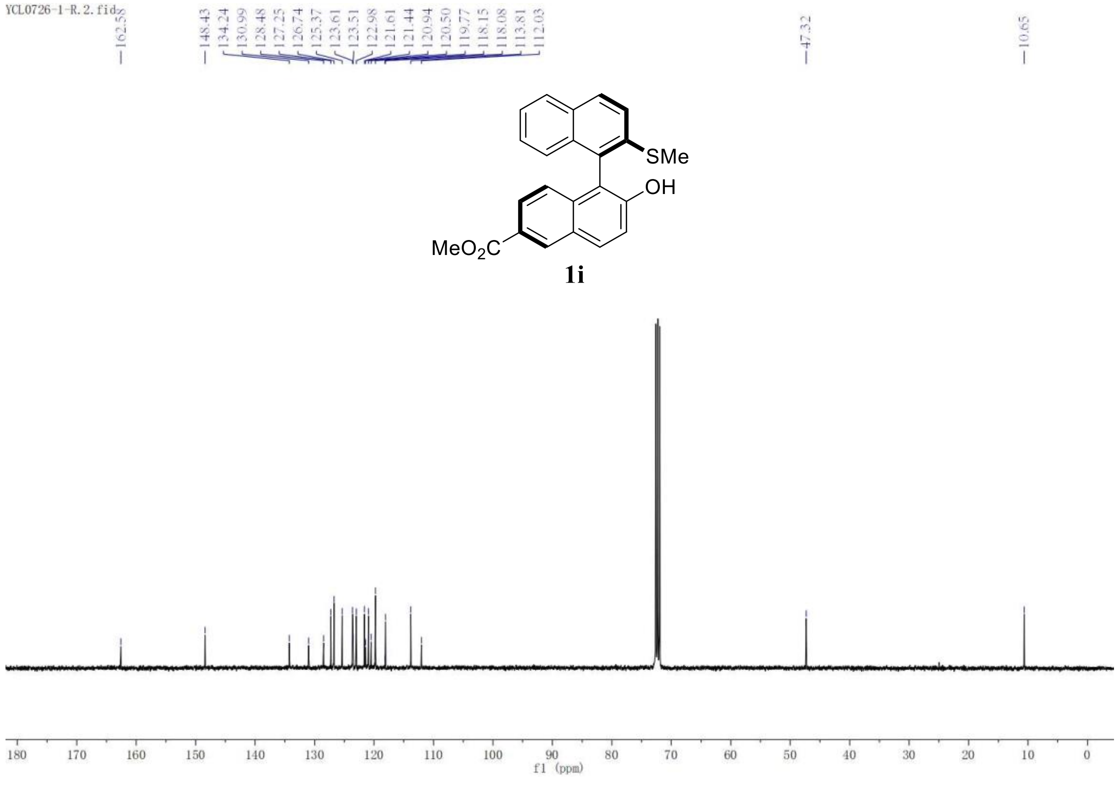
YCL0726-1-R.2.fid
162.58
148.43 134.24 130.99 128.48 127.25 126.74 125.37 123.61 123.51 122.98 121.61 121.44 120.94 120.50 119.77 118.15 118.08 113.81 112.03
47.32
10.65
SMe
OH
MeO2C
1i
f1 (ppm)

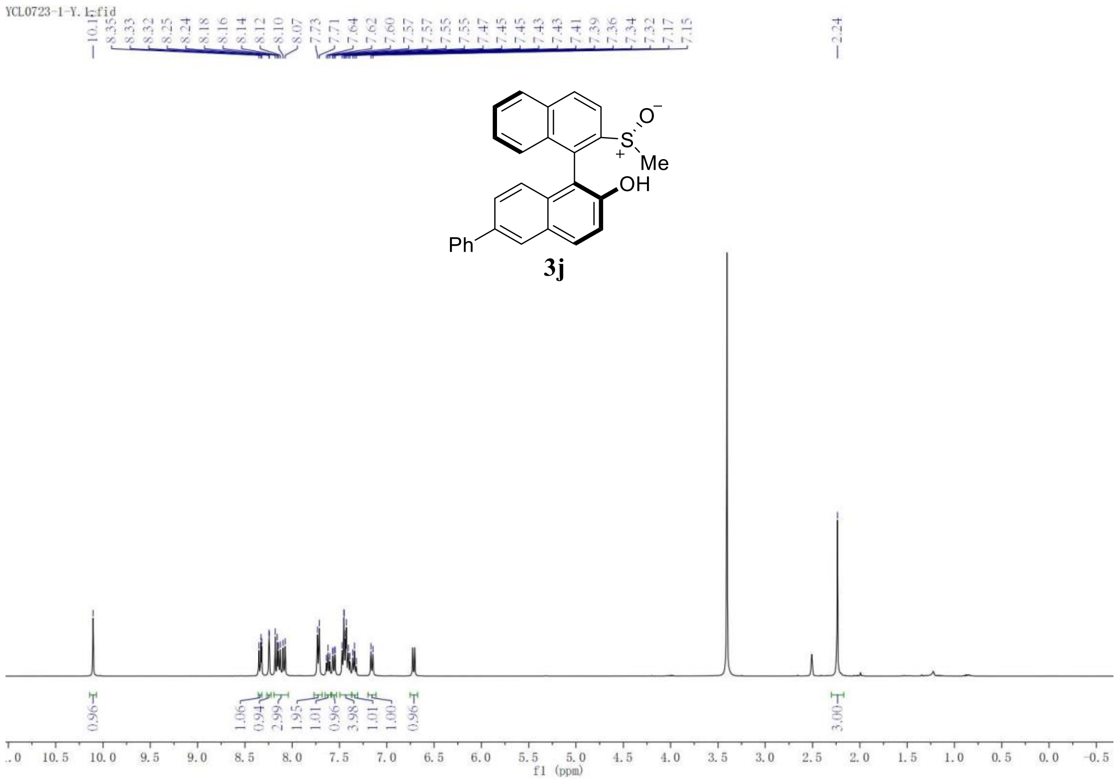
YCL0723-1-Y.1.fid
10.11 8.35 8.33 8.32 8.25 8.24 8.18 8.16 8.14 8.12 8.10 8.07 7.73 7.71 7.64 7.62 7.60 7.57 7.57 7.55 7.55 7.47 7.45 7.45 7.43 7.43 7.41 7.39 7.36 7.34 7.32 7.17 7.15
2.24
O
S
+
Me
OH
Ph
3j
0.96 1.06 0.94 2.99 1.95 1.01 0.96 3.98 1.01 1.00 0.96 3.00
f1 (ppm)

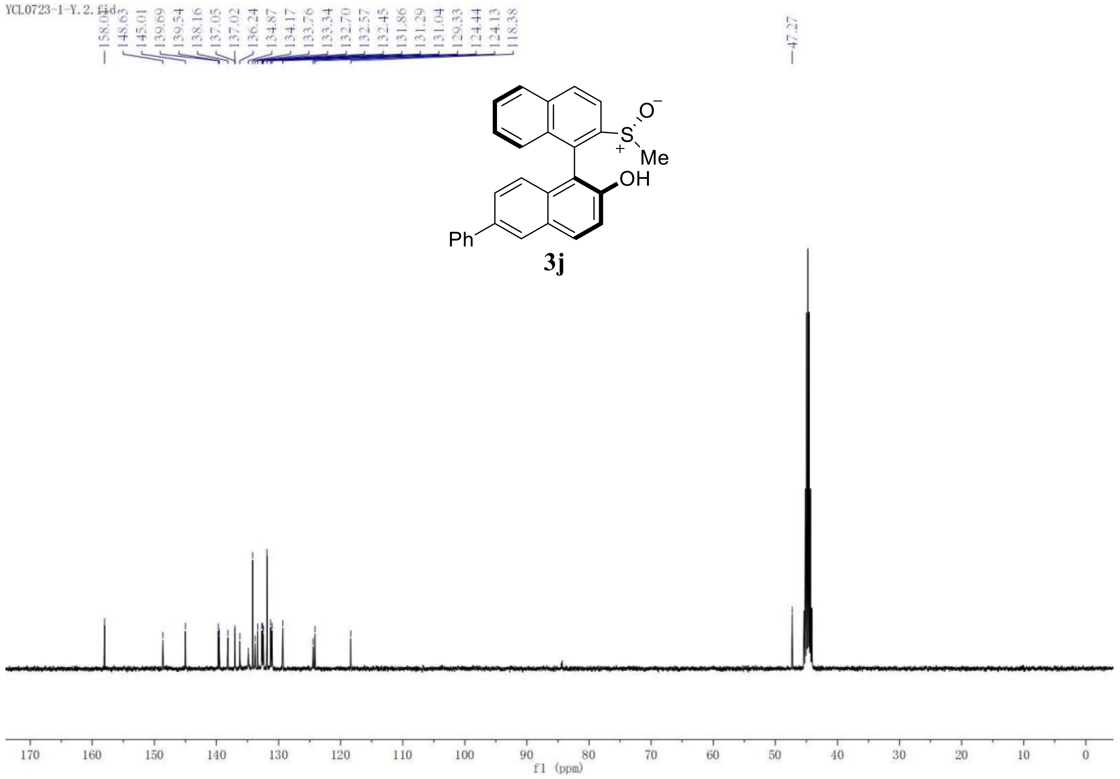
YCL0723-1-Y.2.fid
158.01 148.63 145.01 139.69 139.54 138.16 137.05 137.02 136.24 134.87 134.17 133.76 133.34 132.70 132.57 132.45 131.86 131.29 131.04 129.33 124.44 124.13 118.38
47.27
O
S
+
Me
OH
Ph
3j
f1 (ppm)

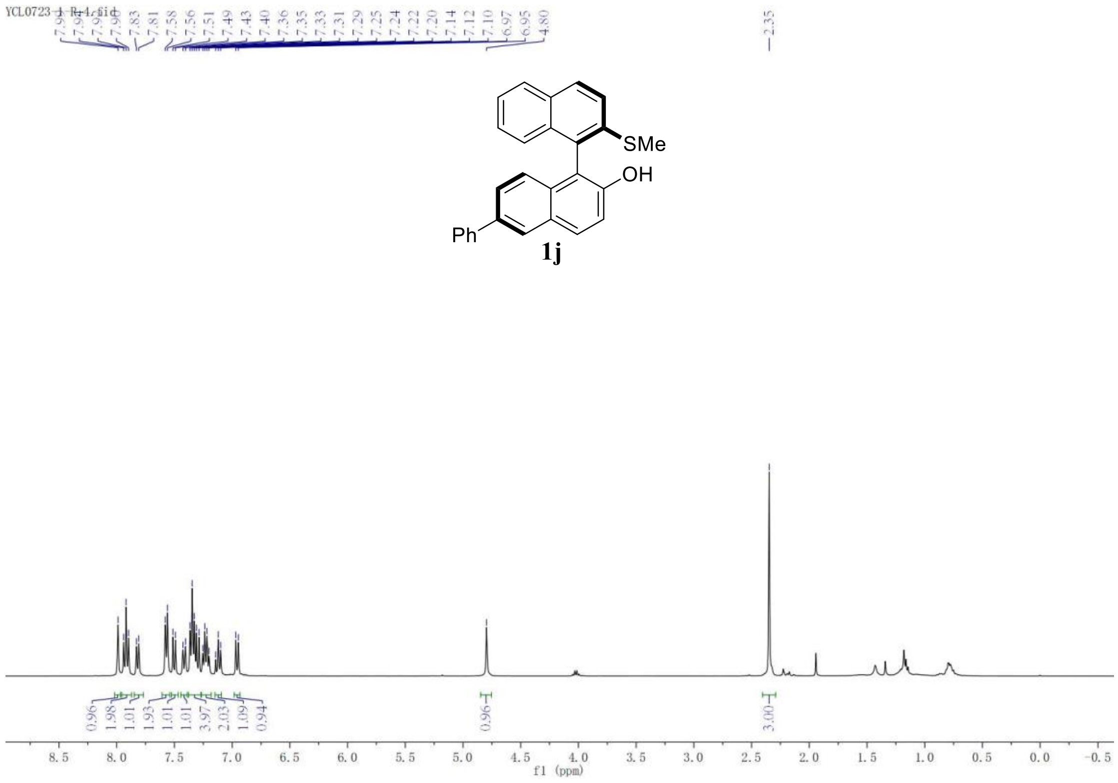
YCL0723-1-R.4.fid
7.99
7.83
7.81
7.58
7.56
7.51
7.49
7.43
7.40
7.36
7.35
7.33
7.31
7.29
7.25
7.24
7.22
7.20
7.14
7.12
7.10
6.97
6.95
4.80
2.35
SMe
OH
Ph
1j
0.96
1.98
1.01
1.93
1.01
1.01
3.97
2.03
1.09
0.94
0.96
3.00
8.5 8.0 7.5 7.0 6.5 6.0 5.5 5.0 4.5 4.0 3.5 3.0 2.5 2.0 1.5 1.0 0.5 0.0 -0.5
f1 (ppm)

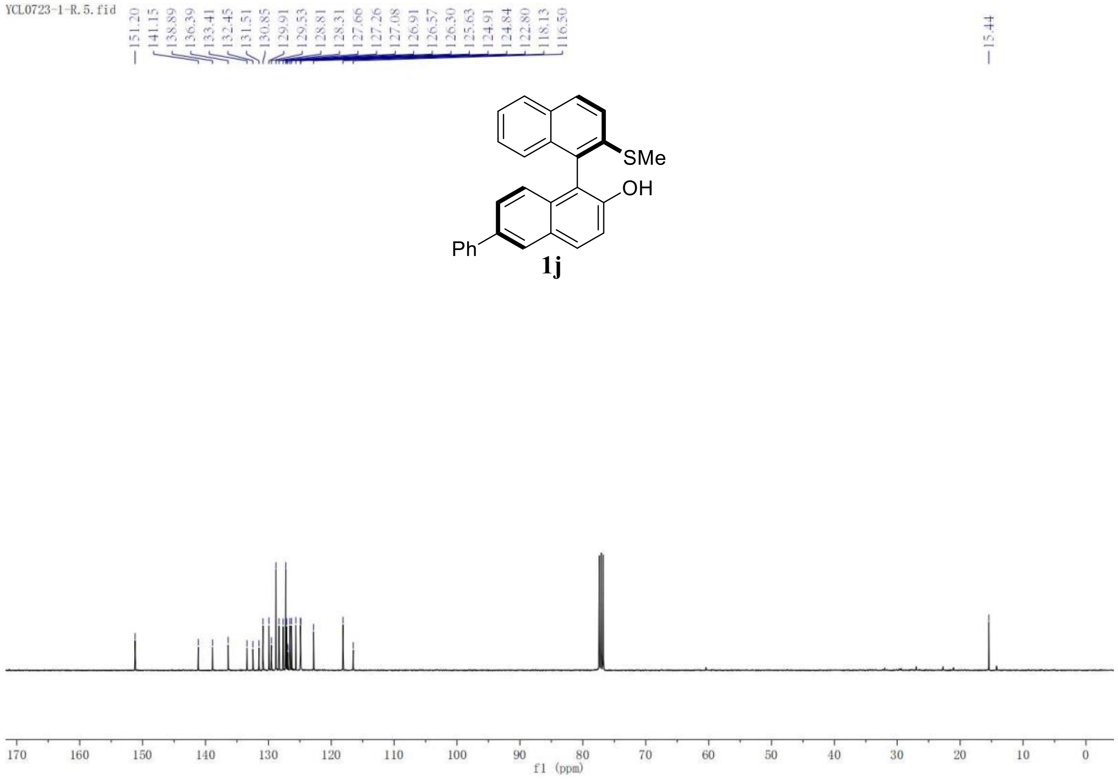
YCL0723-1-R.5.fid
151.20
141.15
138.89
136.39
133.41
132.45
131.51
130.85
129.91
129.53
128.81
128.31
127.66
127.26
127.08
126.91
126.57
126.30
125.63
124.91
124.84
122.80
118.13
116.50
15.44
SMe
OH
Ph
1j
170 160 150 140 130 120 110 100 90 80 70 60 50 40 30 20 10 0
f1 (ppm)

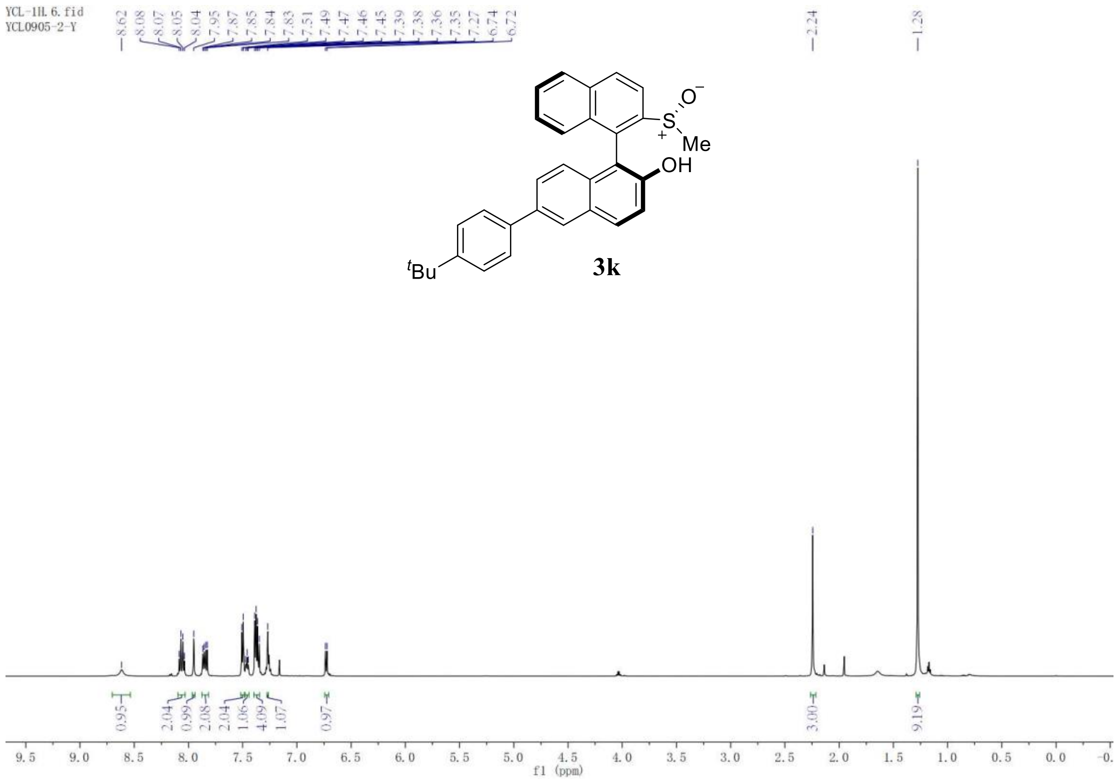
YCL-1H.6.fid
YCL0905-2-Y
8.62
8.08
8.07
8.05
8.04
7.95
7.87
7.85
7.84
7.83
7.51
7.49
7.47
7.46
7.45
7.39
7.38
7.36
7.35
7.27
6.74
6.72
2.24
1.28
OH
Me
3k
0.95
2.04
0.99
2.08
2.04
1.06
4.09
1.07
0.97
3.00
9.19
f1 (ppm)

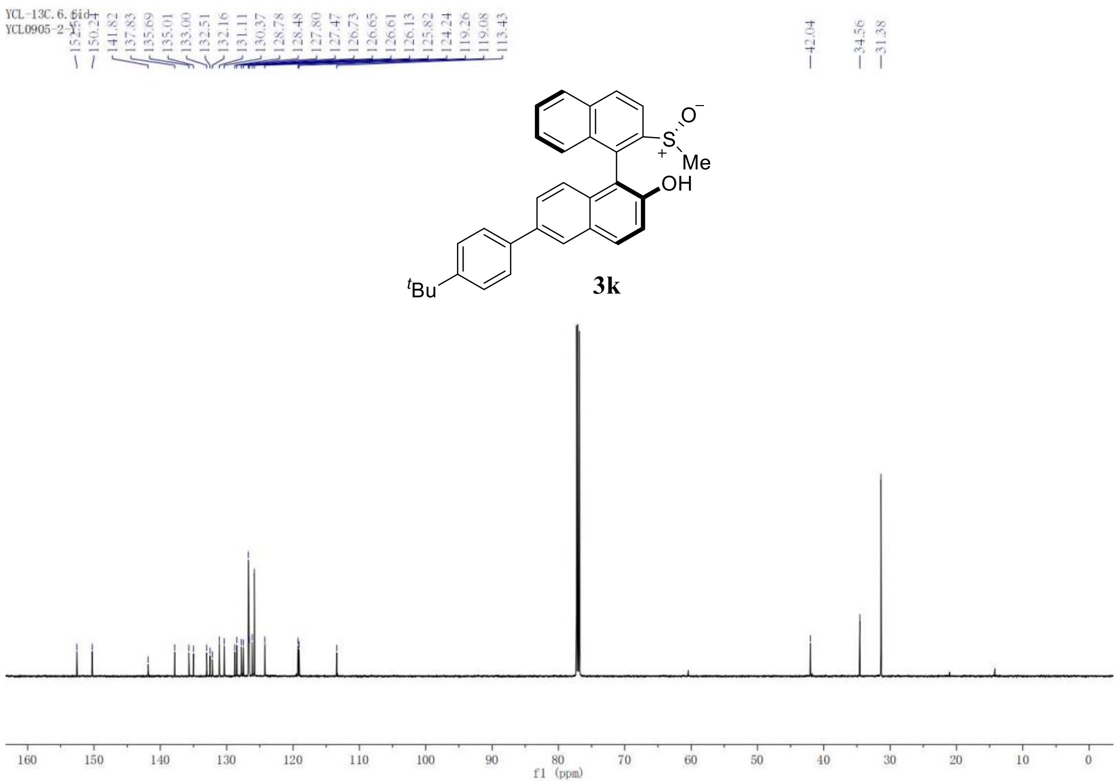
YCL-13C.6.fid
YCL0905-2-Y
150.24
141.82
137.83
135.69
135.01
133.00
132.51
132.16
131.11
130.37
128.78
128.48
127.80
127.47
126.73
126.65
126.61
126.13
125.82
124.24
119.26
119.08
113.43
42.04
34.56
31.38
OH
Me
3k
f1 (ppm)

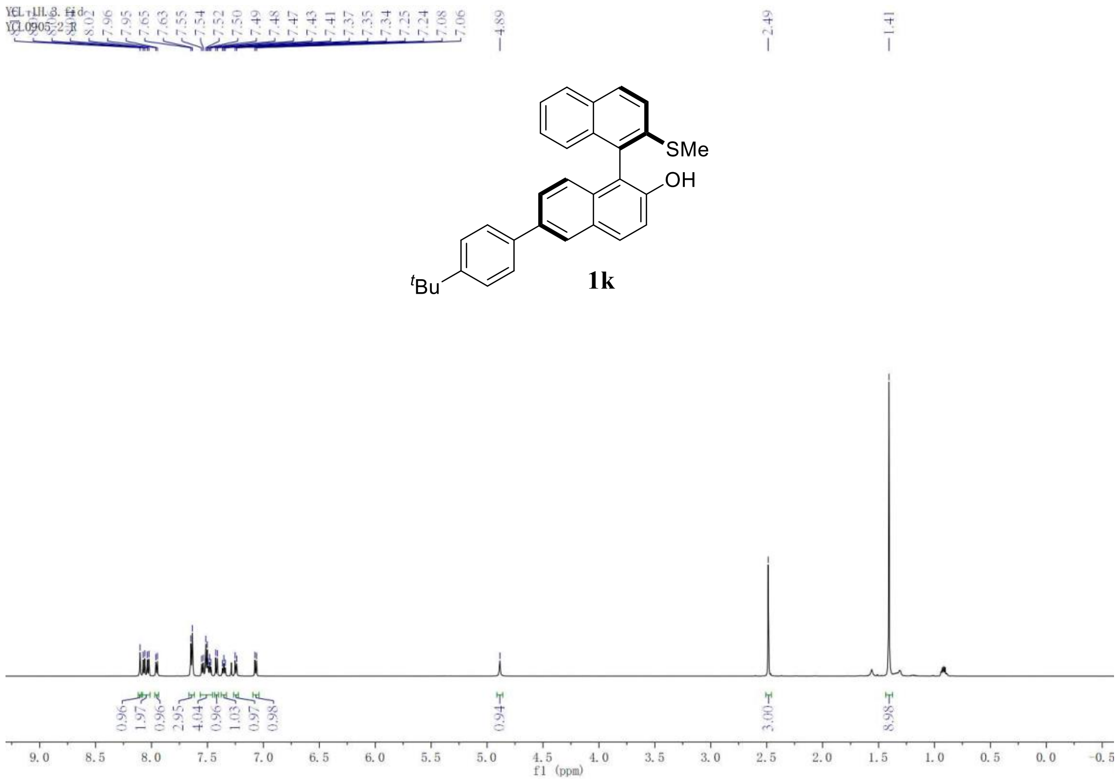
YCL-1H.8.fid
YCL0905-2-R
8.02 7.96 7.95 7.65 7.63 7.55 7.54 7.52 7.50 7.49 7.48 7.47 7.43 7.41 7.37 7.35 7.34 7.25 7.24 7.08 7.06
4.89
2.49
1.41
SMe
OH
tBu
1k
0.96 1.97 0.96 2.95 4.04 0.96 1.03 0.97 0.98
0.94
3.00
8.98
9.0 8.5 8.0 7.5 7.0 6.5 6.0 5.5 5.0 4.5 4.0 3.5 3.0 2.5 2.0 1.5 1.0 0.5 0.0 -0.5
f1 (ppm)

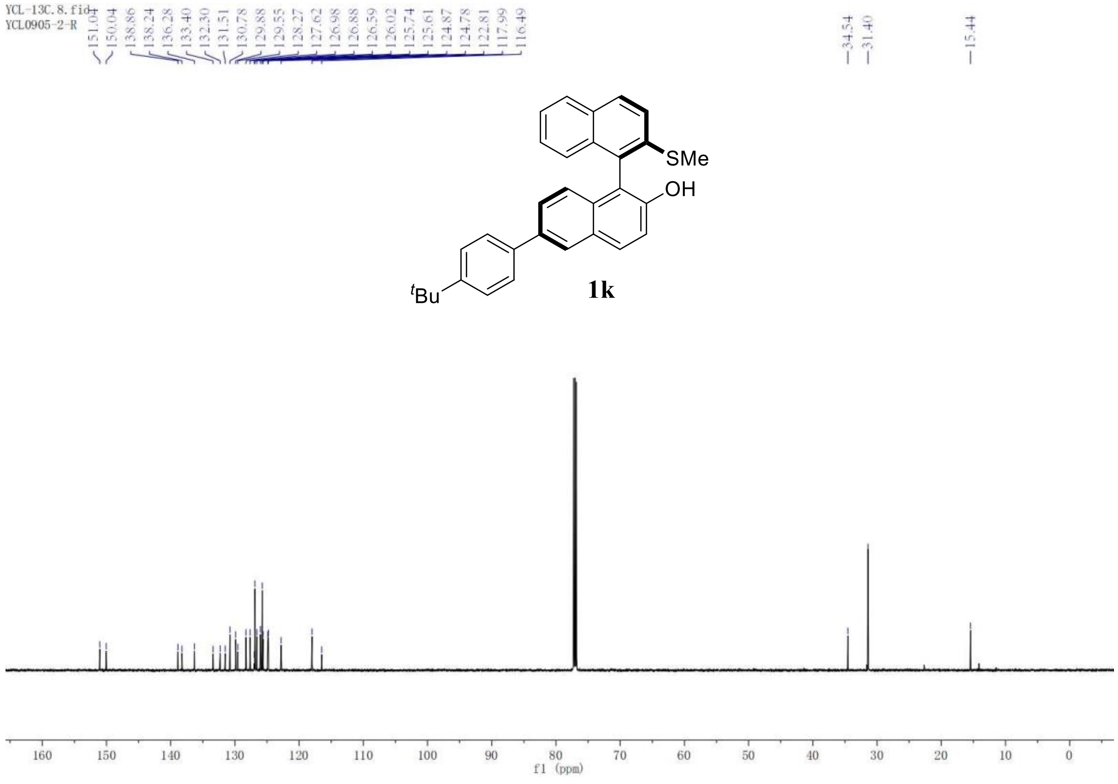
YCL-13C.8.fid
YCL0905-2-R
151.04 150.04 138.86 138.24 136.28 133.40 132.30 131.51 130.78 129.88 129.55 128.27 127.62 126.98 126.88 126.59 126.02 125.74 125.61 124.87 124.78 122.81 117.99 116.49
34.54
31.40
15.44
SMe
OH
tBu
1k
160 150 140 130 120 110 100 90 80 70 60 50 40 30 20 10 0
f1 (ppm)

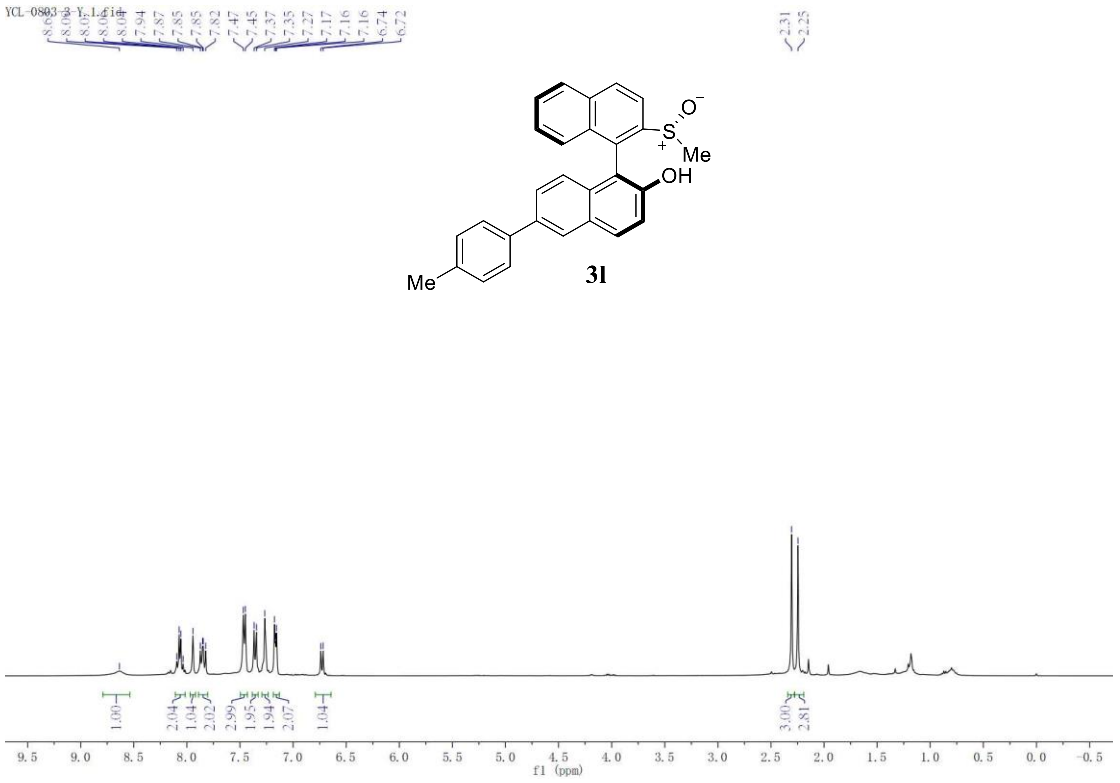
YCL-0803-3-Y.1.fid
8.04
7.94
7.87
7.85
7.85
7.82
7.47
7.45
7.37
7.35
7.27
7.17
7.16
7.16
6.74
6.72
2.31
2.25
O
S
+
Me
OH
Me
3l
1.00
2.04
1.04
2.02
2.99
1.95
1.94
2.07
1.04
3.00
2.81
f1 (ppm)

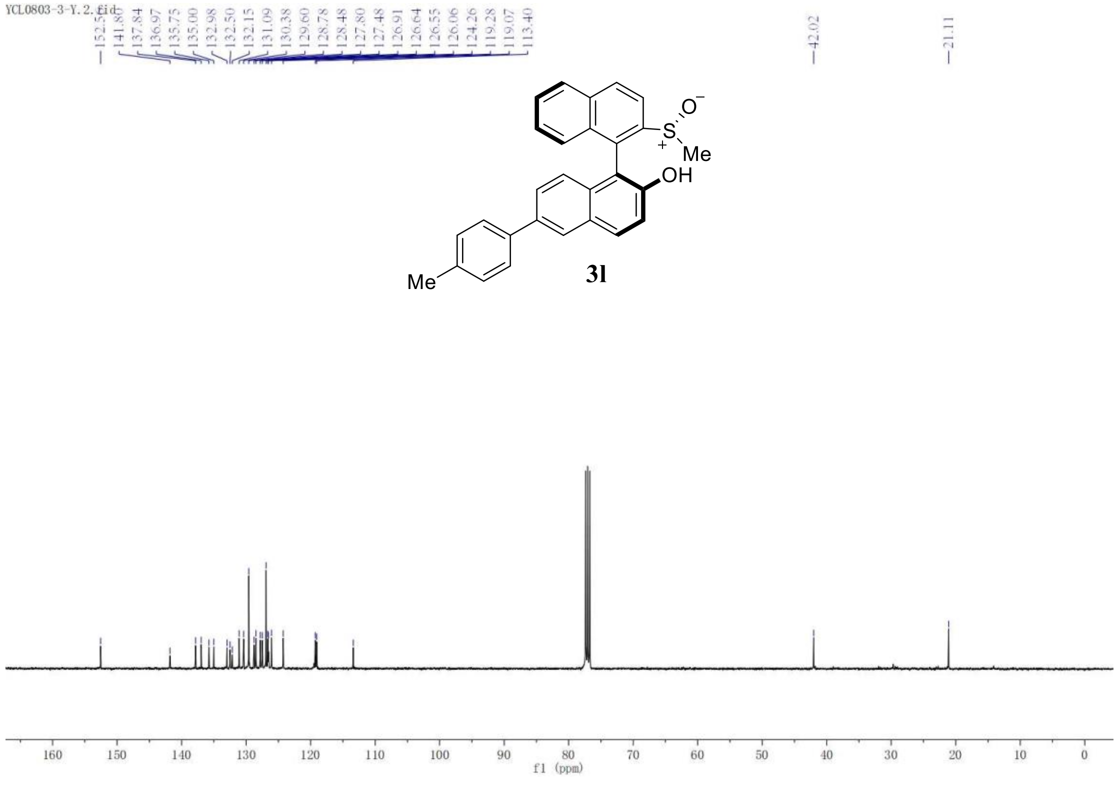
YCL0803-3-Y.2.fid
141.80
137.84
136.97
135.75
135.00
132.98
132.50
132.15
131.09
130.38
129.60
128.78
128.48
127.80
127.48
126.91
126.64
126.55
126.06
124.26
119.28
119.07
113.40
42.02
21.11
O
S
+
Me
OH
Me
3l
f1 (ppm)

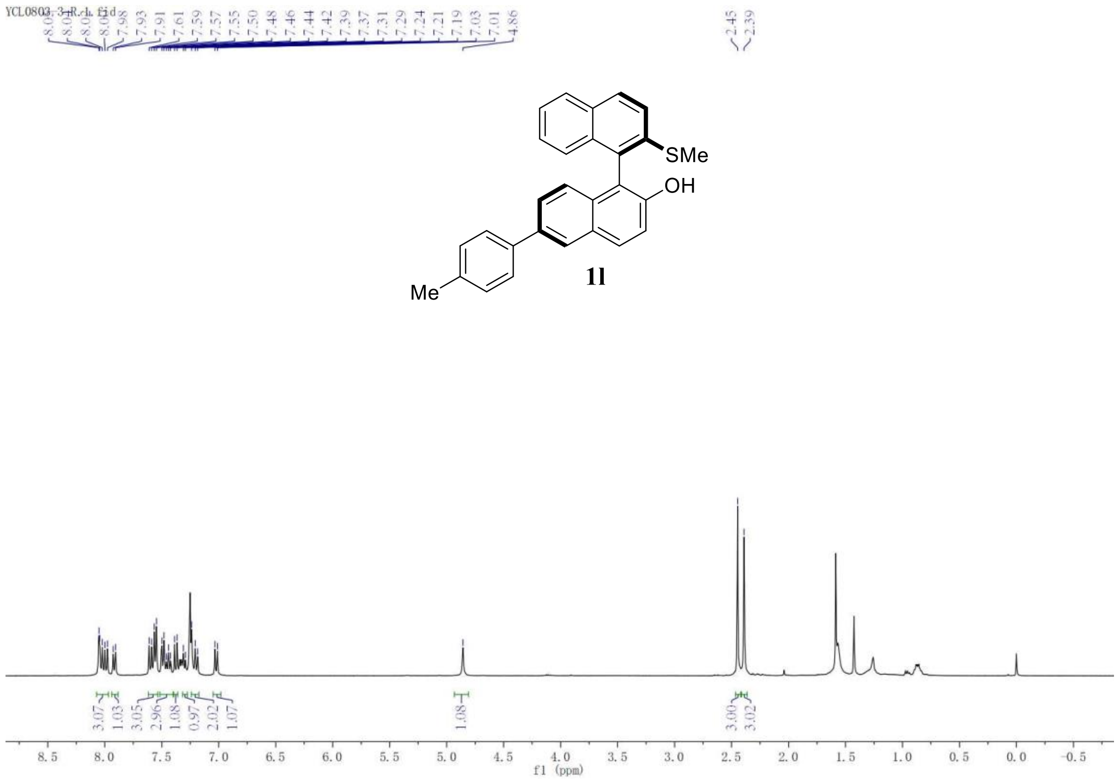
YCL0803-3-R.1.fid
8.0[illegible] 8.0[illegible] 8.0[illegible] 8.01 7.98 7.93 7.91 7.61 7.59 7.57 7.55 7.50 7.48 7.46 7.44 7.42 7.39 7.37 7.31 7.29 7.24 7.21 7.19 7.03 7.01 4.86
2.45 2.39
SMe
OH
Me
3.07 1.03 3.05 2.96 1.08 0.97 2.02 1.07 1.08 3.00 3.02
8.5 8.0 7.5 7.0 6.5 6.0 5.5 5.0 4.5 4.0 3.5 3.0 2.5 2.0 1.5 1.0 0.5 0.0 -0.5
f1 (ppm)

1l

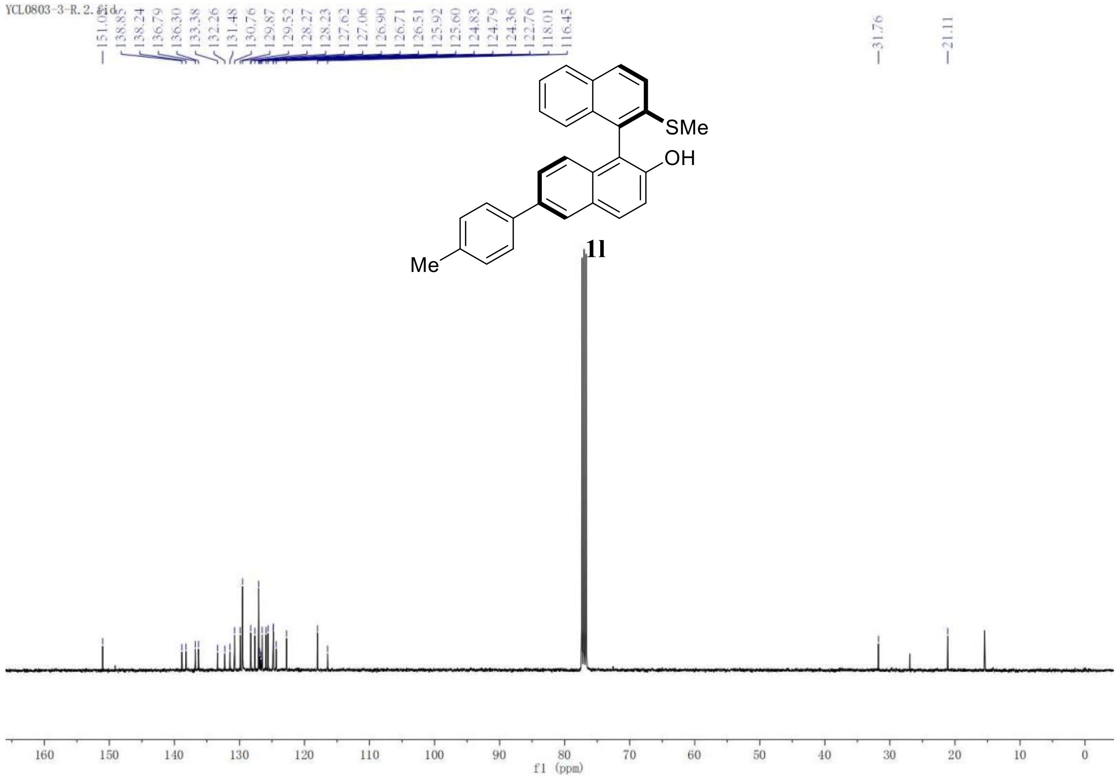
YCL0803-3-R.2.fid
151.05 138.85 138.24 136.79 136.30 133.38 132.26 131.48 130.76 129.87 129.52 128.27 128.23 127.62 127.06 126.90 126.71 126.51 125.92 125.60 124.83 124.79 124.36 122.76 118.01 116.45
31.76
21.11
SMe
OH
Me
160 150 140 130 120 110 100 90 80 70 60 50 40 30 20 10 0
f1 (ppm)

1l

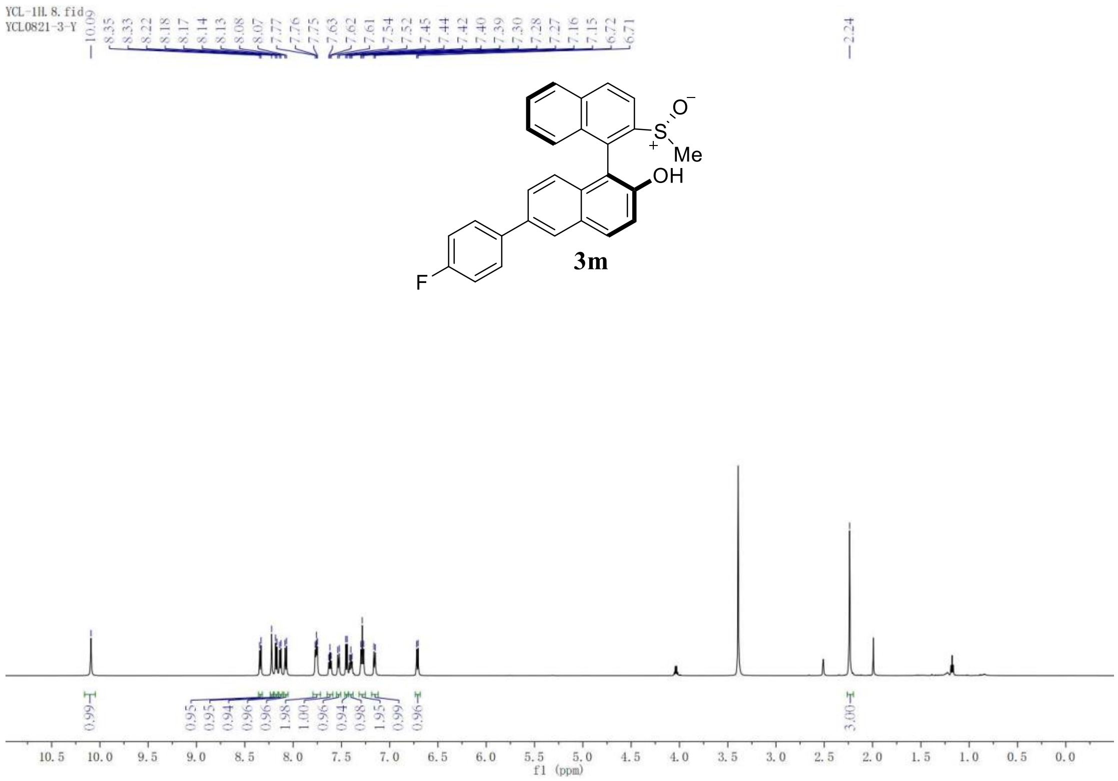
YCL-1H.8.fid
YCL0821-3-Y
10.09 8.35 8.33 8.22 8.18 8.17 8.14 8.13 8.08 8.07 7.77 7.76 7.75 7.63 7.62 7.61 7.54 7.52 7.45 7.44 7.42 7.40 7.39 7.30 7.28 7.27 7.16 7.15 6.72 6.71
2.24
O
S
Me
OH
F
3m
0.99 0.95 0.95 0.94 0.96 0.96 1.98 1.00 0.96 0.94 0.98 1.95 0.99 0.96 3.00
f1 (ppm)

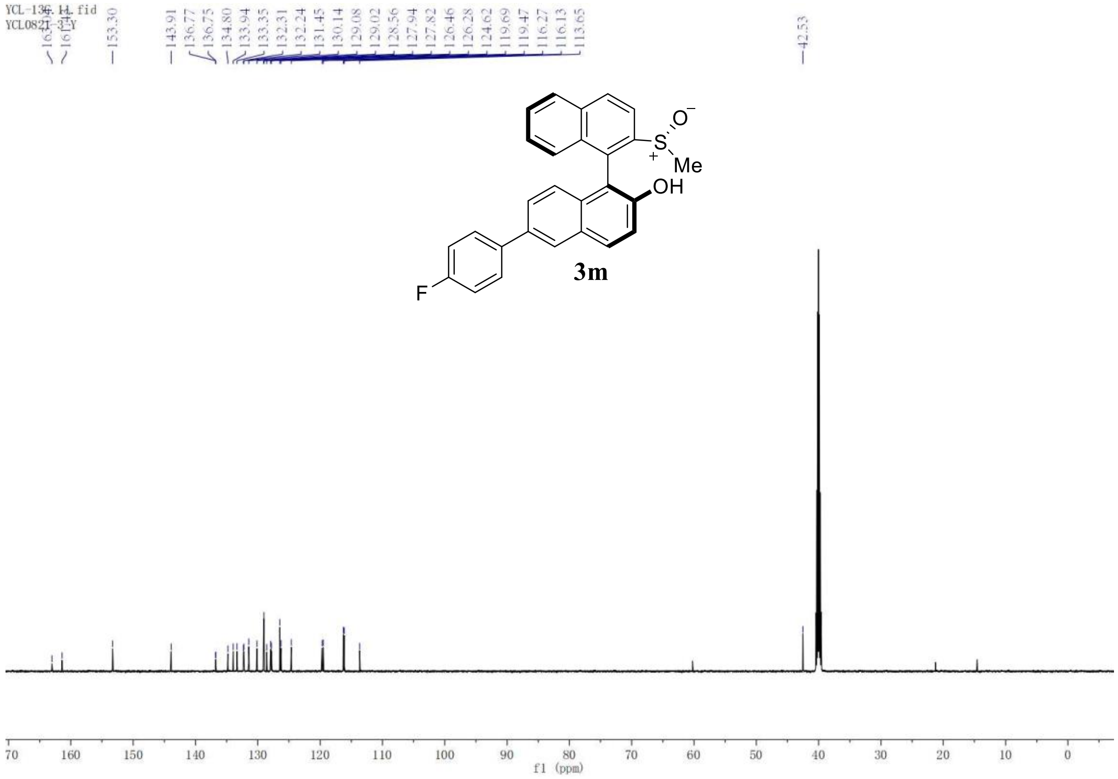
YCL-13C.14.fid
YCL0821-3-Y
[illegible] [illegible] 153.30 143.91 136.77 136.75 134.80 133.94 133.35 132.31 132.24 131.45 130.14 129.08 129.02 128.56 127.94 127.82 126.46 126.28 124.62 119.69 119.47 116.27 116.13 113.65
42.53
O
S
Me
OH
F
3m
f1 (ppm)

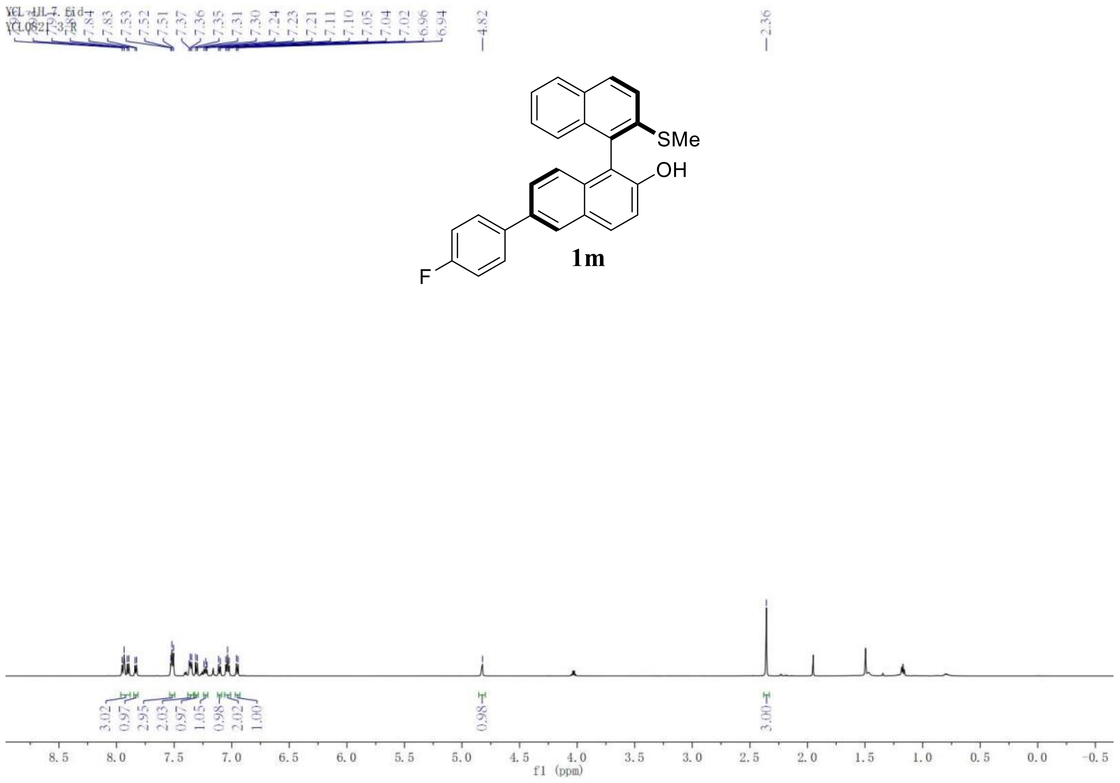
YCL-1H-7.fid
YCL0821-3-R
7.83 7.53 7.52 7.51 7.37 7.36 7.35 7.31 7.30 7.24 7.23 7.21 7.11 7.10 7.05 7.04 7.02 6.96 6.94
4.82
2.36
SMe
OH
F
1m
3.02 0.97 2.95 2.03 0.97 1.05 0.98 2.02 1.00
0.98
3.00
8.5 8.0 7.5 7.0 6.5 6.0 5.5 5.0 4.5 4.0 3.5 3.0 2.5 2.0 1.5 1.0 0.5 0.0 -0.5
f1 (ppm)

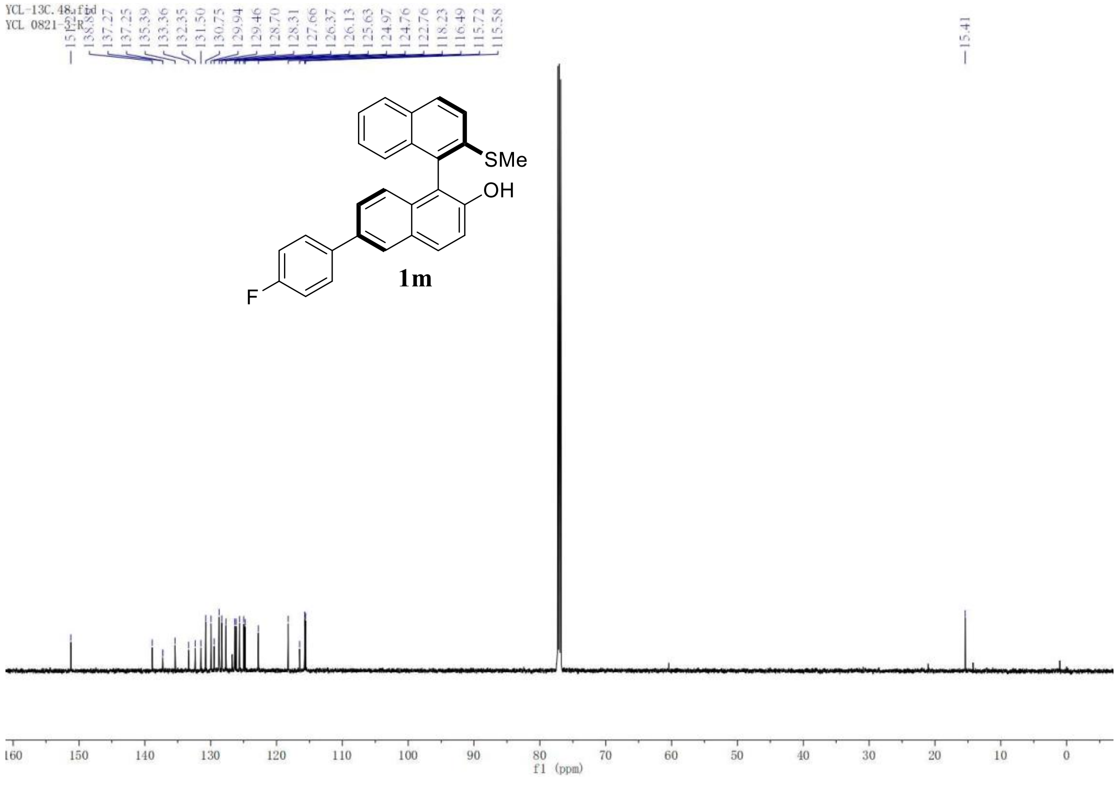
YCL-13C.48.fid
YCL 0821-3-R
137.27 137.25 135.39 133.36 132.35 131.50 130.75 129.94 129.46 128.70 128.31 127.66 126.37 126.13 125.63 124.97 124.76 122.76 118.23 116.49 115.72 115.58
15.41
SMe
OH
F
1m
160 150 140 130 120 110 100 90 80 70 60 50 40 30 20 10 0
f1 (ppm)

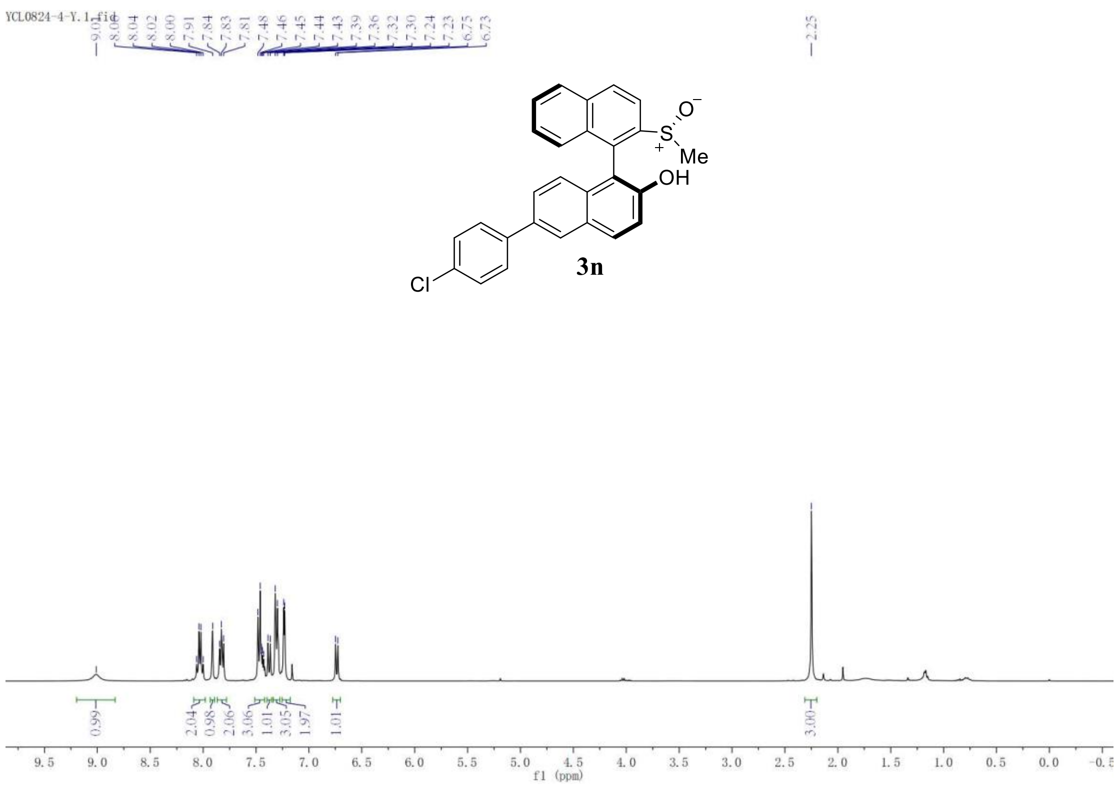
YCL0824-4-Y.1.fid
9.01 8.04 8.02 8.00 7.91 7.84 7.83 7.81 7.48 7.46 7.45 7.44 7.43 7.39 7.36 7.32 7.30 7.24 7.23 6.75 6.73
2.25
O
S
Me
OH
Cl
3n
0.99 2.04 0.98 2.06 3.06 1.01 3.05 1.97 1.01 3.00
9.5 9.0 8.5 8.0 7.5 7.0 6.5 6.0 5.5 5.0 4.5 4.0 3.5 3.0 2.5 2.0 1.5 1.0 0.5 0.0 -0.5
f1 (ppm)

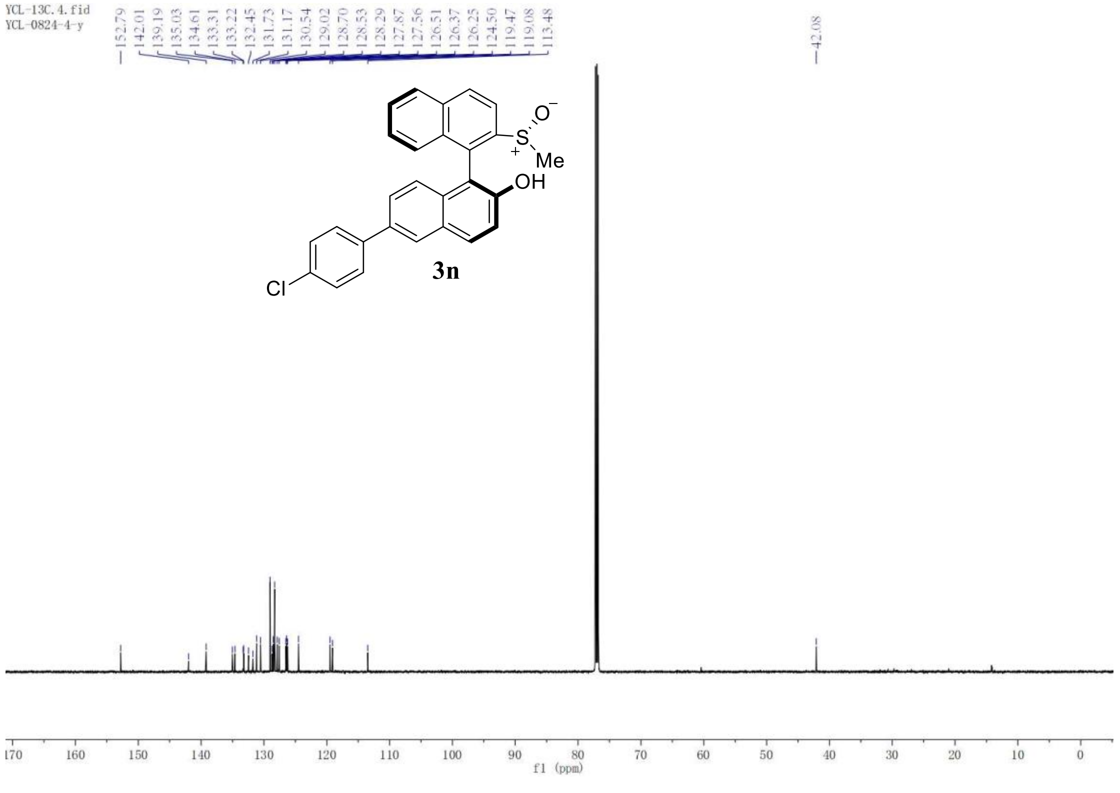
YCL-13C.4.fid
YCL-0824-4-y
152.79 142.01 139.19 135.03 134.61 133.31 133.22 132.45 131.73 131.17 130.54 129.02 128.70 128.53 128.29 127.87 127.56 126.51 126.37 126.25 124.50 119.47 119.08 113.48
42.08
O
S
Me
OH
Cl
3n
170 160 150 140 130 120 110 100 90 80 70 60 50 40 30 20 10 0
f1 (ppm)

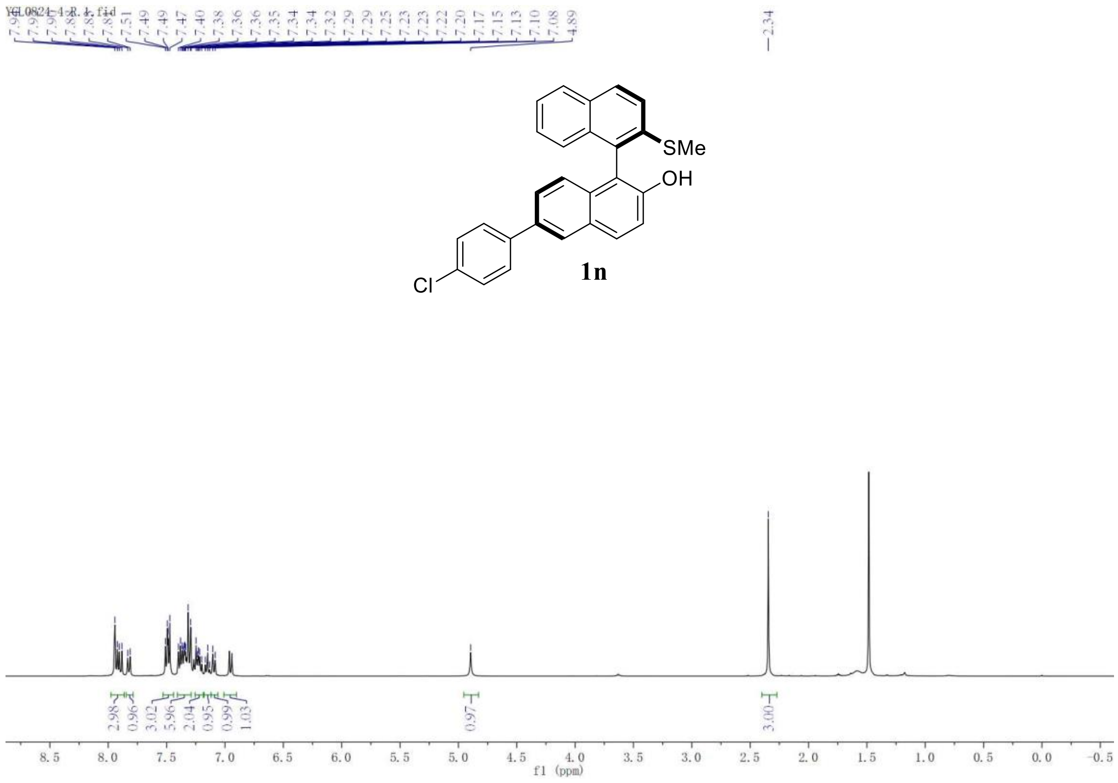

YCL0824-4-R.1.fid
2.34
SMe
OH
Cl
1n
2.98
0.96
3.02
5.96
2.04
0.95
0.99
1.03
0.97
3.00
f1 (ppm)

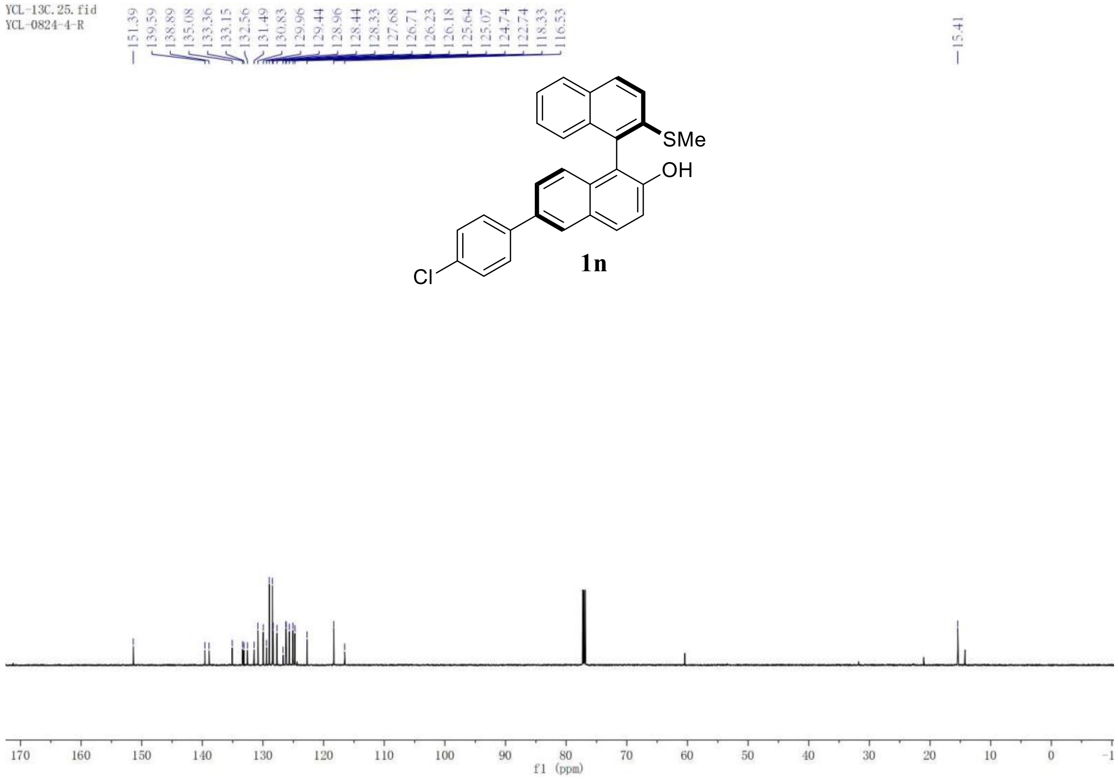

YCL-13C.25.fid
YCL-0824-4-R
151.39
139.59
138.89
135.08
133.36
133.15
132.56
131.49
130.83
129.96
129.44
128.96
128.44
128.33
127.68
126.71
126.23
126.18
125.64
125.07
124.74
122.74
118.33
116.53
15.41
SMe
OH
Cl
1n
f1 (ppm)

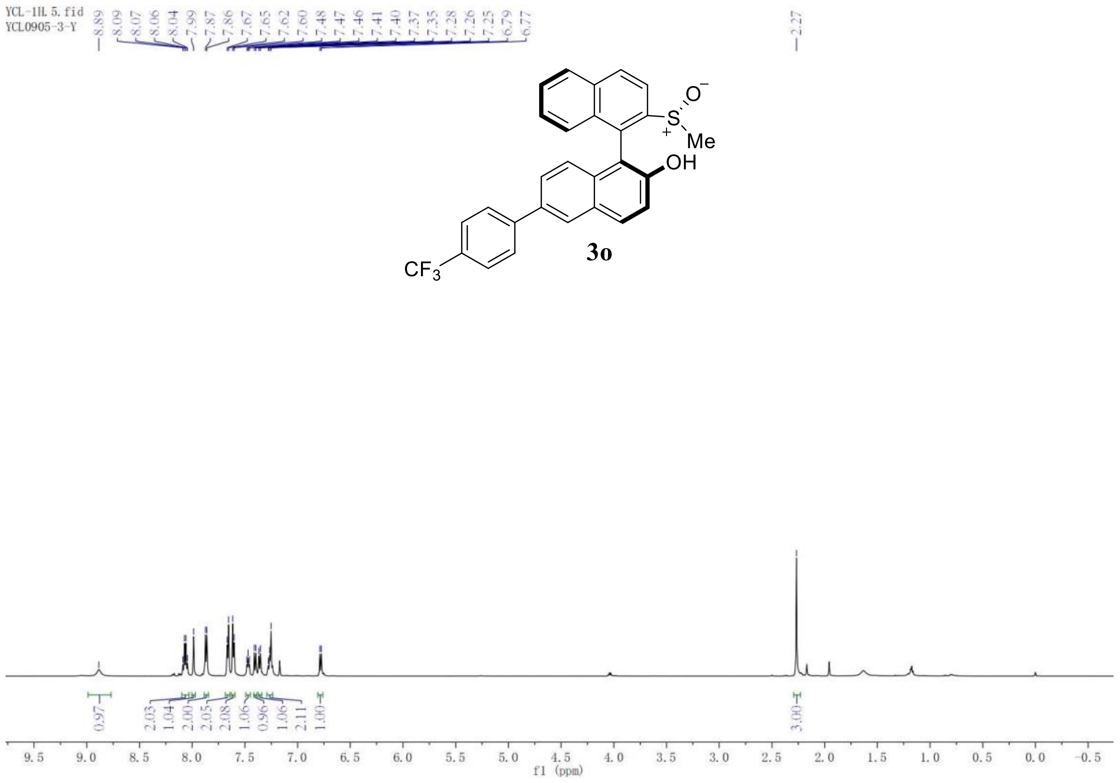

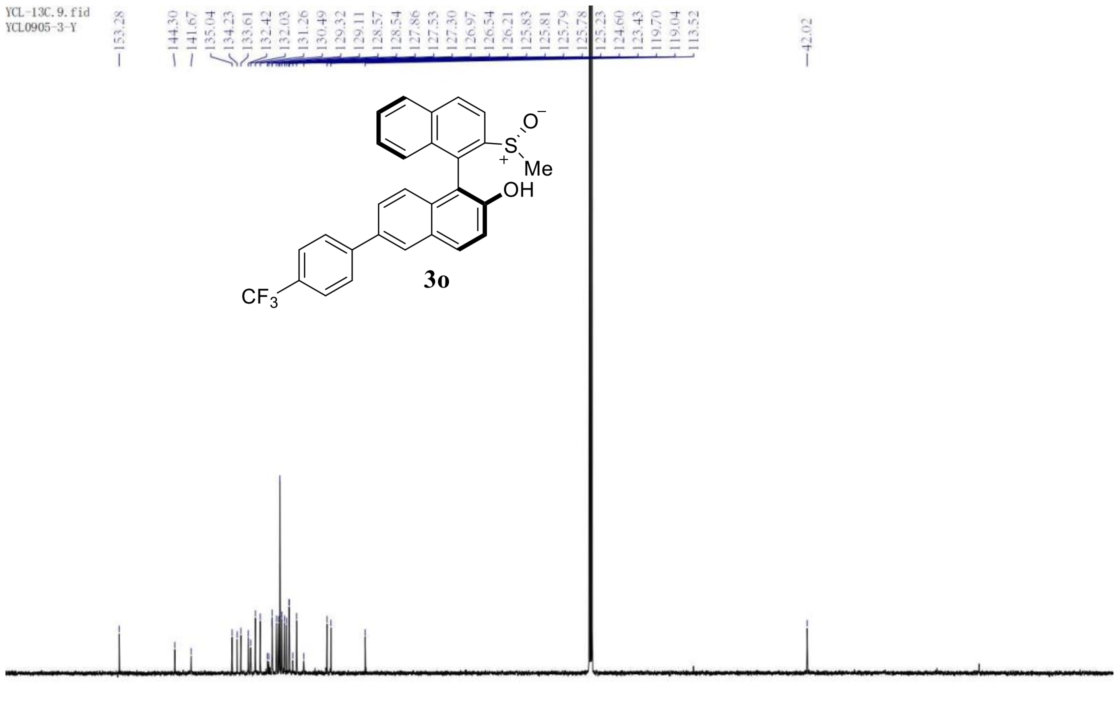

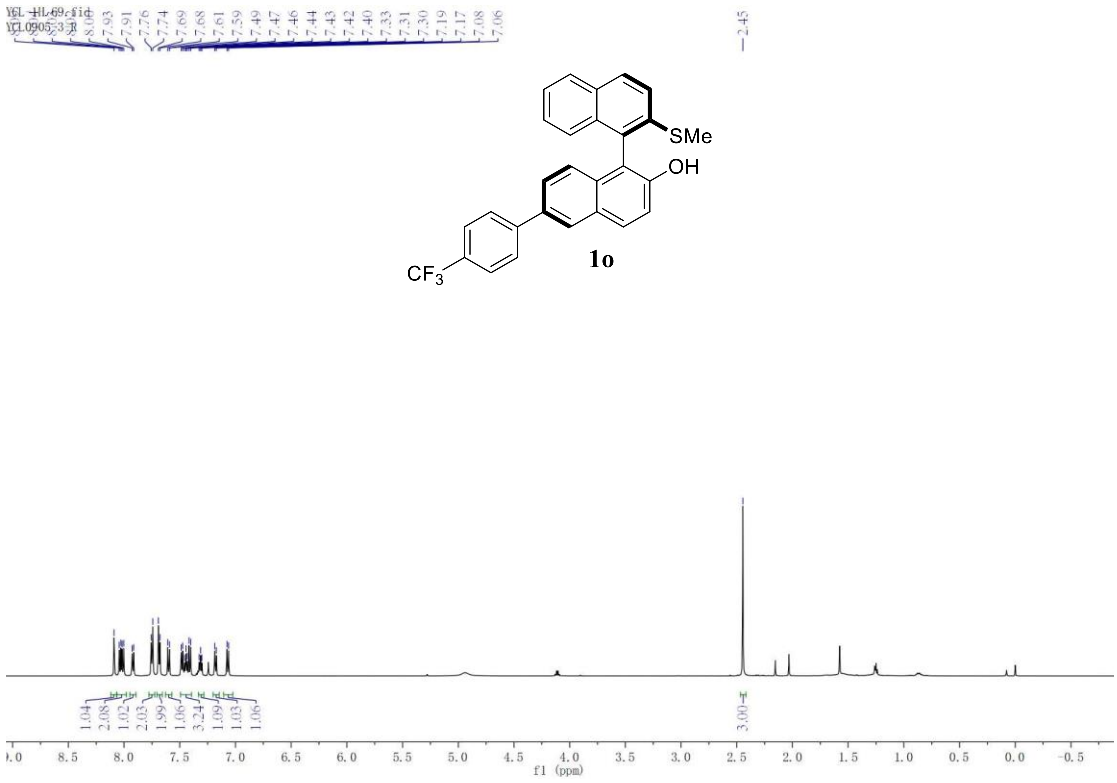
YCL-1H-69.fid
YCL0905-3-R
7.93 7.91 7.76 7.74 7.69 7.68 7.61 7.59 7.49 7.47 7.46 7.44 7.43 7.42 7.40 7.33 7.31 7.30 7.19 7.17 7.08 7.06
2.45
SMe
OH
CF3
1o
1.04 2.08 1.02 2.03 1.99 1.06 3.24 1.09 1.03 1.06
3.00
f1 (ppm)

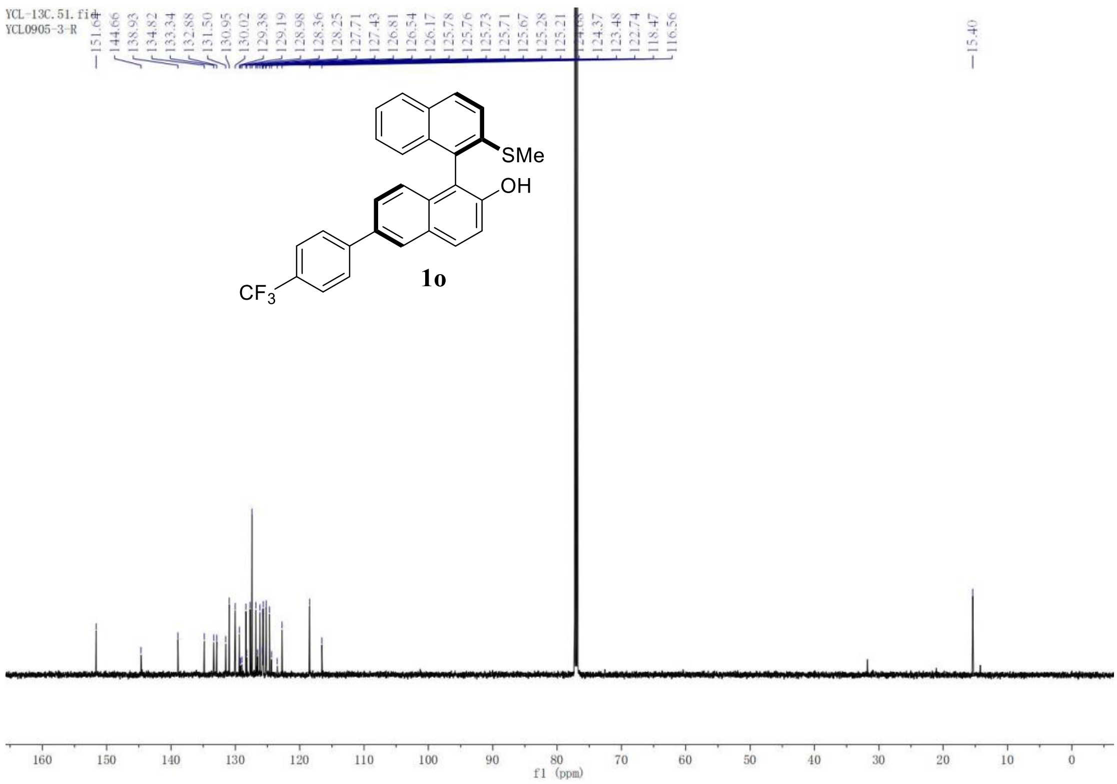
YCL-13C.51.fid
YCL0905-3-R
151.64 144.66 138.93 134.82 133.34 132.88 131.50 130.95 130.02 129.38 129.19 128.98 128.36 128.25 127.71 127.43 126.81 126.54 126.17 125.78 125.76 125.73 125.71 125.67 125.28 125.21 124.37 123.48 122.74 118.47 116.56
15.40
SMe
OH
CF3
1o
f1 (ppm)

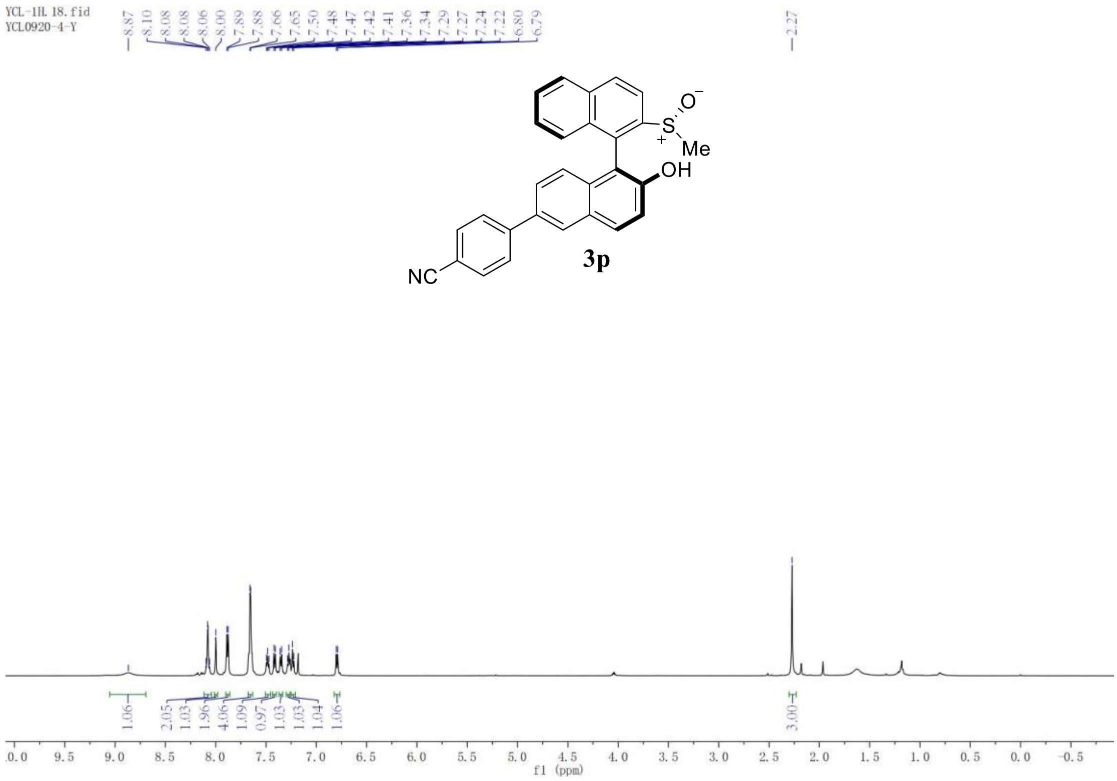
YCL-1H.18.fid
YCL0920-4-Y
8.87 8.10 8.08 8.06 8.00 7.89 7.88 7.66 7.65 7.50 7.48 7.47 7.42 7.41 7.36 7.34 7.29 7.27 7.24 7.22 6.80 6.79
2.27
O
S
Me
OH
NC
3p
1.06 2.05 1.03 1.96 4.06 1.09 0.97 1.03 1.03 1.04 1.06
3.00
f1 (ppm)

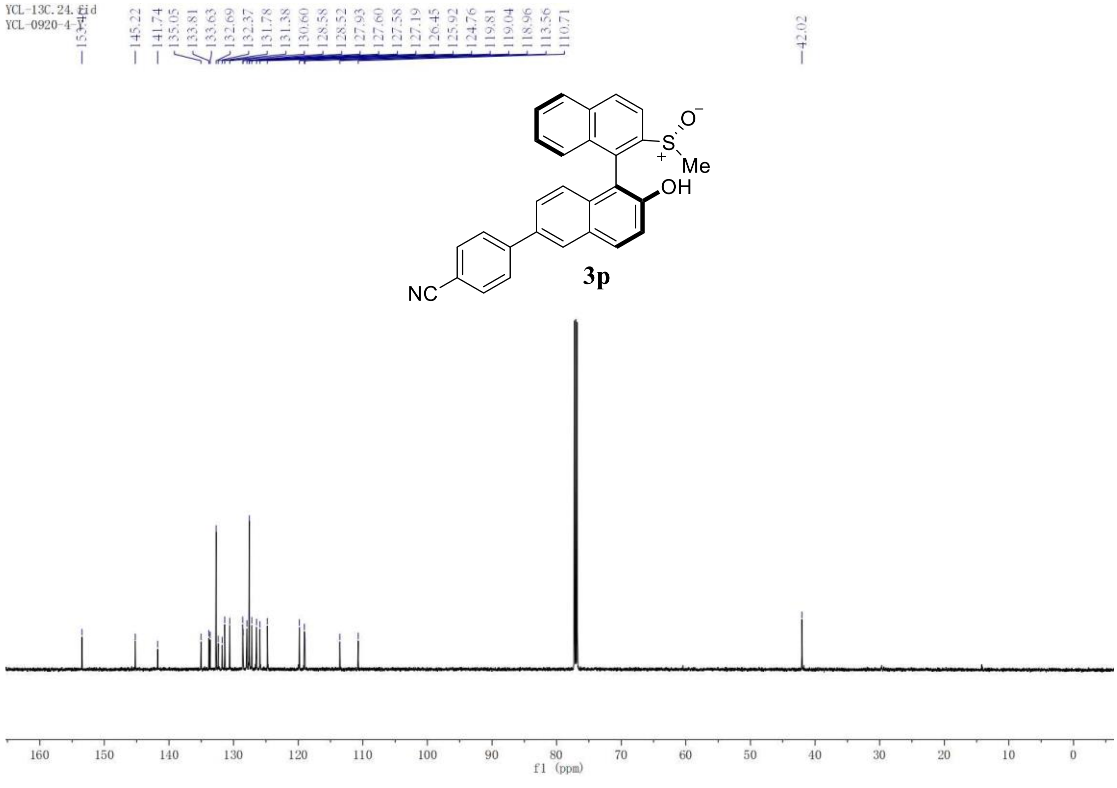
YCL-13C.24.fid
YCL-0920-4-Y
145.22 141.74 135.05 133.81 133.63 132.69 132.37 131.78 131.38 130.60 128.58 128.52 127.93 127.60 127.58 127.19 126.45 125.92 124.76 119.81 119.04 118.96 113.56 110.71
42.02
O
S
Me
OH
NC
3p
f1 (ppm)

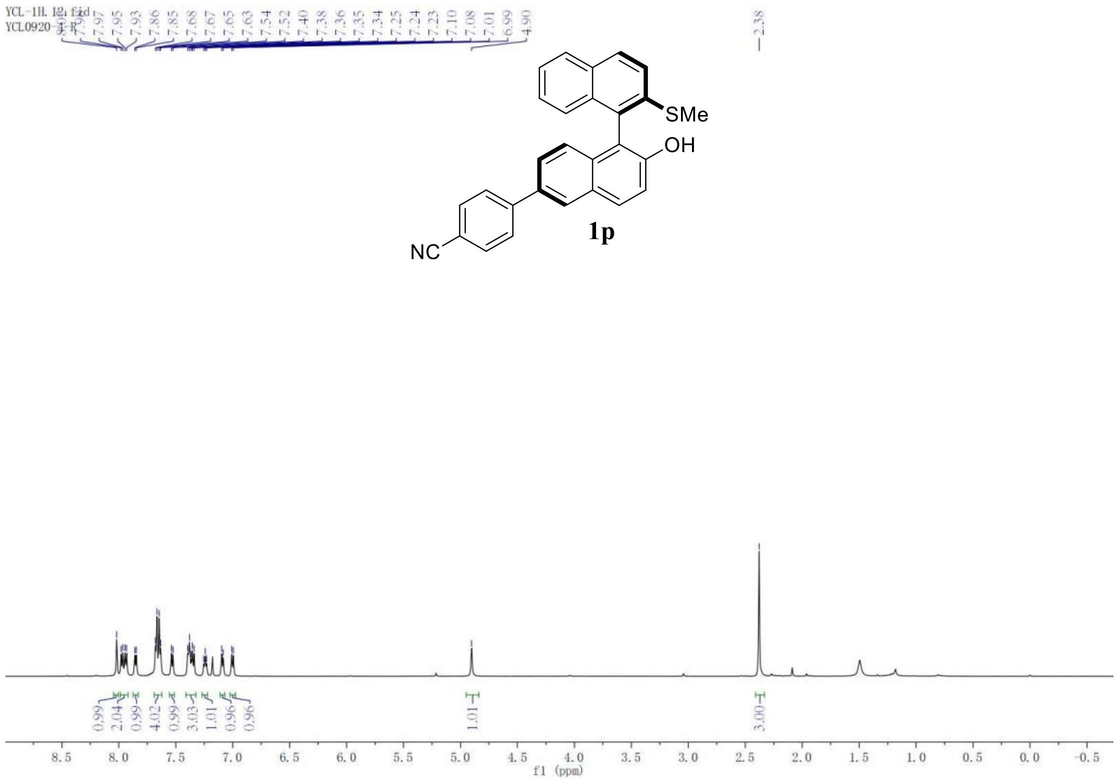
YCL-1H.12.fid
YCL0920-4-R
7.97
7.95
7.93
7.86
7.85
7.68
7.67
7.65
7.63
7.54
7.52
7.40
7.38
7.36
7.35
7.34
7.25
7.24
7.23
7.10
7.08
7.01
6.99
4.90
2.38
SMe
OH
NC
1p
0.99
2.04
0.99
0.99
4.02
0.99
3.03
1.01
0.96
0.96
1.01
3.00
f1 (ppm)

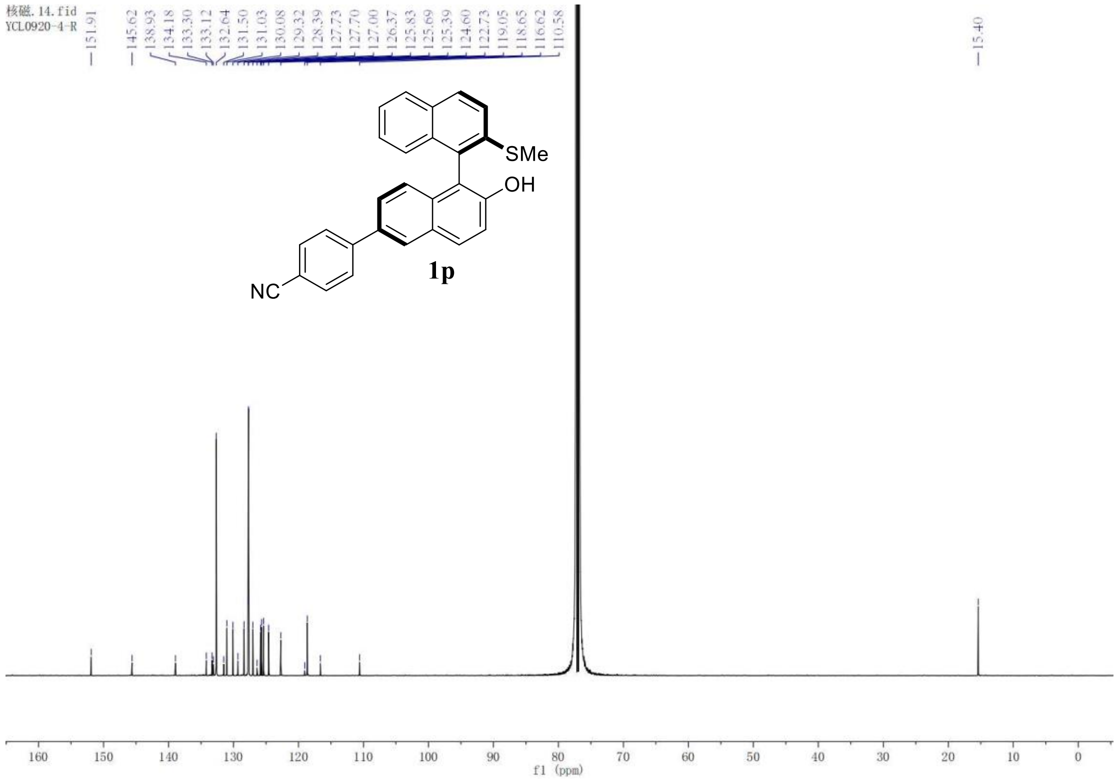
核磁.14.fid
YCL0920-4-R
151.91
145.62
138.93
134.18
133.30
133.12
132.64
131.50
131.03
130.08
129.32
128.39
127.73
127.70
127.00
126.37
125.83
125.69
125.39
124.60
122.73
119.05
118.65
116.62
110.58
15.40
SMe
OH
NC
1p
f1 (ppm)

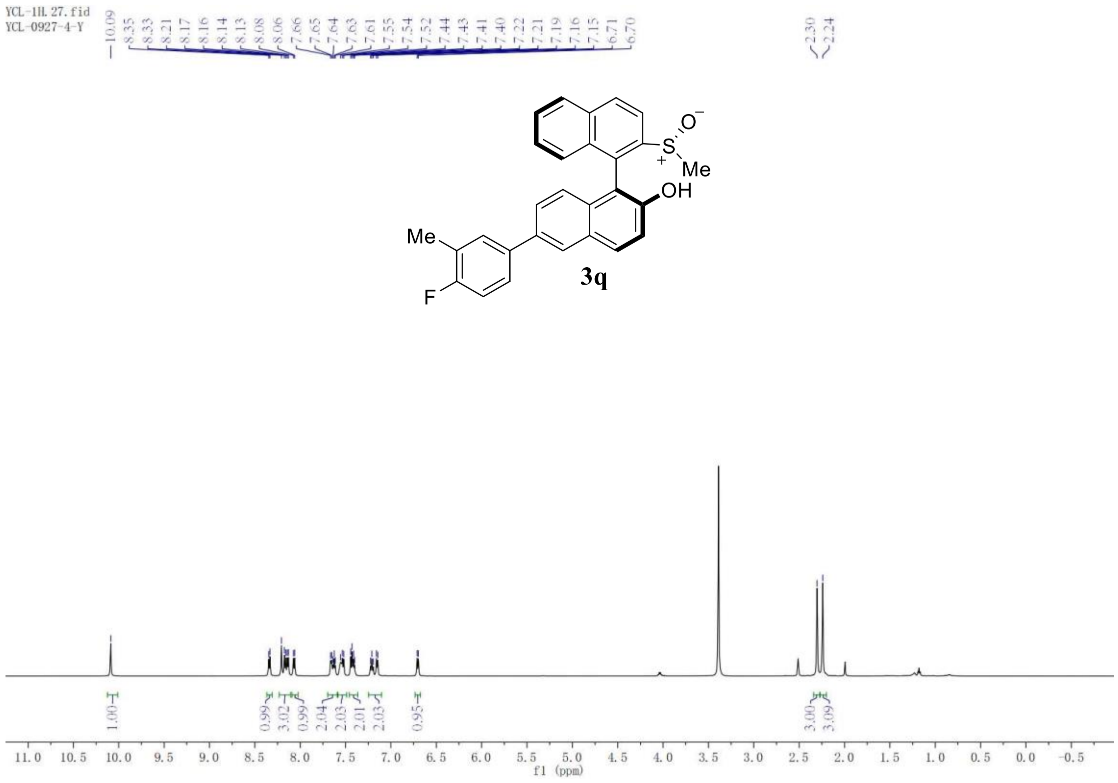
YCL-1H.27.fid
YCL-0927-4-Y
10.09
8.35 8.33 8.21 8.17 8.16 8.14 8.13 8.08 8.06 7.66 7.65 7.64 7.63 7.61 7.55 7.54 7.52 7.44 7.43 7.41 7.40 7.22 7.21 7.19 7.16 7.15 6.71 6.70
2.30 2.24
S
O
Me
OH
Me
F
3q
1.00
0.99 3.02 0.99 2.04 2.03 2.03 2.01 2.03 0.95
3.00 3.09
11.0 10.5 10.0 9.5 9.0 8.5 8.0 7.5 7.0 6.5 6.0 5.5 5.0 4.5 4.0 3.5 3.0 2.5 2.0 1.5 1.0 0.5 0.0 -0.5
f1 (ppm)

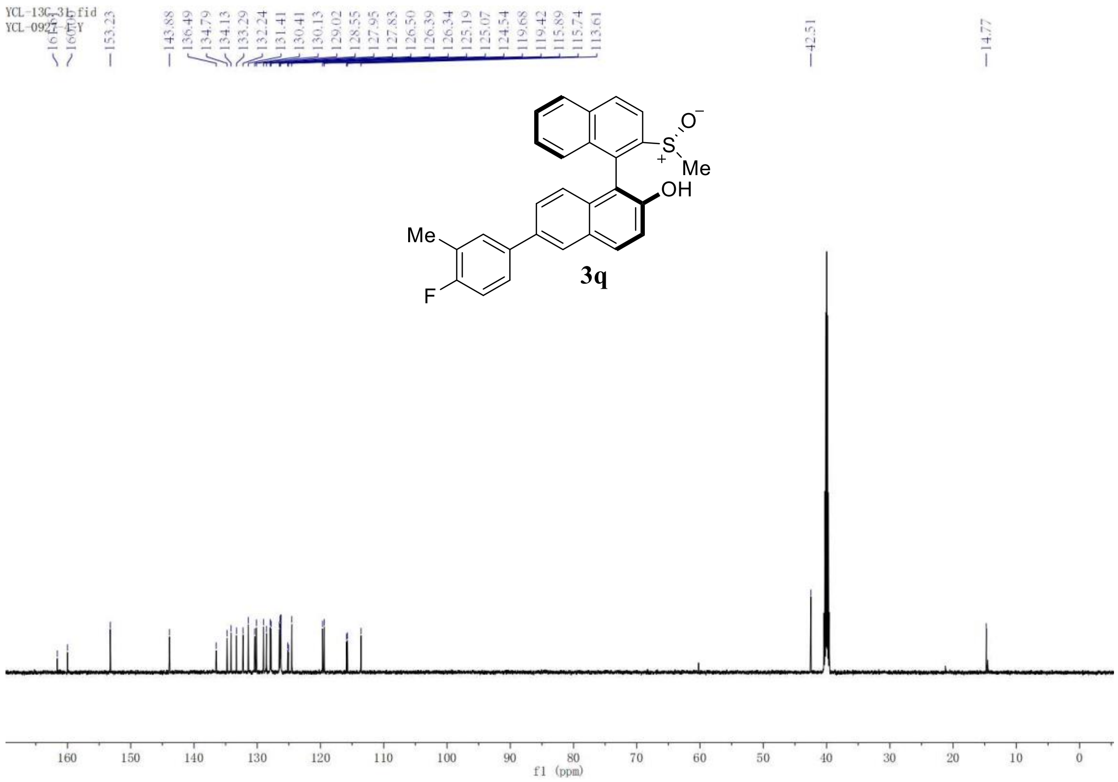
YCL-13C-31.fid
YCL-0927-4-Y
153.23
143.88 136.49 134.79 134.13 133.29 132.24 131.41 130.41 130.13 129.02 128.55 127.95 127.83 126.50 126.34 125.19 125.07 124.54 119.68 119.42 115.89 115.74 113.61
42.51
14.77
S
O
Me
OH
Me
F
3q
160 150 140 130 120 110 100 90 80 70 60 50 40 30 20 10 0
f1 (ppm)

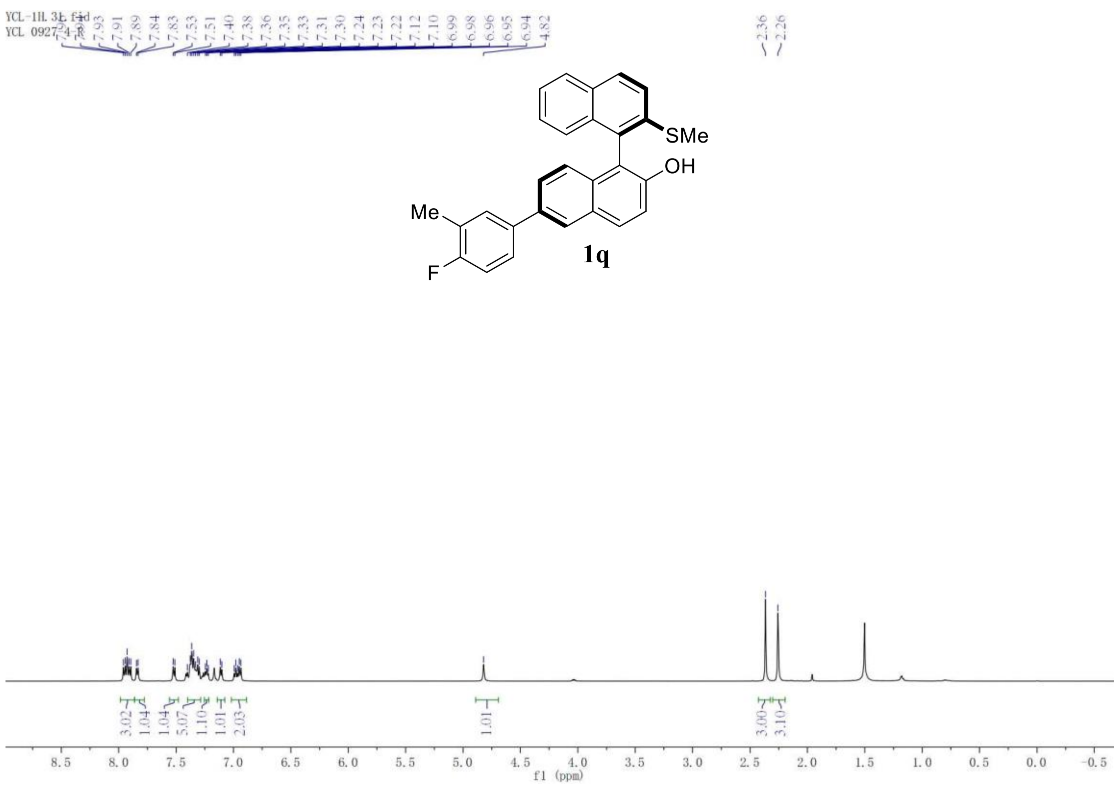
7.93
7.91
7.89
7.84
7.83
7.53
7.51
7.40
7.38
7.36
7.35
7.33
7.31
7.30
7.24
7.23
7.22
7.12
7.10
6.99
6.98
6.96
6.95
6.94
4.82
2.36
2.26
SMe
OH
Me
F
1q
3.02
1.04
1.04
5.07
1.10
1.01
2.03
1.01
3.00
3.10
f1 (ppm)

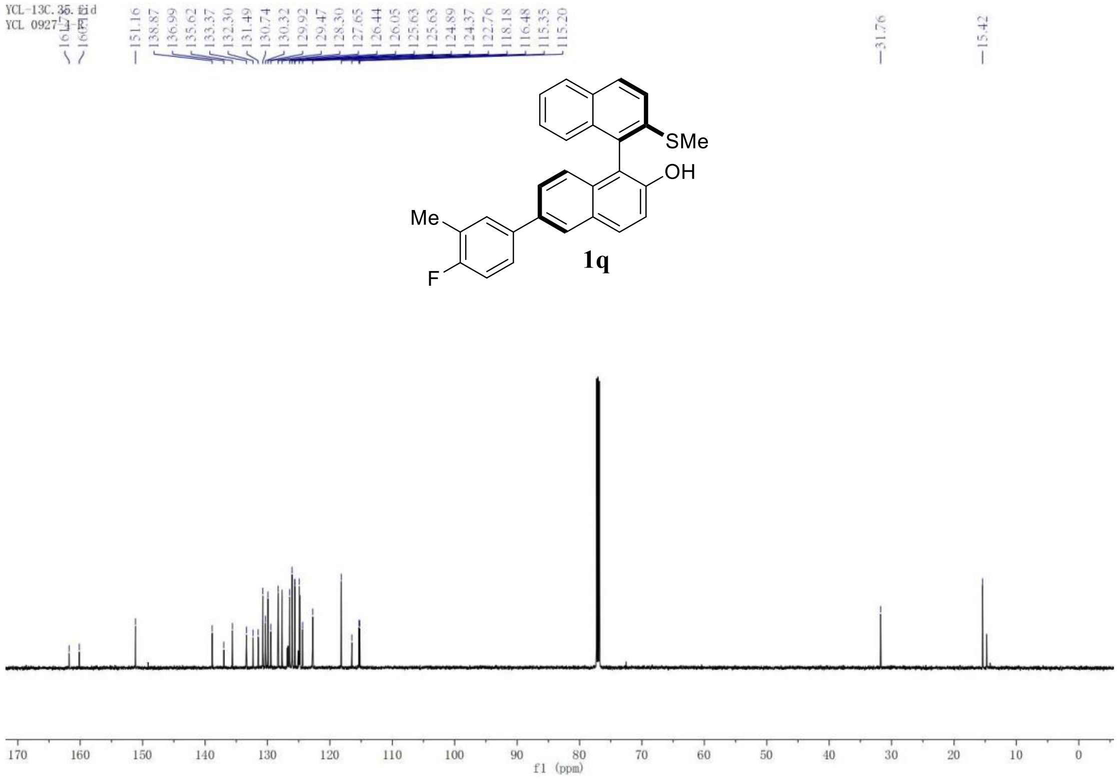
151.16
138.87
136.99
135.62
133.37
132.30
131.49
130.74
130.32
129.92
129.47
128.30
127.65
126.44
126.05
125.63
124.89
124.37
122.76
118.18
116.48
115.35
115.20
31.76
15.42
SMe
OH
Me
F
1q
f1 (ppm)

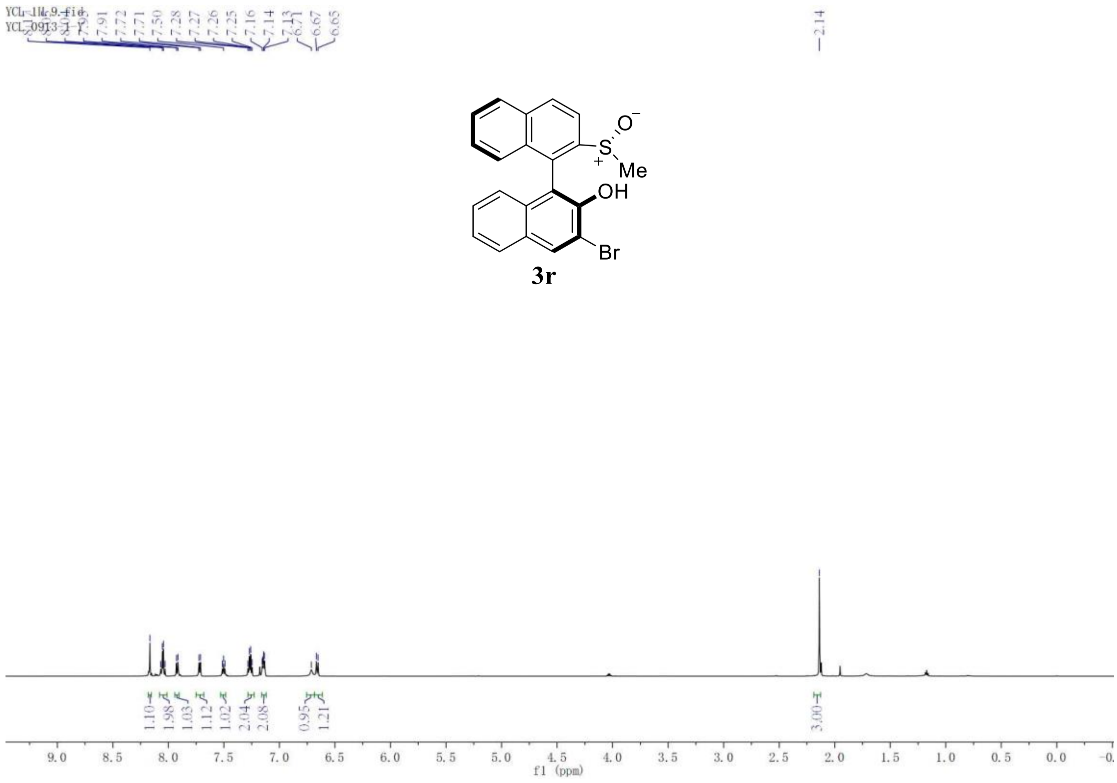
YCL-1H.9.fid
YCL-0913-1-Y
7.91 7.72 7.71 7.50 7.28 7.27 7.26 7.25 7.16 7.14 7.13 6.71 6.67 6.65
2.14
O⁻
S
+
Me
OH
Br
3r
1.10 1.98 1.03 1.12 1.02 2.04 2.08 0.95 1.21
3.00
9.0 8.5 8.0 7.5 7.0 6.5 6.0 5.5 5.0 4.5 4.0 3.5 3.0 2.5 2.0 1.5 1.0 0.5 0.0
f1 (ppm)

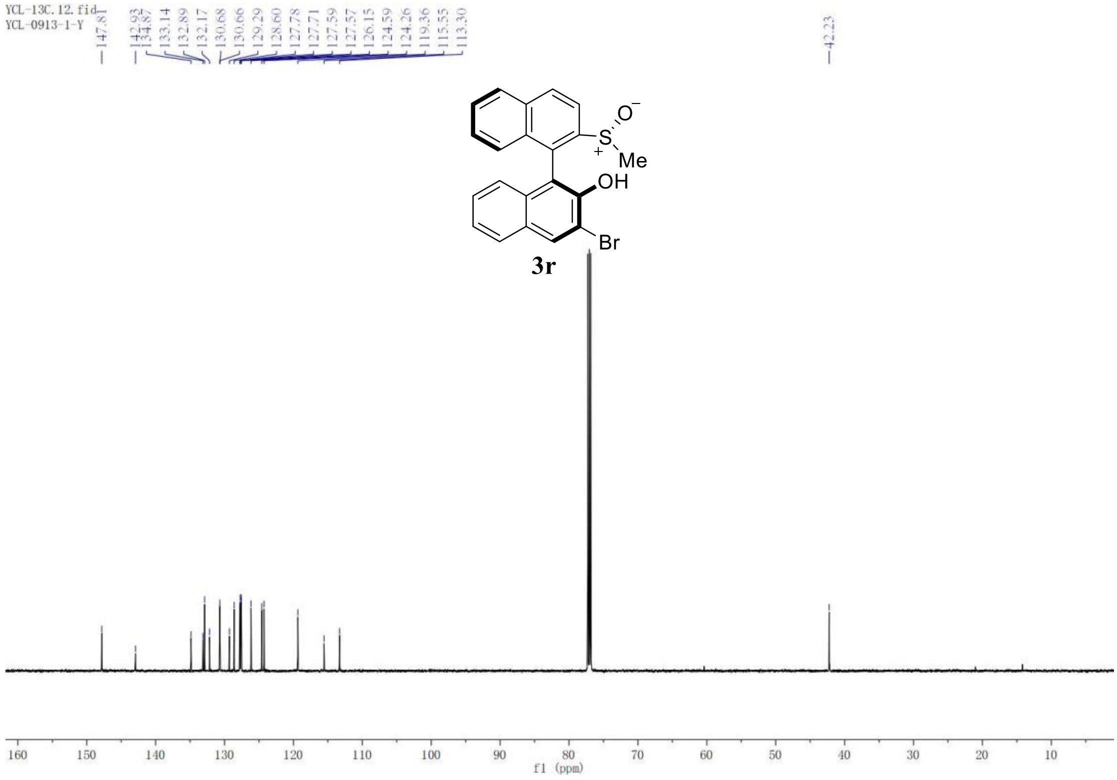
YCL-13C.12.fid
YCL-0913-1-Y
147.81
142.93 134.87 133.14 132.89 132.17 130.68 130.66 129.29 128.60 127.78 127.71 127.59 127.57 126.15 124.59 124.26 119.36 115.55 113.30
42.23
O⁻
S
+
Me
OH
Br
3r
160 150 140 130 120 110 100 90 80 70 60 50 40 30 20 10
f1 (ppm)

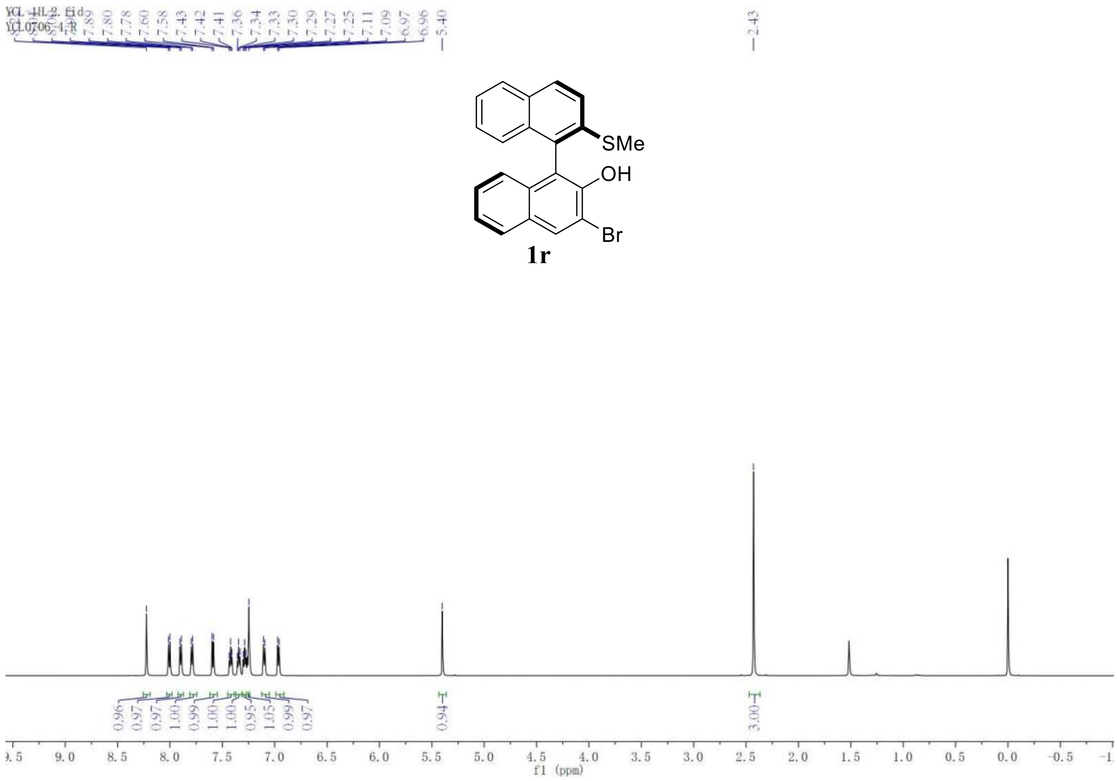
SMe
OH
Br
1r
7.89 7.80 7.78 7.60 7.58 7.43 7.42 7.41 7.36 7.34 7.33 7.30 7.29 7.27 7.25 7.11 7.09 6.97 6.96
5.40
2.43
0.96 0.97 0.97 1.00 0.99 1.00 1.00 0.95 1.05 0.99 0.97
0.94
3.00
f1 (ppm)

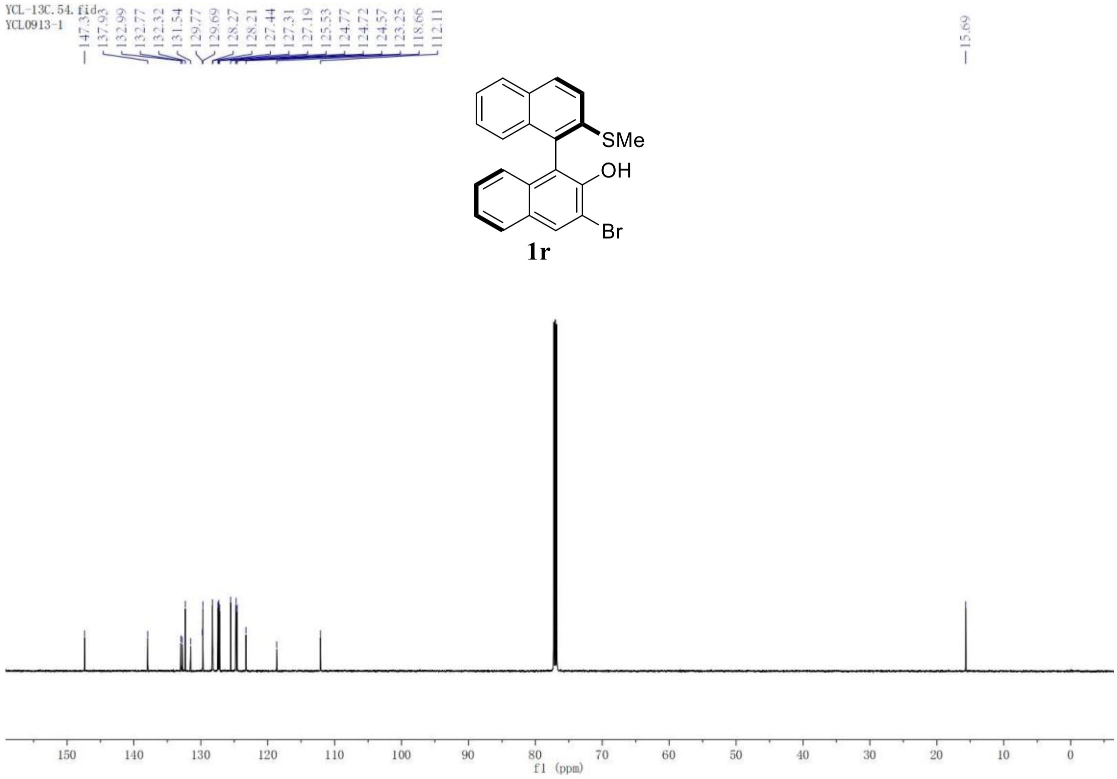
YCL-13C.54.fid
YCL0913-1
SMe
OH
Br
1r
132.99 132.77 132.32 131.54 129.77 129.69 128.27 128.21 127.44 127.31 127.19 125.53 124.77 124.72 124.57 123.25 118.66 112.11
15.69
f1 (ppm)

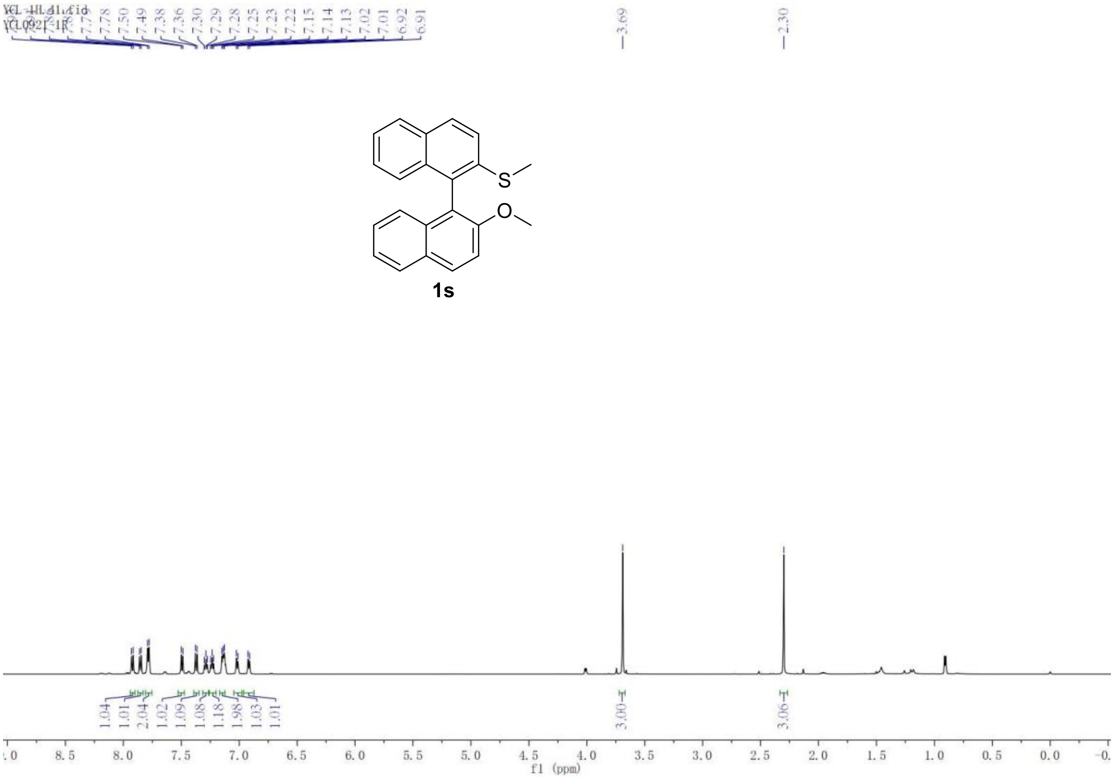
YCL-1H.41.fid
YCL0921-1H
7.78 7.50 7.49 7.38 7.36 7.30 7.29 7.28 7.25 7.23 7.22 7.15 7.14 7.13 7.02 7.01 6.92 6.91
3.69
2.30
S
O
1s
1.04 1.01 2.04 1.02 1.09 1.08 1.18 1.98 1.03 1.01
3.00
3.06
8.5 8.0 7.5 7.0 6.5 6.0 5.5 5.0 4.5 4.0 3.5 3.0 2.5 2.0 1.5 1.0 0.5 0.0
f1 (ppm)

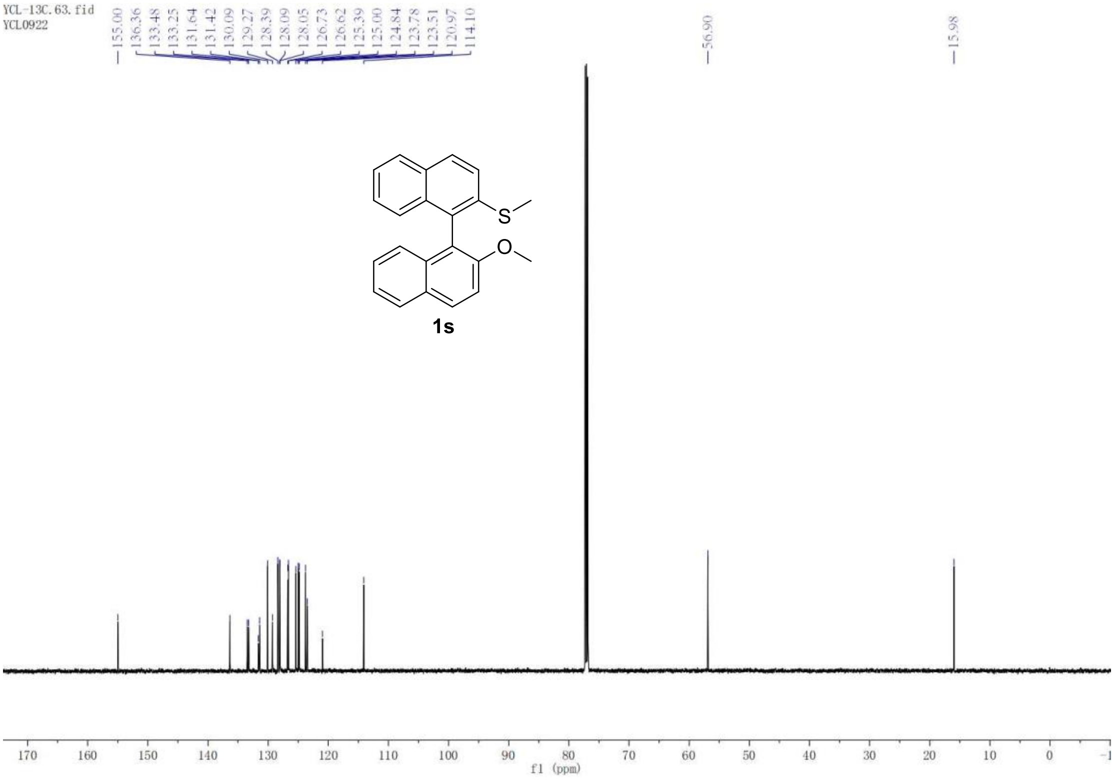
YCL-13C.63.fid
YCL0922
155.00 136.36 133.48 133.25 131.64 131.42 130.09 129.27 128.39 128.09 128.05 126.73 126.62 125.39 125.00 124.84 123.78 123.51 120.97 114.10
56.90
15.98
S
O
1s
170 160 150 140 130 120 110 100 90 80 70 60 50 40 30 20 10 0
f1 (ppm)

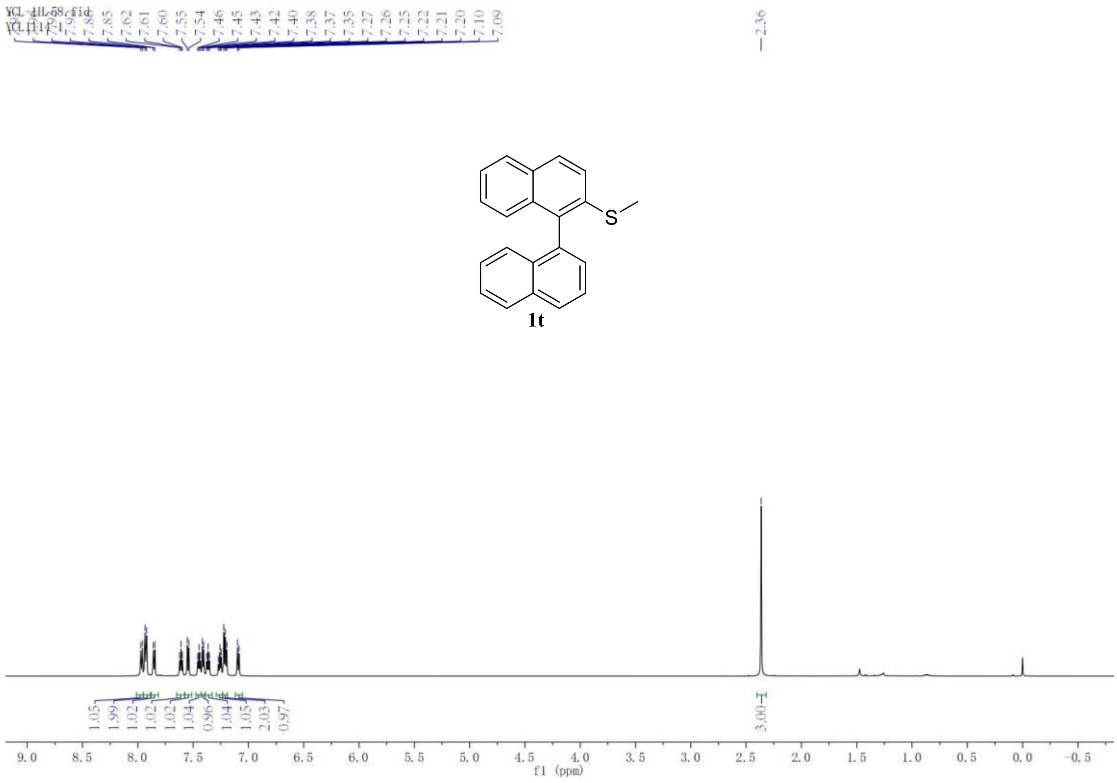
1t
2.36
3.00
f1 (ppm)

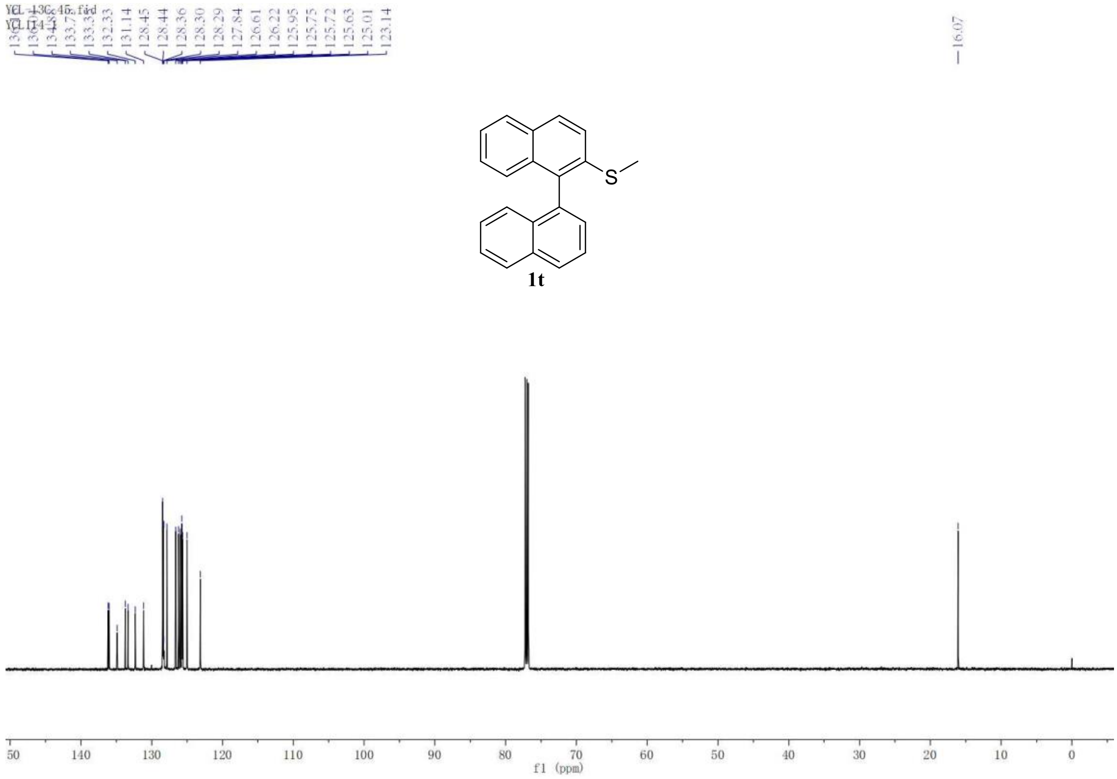
1t
16.07
f1 (ppm)

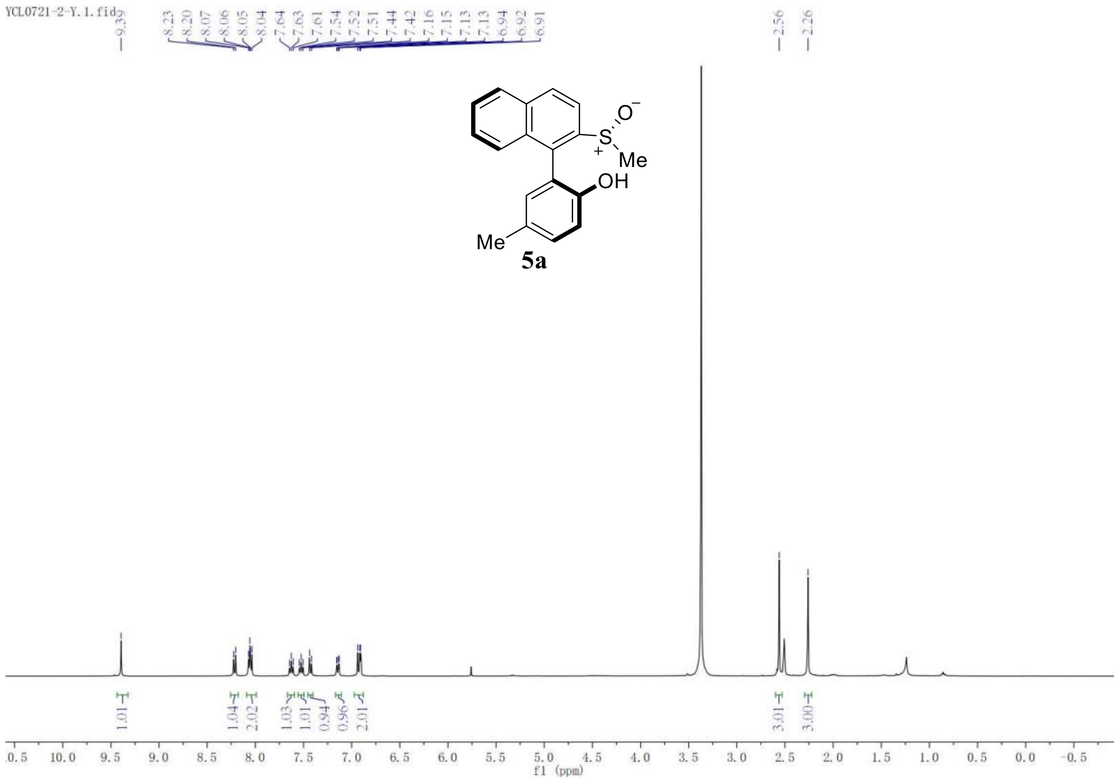
YCL0721-2-Y.1.fid
9.39
8.23
8.20
8.07
8.06
8.05
8.04
7.64
7.63
7.61
7.54
7.52
7.51
7.44
7.42
7.16
7.15
7.13
7.13
6.94
6.92
6.91
2.56
2.26
Me
OH
Me
5a
1.01
1.04
2.02
1.03
1.01
0.94
0.96
2.01
3.01
3.00
f1 (ppm)

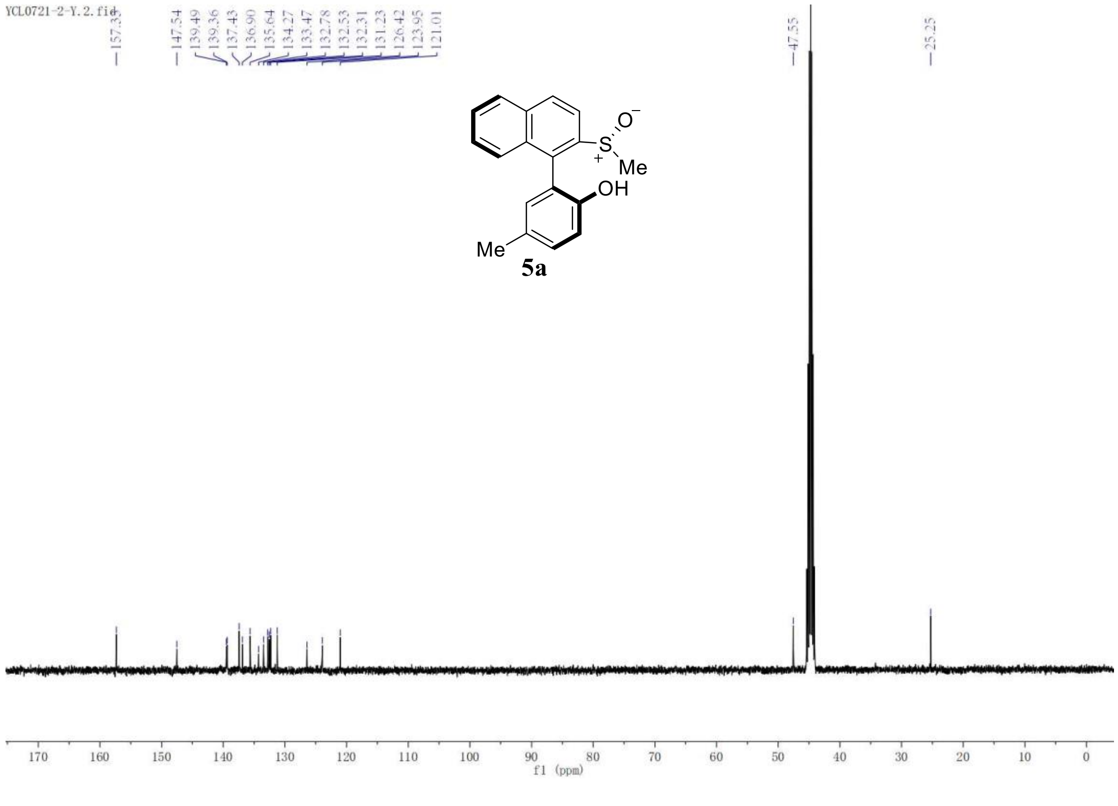
YCL0721-2-Y.2.fid
157.35
147.54
139.49
139.36
137.43
136.90
135.64
134.27
133.47
132.78
132.53
132.31
131.23
126.42
123.95
121.01
47.55
25.25
Me
OH
Me
5a
f1 (ppm)

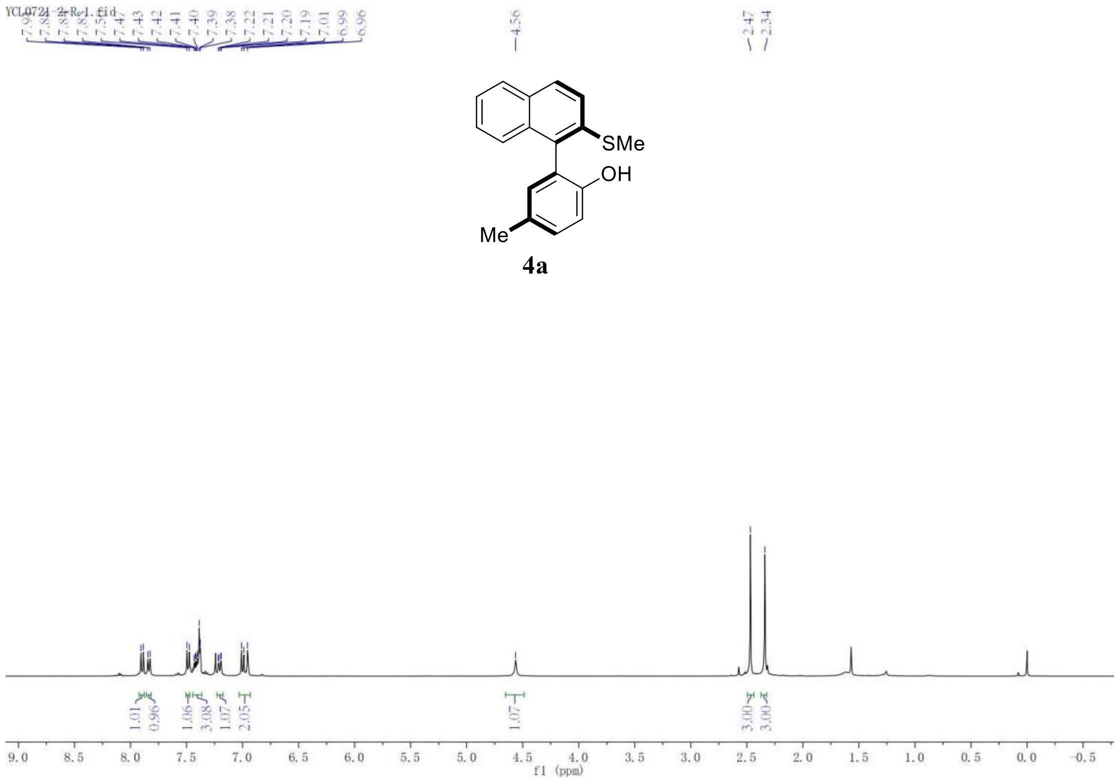
4.56
2.47
2.34
SMe
OH
Me
4a
1.01
0.96
1.06
3.08
1.07
2.05
1.07
3.00
3.00
f1 (ppm)

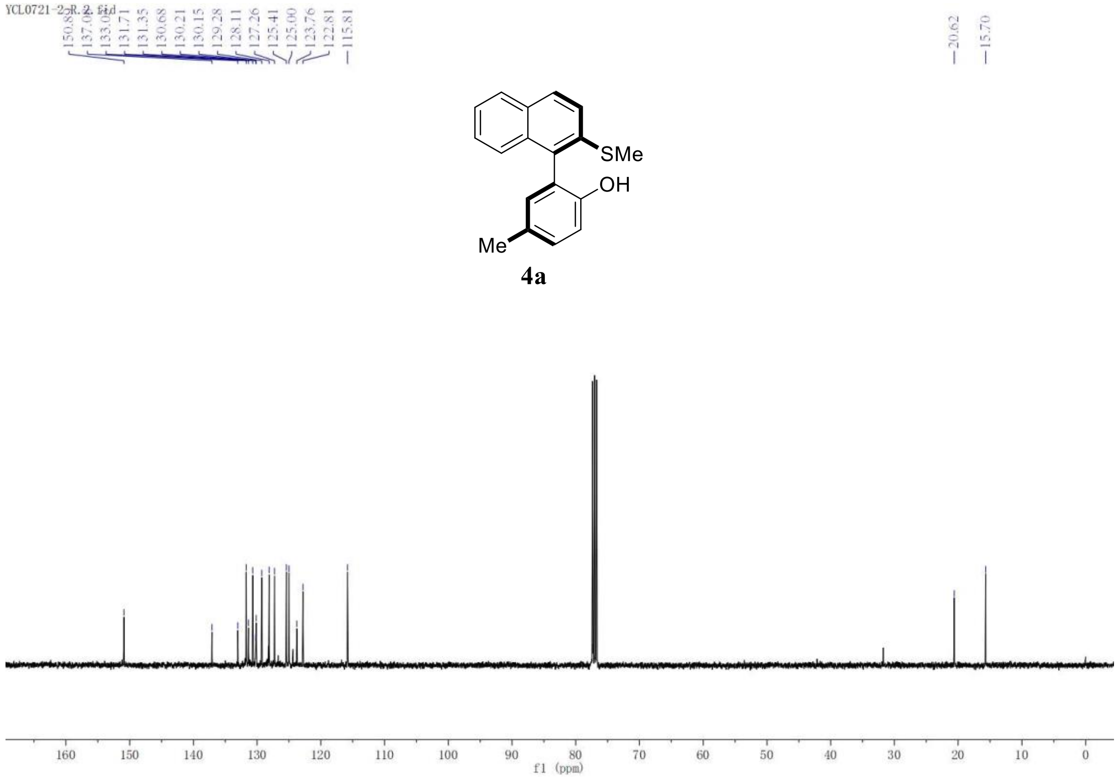
YCL0721-2-R.2.fid
131.71
131.35
130.68
130.21
130.15
129.28
128.11
127.26
125.41
125.00
123.76
122.81
115.81
20.62
15.70
SMe
OH
Me
4a
f1 (ppm)

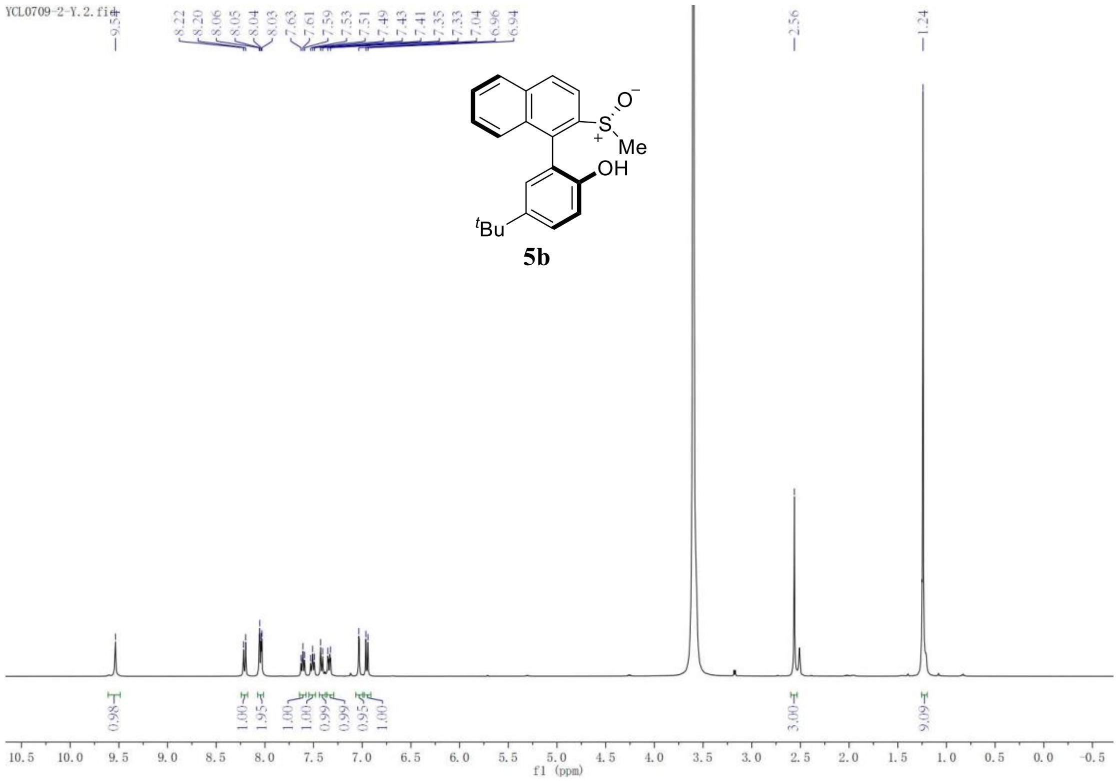
YCL0709-2-Y.2.fid
9.54
8.22
8.20
8.06
8.05
8.04
8.03
7.63
7.61
7.59
7.53
7.51
7.49
7.43
7.41
7.35
7.33
7.04
6.96
6.94
2.56
1.24
O
S
Me
OH
tBu
5b
0.98
1.00
1.95
1.00
1.00
0.99
0.99
0.95
1.00
3.00
9.09
f1 (ppm)

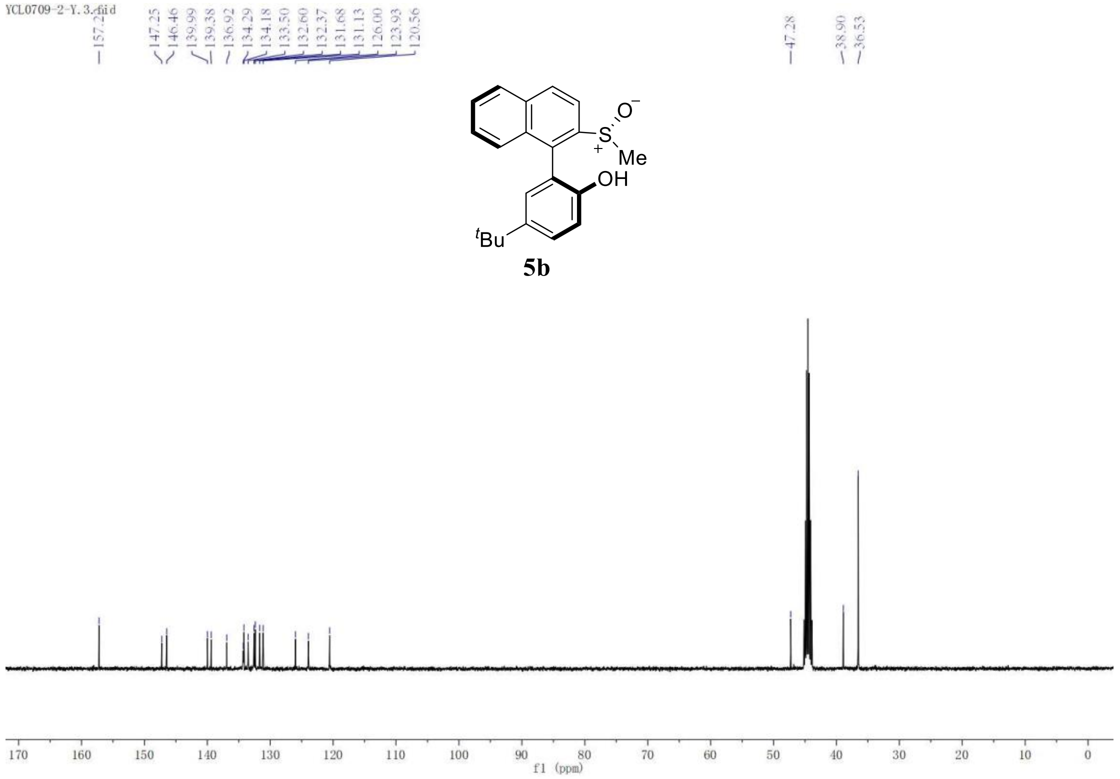
YCL0709-2-Y.3.fid
157.21
147.25
146.46
139.99
139.38
136.92
134.29
134.18
133.50
132.60
132.37
131.68
131.13
126.00
123.93
120.56
47.28
38.90
36.53
O
S
Me
OH
tBu
5b
f1 (ppm)

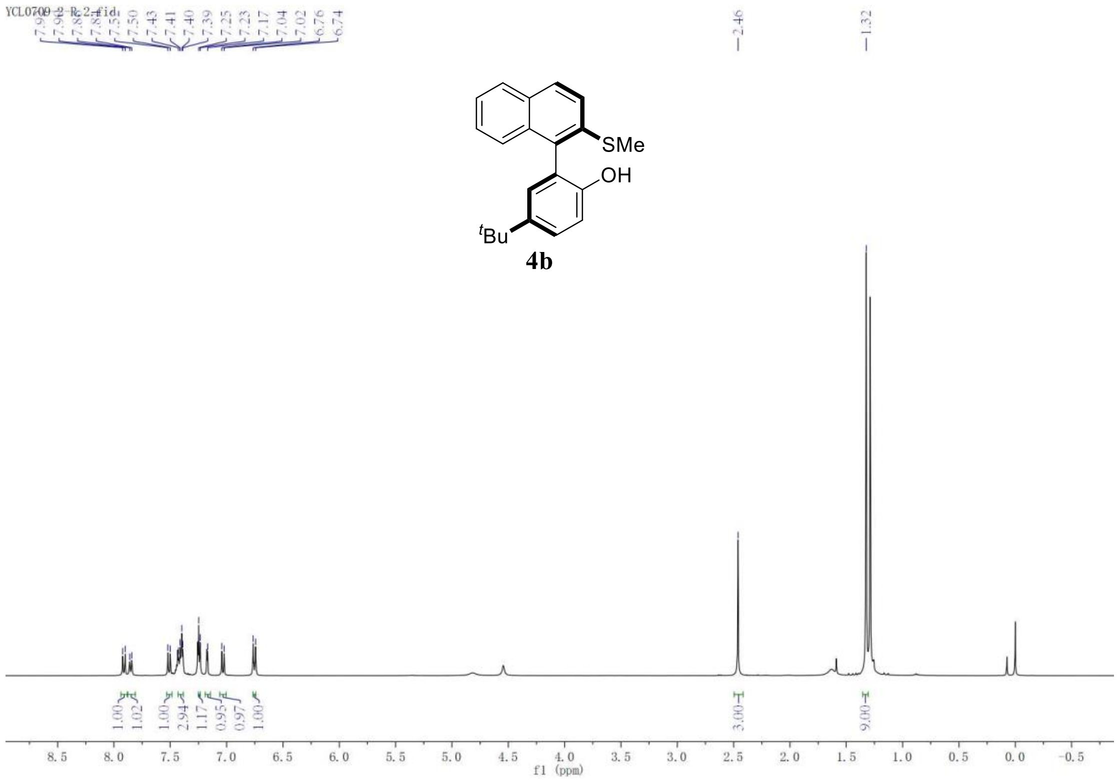
YCL0709-2-R.2.fid
7.50
7.43
7.41
7.40
7.39
7.25
7.23
7.17
7.04
7.02
6.76
6.74
2.46
1.32
SMe
OH
tBu
4b
1.00
1.02
1.00
2.94
1.17
0.95
0.97
1.00
3.00
9.00
f1 (ppm)

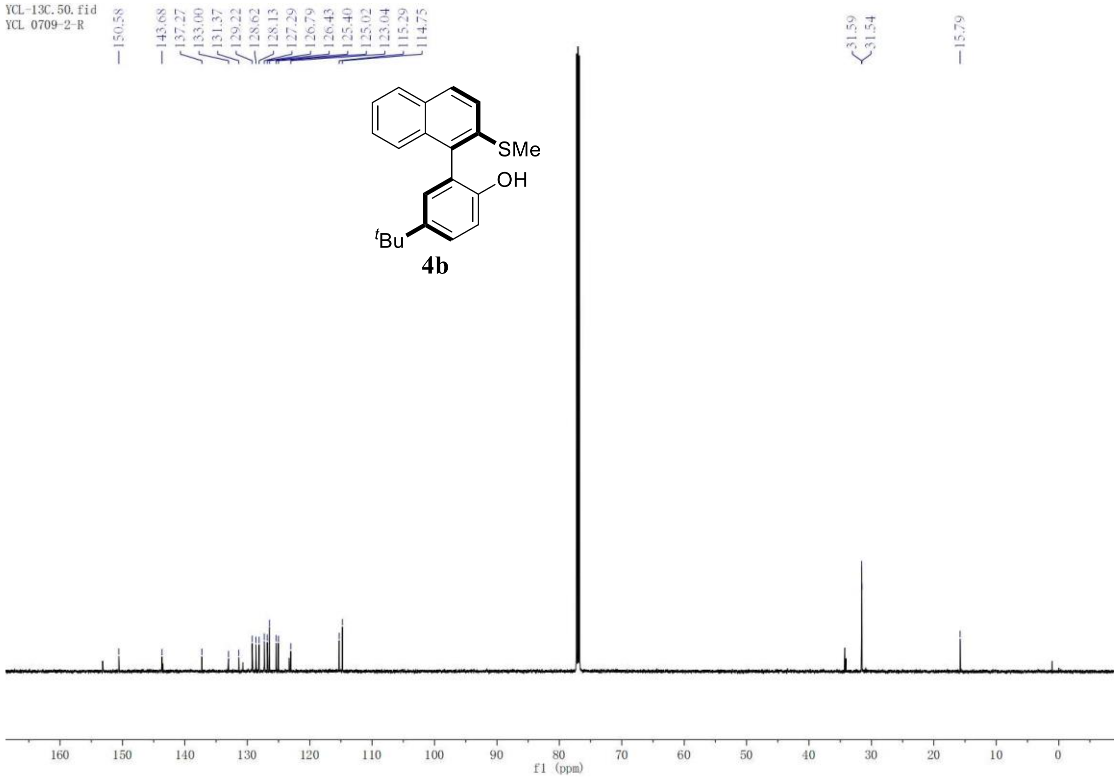
YCL-13C.50.fid
YCL 0709-2-R
150.58
143.68
137.27
133.00
131.37
129.22
128.62
128.13
127.29
126.79
126.43
125.40
125.02
123.04
115.29
114.75
31.59
31.54
15.79
SMe
OH
tBu
4b
f1 (ppm)

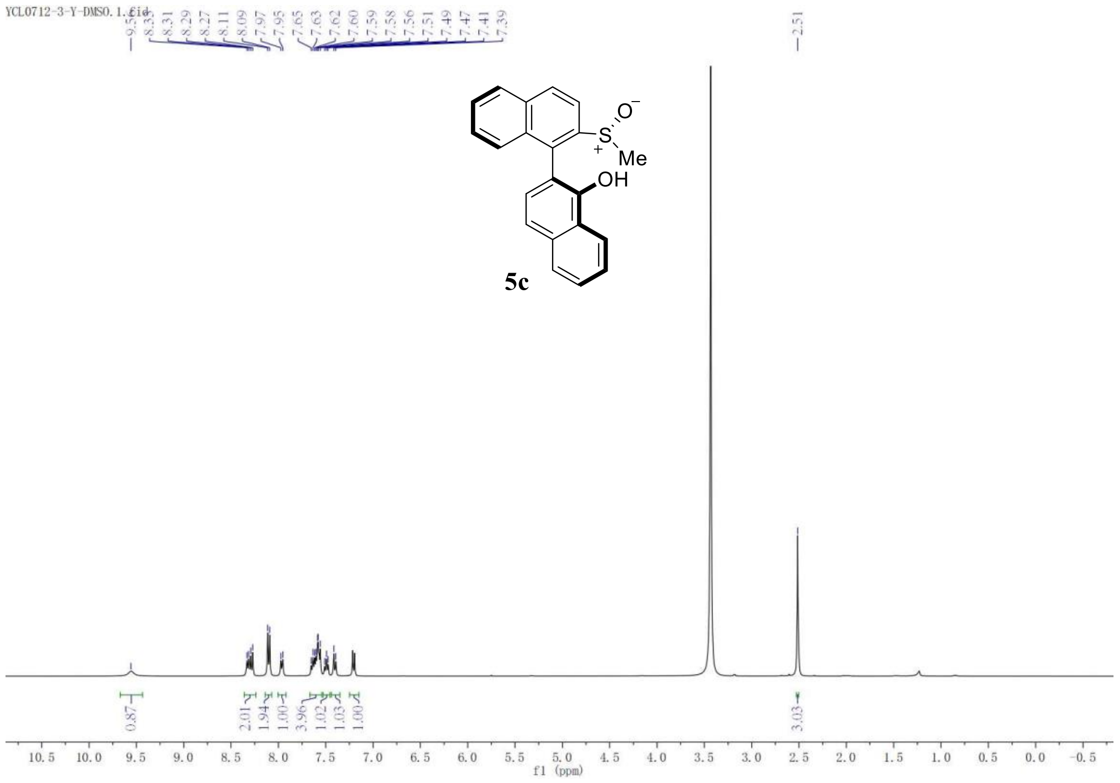
YCL0712-3-Y-DMSO.1.fid
8.33
8.31
8.29
8.27
8.11
8.09
7.97
7.95
7.65
7.63
7.62
7.60
7.59
7.58
7.56
7.51
7.49
7.47
7.41
7.39
2.51
O⁻
S
+
Me
OH
5c
0.87
2.01
1.94
1.00
3.96
1.02
1.03
1.00
3.03
10.5 10.0 9.5 9.0 8.5 8.0 7.5 7.0 6.5 6.0 5.5 5.0 4.5 4.0 3.5 3.0 2.5 2.0 1.5 1.0 0.5 0.0 -0.5
f1 (ppm)

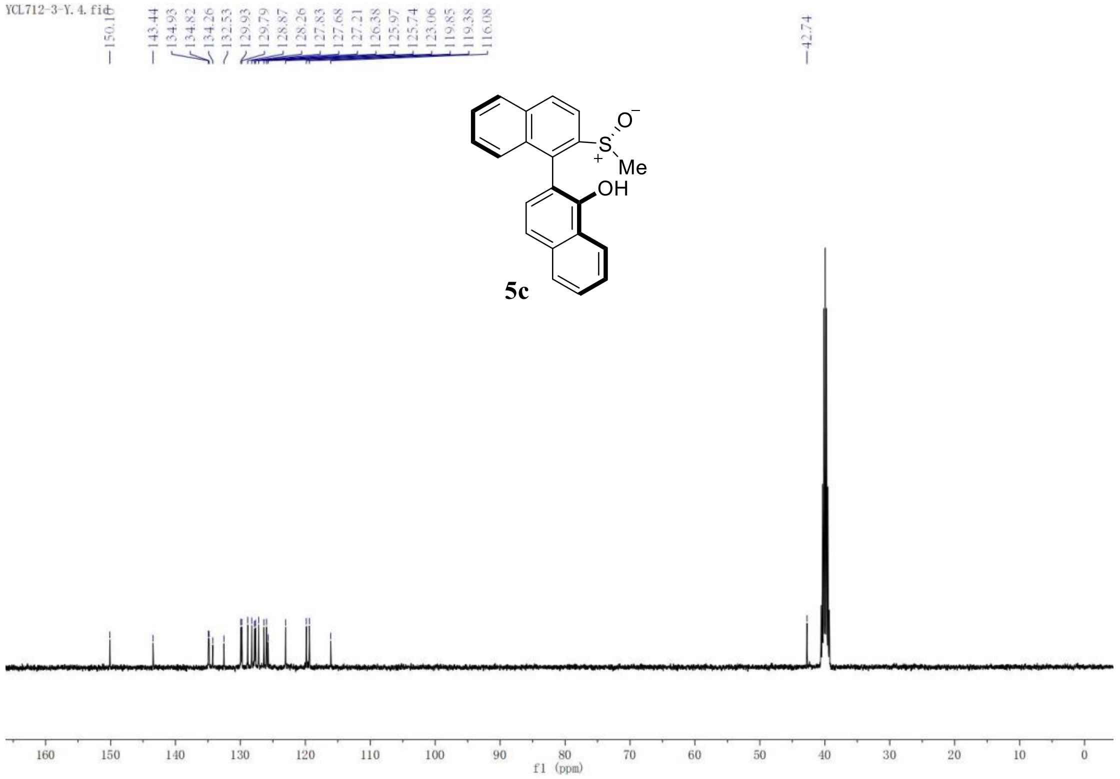
YCL712-3-Y.4.fid
150.10
143.44
134.93
134.82
134.26
132.53
129.93
129.79
128.87
128.26
127.83
127.68
127.21
126.38
125.97
125.74
123.06
119.85
119.38
116.08
42.74
O⁻
S
+
Me
OH
5c
160 150 140 130 120 110 100 90 80 70 60 50 40 30 20 10 0
f1 (ppm)

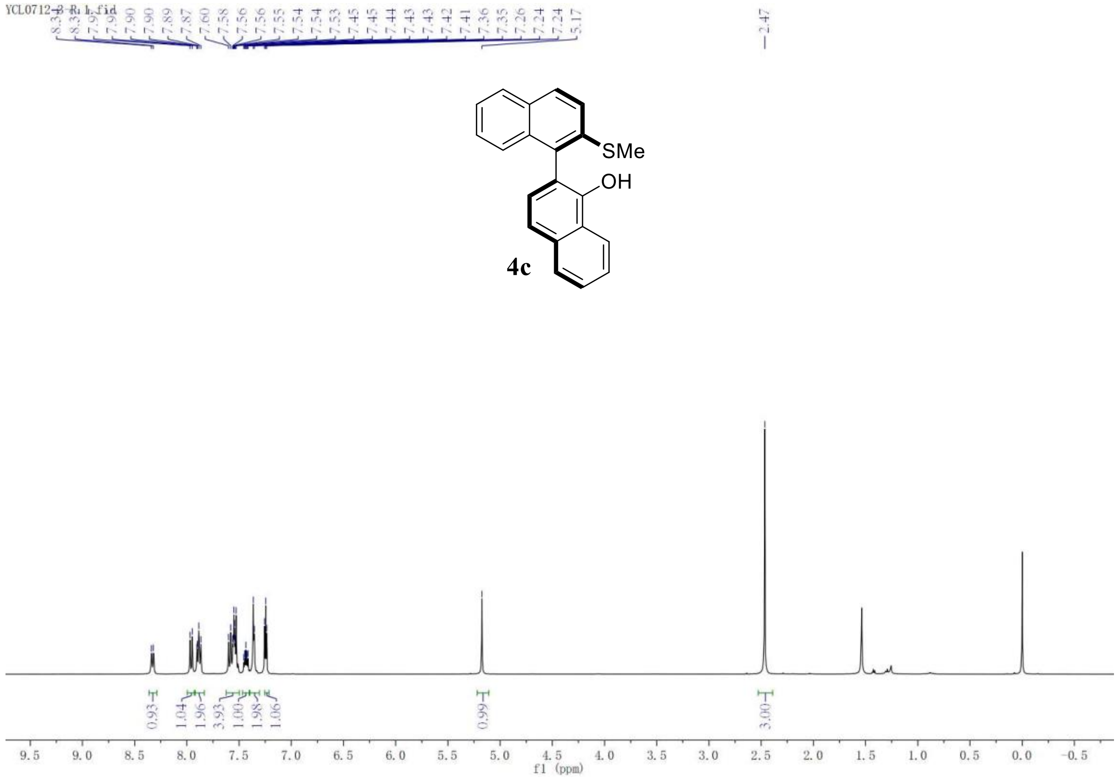
YCL0712-3-R.1.fid
2.47
SMe
OH
4c
0.93
1.04
1.96
3.93
1.00
1.98
1.06
0.99
3.00
f1 (ppm)

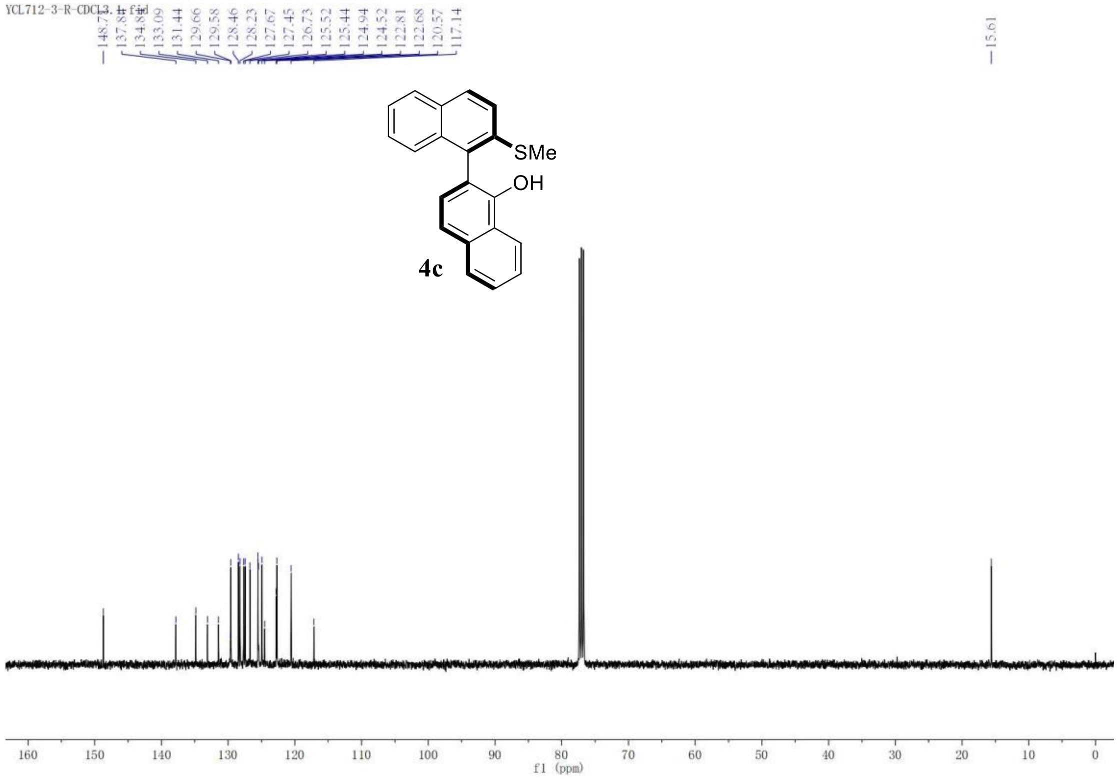
YCL712-3-R-CDCL3.1.fid
148.7
137.8
134.8
133.09
131.44
129.66
129.58
128.46
128.23
127.67
127.45
126.73
125.52
125.44
124.94
124.52
122.81
122.68
120.57
117.14
15.61
SMe
OH
4c
f1 (ppm)

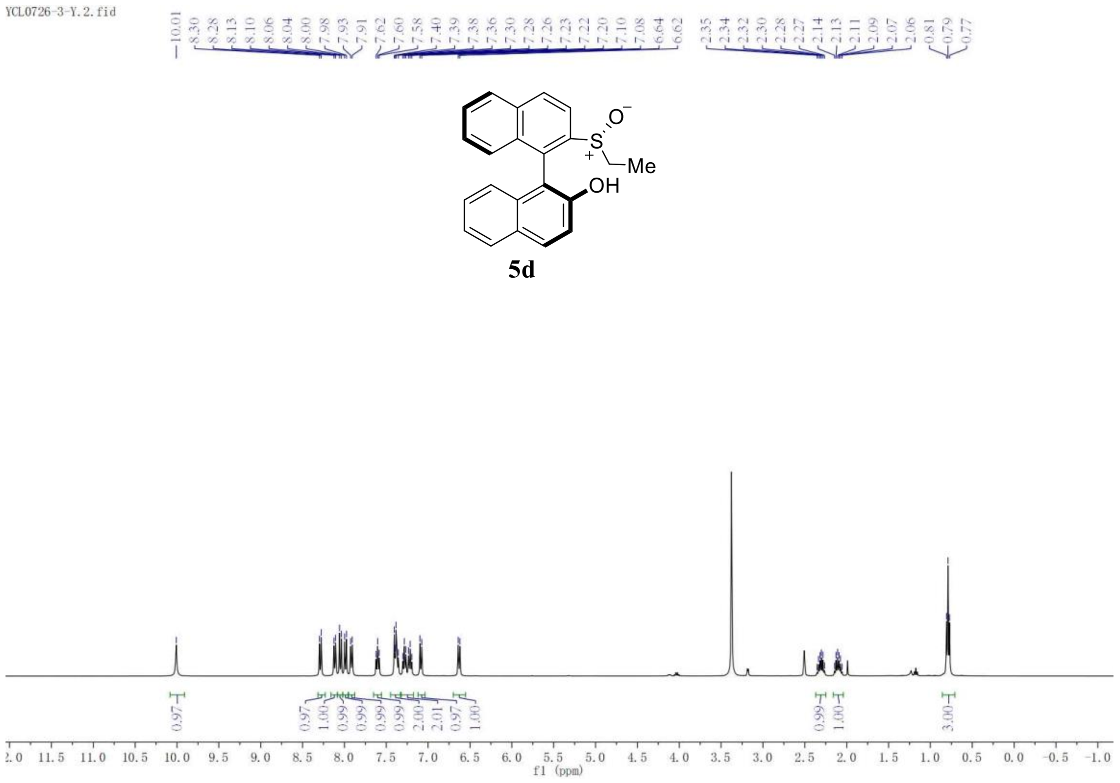
YCL0726-3-Y.2.fid
10.01 8.30 8.28 8.13 8.10 8.06 8.04 8.00 7.98 7.93 7.91 7.62 7.60 7.58 7.40 7.39 7.36 7.30 7.28 7.26 7.23 7.22 7.20 7.10 7.08 6.64 6.62 2.35 2.34 2.32 2.30 2.28 2.27 2.14 2.13 2.11 2.09 2.07 2.06 0.81 0.79 0.77
O
S
+
Me
OH
5d
0.97 0.97 1.00 0.99 0.99 0.99 0.99 2.00 2.01 0.97 1.00 0.99 1.00 3.00
f1 (ppm)

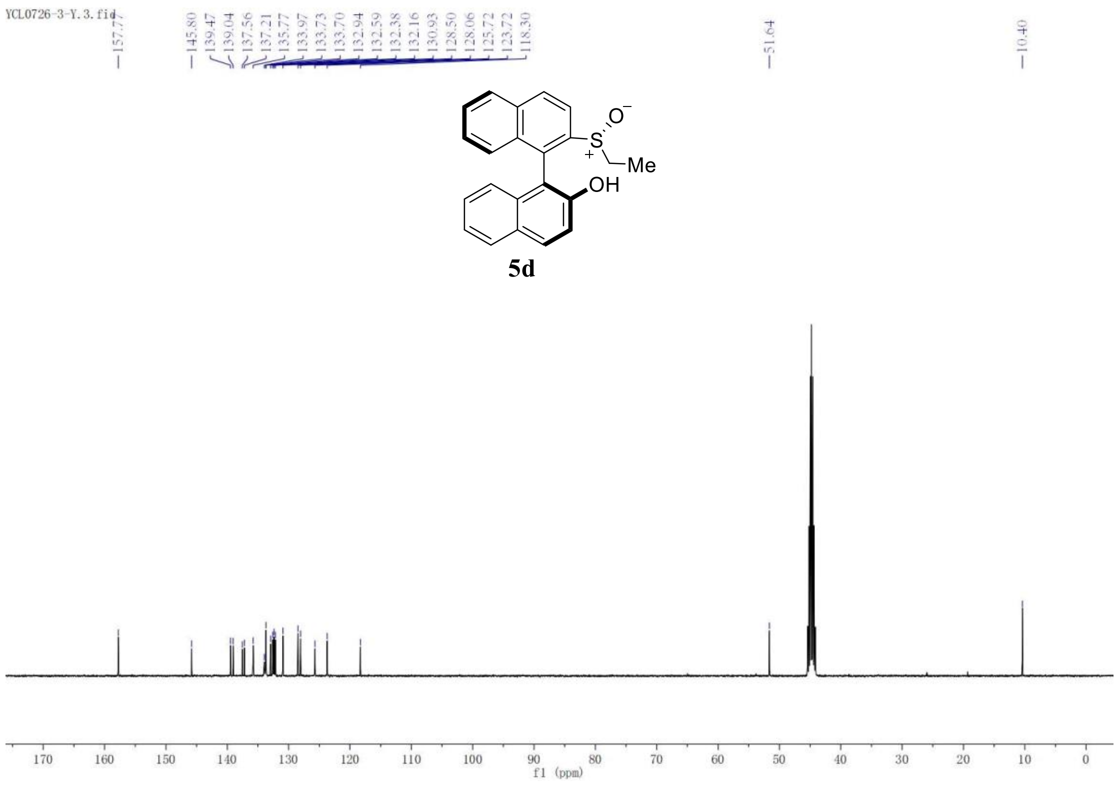
YCL0726-3-Y.3.fid
157.77 145.80 139.47 139.04 137.56 137.21 135.77 133.97 133.73 133.70 132.94 132.59 132.38 132.16 130.93 128.50 128.06 125.72 123.72 118.30 51.64 10.40
O
S
+
Me
OH
5d
f1 (ppm)

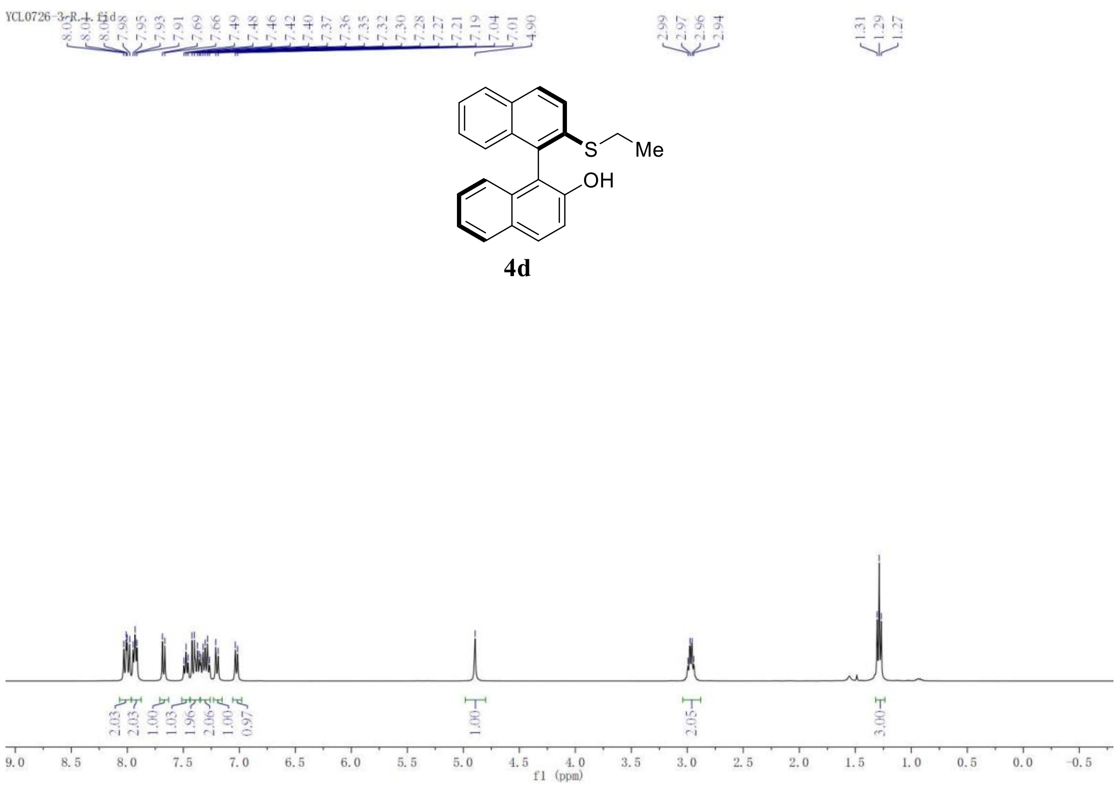
YCL0726-3-R.1.fid
7.98
7.95
7.93
7.91
7.69
7.66
7.49
7.48
7.46
7.42
7.40
7.37
7.36
7.35
7.32
7.30
7.28
7.27
7.21
7.19
7.04
7.01
4.90
2.99
2.97
2.96
2.94
1.31
1.29
1.27
S
Me
OH
2.03
2.03
1.00
1.03
1.96
2.06
1.00
0.97
1.00
2.05
3.00
9.0 8.5 8.0 7.5 7.0 6.5 6.0 5.5 5.0 4.5 4.0 3.5 3.0 2.5 2.0 1.5 1.0 0.5 0.0 -0.5
f1 (ppm)

4d

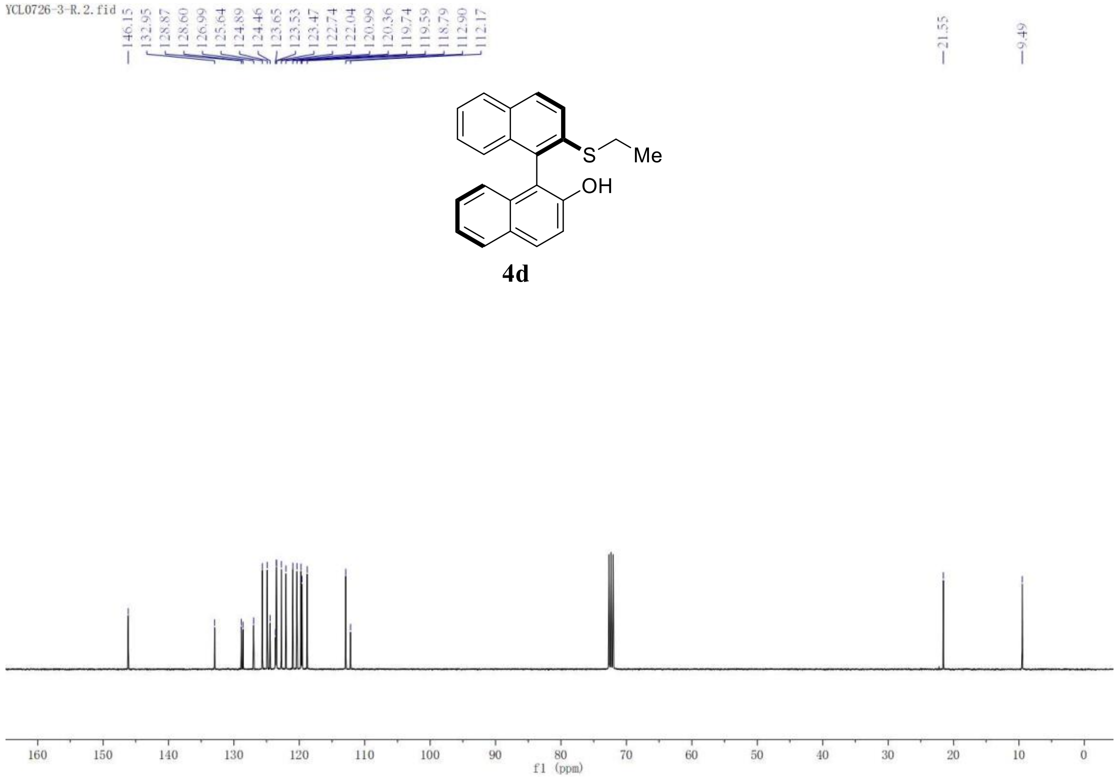
YCL0726-3-R.2.fid
146.15
132.95
128.87
128.60
126.99
125.64
124.89
124.46
123.65
123.53
123.47
122.74
122.04
120.99
120.36
119.74
119.59
118.79
112.90
112.17
21.55
9.49
S
Me
OH
160 150 140 130 120 110 100 90 80 70 60 50 40 30 20 10 0
f1 (ppm)

4d

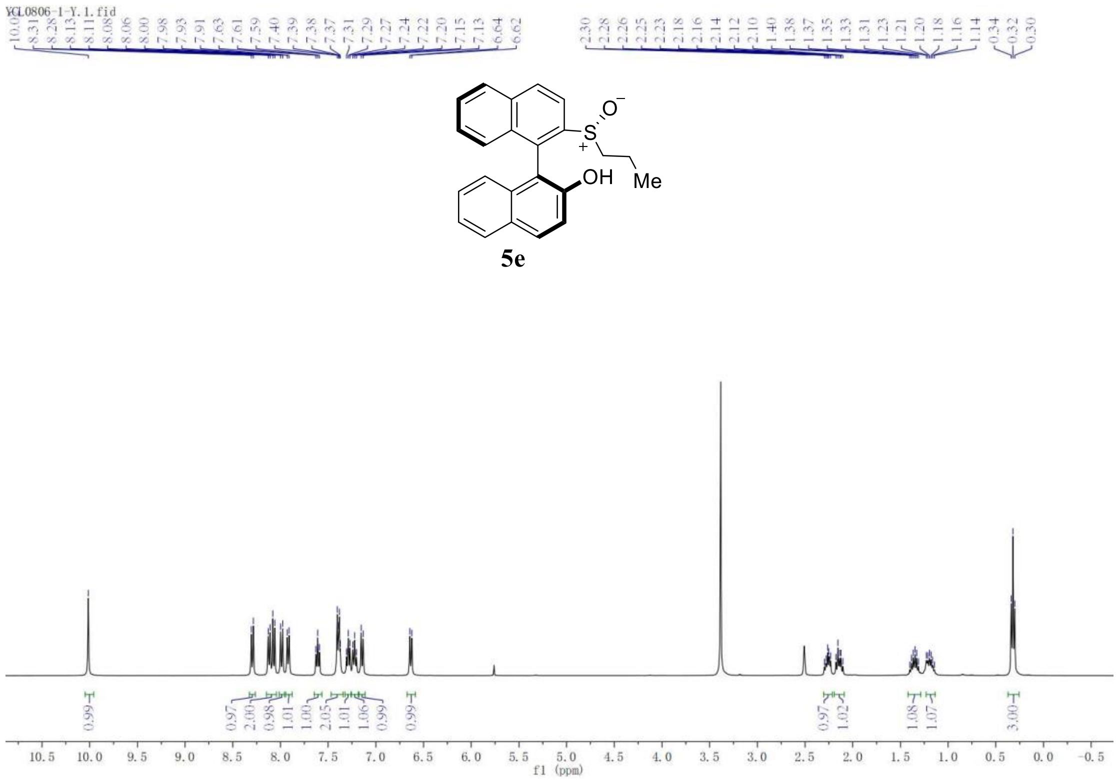
YCL0806-1-Y.1.fid
10.02 8.31 8.28 8.13 8.11 8.08 8.06 8.00 7.98 7.93 7.91 7.63 7.61 7.59 7.40 7.39 7.38 7.37 7.31 7.29 7.27 7.24 7.22 7.20 7.15 7.13 6.64 6.62
2.30 2.28 2.26 2.25 2.23 2.18 2.16 2.14 2.12 2.10 1.40 1.38 1.37 1.35 1.33 1.31 1.23 1.21 1.20 1.18 1.16 1.14 0.34 0.32 0.30
OH
Me
5e
0.99 0.97 2.00 0.98 1.01 1.00 2.05 1.01 1.06 0.99 0.99 0.97 1.02 1.08 1.07 3.00
f1 (ppm)

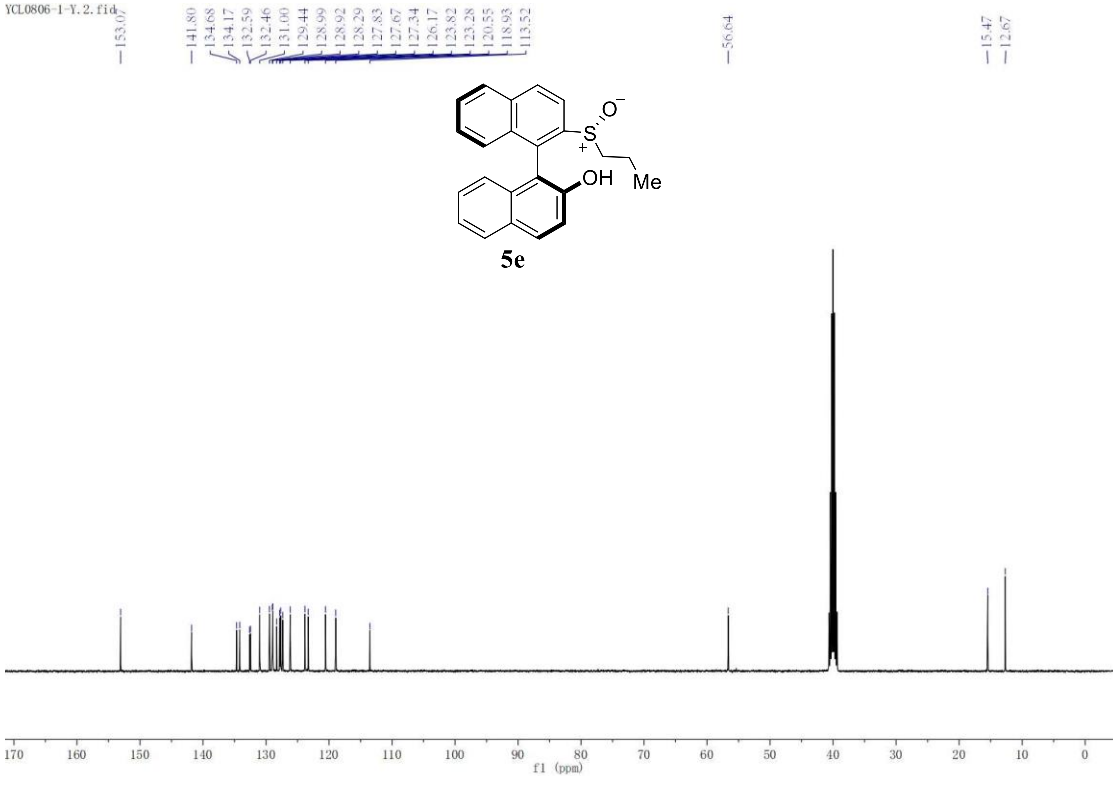
YCL0806-1-Y.2.fid
153.07
141.80 134.68 134.17 132.59 132.46 131.00 129.44 128.99 128.92 128.29 127.83 127.67 127.34 126.17 123.82 123.28 120.55 118.93 113.52
56.64
15.47 12.67
OH
Me
5e
f1 (ppm)

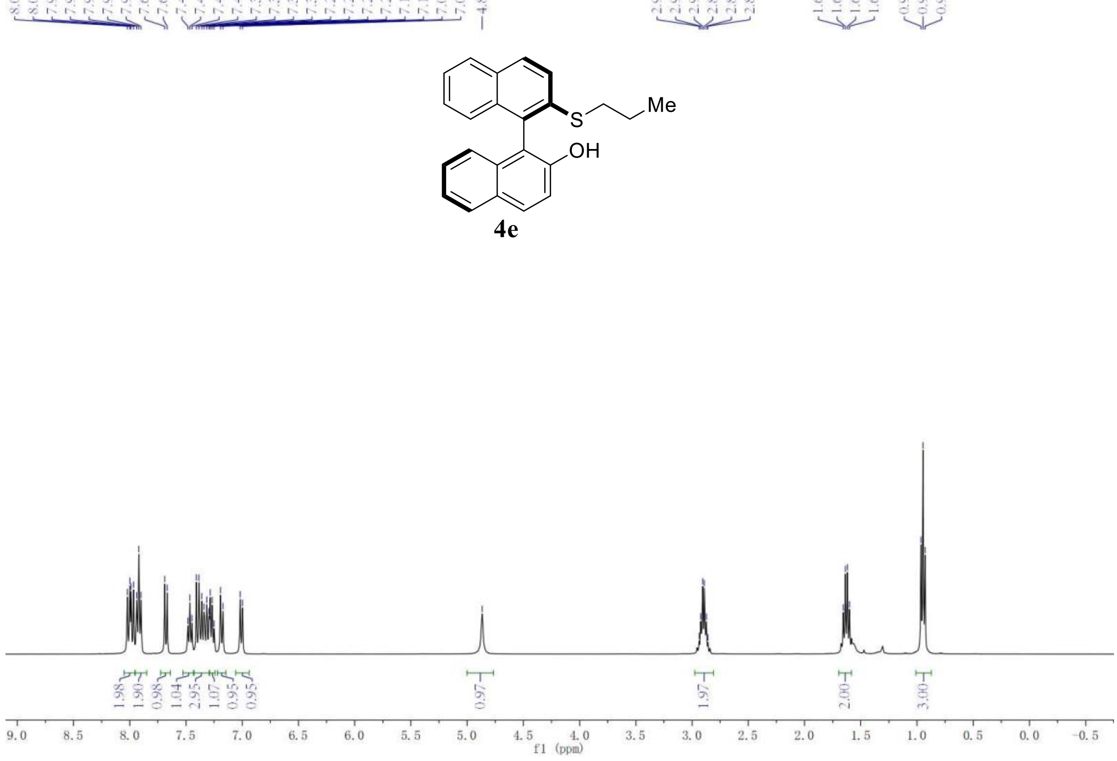

YCL 0806-1-R.1.fid
8.01 8.00 7.99 7.97 7.95 7.93 7.90 7.69 7.67 7.48 7.47 7.45 7.41 7.39 7.36 7.34 7.31 7.29 7.28 7.27 7.25 7.19 7.17 7.02 7.00
4.87
2.94 2.92 2.91 2.89 2.87 2.86
1.66 1.64 1.62 1.60
0.96 0.95 0.93
S
Me
OH
1.98 1.90 0.98 1.04 2.95 1.07 0.93 0.95
0.97
1.97
2.00
3.00
9.0 8.5 8.0 7.5 7.0 6.5 6.0 5.5 5.0 4.5 4.0 3.5 3.0 2.5 2.0 1.5 1.0 0.5 0.0 -0.5
f1 (ppm)

4e

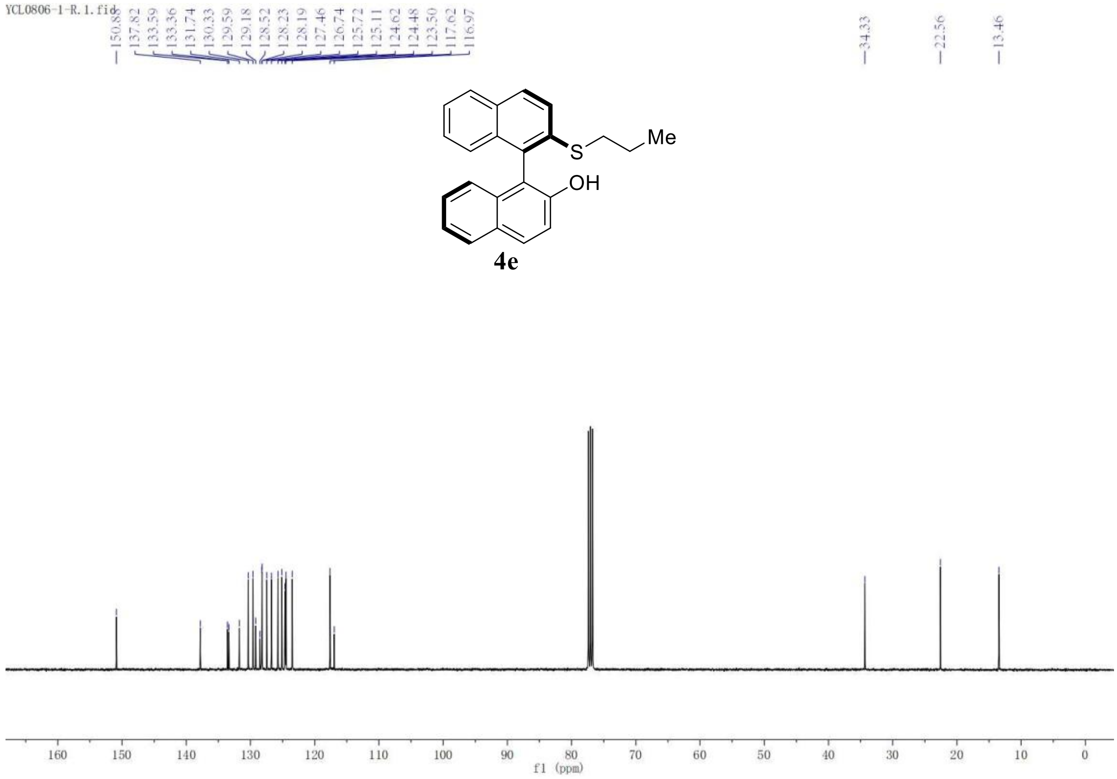

YCL0806-1-R.1.fid
150.88 137.82 133.59 133.36 131.74 130.33 129.59 129.18 128.52 128.23 128.19 127.46 126.74 125.72 125.11 124.62 124.48 123.50 117.62 116.97
34.33
22.56
13.46
S
Me
OH
160 150 140 130 120 110 100 90 80 70 60 50 40 30 20 10 0
f1 (ppm)

4e

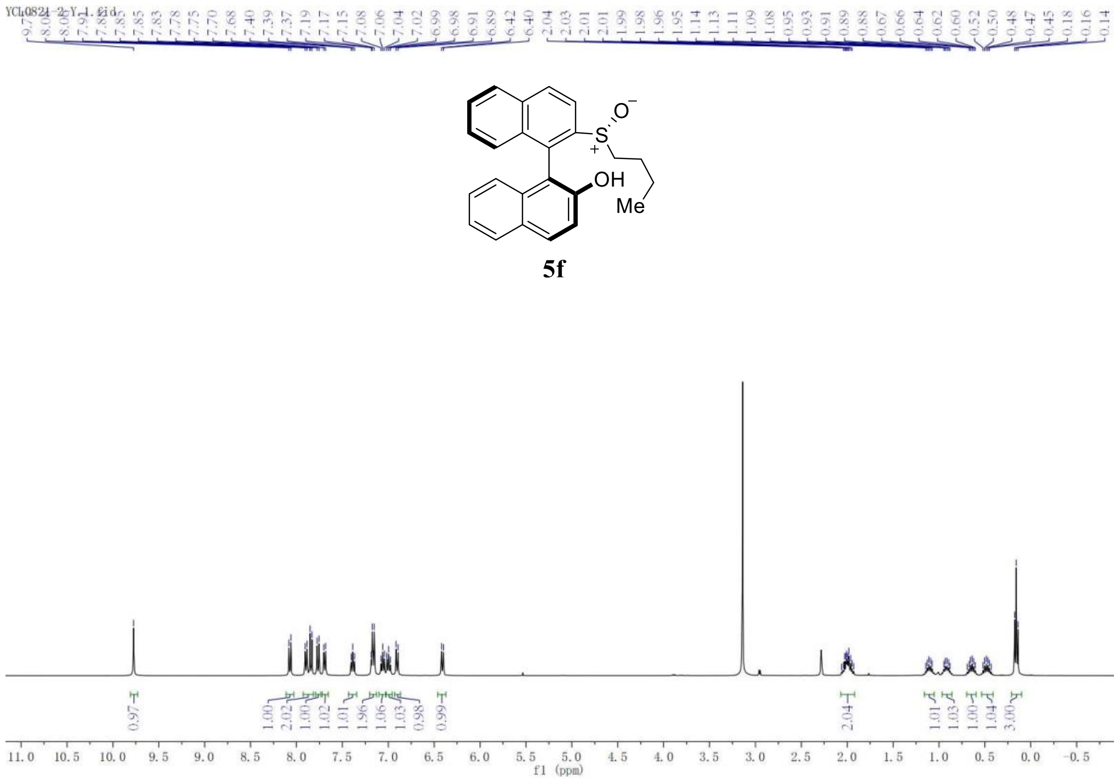
YCL0821-2-Y.1.fid
7.85 7.83 7.78 7.75 7.70 7.68 7.40 7.39 7.37 7.19 7.17 7.15 7.08 7.06 7.04 7.02 6.99 6.98 6.91 6.89 6.42 6.40 2.04 2.03 2.01 1.99 1.98 1.96 1.95 1.14 1.13 1.11 1.09 1.08 0.95 0.93 0.91 0.89 0.88 0.67 0.66 0.64 0.62 0.60 0.52 0.50 0.48 0.47 0.45 0.18 0.16 0.14
O
S
+
OH
Me
5f
0.97 1.00 2.02 1.00 1.02 1.01 1.96 1.06 1.03 0.98 0.99 2.04 1.01 1.03 1.00 1.04 3.00
11.0 10.5 10.0 9.5 9.0 8.5 8.0 7.5 7.0 6.5 6.0 5.5 5.0 4.5 4.0 3.5 3.0 2.5 2.0 1.5 1.0 0.5 0.0 -0.5
f1 (ppm)

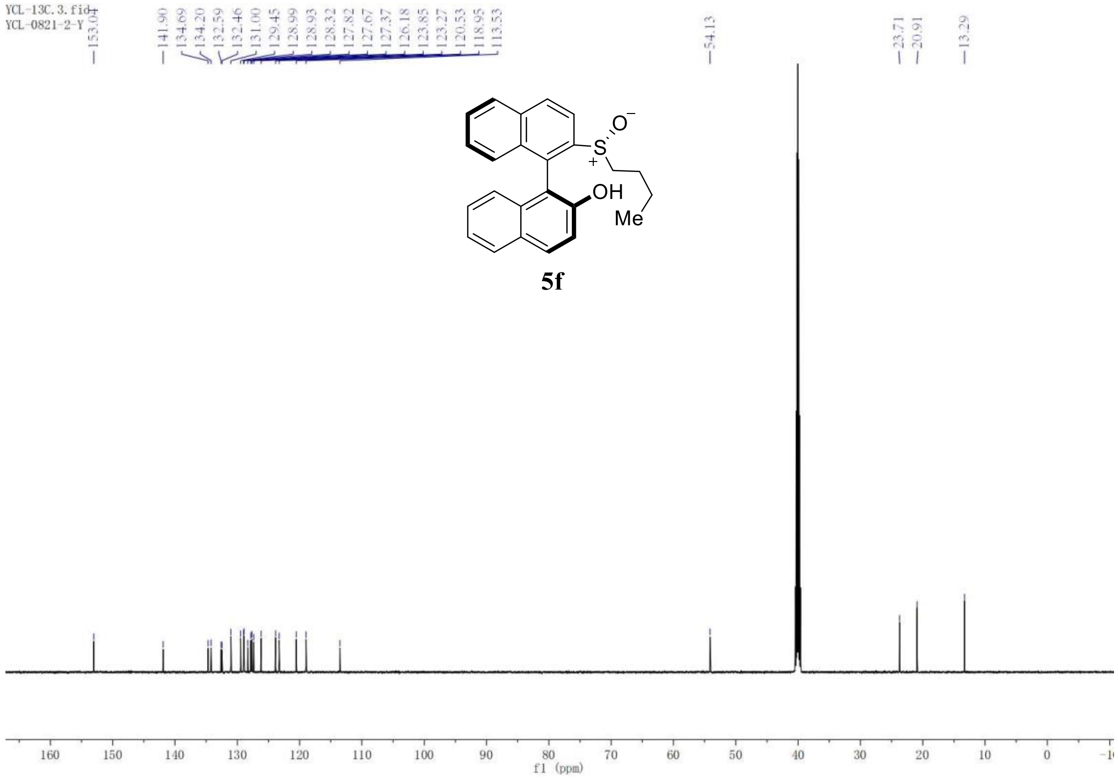
YCL-13C.3.fid
YCL-0821-2-Y
153.04 141.90 134.69 134.20 132.59 132.46 131.00 129.45 128.99 128.93 128.32 127.82 127.67 127.37 126.18 123.85 123.27 120.53 118.95 113.53 54.13 23.71 20.91 13.29
O
S
+
OH
Me
5f
160 150 140 130 120 110 100 90 80 70 60 50 40 30 20 10 0
f1 (ppm)

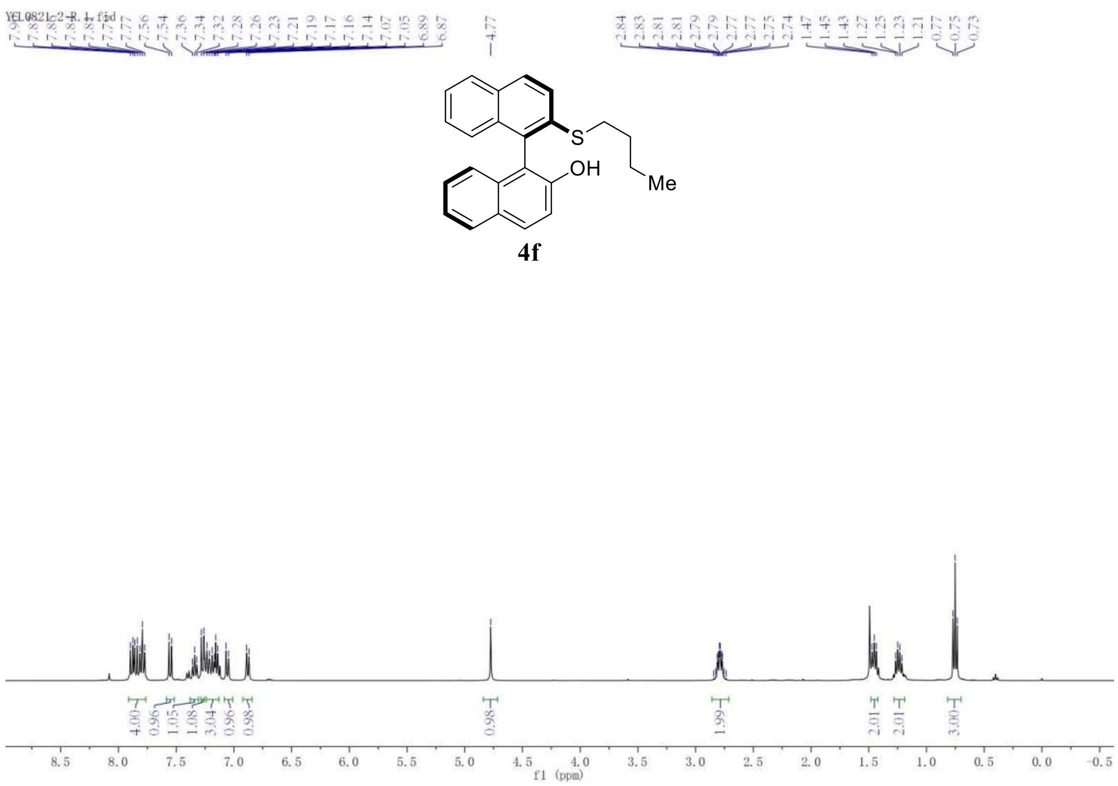
YCL0821-2-R.1.fid
S
OH
Me
4f
f1 (ppm)

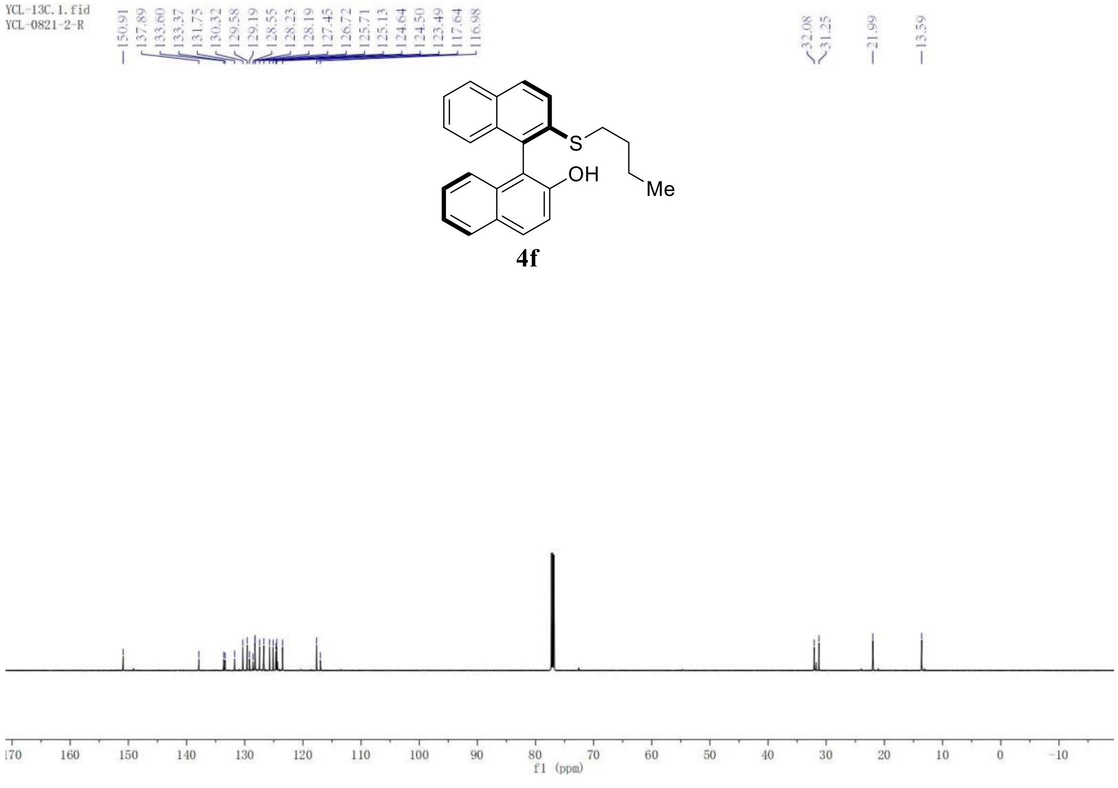
YCL-13C.1.fid
YCL-0821-2-R
S
OH
Me
4f
f1 (ppm)

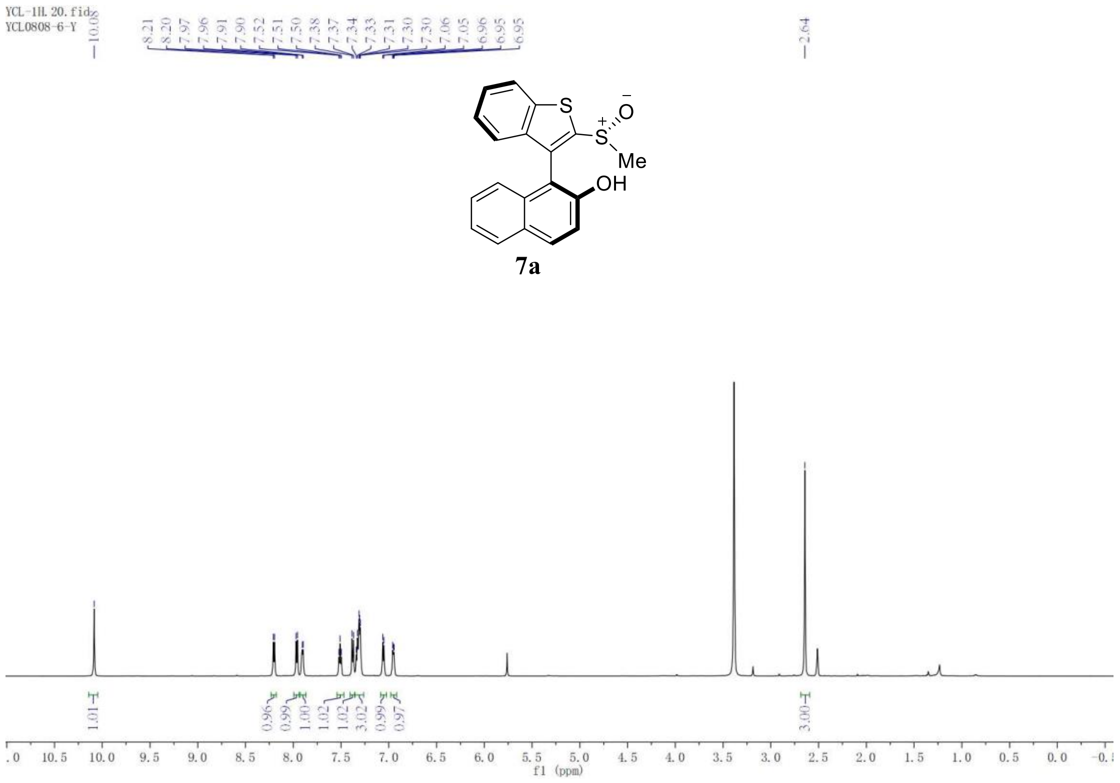
YCL-1H.20.fid
YCL0808-6-Y
10.08
8.21 8.20 7.97 7.96 7.91 7.90 7.52 7.51 7.50 7.38 7.37 7.34 7.33 7.31 7.30 7.06 7.05 6.96 6.95 6.95
2.64
S
O
Me
OH
7a
1.01
0.96 0.99 1.00 1.02 1.02 3.02 0.99 0.97
3.00
f1 (ppm)

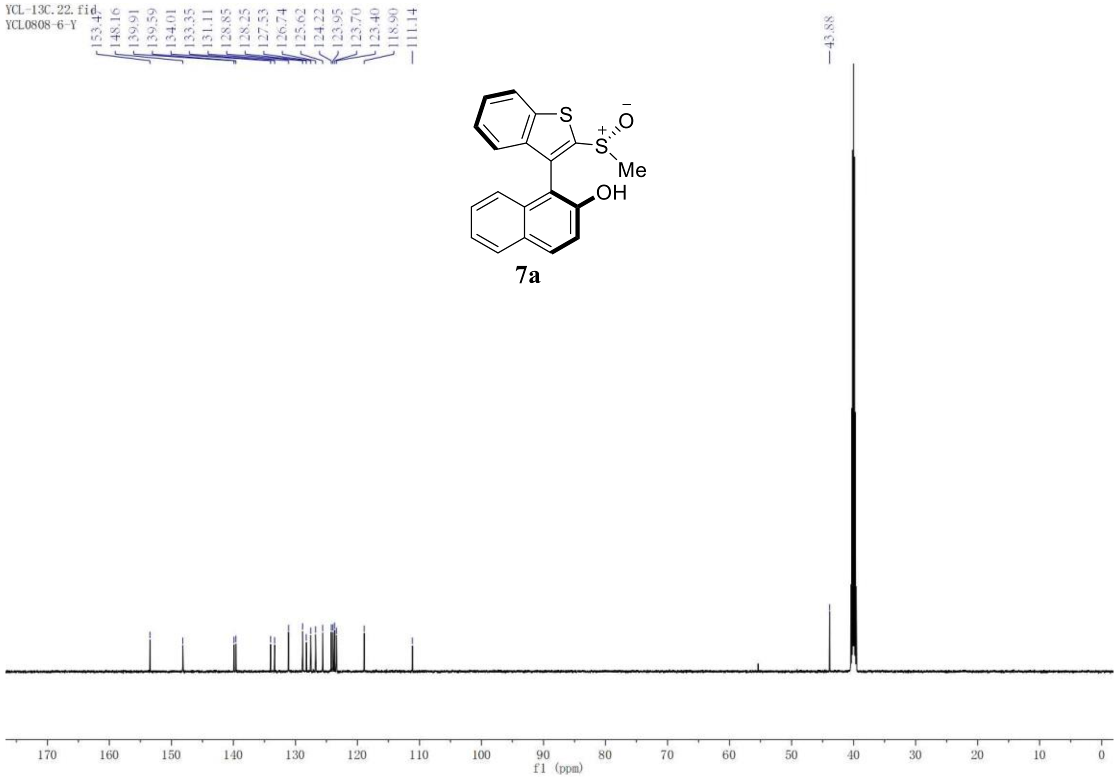
YCL-13C.22.fid
YCL0808-6-Y
153.44 148.16 139.91 139.59 134.01 133.35 131.11 128.85 128.25 127.53 126.74 125.62 124.22 123.95 123.70 123.40 118.90 111.14
43.88
S
O
Me
OH
7a
f1 (ppm)

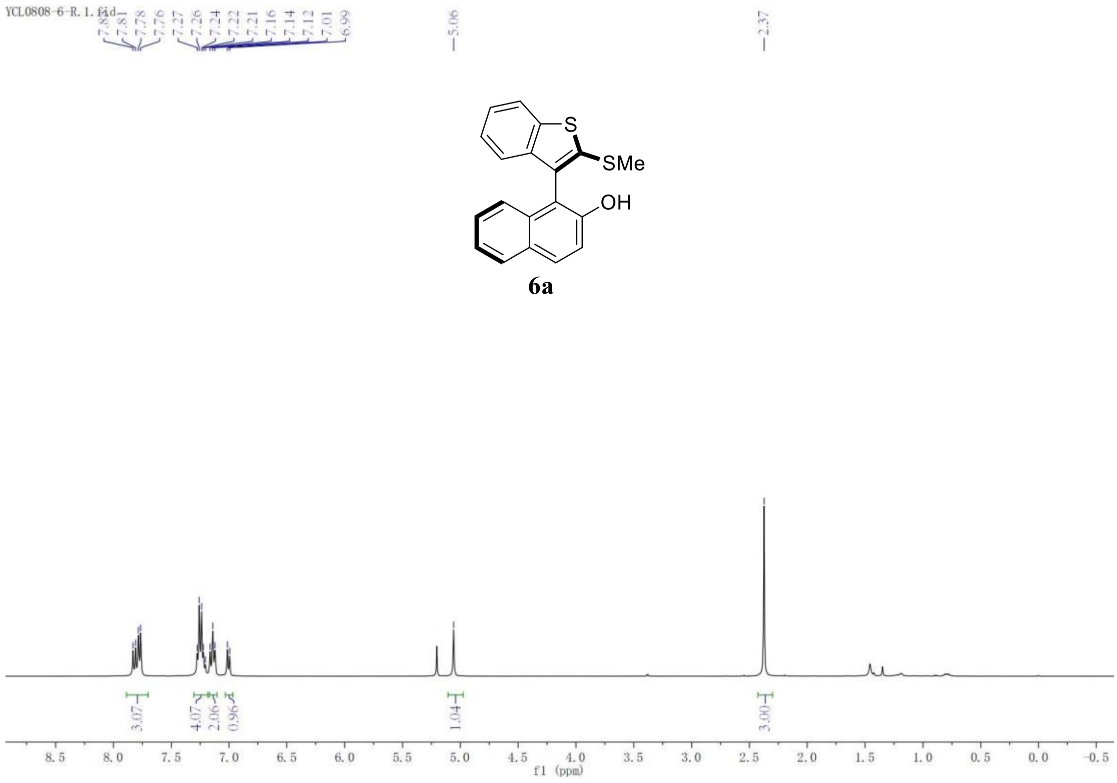
YCL0808-6-R.1.fid
7.81
7.78
7.76
7.27
7.26
7.24
7.22
7.21
7.16
7.14
7.12
7.01
6.99
5.06
2.37
SMe
OH
S
3.07
4.07
2.06
0.96
1.04
3.00
f1 (ppm)

6a

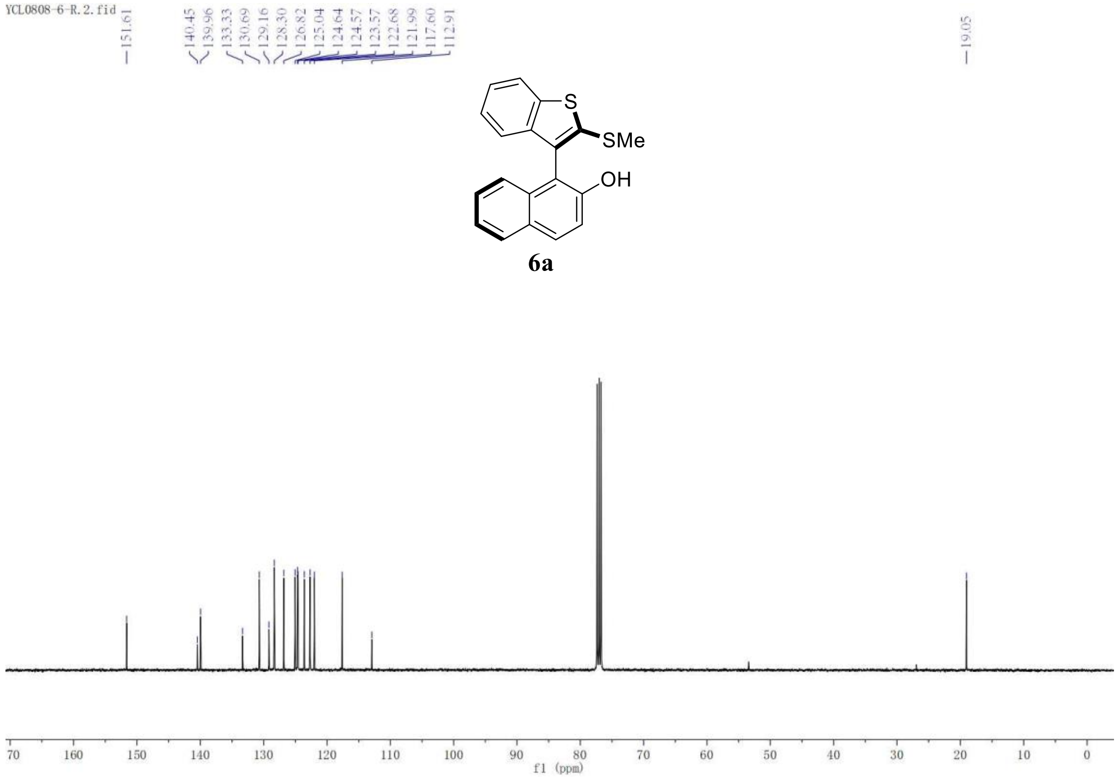
YCL0808-6-R.2.fid
151.61
140.45
139.96
133.33
130.69
129.16
128.30
126.82
125.04
124.64
124.57
123.57
122.68
121.99
117.60
112.91
19.05
SMe
OH
S
f1 (ppm)

6a

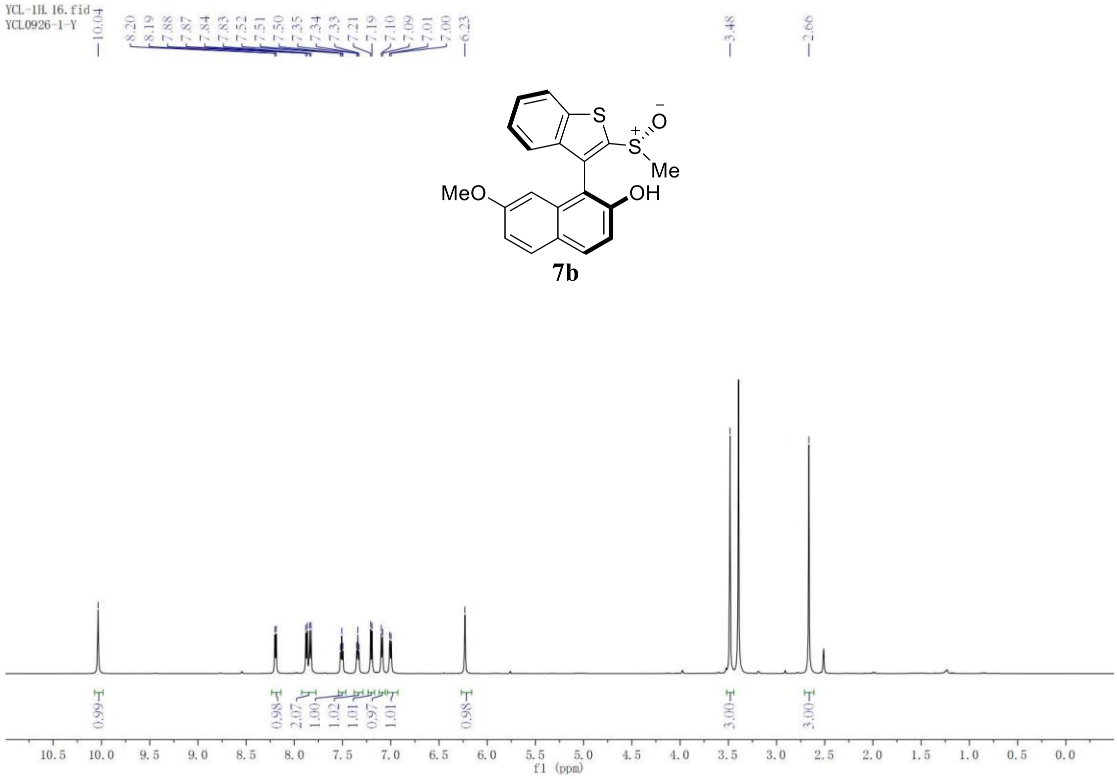
YCL-1H. 16. fid
YCL0926-1-Y
10.04
8.20
8.19
7.88
7.87
7.84
7.83
7.52
7.51
7.50
7.35
7.34
7.33
7.21
7.19
7.10
7.09
7.01
7.00
6.23
3.48
2.66
MeO
OH
S
O
Me
7b
0.99
0.98
2.07
1.00
1.02
1.01
0.97
1.01
0.98
3.00
3.00
f1 (ppm)

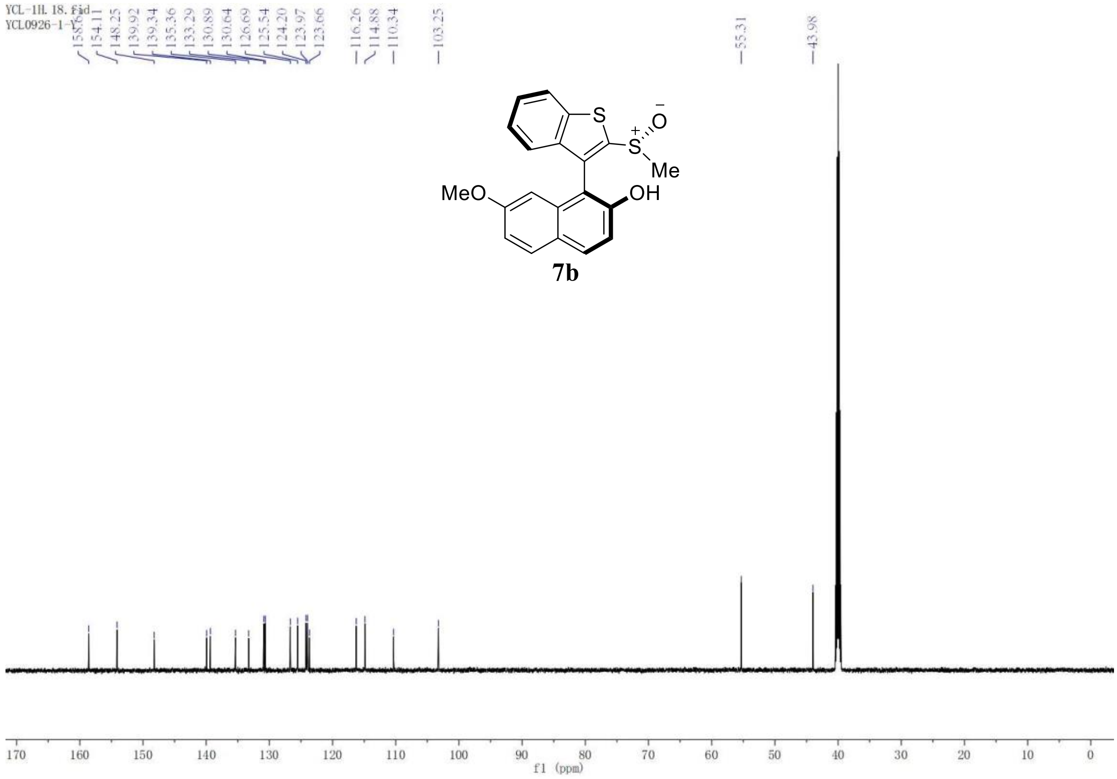
YCL-1H. 18. fid
YCL0926-1-Y
154.11
148.25
139.92
139.34
135.36
133.29
130.89
130.64
126.69
125.54
124.20
123.97
123.66
116.26
114.88
110.34
103.25
55.31
43.98
MeO
OH
S
O
Me
7b
f1 (ppm)

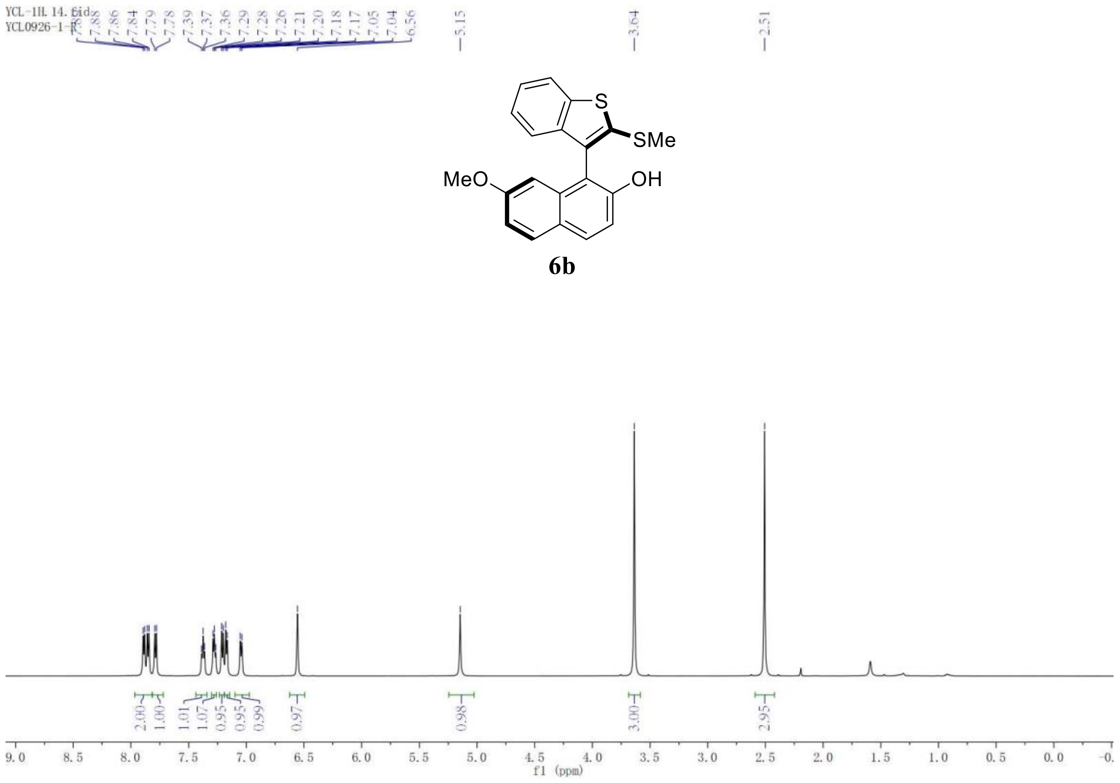
YCL-1H.14.fid
YCL0926-1-R
SMe
MeO
OH
6b
f1 (ppm)

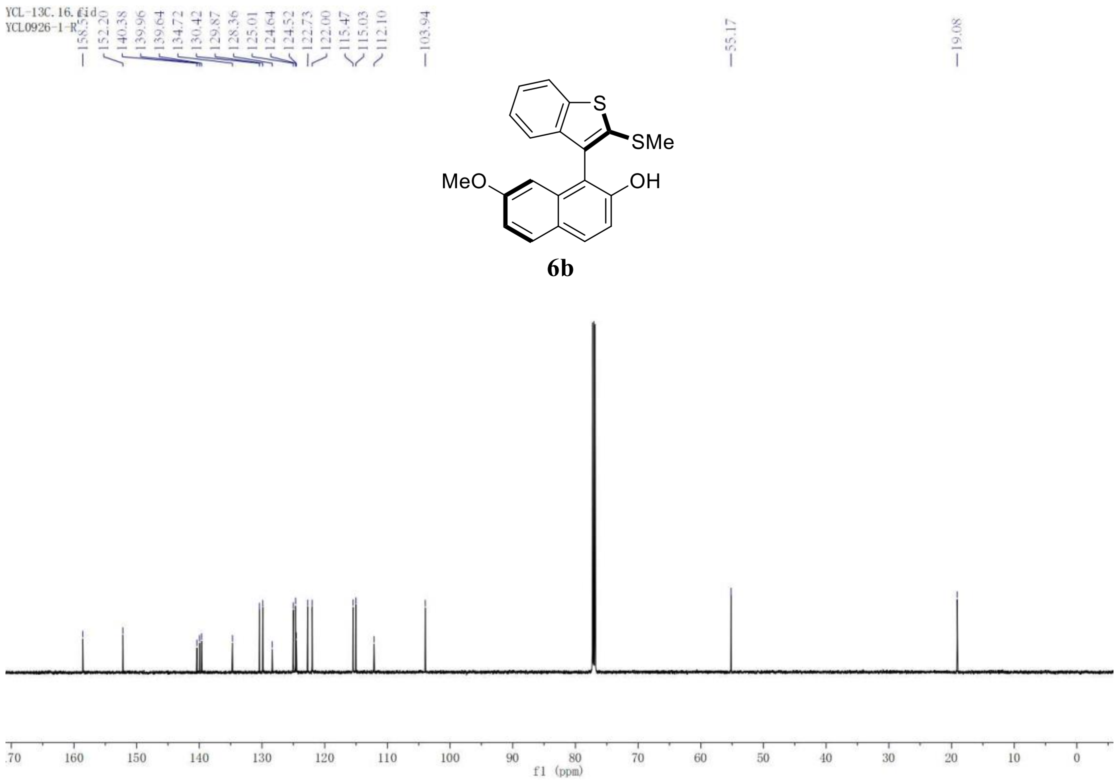
YCL-13C.16.fid
YCL0926-1-R
SMe
MeO
OH
6b
f1 (ppm)

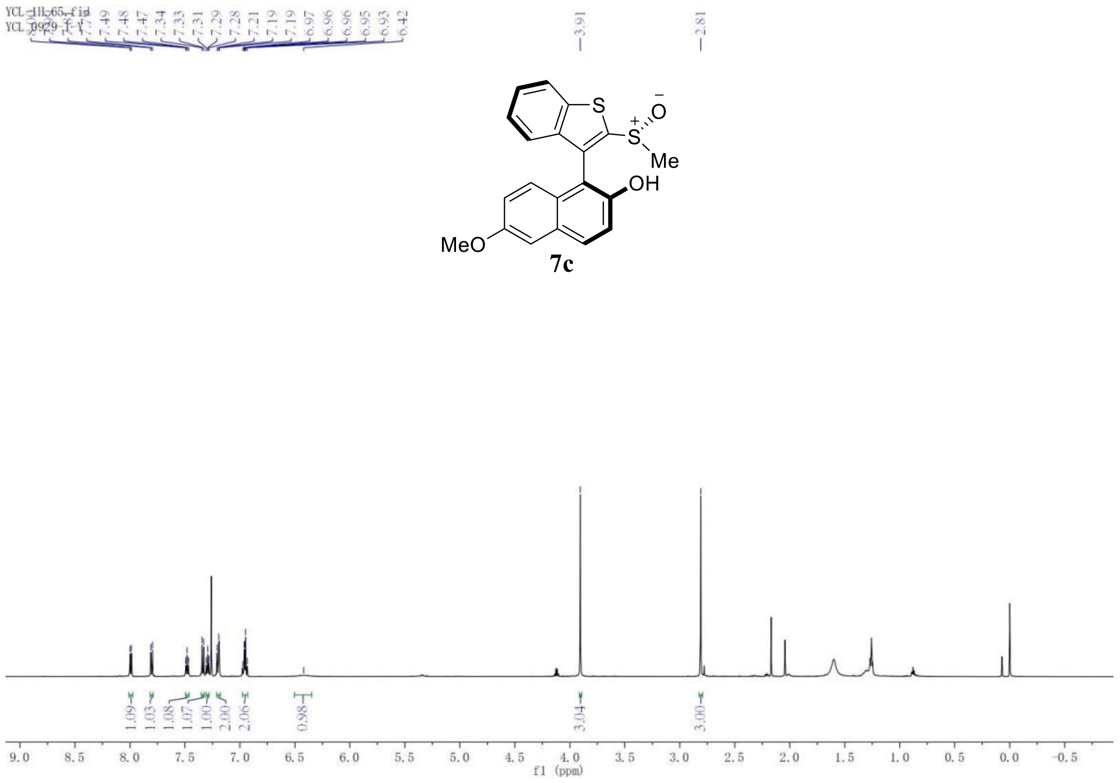

YCL-1H-65.fid
YCL 0929-1-1
7.49
7.48
7.47
7.34
7.33
7.31
7.29
7.28
7.21
7.19
7.19
6.97
6.96
6.96
6.95
6.93
6.42
3.91
2.81
S
O
S
Me
OH
MeO
7c
1.09
1.03
1.08
1.07
1.00
2.00
2.06
0.98
3.04
3.00
9.0
8.5
8.0
7.5
7.0
6.5
6.0
5.5
5.0
4.5
4.0
3.5
3.0
2.5
2.0
1.5
1.0
0.5
0.0
-0.5
f1 (ppm)

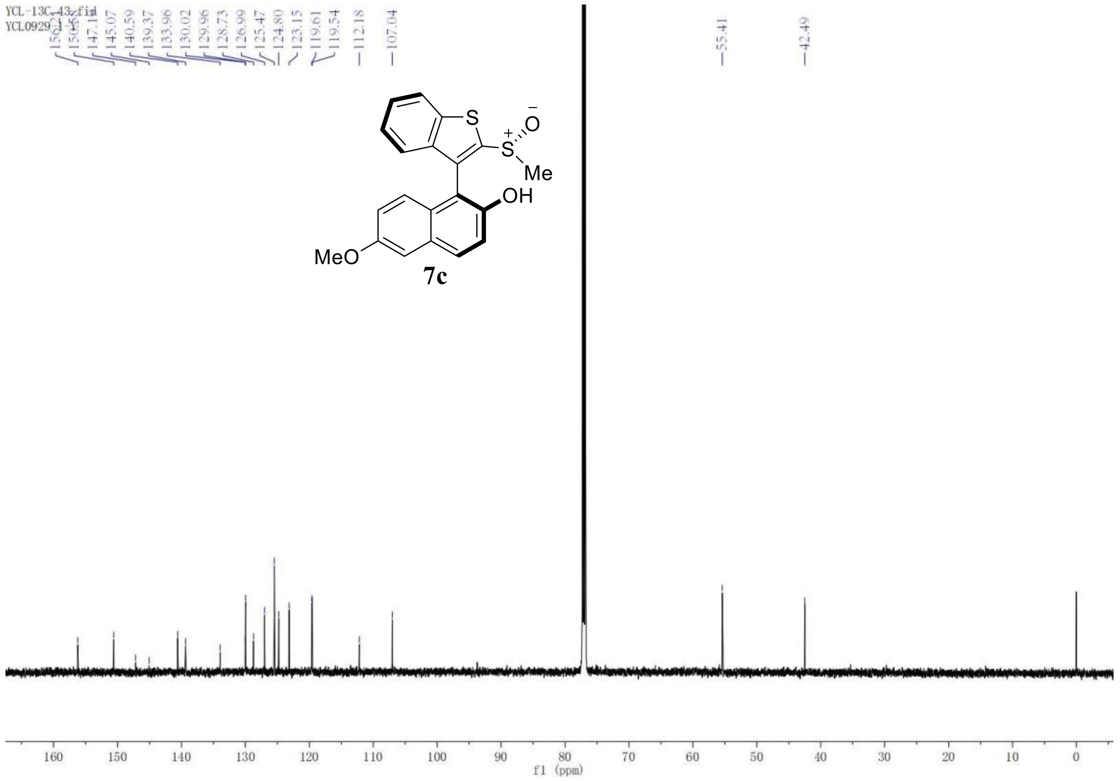

YCL-13C-43.fid
YCL0929-1-1
147.11
145.07
140.59
139.37
133.96
130.02
129.96
128.73
126.99
125.47
124.80
123.15
119.61
119.54
112.18
107.04
55.41
42.49
S
O
S
Me
OH
MeO
7c
160
150
140
130
120
110
100
90
80
70
60
50
40
30
20
10
0
f1 (ppm)

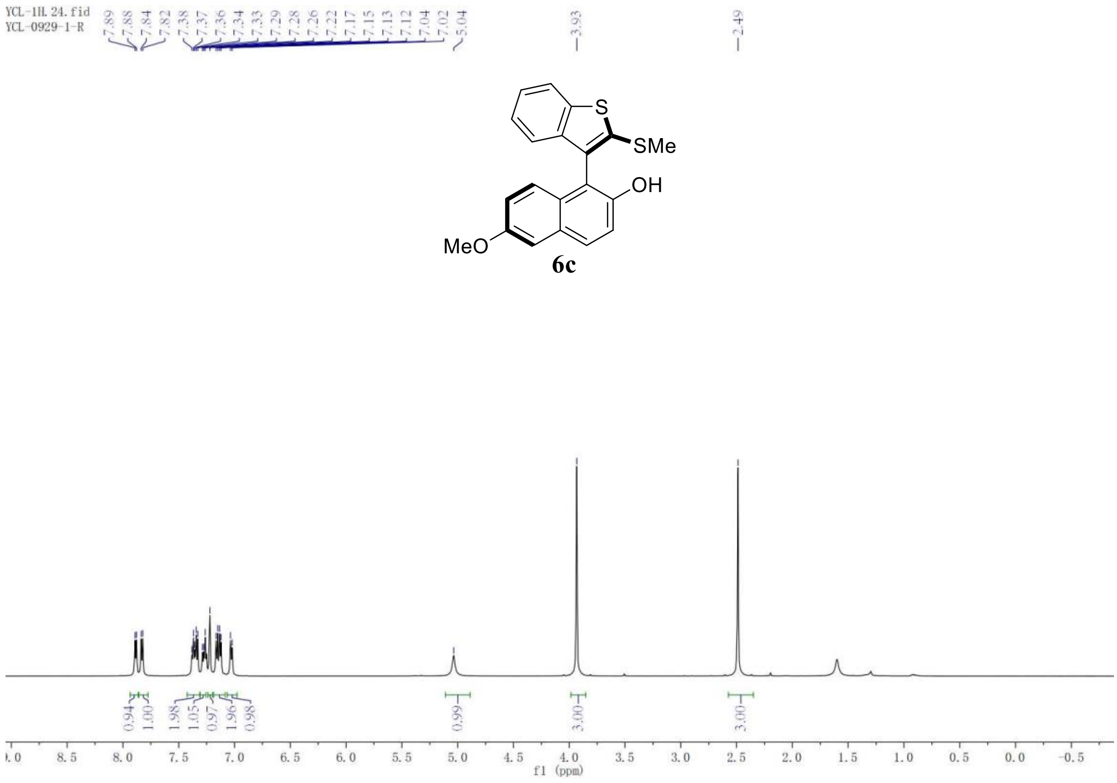
YCL-1H.24.fid
YCL-0929-1-R
7.89 7.88 7.84 7.82 7.38 7.37 7.36 7.34 7.33 7.29 7.28 7.26 7.22 7.17 7.15 7.13 7.12 7.04 7.02 5.04
3.93
2.49
S
SMe
OH
MeO
6c
0.94 1.00 1.98 1.05 0.97 1.96 0.98
0.99
3.00
3.00
f1 (ppm)

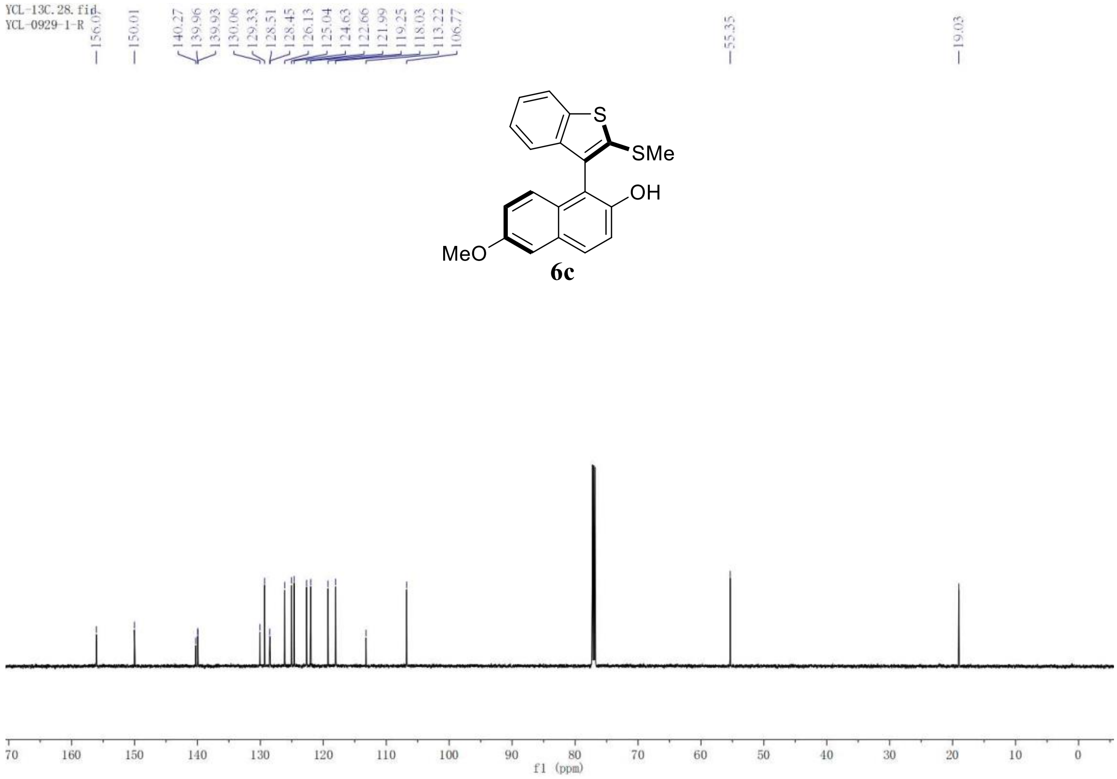
YCL-13C.28.fid
YCL-0929-1-R
156.07 150.01 140.27 139.96 139.93 130.06 129.33 128.51 128.45 126.13 125.04 124.63 122.66 121.99 119.25 118.03 113.22 106.77
55.35
19.03
S
SMe
OH
MeO
6c
f1 (ppm)

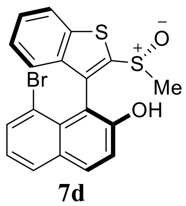

7d

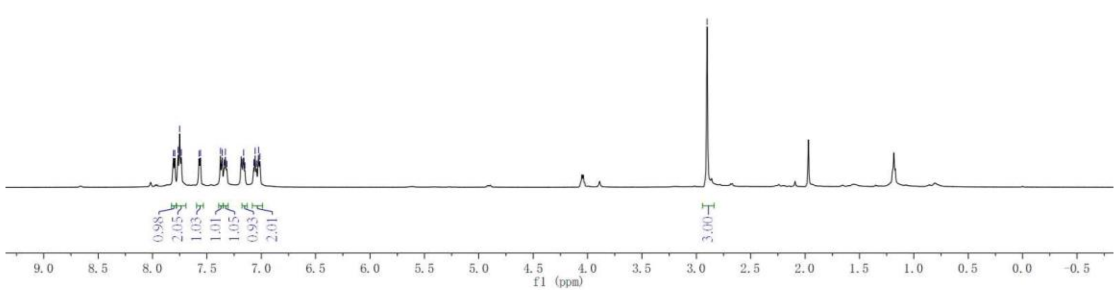

YCL-1H 29.fid
YCL-0927-3-Y
7.75 7.73 7.57 7.56 7.37 7.36 7.34 7.33 7.32 7.18 7.16 7.15 7.07 7.06 7.04 7.03 7.01
2.90
0.98 2.05 1.03 1.01 1.05 0.93 2.01
3.00
9.0 8.5 8.0 7.5 7.0 6.5 6.0 5.5 5.0 4.5 4.0 3.5 3.0 2.5 2.0 1.5 1.0 0.5 0.0 -0.5
f1 (ppm)

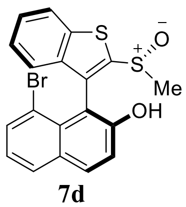

7d

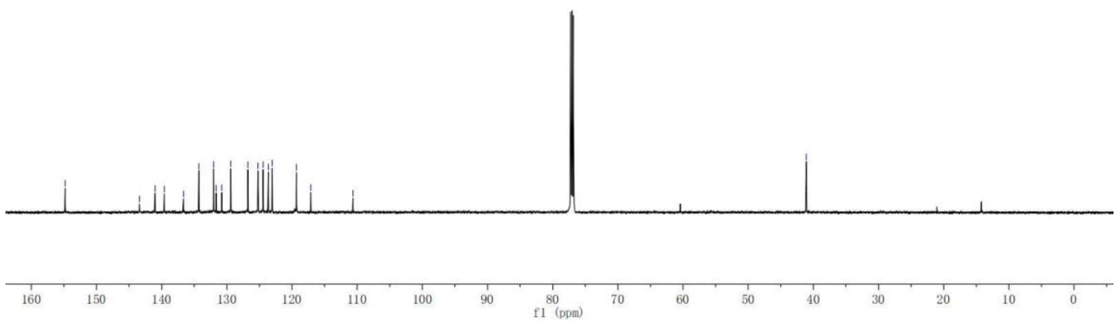

YCL-13C 23.fid
YCL-0927-3-Y
139.65 136.65 134.30 132.02 131.63 130.79 129.38 126.77 125.21 124.44 123.64 123.03 119.31 117.11 110.65
41.10
160 150 140 130 120 110 100 90 80 70 60 50 40 30 20 10 0
f1 (ppm)

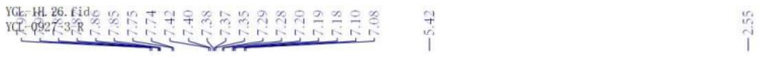
YCL-1H.26.fid
YCL-0927-3-R
7.86
7.85
7.75
7.74
7.42
7.40
7.38
7.37
7.35
7.29
7.28
7.20
7.19
7.18
7.10
7.08
5.42
2.55

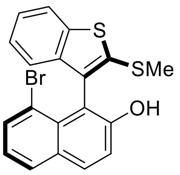
S
SMe
Br
OH

6d

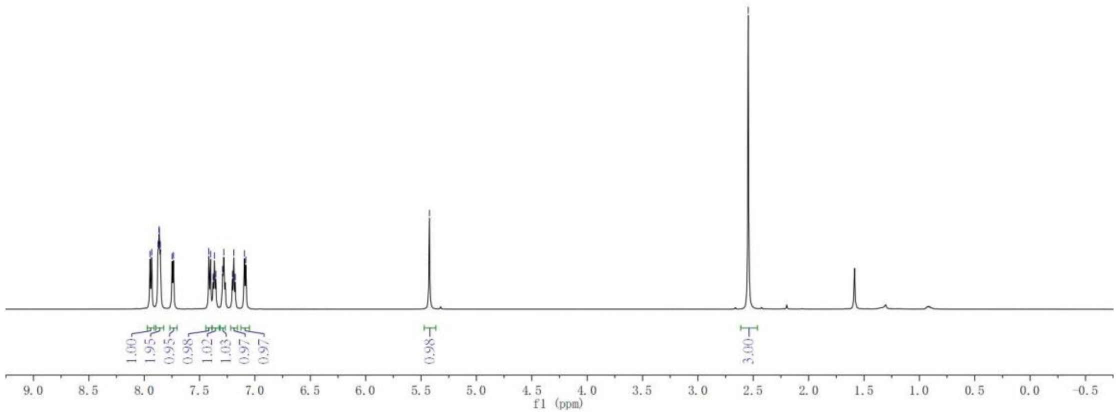
1.00
1.95
0.95
0.98
1.02
1.03
0.97
0.97
0.98
3.00
9.0 8.5 8.0 7.5 7.0 6.5 6.0 5.5 5.0 4.5 4.0 3.5 3.0 2.5 2.0 1.5 1.0 0.5 0.0 -0.5
f1 (ppm)

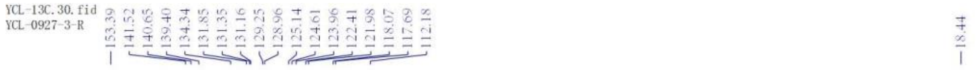
YCL-13C.30.fid
YCL-0927-3-R
153.39
141.52
140.65
139.40
134.34
131.85
131.35
131.16
129.25
128.96
125.14
124.61
123.96
122.41
121.98
118.07
117.69
112.18
18.44

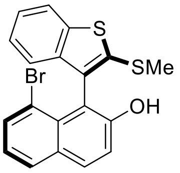
S
SMe
Br
OH

6d

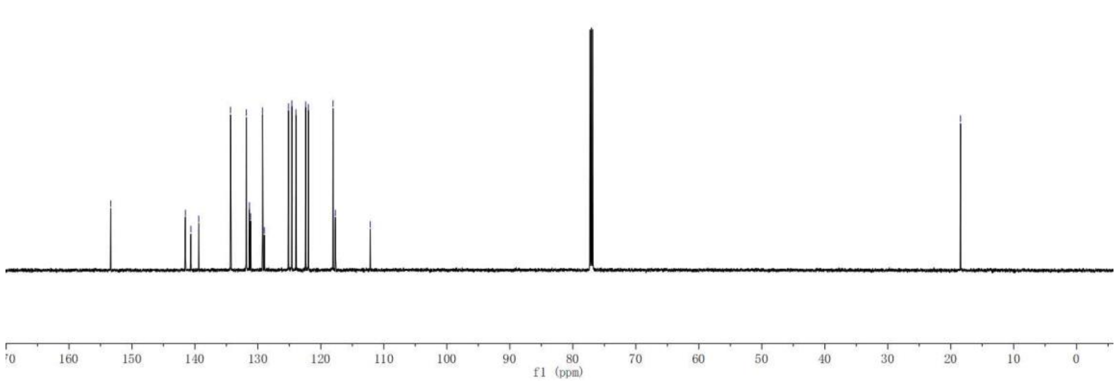
160 150 140 130 120 110 100 90 80 70 60 50 40 30 20 10 0
f1 (ppm)

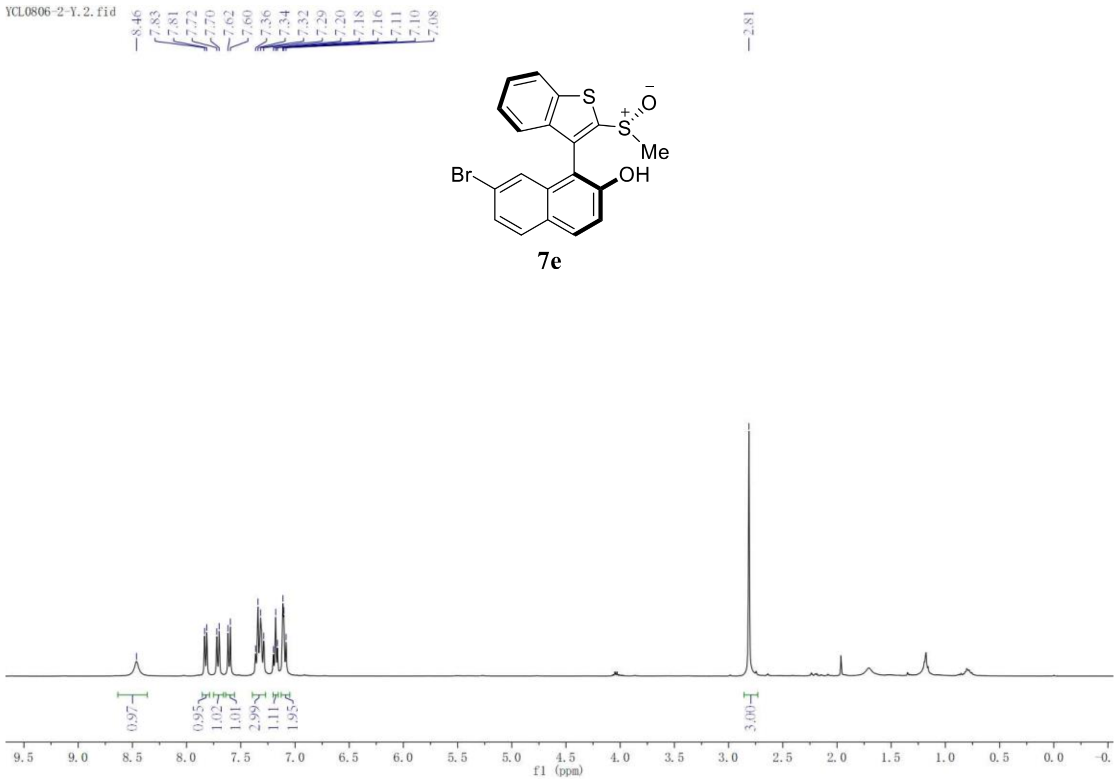

YCL0806-2-Y.2.fid
8.46
7.83
7.81
7.72
7.70
7.62
7.60
7.36
7.34
7.32
7.29
7.20
7.18
7.16
7.11
7.10
7.08
2.81
S
O
Me
Br
OH
0.97
0.95
1.02
1.01
2.99
1.11
1.95
3.00
9.5
9.0
8.5
8.0
7.5
7.0
6.5
6.0
5.5
5.0
4.5
4.0
3.5
3.0
2.5
2.0
1.5
1.0
0.5
0.0
f1 (ppm)

7e

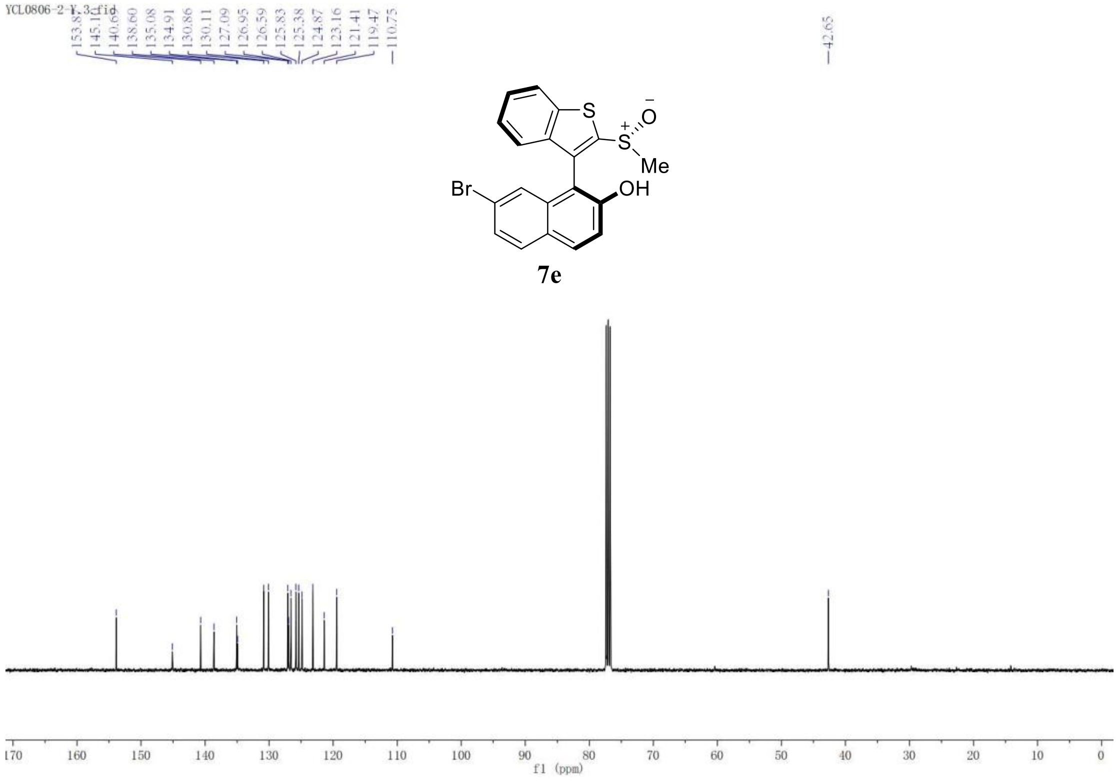

138.60
135.08
134.91
130.86
130.11
127.09
126.95
126.59
125.83
125.38
124.87
123.16
121.41
119.47
110.75
42.65
S
O
Me
Br
OH
170
160
150
140
130
120
110
100
90
80
70
60
50
40
30
20
10
0
f1 (ppm)

7e

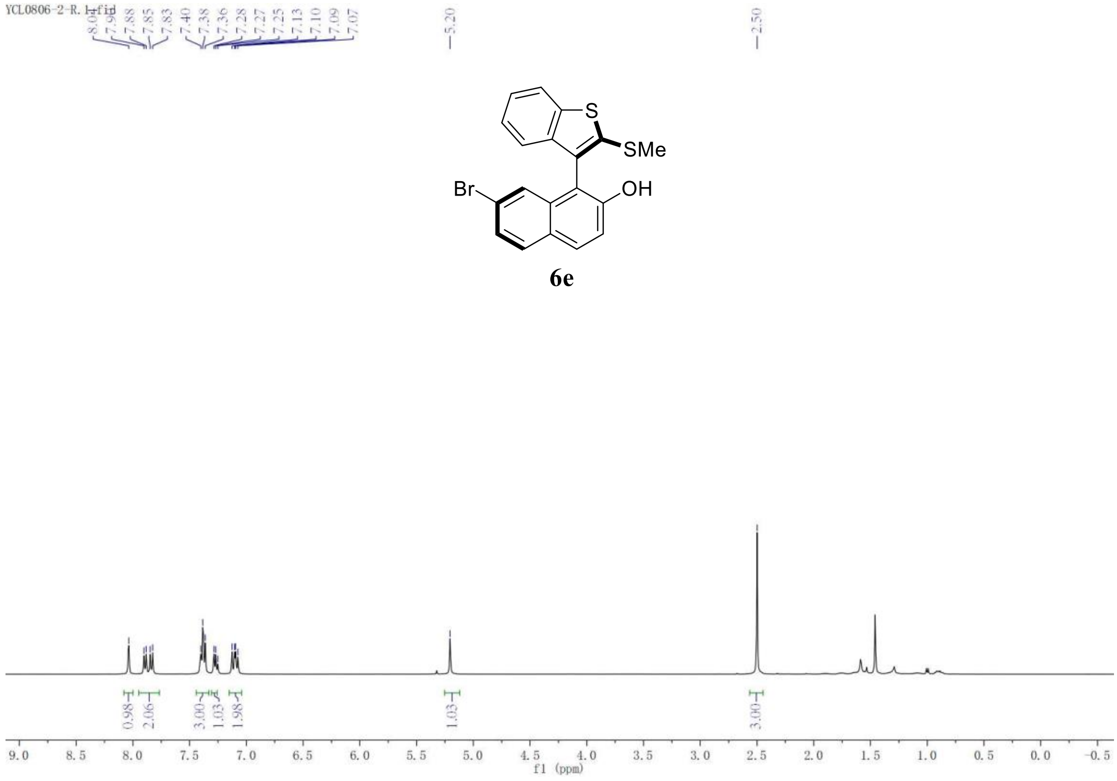
YCL0806-2-R.1.fid
8.04
7.90
7.88
7.85
7.83
7.40
7.38
7.36
7.28
7.27
7.25
7.13
7.10
7.09
7.07
5.20
2.50
S
SMe
Br
OH
6e
0.98
2.06
3.00
1.03
1.98
1.03
3.00
f1 (ppm)

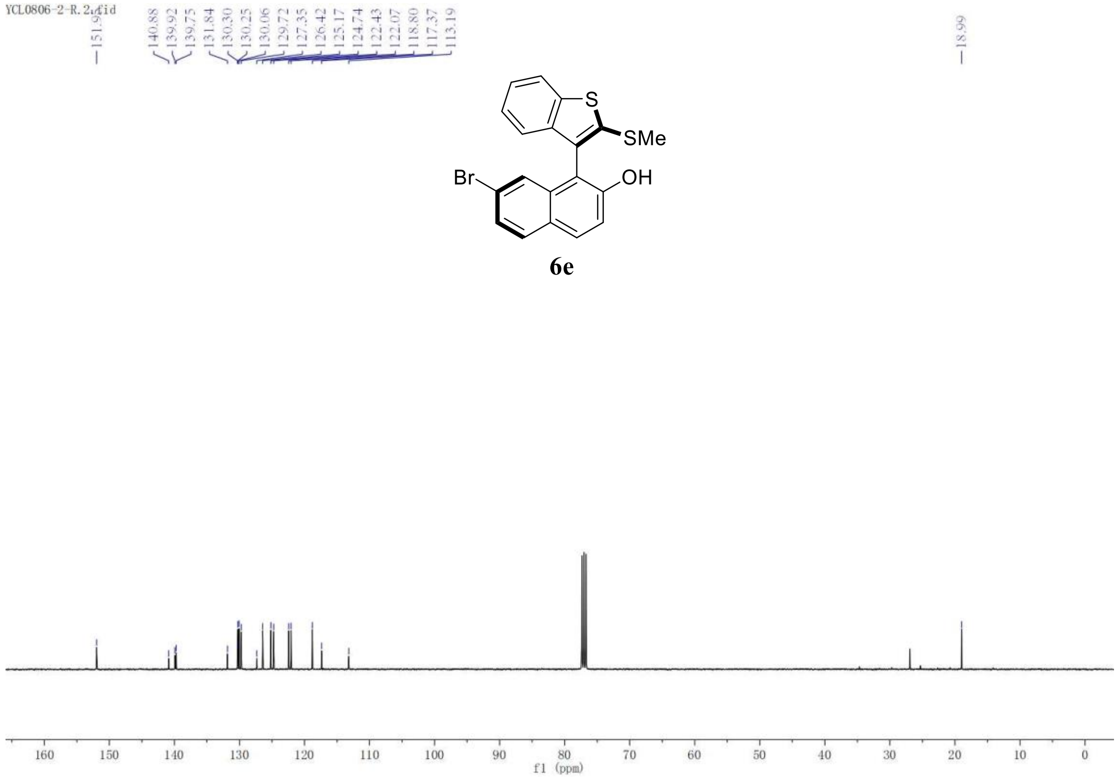
YCL0806-2-R.2.fid
151.93
140.88
139.92
139.75
131.84
130.30
130.25
130.06
129.72
127.35
126.42
125.17
124.74
122.43
122.07
118.80
117.37
113.19
18.99
S
SMe
Br
OH
6e
f1 (ppm)

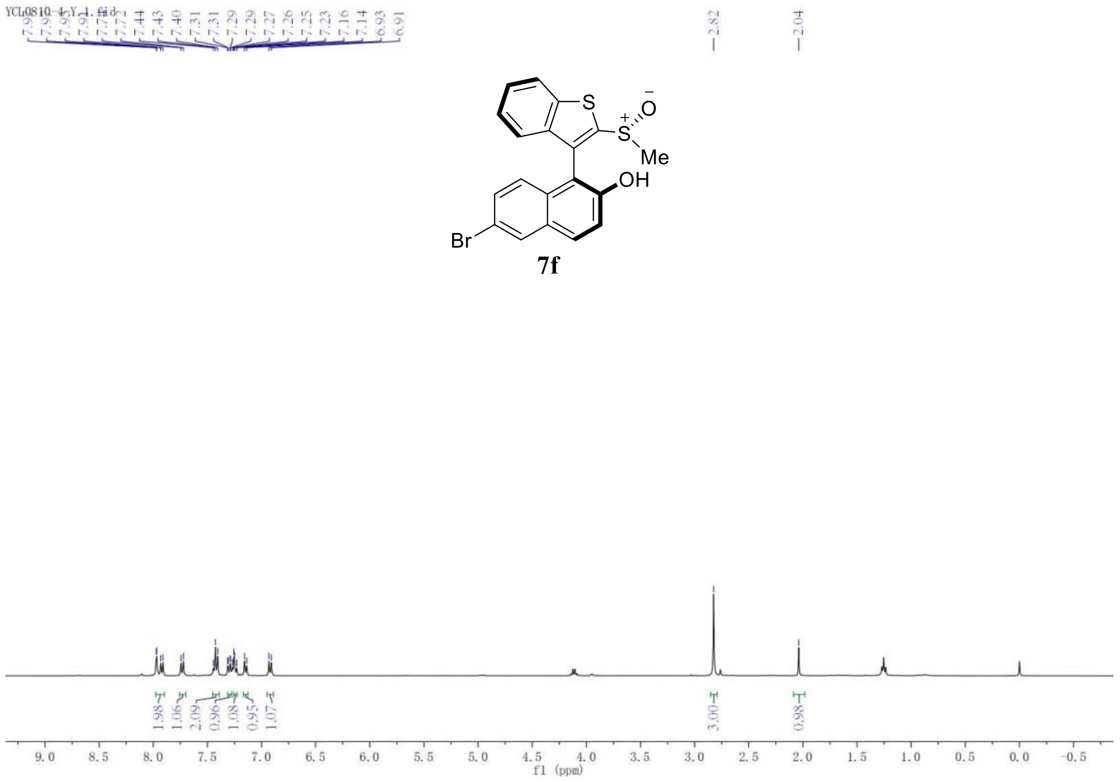
YCL0810-4-Y.1.fid
2.82
2.04
S
O
Me
OH
Br
7f
1.98
1.06
2.09
0.96
1.08
0.95
1.07
3.00
0.98
f1 (ppm)

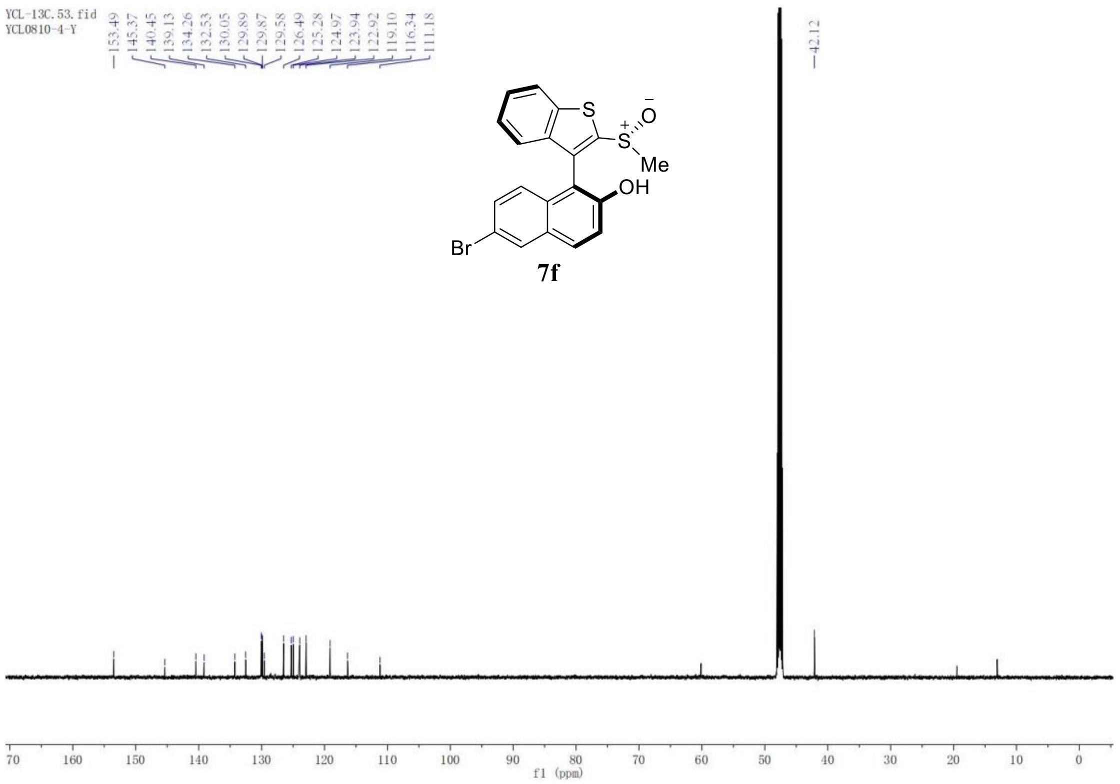
YCL-13C.53.fid
YCL0810-4-Y
153.49
145.37
140.45
139.13
134.26
132.53
130.05
129.89
129.87
129.58
126.49
125.28
124.97
123.94
122.92
119.10
116.34
111.18
42.12
S
O
Me
OH
Br
7f
f1 (ppm)

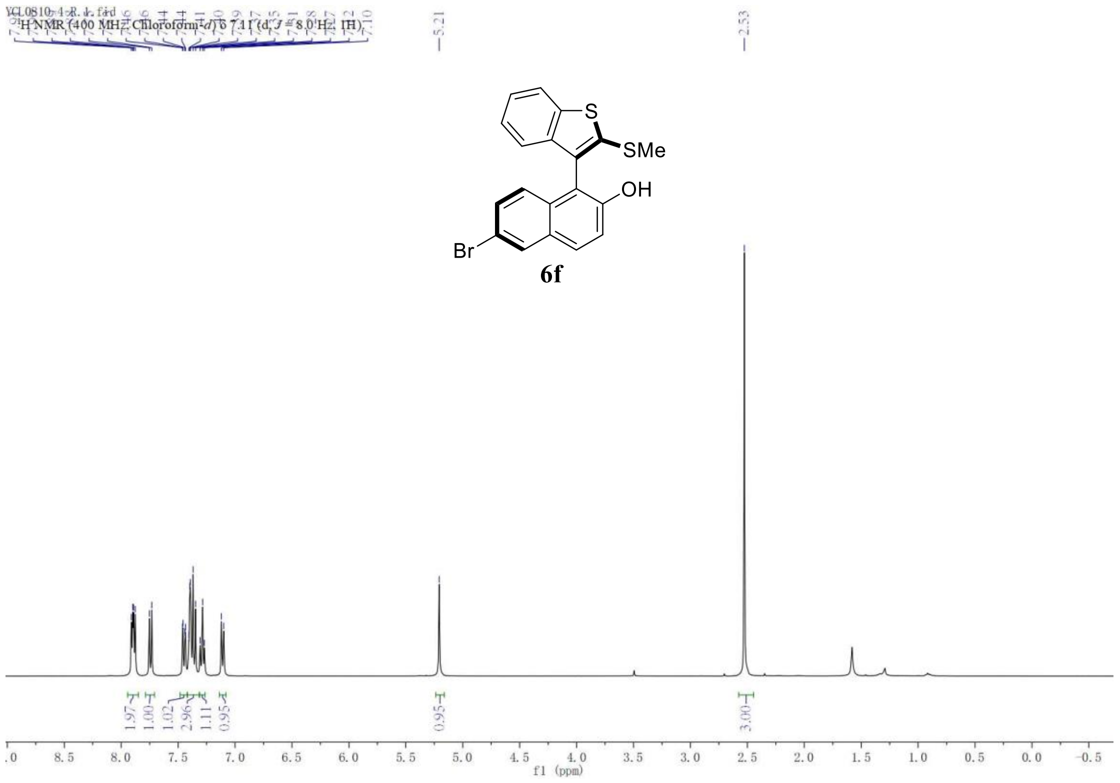
YCL0810-4-R.1.fid
1H NMR (400 MHz, Chloroform-d) δ 7.11 (d, J = 8.0 Hz, 1H).
5.21
2.53
S
SMe
OH
Br
6f
1.97
1.00
1.02
2.96
1.11
0.95
0.95
3.00
f1 (ppm)

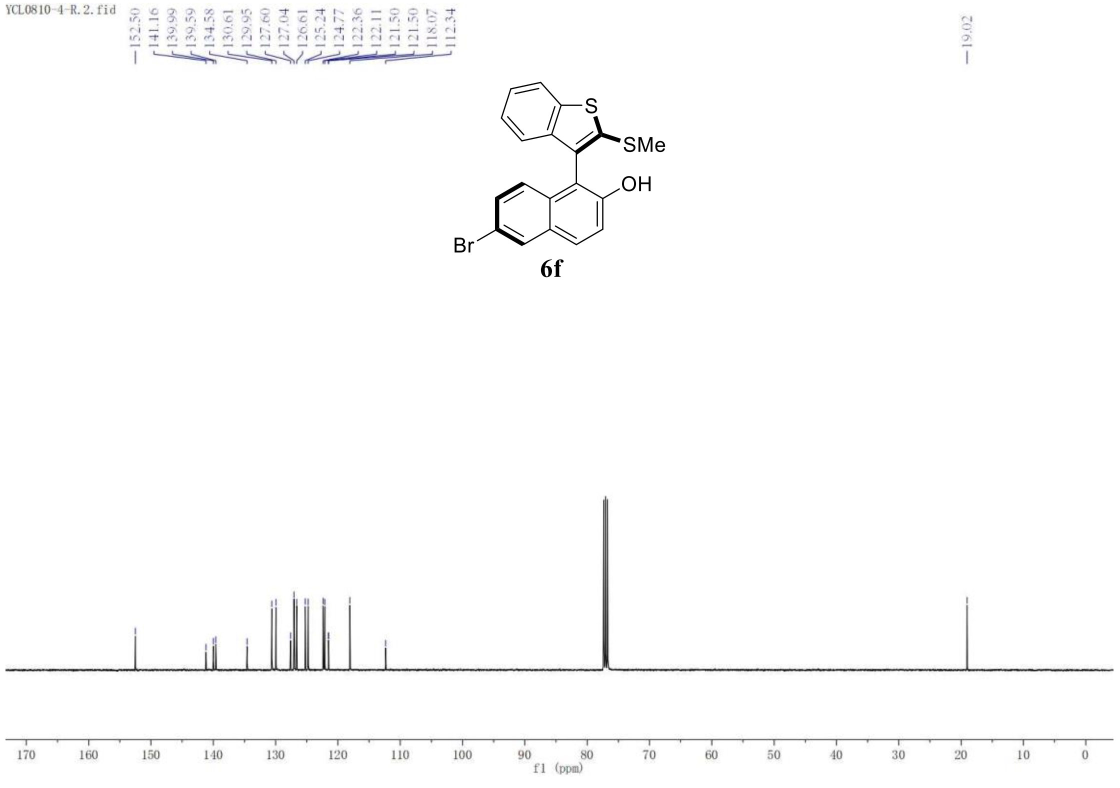
YCL0810-4-R.2.fid
152.50
141.16
139.99
139.59
134.58
130.61
129.95
127.60
127.04
126.61
125.24
124.77
122.36
122.11
121.50
118.07
112.34
19.02
S
SMe
OH
Br
6f
f1 (ppm)

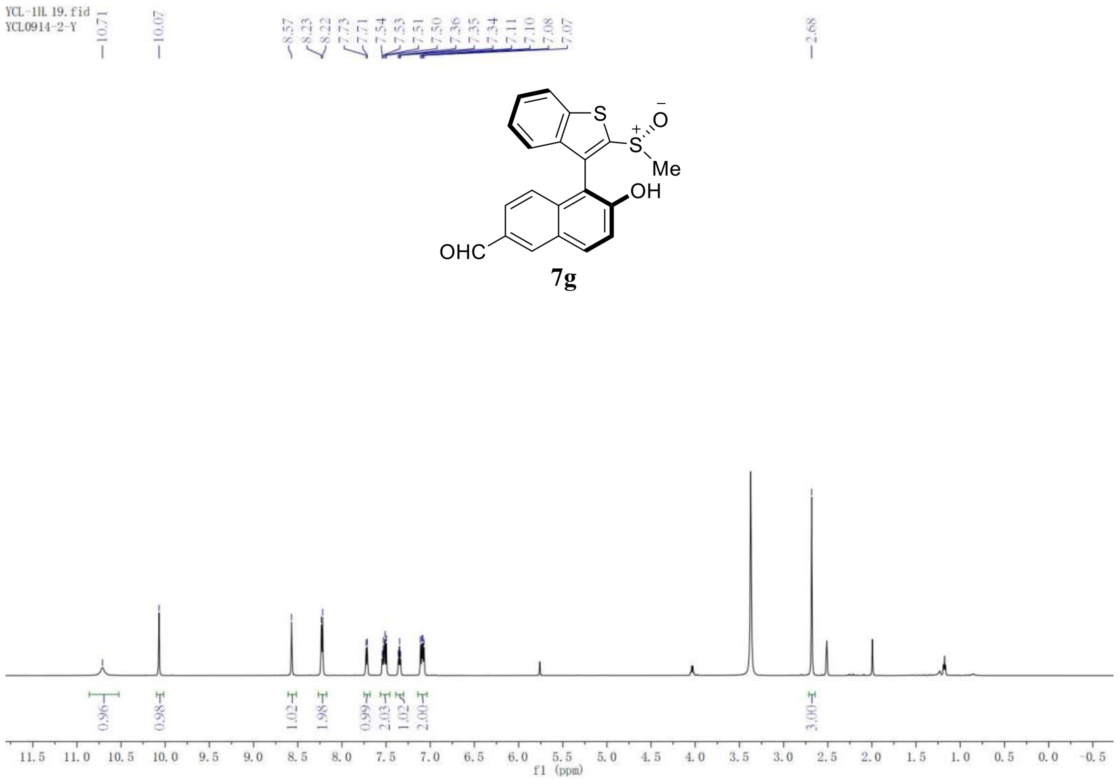
YCL-1H.19.fid
YCL0914-2-Y
10.71
10.07
8.57
8.23
8.22
7.73
7.71
7.54
7.53
7.51
7.50
7.36
7.35
7.34
7.11
7.10
7.08
7.07
2.68
S
O
Me
OH
OHC
7g
0.96
0.98
1.02
1.98
0.99
2.03
1.02
2.00
3.00
f1 (ppm)

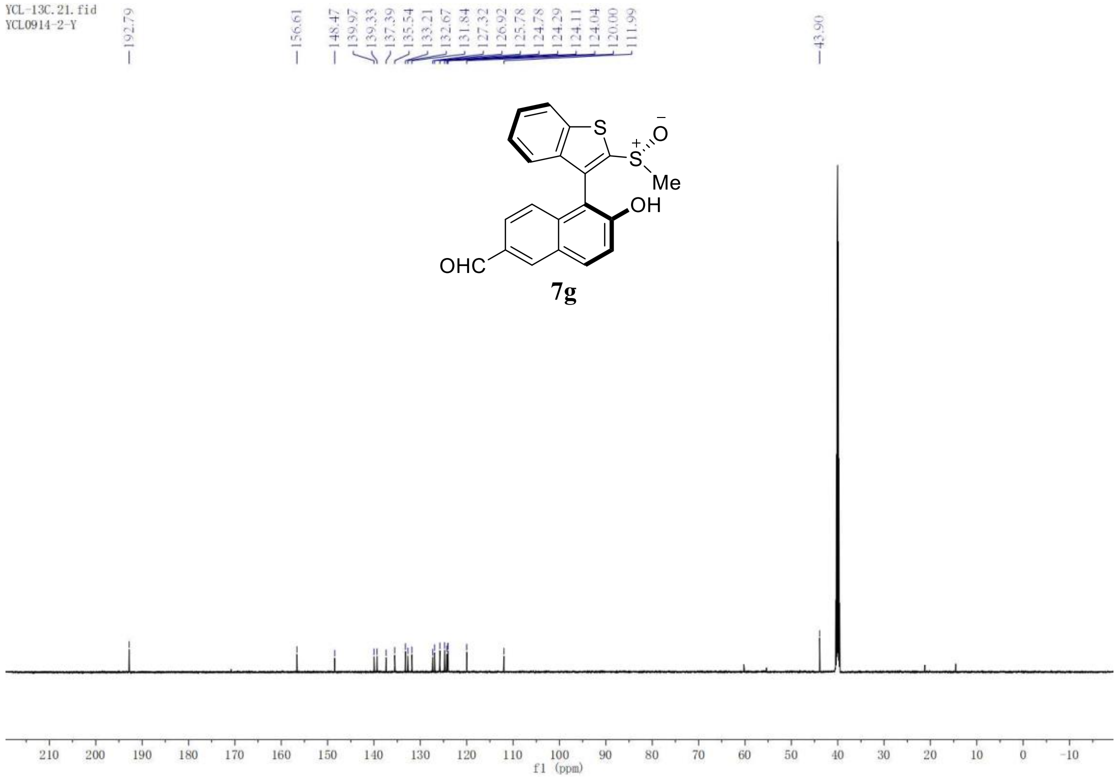
YCL-13C.21.fid
YCL0914-2-Y
192.79
156.61
148.47
139.97
139.33
137.39
135.54
133.21
132.67
131.84
127.32
126.92
125.78
124.78
124.29
124.11
124.04
120.00
111.99
43.90
S
O
Me
OH
OHC
7g
f1 (ppm)

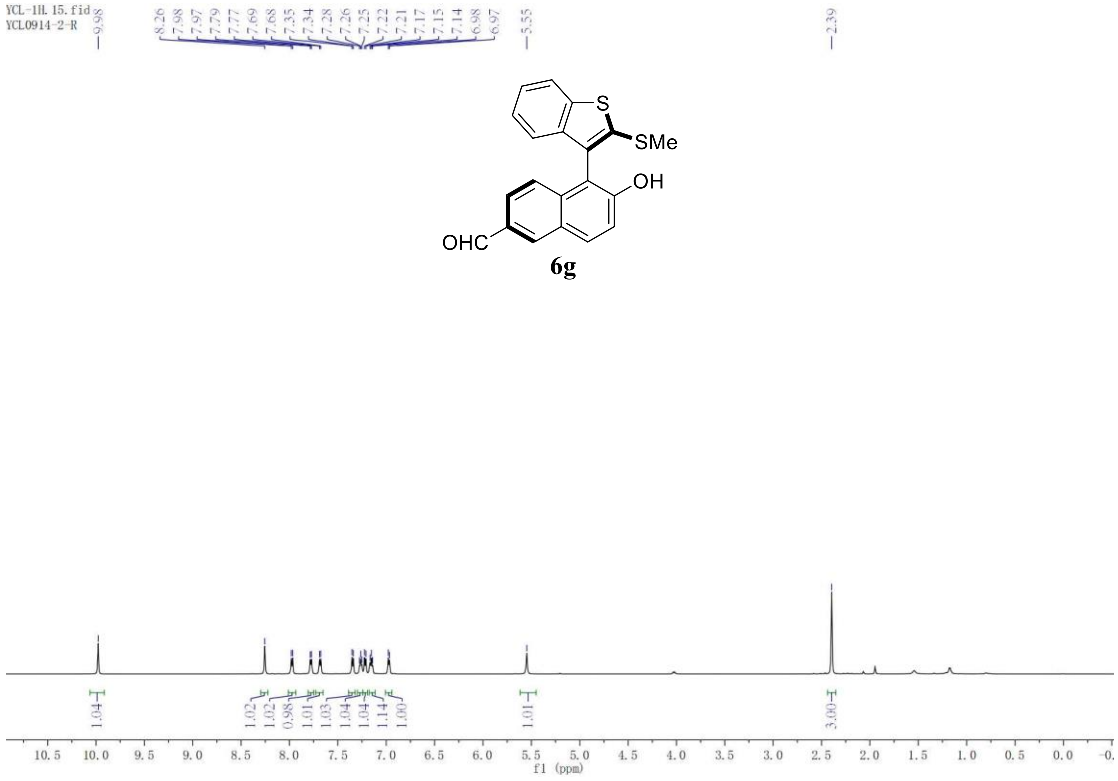
YCL-1H.15.fid
YCL0914-2-R
9.98
8.26 7.98 7.97 7.79 7.77 7.69 7.68 7.35 7.34 7.28 7.26 7.25 7.22 7.21 7.17 7.15 7.14 6.98 6.97
5.55
2.39
S
SMe
OH
OHC
6g
1.04
1.02 1.02 0.98 1.01 1.03 1.04 1.04 1.14 1.00
1.01
3.00
10.5 10.0 9.5 9.0 8.5 8.0 7.5 7.0 6.5 6.0 5.5 5.0 4.5 4.0 3.5 3.0 2.5 2.0 1.5 1.0 0.5 0.0
f1 (ppm)

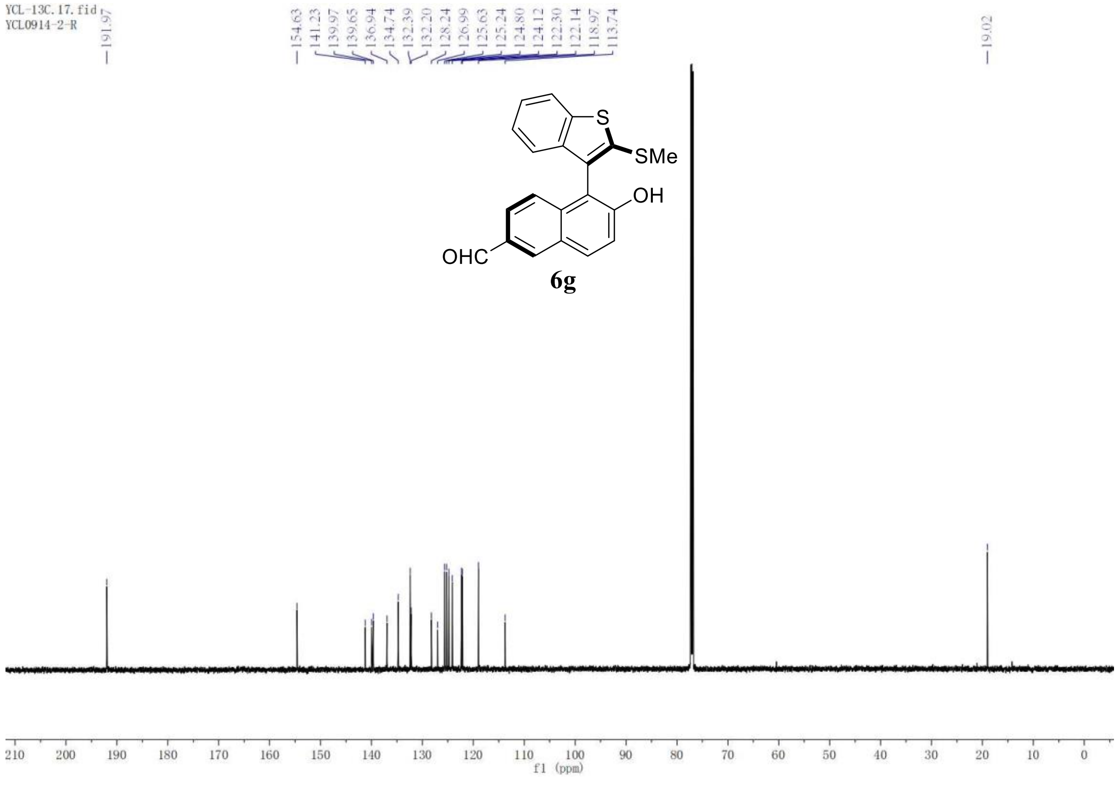
YCL-13C.17.fid
YCL0914-2-R
191.97
154.63 141.23 139.97 139.65 136.94 134.74 132.39 132.20 128.24 126.99 125.63 125.24 124.80 124.12 122.30 122.14 118.97 113.74
19.02
S
SMe
OH
OHC
6g
210 200 190 180 170 160 150 140 130 120 110 100 90 80 70 60 50 40 30 20 10 0
f1 (ppm)

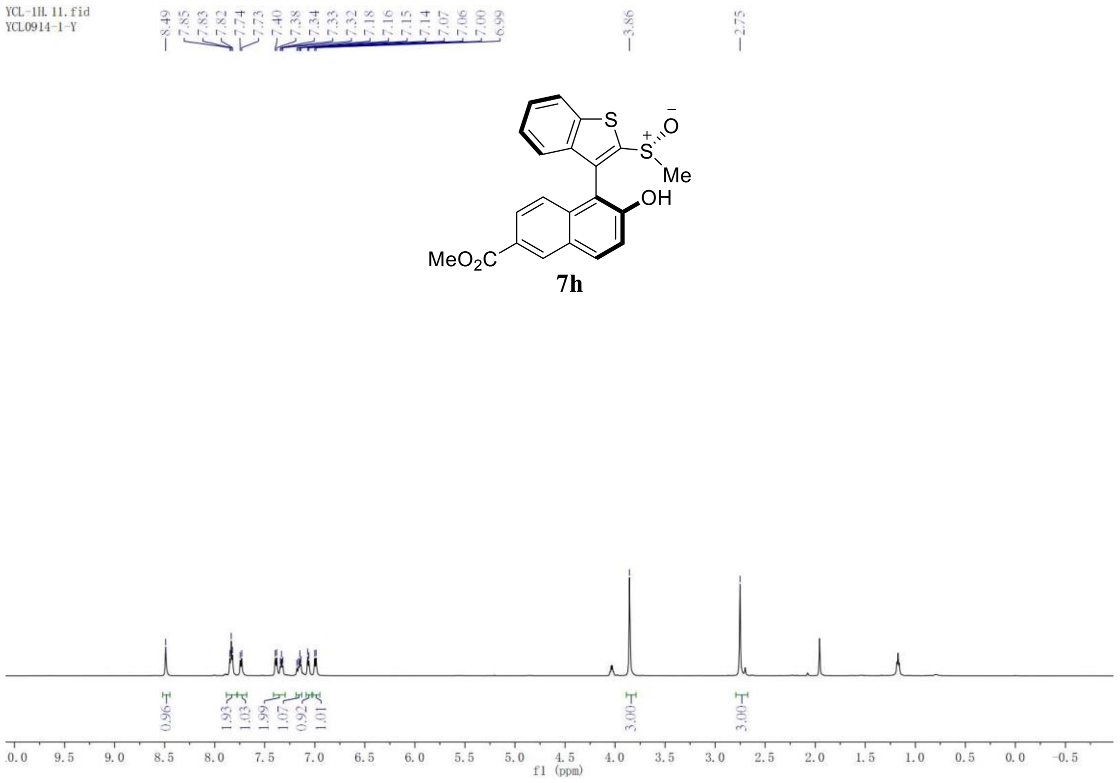
YCL-1H.11.fid
YCL0914-1-Y
8.49
7.85
7.83
7.82
7.74
7.73
7.40
7.38
7.34
7.33
7.32
7.18
7.16
7.15
7.14
7.07
7.06
7.00
6.99
3.86
2.75
S
O
S
Me
OH
MeO2C
7h
0.96
1.93
1.03
1.99
1.07
0.92
1.01
3.00
3.00
f1 (ppm)

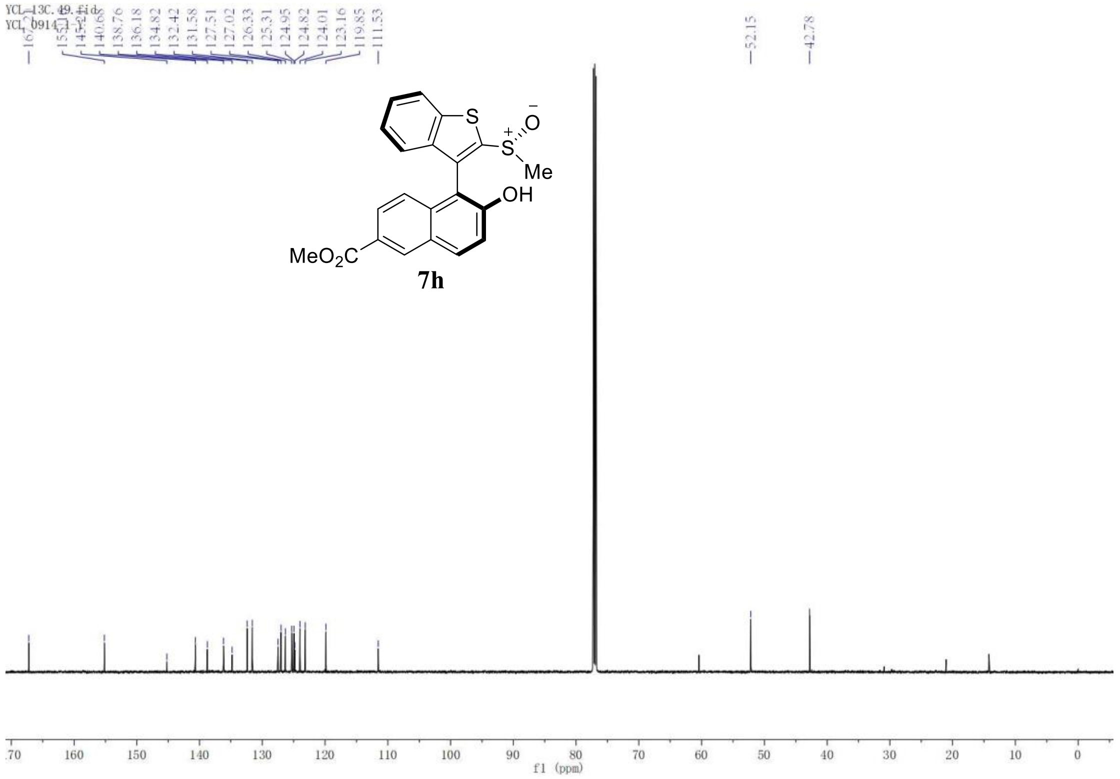
138.76
136.18
134.82
132.42
131.58
127.51
127.02
126.33
125.31
124.95
124.82
124.01
123.16
119.85
111.53
52.15
42.78
S
O
S
Me
OH
MeO2C
7h
f1 (ppm)

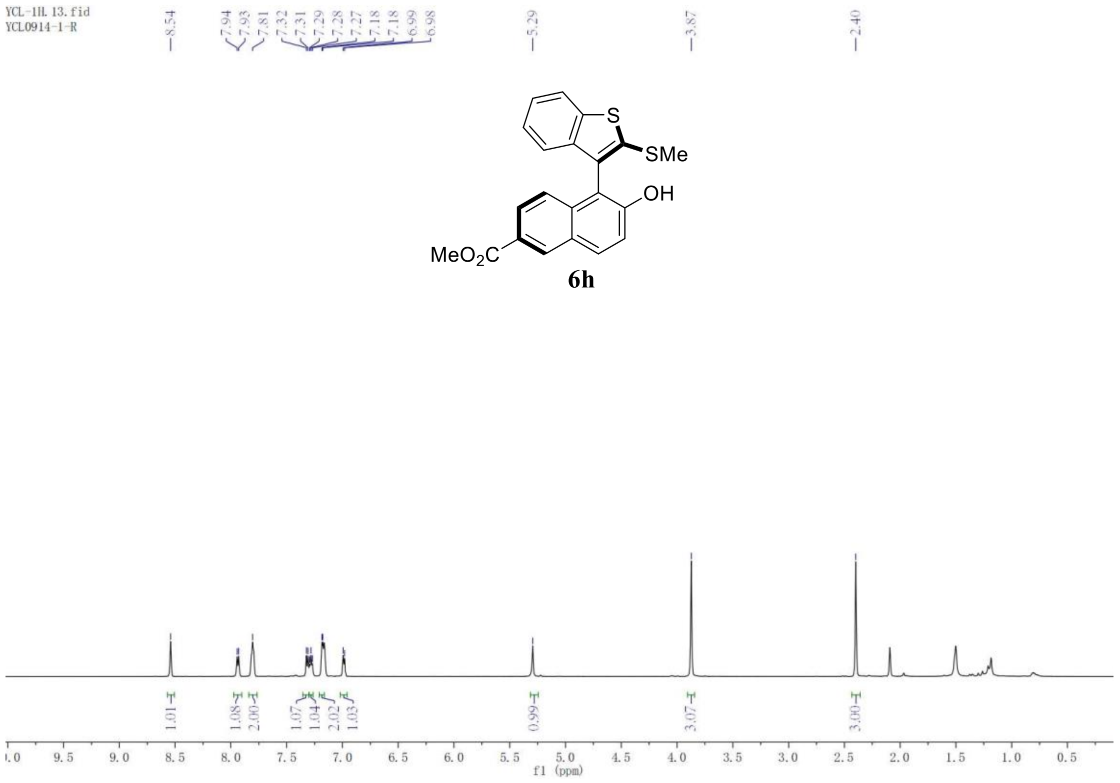
YCL-1H.13.fid
YCL0914-1-R
8.54
7.94
7.93
7.81
7.32
7.31
7.29
7.28
7.27
7.18
7.18
6.99
6.98
5.29
3.87
2.40
S
SMe
OH
MeO2C
6h
1.01
1.08
2.00
1.07
1.04
2.02
1.03
0.99
3.07
3.00
f1 (ppm)

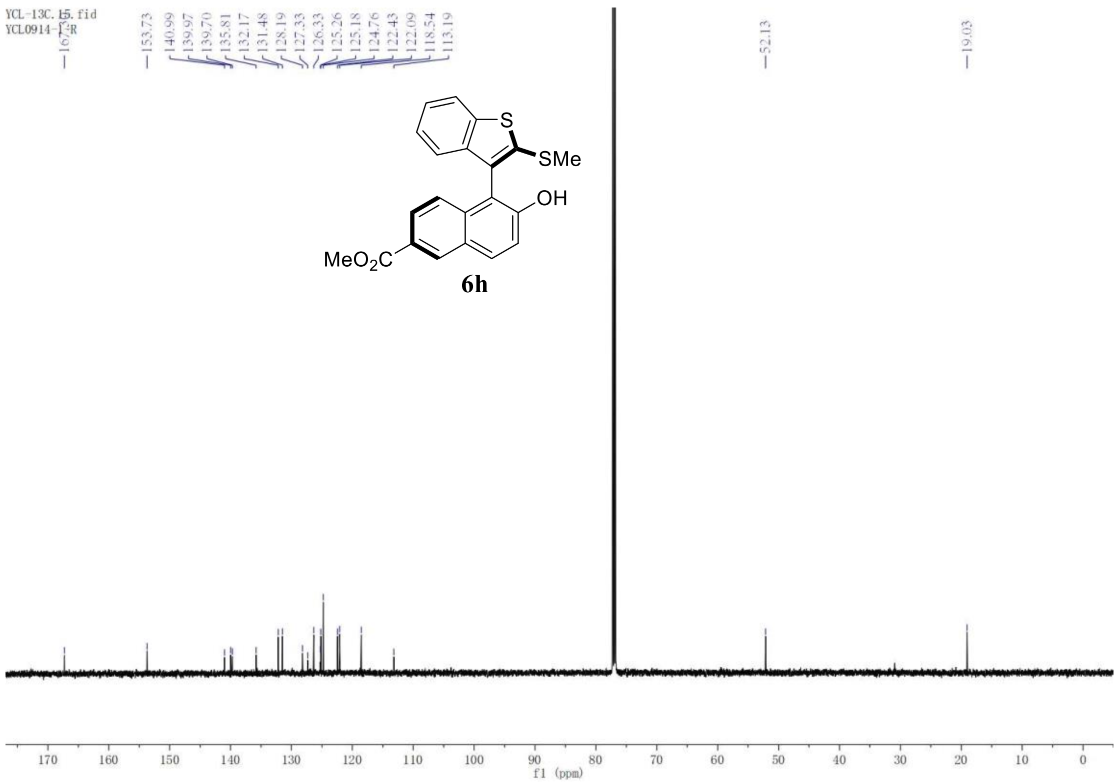
YCL-13C.15.fid
YCL0914-1-R
153.73
140.99
139.97
139.70
135.81
132.17
131.48
128.19
127.33
126.33
125.26
125.18
124.76
122.43
122.09
118.54
113.19
52.13
19.03
S
SMe
OH
MeO2C
6h
f1 (ppm)

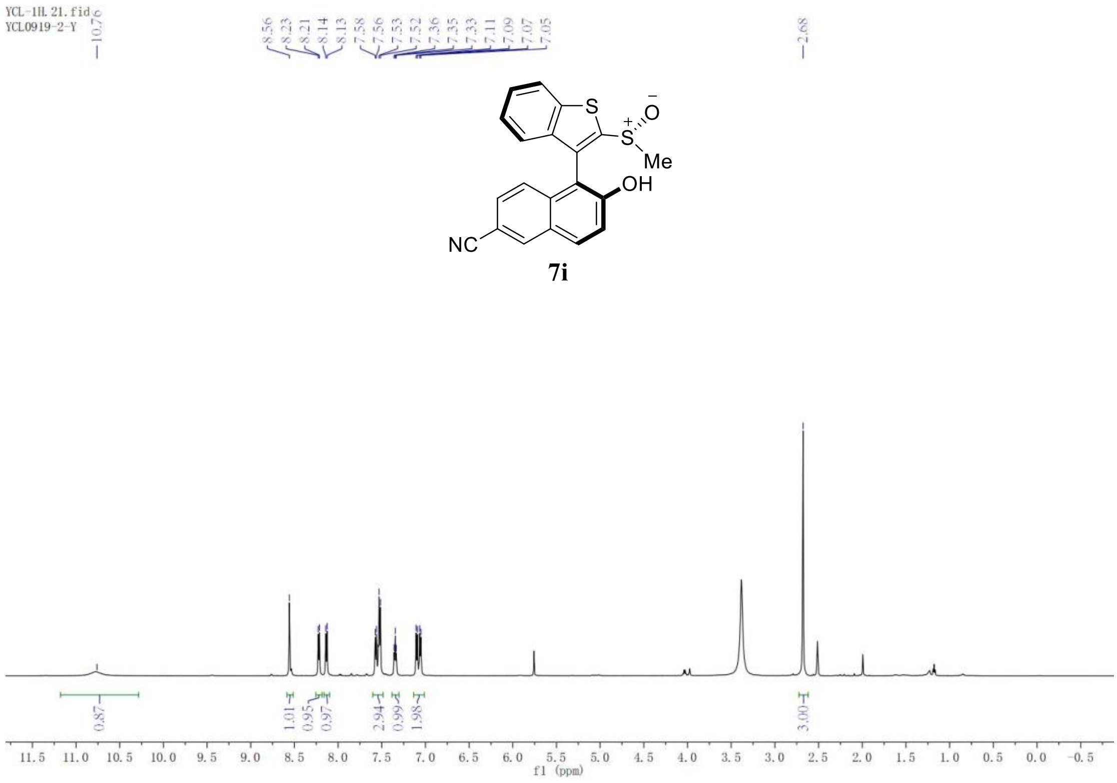
YCL-1H.21.fid
YCL0919-2-Y
10.76
8.56 8.23 8.21 8.14 8.13 7.58 7.56 7.53 7.52 7.36 7.35 7.33 7.11 7.09 7.07 7.05
2.68
S
O
Me
OH
NC
7i
0.87
1.01 0.95 0.97
2.94 0.99 1.98
3.00
11.5 11.0 10.5 10.0 9.5 9.0 8.5 8.0 7.5 7.0 6.5 6.0 5.5 5.0 4.5 4.0 3.5 3.0 2.5 2.0 1.5 1.0 0.5 0.0 -0.5
f1 (ppm)

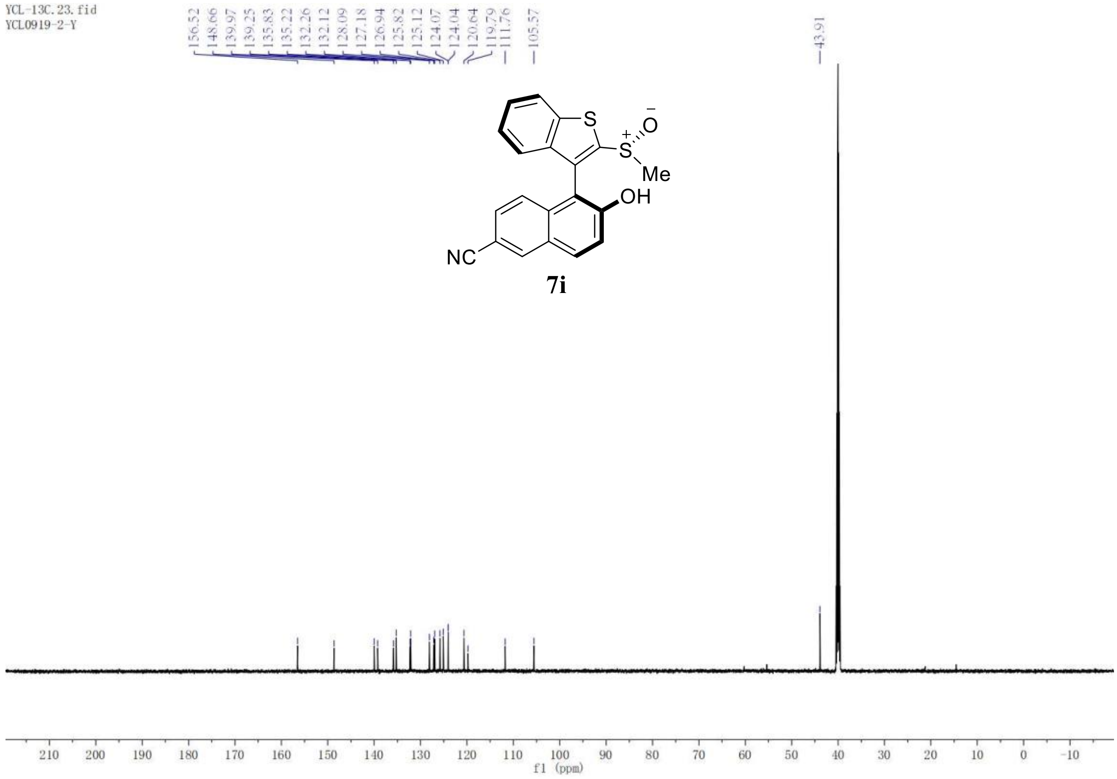
YCL-13C.23.fid
YCL0919-2-Y
156.52 148.66 139.97 139.25 135.83 135.22 132.26 132.12 128.09 127.18 126.94 125.82 125.12 124.07 124.04 120.64 119.79 111.76 105.57
43.91
S
O
Me
OH
NC
7i
210 200 190 180 170 160 150 140 130 120 110 100 90 80 70 60 50 40 30 20 10 0 -10
f1 (ppm)

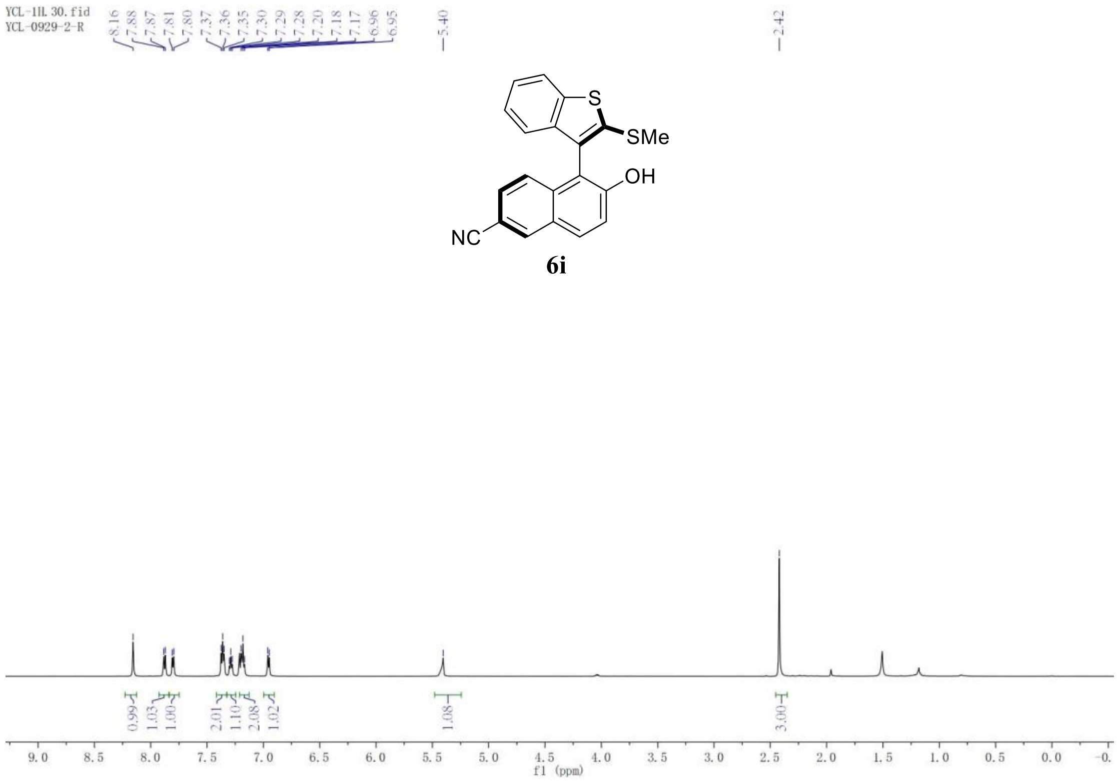
YCL-1H 30.fid
YCL-0929-2-R
8.16 7.88 7.87 7.81 7.80 7.37 7.36 7.35 7.30 7.29 7.28 7.20 7.18 7.17 6.96 6.95
5.40
2.42
S
SMe
OH
NC
6i
0.99 1.03 1.00 2.01 1.10 2.08 1.02 1.08 3.00
f1 (ppm)

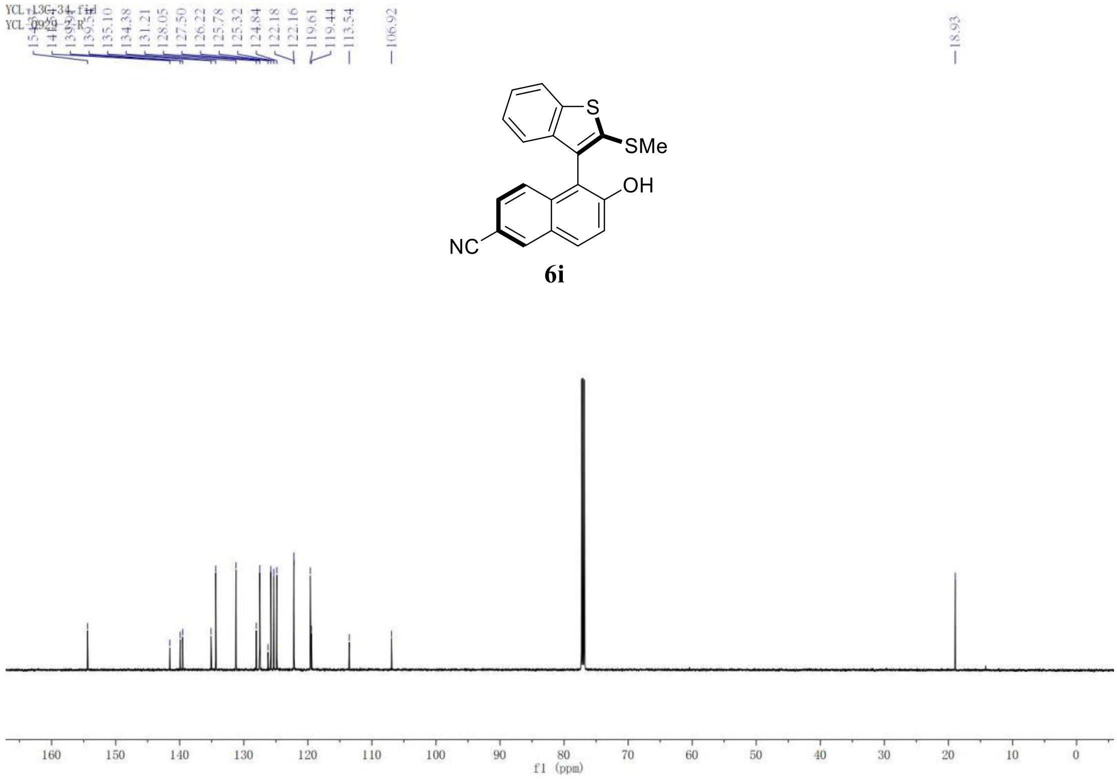
135.10 134.38 131.21 128.05 127.50 126.22 125.78 125.32 124.84 122.18 122.16 119.61 119.44 113.54
106.92
18.93
S
SMe
OH
NC
6i
f1 (ppm)

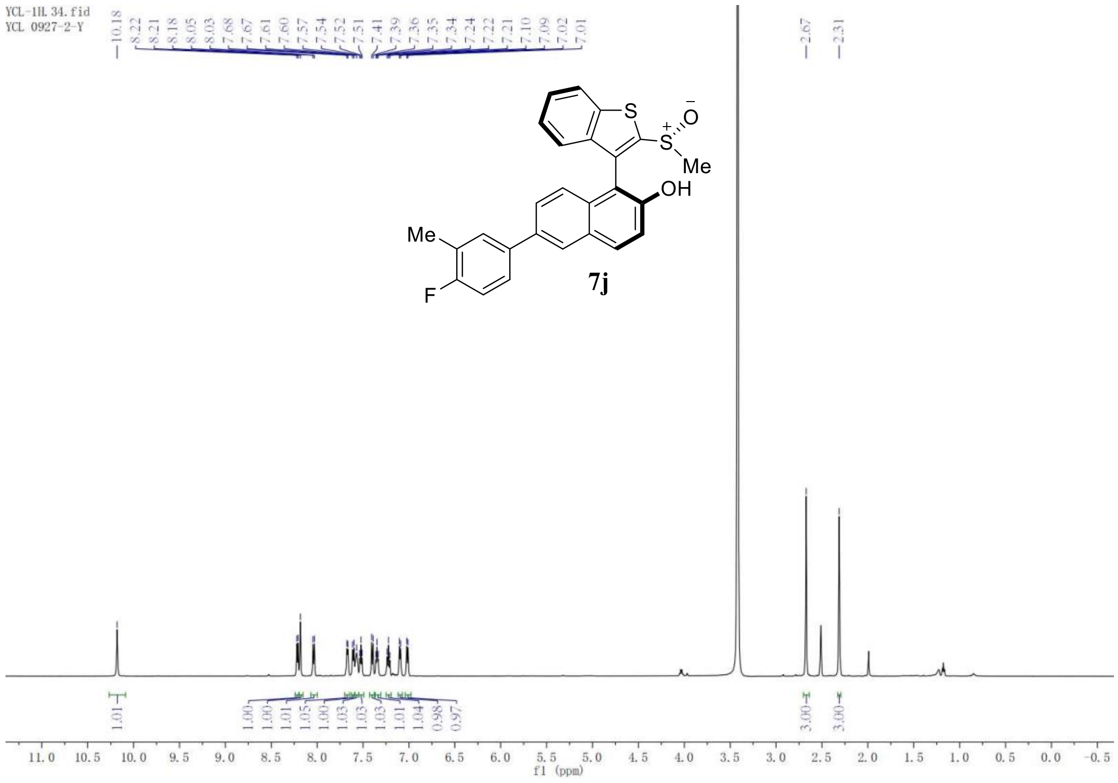
YCL-1H.34.fid
YCL 0927-2-Y
10.18
8.22
8.21
8.18
8.05
8.03
7.68
7.67
7.61
7.60
7.57
7.54
7.52
7.51
7.41
7.39
7.36
7.35
7.34
7.24
7.22
7.21
7.10
7.09
7.02
7.01
2.67
2.31
S
O
Me
OH
Me
F
7j
1.01
1.00
1.00
1.01
1.05
1.00
1.03
1.03
1.03
1.01
1.04
0.98
0.97
3.00
3.00
f1 (ppm)

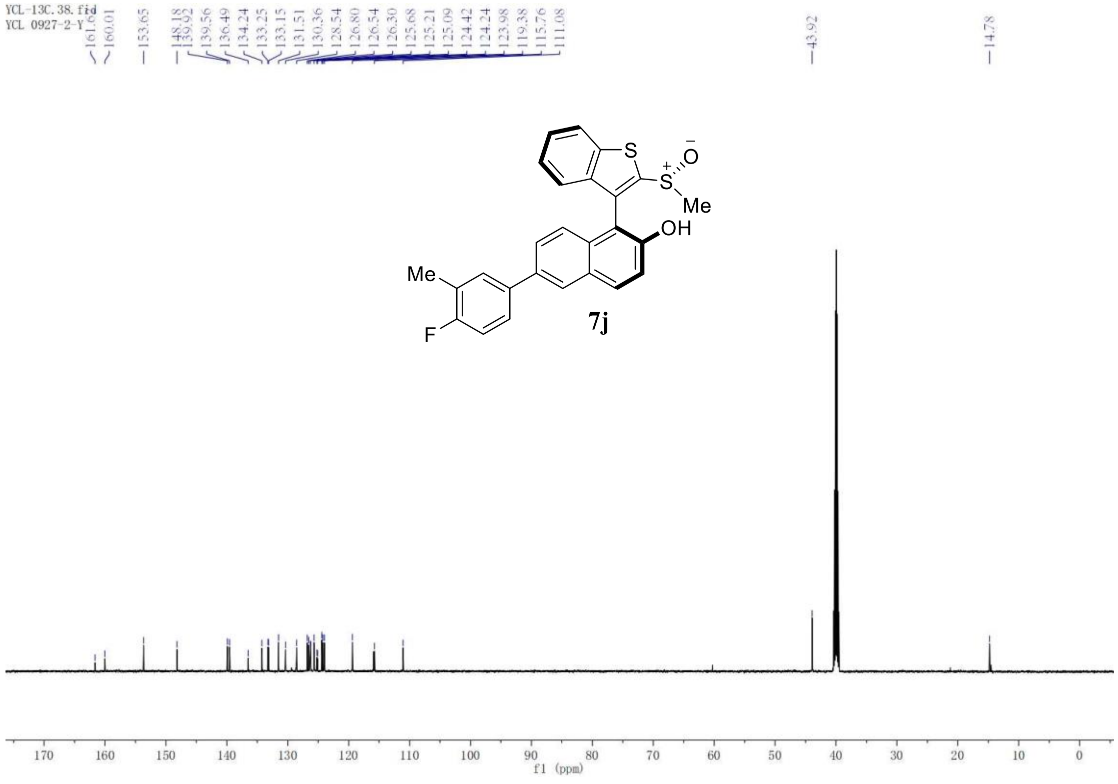
YCL-13C.38.fid
YCL 0927-2-Y
160.01
153.65
148.18
139.92
139.56
136.49
134.24
133.25
133.15
131.51
130.36
128.54
126.80
126.54
126.30
125.68
125.21
125.09
124.42
124.24
123.98
119.38
115.76
111.08
43.92
14.78
S
O
Me
OH
Me
F
7j
f1 (ppm)

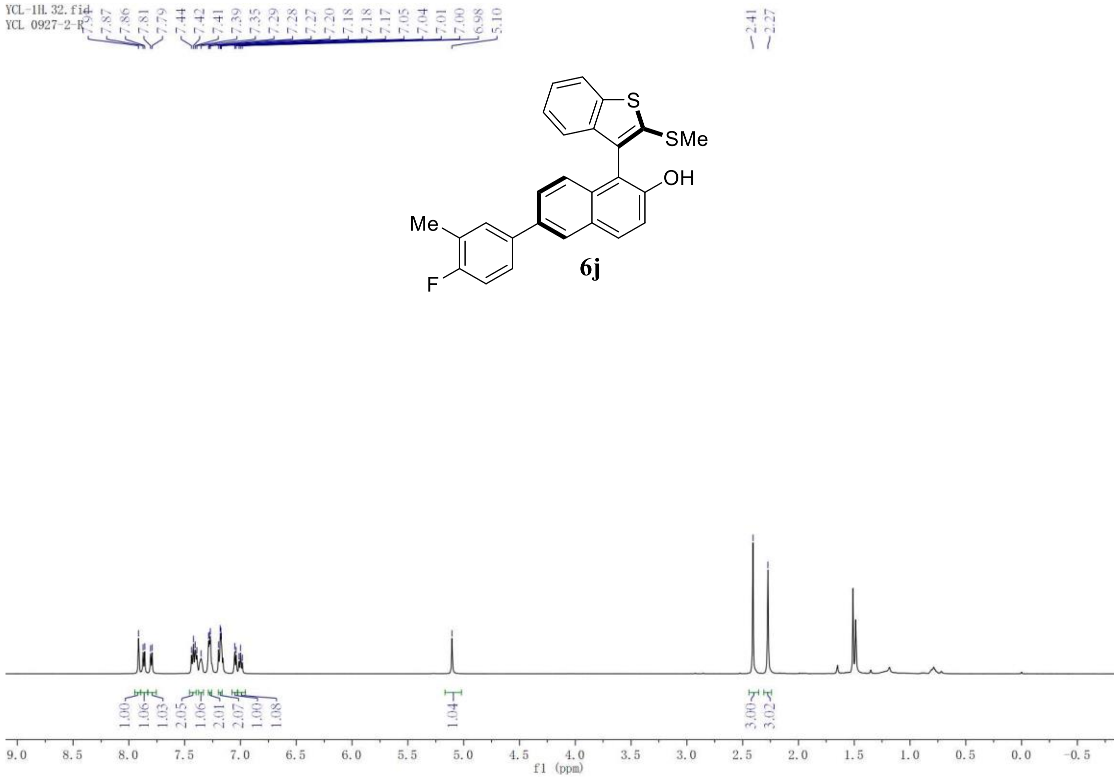
YCL-1H.32.fid
YCL 0927-2-R
7.89 7.87 7.86 7.81 7.79 7.44 7.42 7.41 7.39 7.35 7.29 7.28 7.27 7.20 7.18 7.18 7.17 7.05 7.04 7.01 7.00 6.98 5.10
2.41 2.27
S
SMe
OH
Me
F
6j
1.00 1.06 1.03 2.05 1.06 2.01 2.07 1.00 1.08
1.04
3.00 3.02
9.0 8.5 8.0 7.5 7.0 6.5 6.0 5.5 5.0 4.5 4.0 3.5 3.0 2.5 2.0 1.5 1.0 0.5 0.0 -0.5
f1 (ppm)

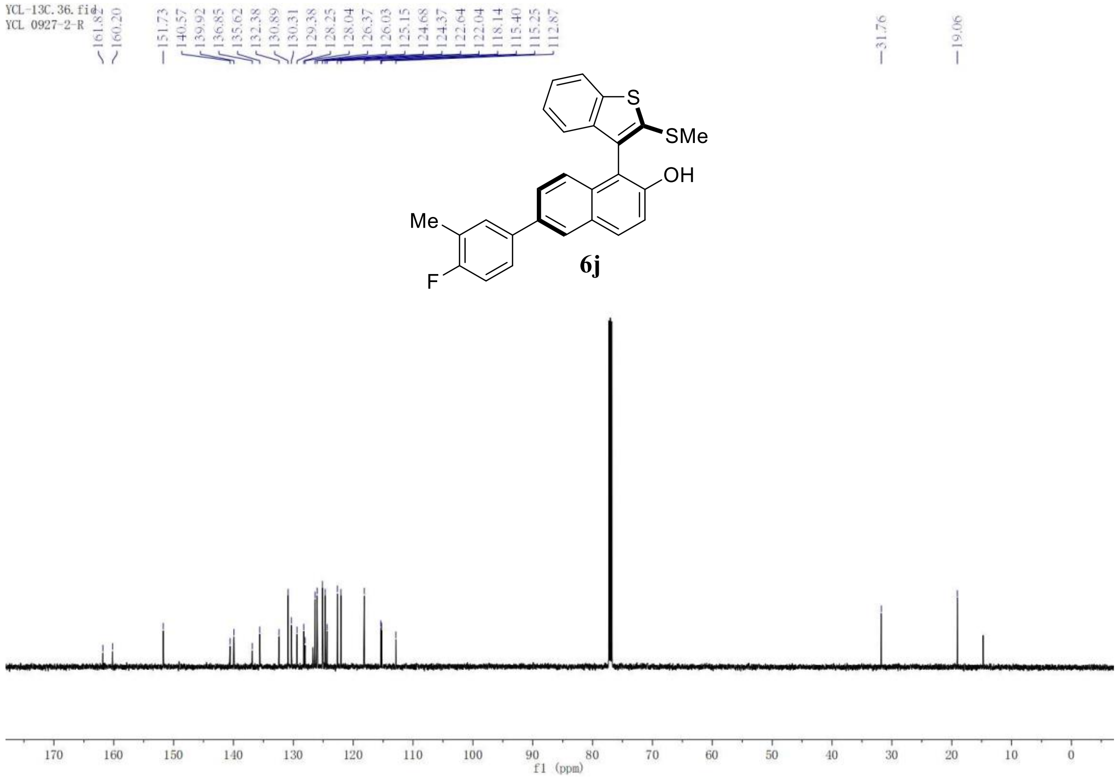
YCL-13C.36.fid
YCL 0927-2-R
161.82 160.20 151.73 140.57 139.92 136.85 135.62 132.38 130.89 130.31 129.38 128.25 128.04 126.37 126.03 125.15 124.68 124.37 122.64 122.04 118.14 115.40 115.25 112.87
31.76
19.06
S
SMe
OH
Me
F
6j
170 160 150 140 130 120 110 100 90 80 70 60 50 40 30 20 10 0
f1 (ppm)

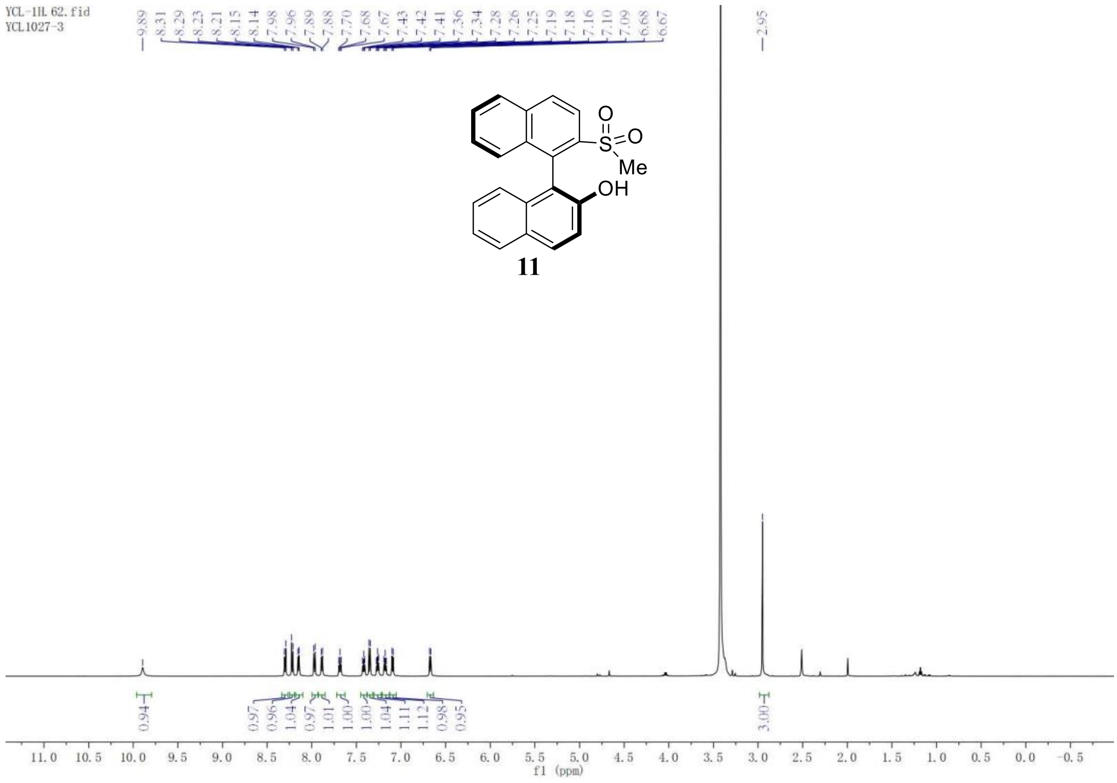
YCL-1H 62.fid
YCL1027-3
9.89
8.31
8.29
8.23
8.21
8.15
8.14
7.98
7.96
7.89
7.88
7.70
7.68
7.67
7.43
7.42
7.41
7.36
7.34
7.28
7.26
7.25
7.19
7.18
7.16
7.10
7.09
6.68
6.67
2.95
O
S
O
Me
OH
11
0.94
0.97
0.96
1.04
0.97
1.01
1.00
1.00
1.04
1.11
1.12
0.98
0.95
3.00
11.0 10.5 10.0 9.5 9.0 8.5 8.0 7.5 7.0 6.5 6.0 5.5 5.0 4.5 4.0 3.5 3.0 2.5 2.0 1.5 1.0 0.5 0.0 -0.5
f1 (ppm)

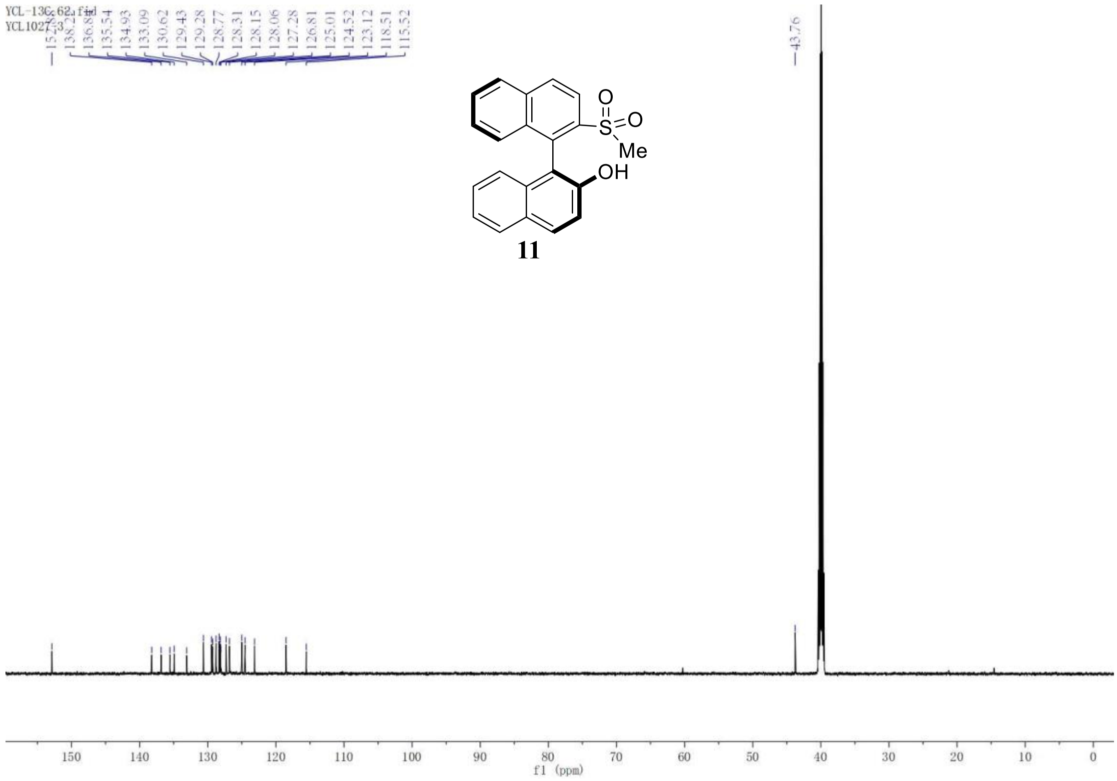
YCL1027-3
136.88
135.54
134.93
133.09
130.62
129.43
129.28
128.77
128.31
128.15
128.06
127.28
126.81
125.01
124.52
123.12
118.51
115.52
43.76
O
S
O
Me
OH
11
150 140 130 120 110 100 90 80 70 60 50 40 30 20 10 0
f1 (ppm)

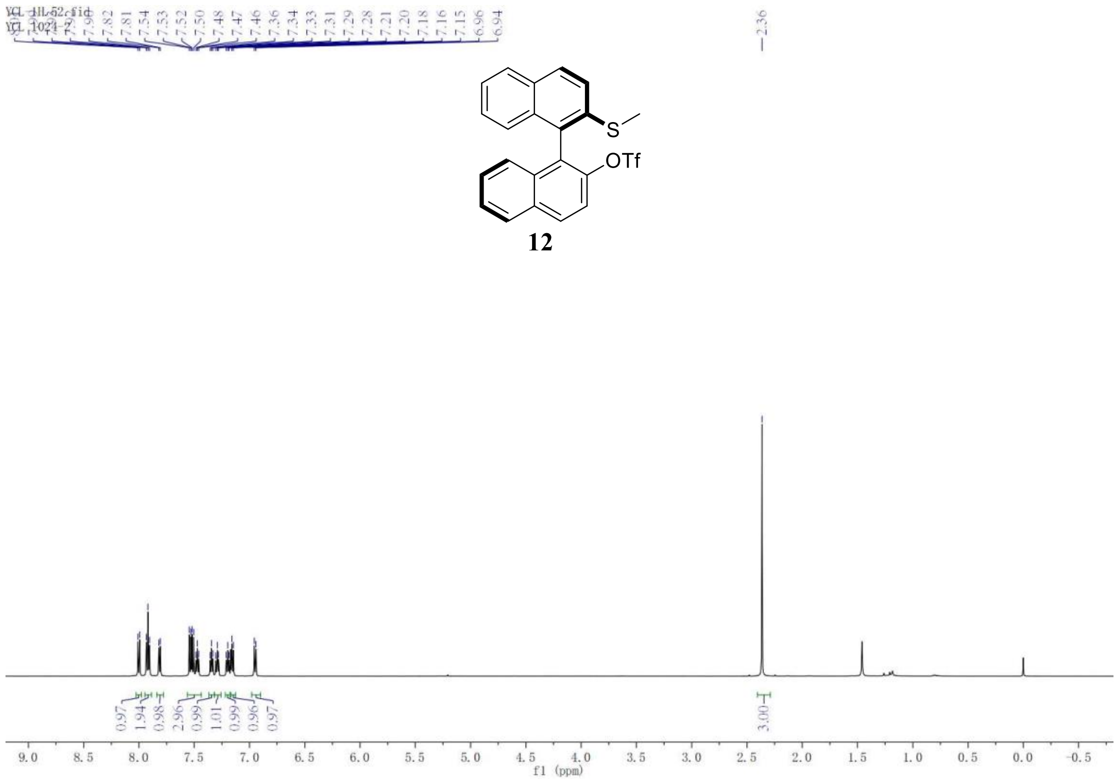
YCL-1H.52.fid
YCL 1024-2
7.96 7.82 7.81 7.54 7.53 7.52 7.50 7.48 7.47 7.46 7.36 7.34 7.33 7.31 7.29 7.28 7.21 7.20 7.18 7.16 7.15 6.96 6.94
2.36
S
OTf
12
0.97 1.94 0.98 2.96 0.99 1.01 0.99 0.96 0.97
3.00
9.0 8.5 8.0 7.5 7.0 6.5 6.0 5.5 5.0 4.5 4.0 3.5 3.0 2.5 2.0 1.5 1.0 0.5 0.0 -0.5
f1 (ppm)

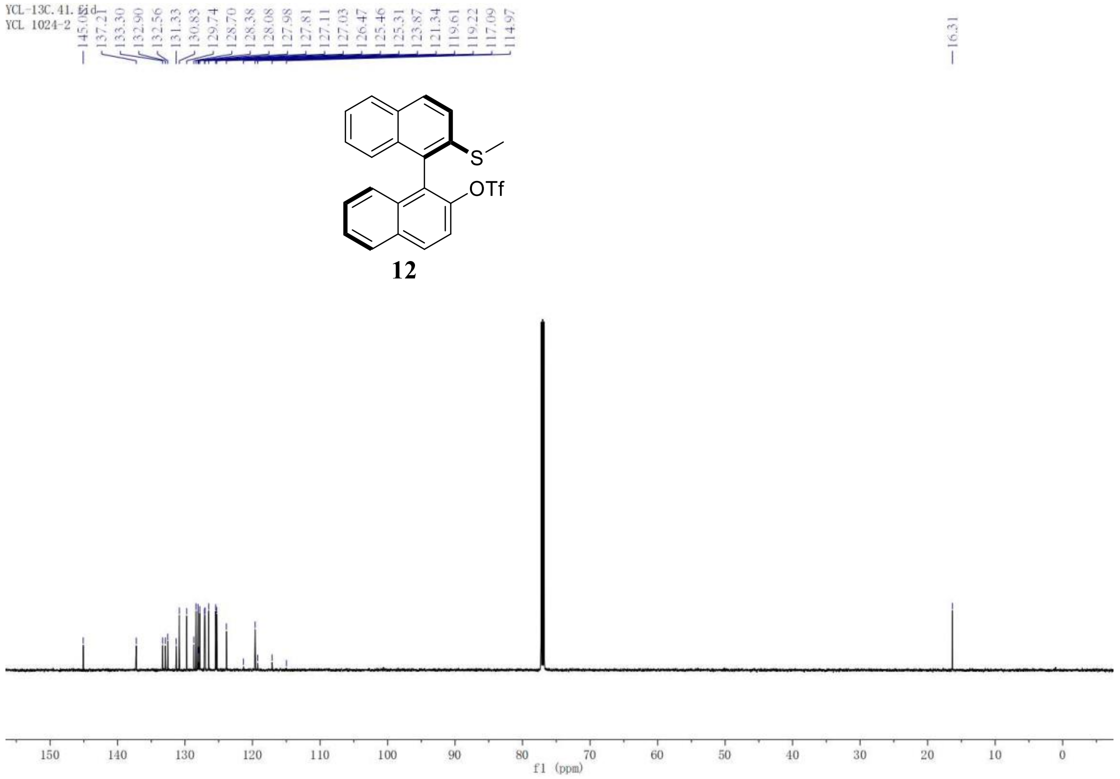
YCL-13C.41.fid
YCL 1024-2
145.05 137.21 133.30 132.90 132.56 131.33 130.83 129.74 128.70 128.38 128.08 127.98 127.81 127.11 127.03 126.47 125.46 125.31 123.87 121.34 119.61 119.22 117.09 114.97
16.31
S
OTf
12
150 140 130 120 110 100 90 80 70 60 50 40 30 20 10 0
f1 (ppm)

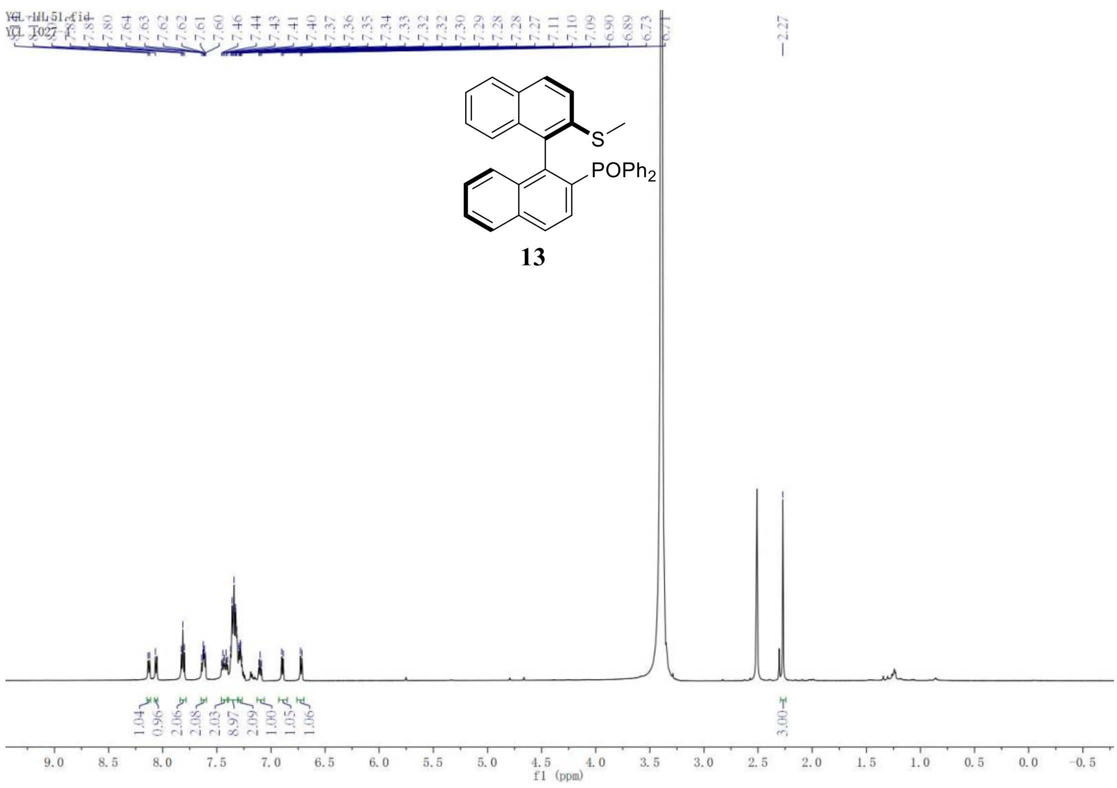

2.27
S
POPh2
13
f1 (ppm)

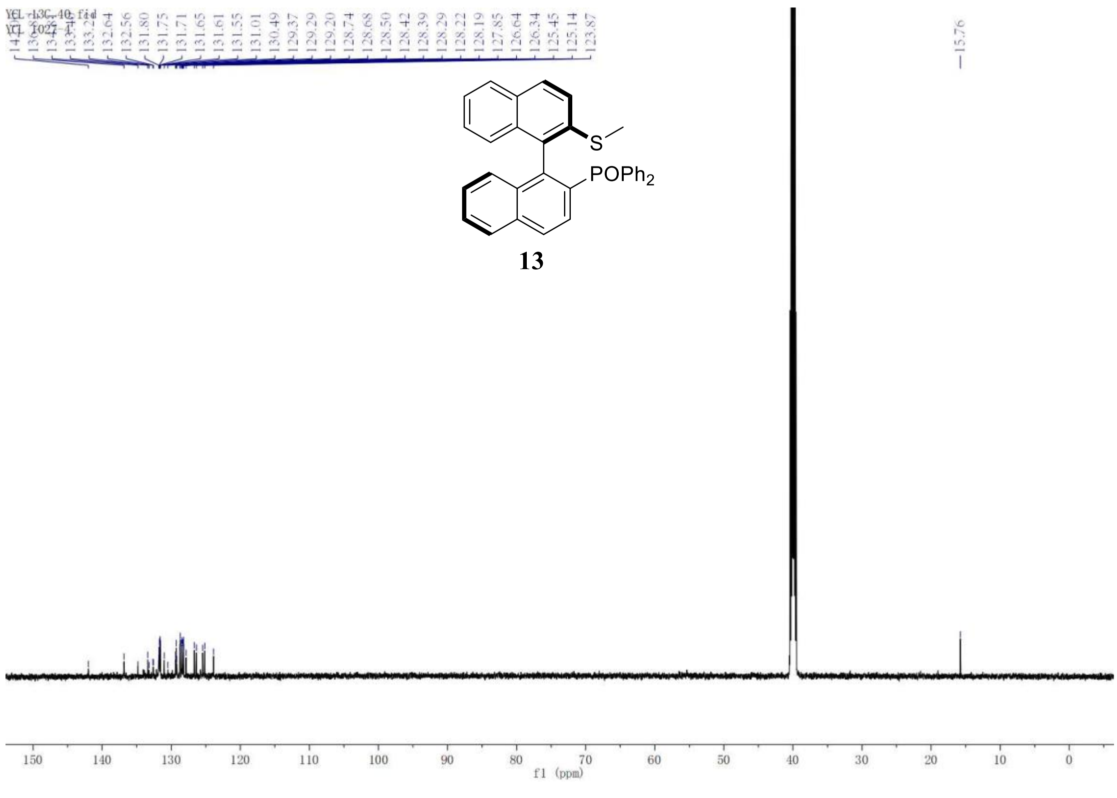

15.76
S
POPh2
13
f1 (ppm)

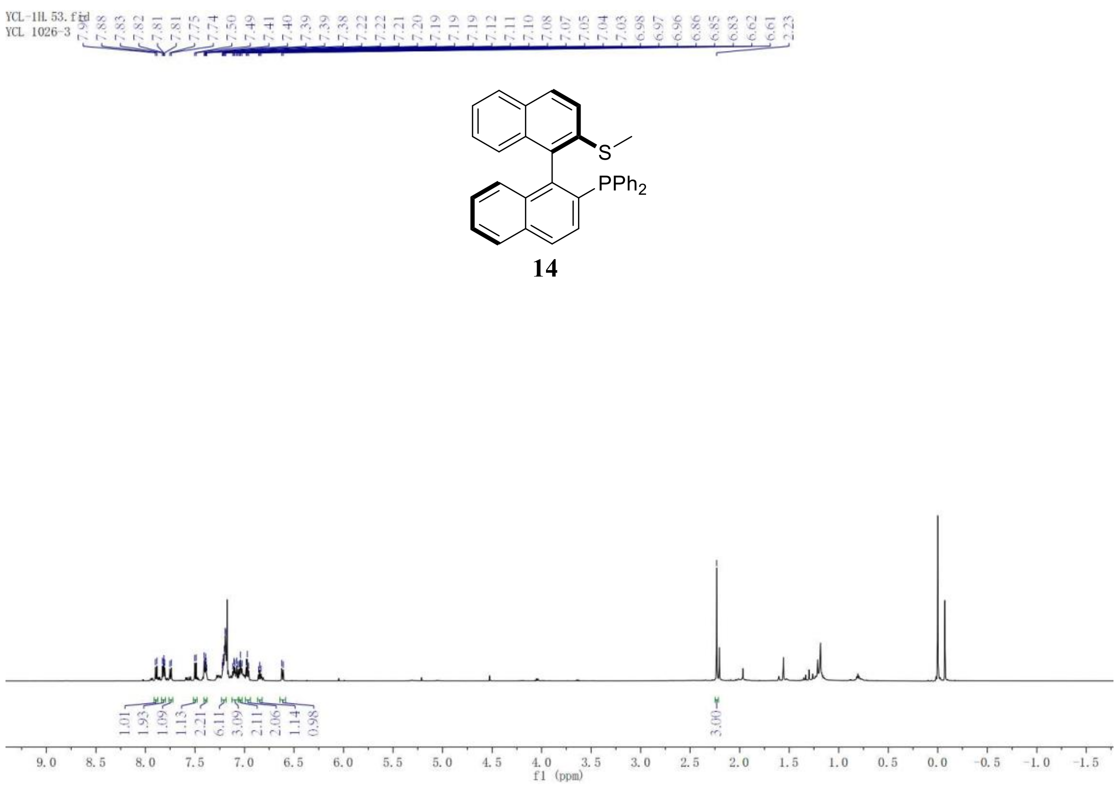
YCL-1H.53.fid
YCL 1026-3
7.88 7.83 7.82 7.81 7.81 7.75 7.74 7.50 7.49 7.41 7.40 7.39 7.39 7.38 7.22 7.22 7.21 7.20 7.19 7.19 7.12 7.11 7.10 7.08 7.07 7.05 7.04 7.03 6.98 6.97 6.96 6.86 6.85 6.83 6.62 6.61 2.23
PPh2
S
14
1.01 1.93 1.09 1.13 2.21 6.11 3.09 2.11 2.06 1.14 0.98
3.00
9.0 8.5 8.0 7.5 7.0 6.5 6.0 5.5 5.0 4.5 4.0 3.5 3.0 2.5 2.0 1.5 1.0 0.5 0.0 -0.5 -1.0 -1.5
f1 (ppm)

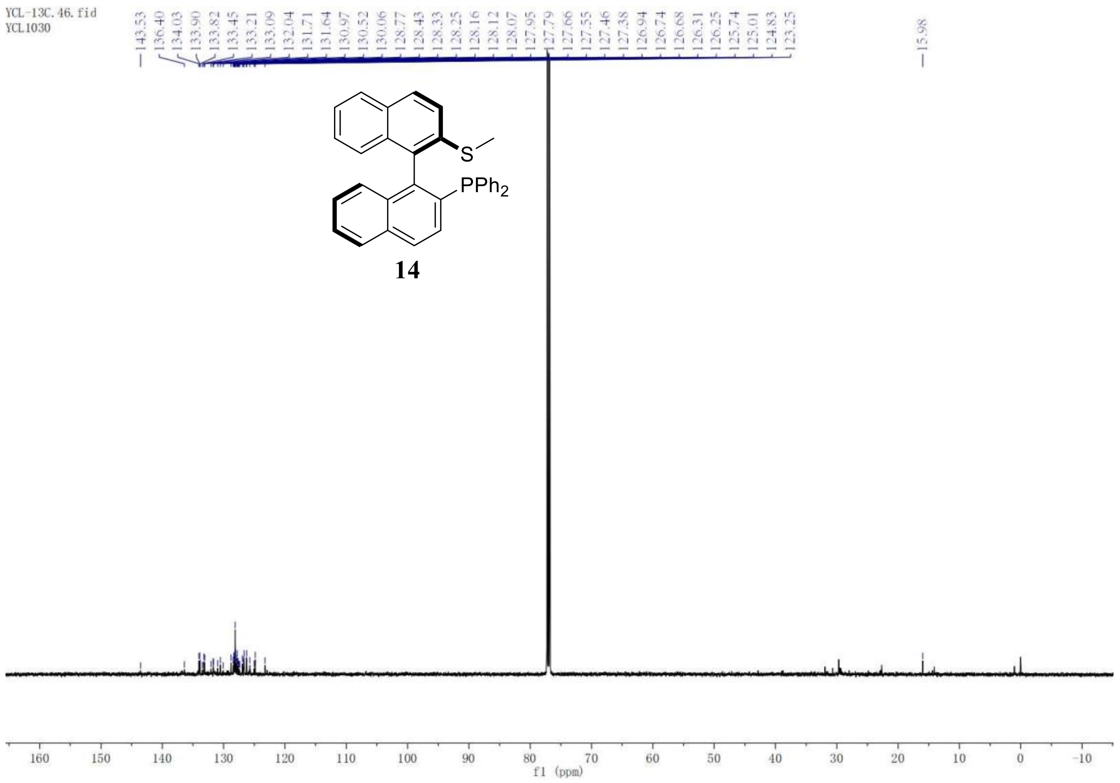
YCL-13C.46.fid
YCL1030
143.53 136.40 134.03 133.90 133.82 133.45 133.21 133.09 132.04 131.71 131.64 130.97 130.52 130.06 128.77 128.43 128.33 128.25 128.16 128.12 128.07 127.95 127.79 127.66 127.55 127.46 127.38 126.94 126.74 126.68 126.31 126.25 125.74 125.01 124.83 123.25
15.98
PPh2
S
14
160 150 140 130 120 110 100 90 80 70 60 50 40 30 20 10 0 -10
f1 (ppm)

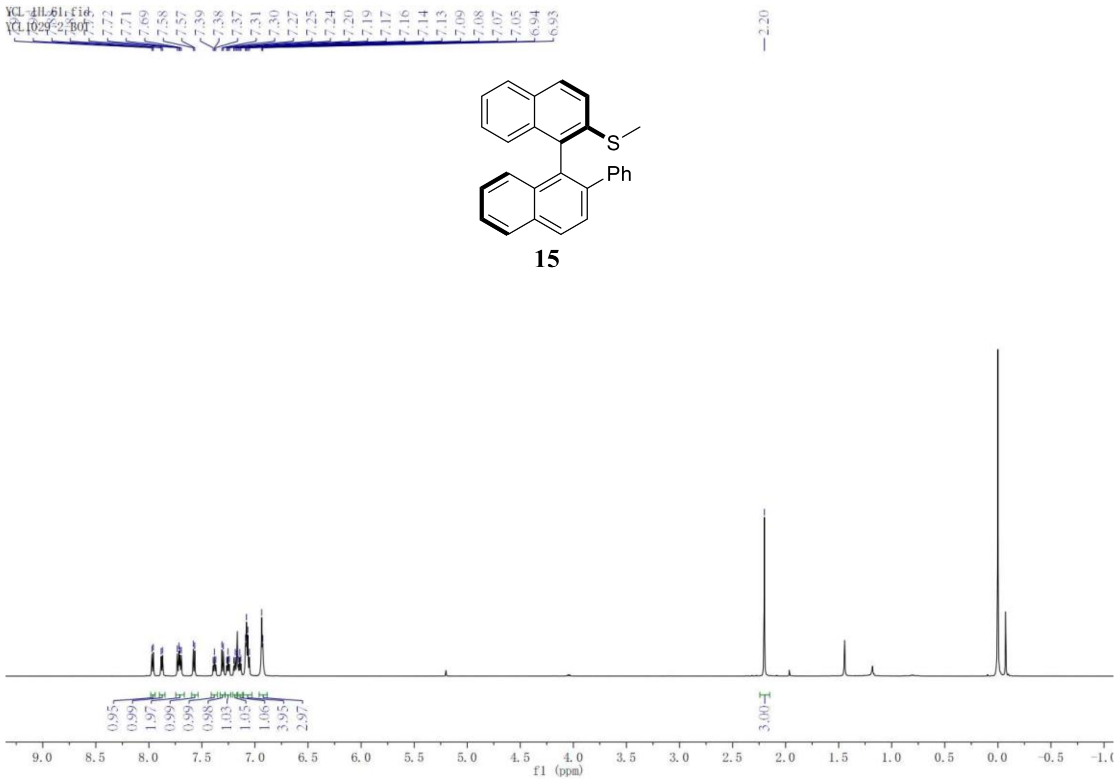
YCL-1H-61.fid
YCL1029-2-B01
7.72
7.71
7.69
7.58
7.57
7.39
7.38
7.37
7.31
7.30
7.27
7.25
7.24
7.20
7.19
7.17
7.16
7.14
7.13
7.09
7.08
7.07
7.05
6.94
6.93
2.20
S
Ph
15
0.95
0.99
1.97
0.99
0.99
0.98
1.03
1.05
1.06
3.95
2.97
3.00
f1 (ppm)

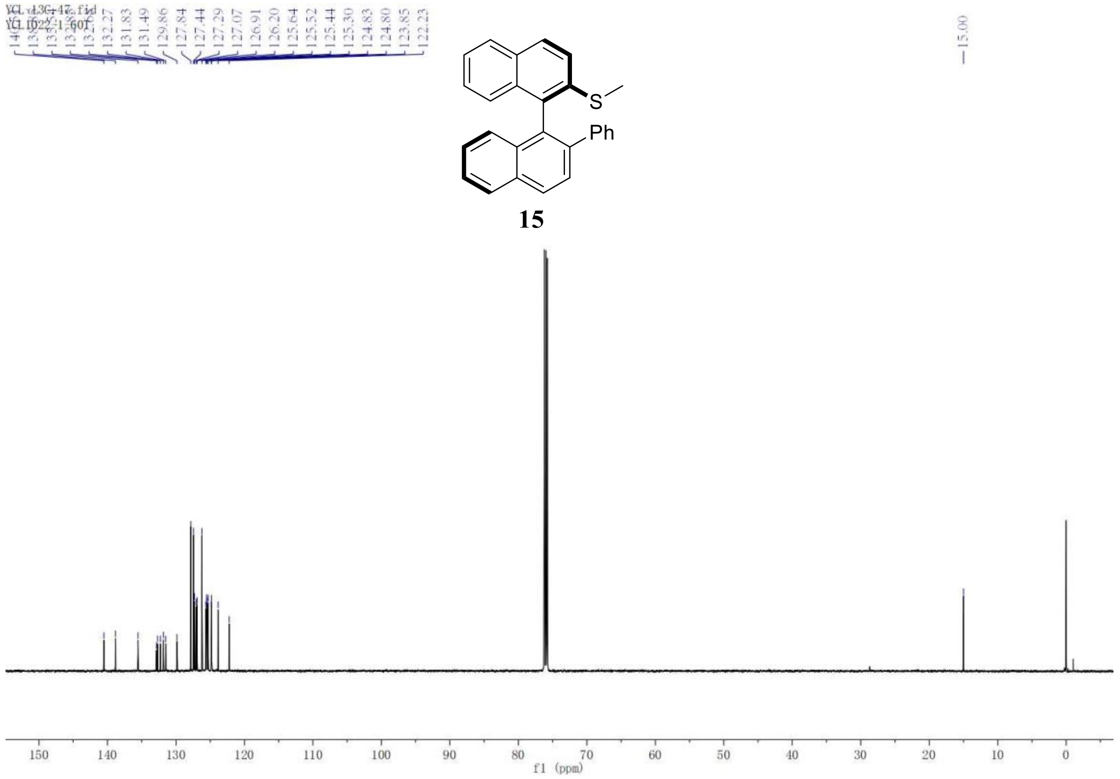
YCL-13C-47.fid
YCL1022-1-60T
132.27
131.83
131.49
129.86
127.84
127.44
127.29
127.07
126.91
126.20
125.64
125.52
125.44
125.30
124.83
124.80
123.85
122.23
15.00
S
Ph
15
f1 (ppm)

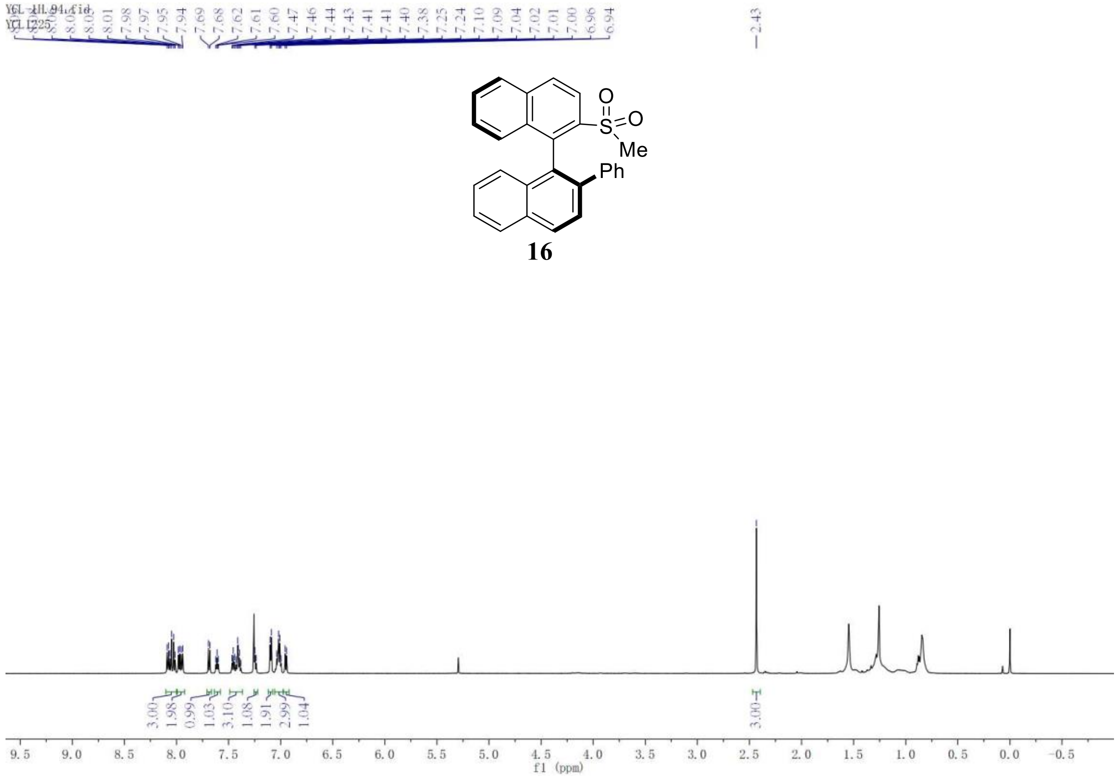

2.43
O
S
O
Me
Ph
16
3.00
f1 (ppm)

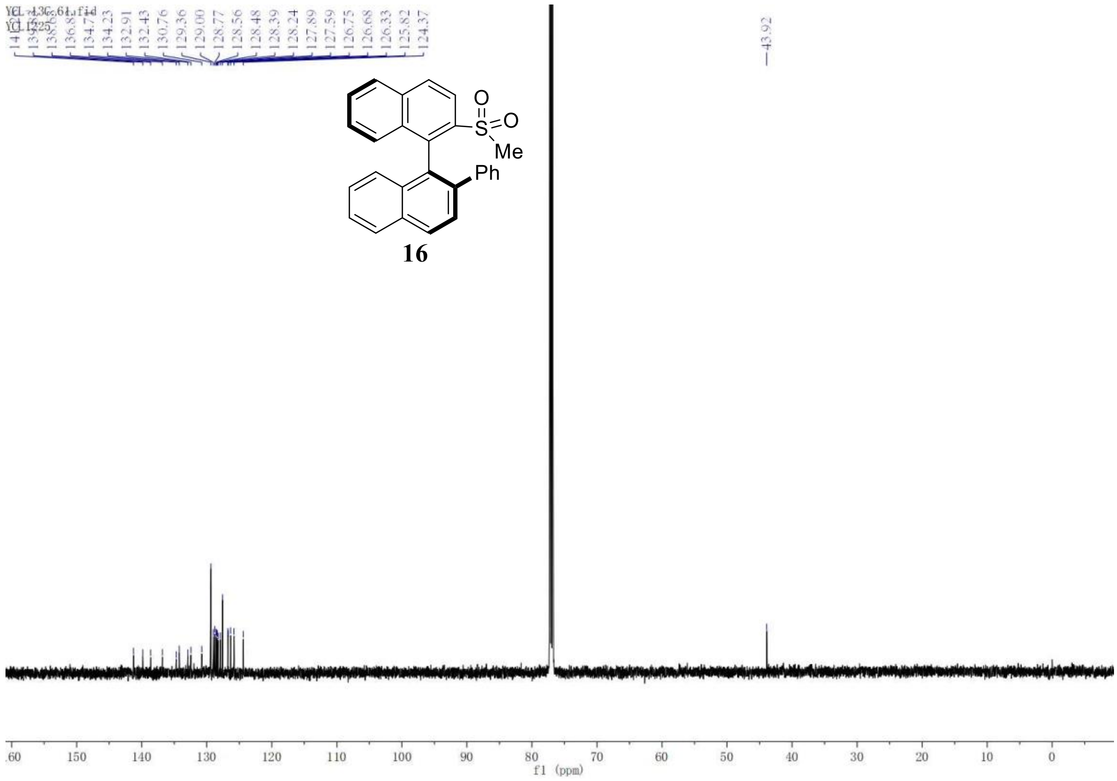

43.92
O
S
O
Me
Ph
16
f1 (ppm)
